# *Giardia* secretome highlights secreted tenascins as a key component of pathogenesis

**DOI:** 10.1093/gigascience/giy003

**Published:** 2018-01-29

**Authors:** Audrey Dubourg, Dong Xia, John P Winpenny, Suha Al Naimi, Maha Bouzid, Darren W Sexton, Jonathan M Wastling, Paul R Hunter, Kevin M Tyler

**Affiliations:** 1 NIHR Health Protection Research Unit in Gastrointestinal Infections, Norwich Medical School, University of East Anglia, Norwich Research Park, Norwich, NR4 7TJ, UK; 2 Department of Infection Biology, Institute of Infection and Global Health, Faculty of Health and Life Sciences, University of Liverpool, Liverpool Science Park IC2, 146 Brownlow Hill, Liverpool, L3 5RF, UK; 3 Department of Science and Technology, Faculty of Health and Science, James Hehir Building, Neptune Quay, University of Suffolk, Ipswich, IP4 1QJ, UK; 4 School of Pharmacy and Biomolecular Sciences, Liverpool John Moores University, Liverpool, L3 3AF, UK; 5 Comparative Biomedical Sciences, Royal Veterinary College, Royal College Street, University of London, London, NW1 0TU, UK; 6 Faculty of Natural Sciences, Keele University, Staffordshire, ST5 5BG, UK

**Keywords:** *Giardia*, secretion, proteomics, quantitative proteomics, tenascin, cysteine protease, enteric pathogen

## Abstract

**Background:**

*Giardia* is a protozoan parasite of public health relevance that causes gastroenteritis in a wide range of hosts. Two genetically distinct lineages (assemblages A and B) are responsible for the human disease. Although it is clear that differences in virulence occur, the pathogenesis and virulence of *Giardia* remain poorly understood.

**Results:**

The genome of *Giardia* is believed to contain open reading frames that could encode as many as 6000 proteins. By successfully applying quantitative proteomic analyses to the whole parasite and to the supernatants derived from parasite culture of assemblages A and B, we confirm expression of ∼1600 proteins from each assemblage, the vast majority of which are common to both lineages. To look for signature enrichment of secreted proteins, we considered the ratio of proteins in the supernatant compared with the pellet, which defined a small group of enriched proteins, putatively secreted at a steady state by cultured growing trophozoites of both assemblages. This secretome is enriched with proteins annotated to have N-terminal signal peptide. The most abundant secreted proteins include known virulence factors such as cathepsin B cysteine proteases and members of a *Giardia* superfamily of cysteine-rich proteins that comprise variant surface proteins, high-cysteine membrane proteins, and a new class of virulence factors, the *Giardia* tenascins. We demonstrate that physiological function of human enteric epithelial cells is disrupted by such soluble factors even in the absence of the trophozoites.

**Conclusions:**

We are able to propose a straightforward model of *Giardia* pathogenesis incorporating key roles for the major *Giardia*-derived soluble mediators.

## Background

With some 280 million symptomatic cases, giardiasis causes more bouts of human illness than any other parasitic disease [1]. The mechanism and mediators of pathogenesis by *Giardia*, however, remain largely unknown. Thanks to human volunteer studies, the association of *Giardia* infection itself, and the significance of the virulence of the infecting *Giardia* strain, is experimentally unambiguous [2]. The molecular definition associated with strain virulence, though, is largely unexplored. It is clear that the majority of *Giardia* infections are asymptomatic. It is also clear that infection is primarily localized to the duodenum and that some localized damage, close to the sites of colonization, causes villus atrophy and apoptosis of surrounding cells. However, this localized damage cannot be the sole cause of the profound diarrhoea that is often characteristic of the disease and that appears to affect absorption over a much wider area of the digestive tract than the site of infection alone.

One of the secreted mediators of damage to the duodenum is believed to be cathepsin B protease [3]. Cathepsin B-like proteases compose one of the superfamilies belonging to the CA clan of cysteine peptidases [4]. Compared with other cathepsins, cathepsin B proteases possess an additional 20 amino acid insertions, named the occluding loop, that enable their function as an endo- or exopeptidase [5]. Although 27 genes encoding cathepsin proteases have been identified in *Giardia*, for the majority of these proteases, function remains elusive [6]. While some parasites may secrete cathepsin B proteases to either evade or modulate their host’s immune responses [7], a recent study has demonstrated that *Giardia* trophozoites secrete cathepsin B–like proteases, degrading intestinal IL-8 and thereby reducing the inflammation reaction by the host [3]. Secreted *Giardia* cathepsin B protease (*G*CATB) may also contribute to degradation of intestinal mucin and facilitate trophozoite attachment to intestinal epithelia [8, 9].

Most of the proteomic studies so far reported for *Giardia* were undertaken in trophozoites undergoing encystation [10–12]. Only a few studies have focused on proteins secreted by *Giardia* and their role in the host-pathogen interaction [3, 13–15]. These studies were focused on parasite interaction with intestinal cell lines. No studies have yet attempted to quantify proteins that are the product of steady state secretion by healthy, growing *Giardia* trophozoites, which we hypothesize as the primary mediators of giardiasis pathology. In this study, we have identified, to the limit of existing technology, the proteins expressed by populations of healthy, growing human infective *Giardia* trophozoites. We have provided quantitation of the relative abundance of retained and released trophozoite proteins from 2 human infective assemblages, affording calculation of the specific enrichment of released proteins and thereby the description of which proteins are most likely to be secreted by trophozoites of each assemblage. Thereafter, we compared the profile of enrichment between the 2 assemblages in order to identify conserved as well as assemblage-specific secreted proteins. We provide electrophysiological analysis that confirms that trophozoite-secreted molecules adversely affect the homeostasis of enteric epithelia, and our analysis of the heterogeneity of encoding genes between lineages demonstrates the direct selective pressure on these virulence factors and affords their use in discriminating clinically important strains and outbreaks. Finally, the discovery of tenascins as a highly represented and variable group of proteins secreted by trophozoites strongly implicates this new class of virulence factors in a novel model for the mechanism of *Giardia* pathogenesis. We propose that tenascin action follows degradation of the protective mucous afforded by the action of a secreted nuclease and *G*CATB, and damage to cellular junctions by *G*CATB. Tenascins act by means of epidermal growth factor (EGF) receptor ligation, to prevent repair to those damaged junctions.

## Data Description

Soluble and cytosolic fractions from *in vitro* grown assemblage A and B trophozoites, the aetiologic agents of human giardiasis, were extracted in order to establish which proteins are secreted in the steady state by healthy, growing trophozoite populations. We reasoned that secreted proteins would be overrepresented in the medium in which parasites were incubated compared with the trophozoites that produced them. This ostensibly straightforward assessment relied on the sensitive, specific, and quantitative detection of the proteins expressed by *Giardia* trophozoites in whole cells and in the medium in which the trophozoites were incubated.

The WB (assemblage A: ATCC_50803) and GS (assemblage B: ATCC_50581) reference strains were utilized to facilitate ease of comparison between genetically divergent human infective isolates with the available reference genomes. For each experiment, trophozoites were harvested from mid–log growth and incubated in nonsupplemented Dulbecco's Modified Eagle medium (DMEM) for 45 minutes at 37°C before supernatants and pellets were collected for proteomic and other analyses including validation of their viability by flow cytometry (Additional file 1: Fig. S1). Proteomic analyses were based on samples from 3 distinct biological replicates. Each sample was analysed using 2 quantitative proteomic platforms, the Orbitrap MS and the Q-Exactive MS. Thus, in total, the results from 24 (2 × 2 × 2 × 3) proteomic analyses are reported.

The identification of abundant, secreted, *Giardia* virulence factors led us to consider whether the secretions from *Giardia* alone could affect changes in the behaviour of enteric epithelia—even in the absence of the trophozoites themselves. In order to determine the effect of *Giardia* trophozoite–secreted factors on the intestinal epithelia, chopstick-type electrodes connected to a voltmeter were used to measure the trans-epithelial electrical resistance (TEER) of polarized CaCo-2 epithelial cells grown on permeable supports. CaCo-2 cells were cultured over 6 days until confluent. TEER across the developing CaCo-2 monolayer was measured on a daily basis, as shown in Fig. 2A. Once confluence was established, *Giardia* trophozoites were added to the apical side of the confluent epithelium, and after 24 hours’ incubation, the trophozoites were washed from the apical surface. In order to determine whether co-cultures of *Giardia* trophozoites or diluted *Giardia* supernatants affected the ion channels responsible for secretory movement across the epithelium, an Ussing chamber system was utilized with different chloride secretion inhibitors and activators.

Further details about sample collection, secretome analysis, and electrophysiology can be found in the Methods section and protocols provided.

## Analyses

### Protein expression in *Giardia* trophozoites

To describe definitive *Giardia* secretomes under a standard set of conditions with high confidence and based on a robust dataset and to reduce the potential for technical artefact, the 2 MS techniques, Q-Exactive and Orbitrap MS, were used with similar settings on the same 3 independent replicates to increase coverage. Only proteins identified by both techniques within the 3 replicate datasets were included in the analysis to increase the robustness of the data. The protein quantification was performed using a label-free method: intensity-based absolute quantification (iBAQ), which calculates the sum of parent ion intensities of identified peptides per protein [16]. The average normalized abundance was divided by the iBAQ values, giving the “Abundance-iBAQ.” The quantitative datasets from both MS techniques and for each independent replicate were shown to be strongly correlated by a Spearman correlation test (data not shown) and therefore exploitable for proteomic analysis.

The Q-Exactive MS identified almost all of the proteins identified by use of the Orbitrap MS, and in total the 2 techniques identified 1587 GS proteins and 1690 WB proteins (Additional File 1: Fig. S2). This represents more than a quarter of the open reading frames predicted by the respective genomes in this single life cycle stage under this steady state set of *in vitro* culture conditions and compares favourably with other recent proteomic analyses of *Giardia* [17, 18]. Lists of proteins detected in only 1 of the 2 assemblages are provided (Additional file 2: Tables S1 and S2). Protein from 2 of the 8 predicted assemblage-specific genes previously identified by comparative genomics was detected [19].

Overall, both assemblages gave comparable and consistent results using both platforms, with the sensitivity of detection being greater for Q-Exactive MS, which provided a range of detection spanning 5 logs. In total, Q-Exactive MS identified 1542 GS proteins and 1641 WB proteins (Fig. S3). Of these, 946 GS proteins were present in both pellet and supernatant, 27 in the supernatant only, and 569 GS proteins in pellet only. By comparison, 490 WB proteins were identified in supernatant and pellet and 24 in the supernatant only, with 1127 WB proteins in pellet only.

### *Giardia* secretome

To evaluate supernatant enrichment, proteins identified in the supernatant (SP) datasets were gathered and compared with their concentration in the pellet (P) to provide a ratio using the following formula: $\frac{{SP\ abundance - iBAQ\ }}{{P\ abundance - iBAQ\ }}$. These proteins were then ranked from highest to lowest by ratiometric value, and an arbitrary cutoff was invoked such that the top 50 were considered the most likely to be secreted. Proteins identified only in SP were also included in the analysis as most likely to be secreted. All the proteins selected as “of interest” were ranked according to their SP expression from most to least abundant to obtain a quantitative enrichment profile for each isolate, and this was performed for each platform. Orbitrap and Q-Exactive enrichment profiles were compared, and proteins were considered most likely to be enriched in the supernatant when identified as such by Q-Exactive MS and confirmed by Orbitrap MS. The different enrichment profiles were then also compared between assemblages.

The results yielded a set of 15 orthologous proteins that were identified in both isolates by both techniques (Table 1). Eleven of these were predicted to possess an N-terminal signal sequence. Just 2 of these were of unknown function, and 2 groups dominated the annotated genes encoding the rest of these proteins; 5 were annotated as tenascins and 3 as cathepsin B cysteine proteases. The most abundant enriched protein was found to be pyridoxamine 5'-phosphate oxidase (PNPO), a flavin mononucleotide–dependent enzyme capable of fixing molecular oxygen that lacks a signal peptide and which was also recently identified as a secreted *Giardia* trophozoite protein upregulated during interaction with epithelial cells [15]. An extracellular nuclease was also present, along with a high-cysteine membrane protein, as well as a protein product of a gene misannotated as a variant surface protein (VSP; as it was well conserved between assemblages).

**Table 1:** The proteins which comprise the Giardia secretome ranked by abundance.

						Protein abundance			
Protein description	GI number assemblage A	GI number assemblage B	A:B identity	dN/dS	Signal peptide^b^	Pellet (P) iBAQ	Supernatant (SP) iBAQ	SP/P ratio	Abundance ranking
PNPO	GL50803_5810	GL50581_4133	99.2	0.038	NP^c^	5.71E+07	1.18E+08	2.063091	1
Tenascin	GL50803_95162	GL50581_1982	76.2	**1.597^a^**	0.99	2.97E+07	4.77E+07	1.607024	2
Tenascin	GL50803_10330	GL50581_4057	73.5	0.347	0.99	4.66E+06	2.02E+07	4.342293	3
Cathepsin B	GL50803_16468	GL50581_438	83.6	0.1072	0.78	9.63E+06	1.79E+07	1.861309	4
Tenascin	GL50803_8687	GL50581_4316	77.6	**44.176^a^**	0.98	6.22E+06	1.10E+07	1.770526	5
Uncharacterized	GL50803_5258	GL50581_2767	91.2	0.029	NP^c^	3.93E+06	1.09E+07	2.780918	6
Extracellular nuclease	GL50803_8742	GL50581_3607	83.1	0.234	1	1.03E+06	4.57E+06	4.436193	7
Tenascin-37	GL50803_16477	GL50581_3575	79.8	0.1256	0.99	8.35E+05	4.03E+06	4.830956	8
Cathepsin B	GL50803_15564	GL50581_2036	79.1	**26.5782^a^**	1	1.10E+06	3.95E+06	3.589608	9
CKS1	GL50803_2661	GL50581_3484	100	0.001	NP^c^	1.14E+06	3.20E+06	2.803062	10
Tenascin	GL50803_113038	GL50581_4180	79	0.0949	1	1.20E+06	3.14E+06	2.620931	11
HCMP Group 1	GL50803_7715	GL50581_727	67	0.1821	0.99	ND^d^	2.54E+06	∞	12
Uncharacterized	GL50803_16522	GL50581_352	76	0.1591	NP^c^	1.15E+06	2.21E+06	1.928833	13
HCMP	GL50803_12063	GL50581_2622	83	0.246	1	3.62E+05	1.94E+06	5.354665	14
Cathepsin B	GL50803_17516	GL50581_2318	72.8	0.2056	1	ND^d^	7.81E+05	∞	15

The secretome of human infective *Giardia* trophozoites of assemblage A and B have a conserved repertoire of abundant secreted factors identified by both Orbitrap MS and Q-Exactive MS. Fifteen proteins were identified as most likely to be secreted by both GS and WB isolates. Thirteen are annotated proteins, and 2 are hypothetical proteins. Proteins are ranked according to GS Q-Exactive supernatant (SP) protein abundance, from most to least abundant. Of the 12 annotated proteins, 5 are tenascins and 3 are related high-cysteine membrane proteins or VSP, and 3 are cathepsin Bs. The other annotated abundant secreted proteins are an extracellular nuclease and PNPO. Protein ranking represents the proteins’ rank within this table, from most to least abundant. Detailed breakdowns of the secretome for each assemblage by each method are provided in Supplemental Tables S1–S4.

^a^dN/dS in bold indicate that protein shows evidence of positive selective pressure during divergence from a common ancestor.

^b^Probability of N-terminal signal peptide using SignalP.

^c^Not predicted.

^d^Not detected.

We considered that where proteins were shown to be enriched in the supernatant by both platforms and in both assemblages and possessed an N-terminal signal sequence, they were truly secreted proteins. Secreted proteins involved in adapting *Giardia* to the host environment of the human gut might be expected to be engaged in Red Queen evolution and have dN/dS indicative of positive selection. While amino acid divergence between orthologs of secreted proteins varied considerably from 67% for the high-cysteine membrane protein (HCMP) to 83% for, e.g., the extracellular nuclease, only 3 proteins showed evidence of positive selections, 2 tenascins and 1 of the cathepsins. One cathepsin and 1 tenascin in particular showed evidence of evolution under a very high degree of selective pressure (Table 1). Interestingly, some cathepsins and some tenascins with similar levels of amino acid identity between the assemblages to those under high selective pressure showed little or no evidence of positive selection.

We considered whether lineage-specific soluble mediators might also be present and identified by this method, comparing those proteins identified by both methods as having the highest relative expression in the supernatant (Tables S3 and S4). The 5 most abundant conserved secreted proteins from Table 1 were also present in the top 10 secreted proteins from each assemblage amongst other VSPs, tenascins, and cathepsin B, and this regardless of the MS technique or the isolate. Unsurprisingly, VSPs were the primary proteins enriched in supernatants that were lineage-specific. Amongst the multigene families, however, there were also differences in the cathepsin B and tenascins/HCMP repertoires. No other proteins with N-terminal peptides were encoded in either assemblage except for 1 CxC-rich protein. Interestingly, none of the 8 proteins encoded by assemblage-specific genes and identified by comparative genomics was found to be enriched in the supernatants.

When comparing secretion profiles between the 2 assemblages, 7 proteins were over-represented in the supernatants by only 1 assemblage or only identified by Q-Exactive MS or present at very low abundance in 1 of the 2 (Table 2). Only 2 proteins, sentrin and A-type flavoprotein lateral transfer candidate, were present in the top 50 proteins of assemblage B (GS strain) trophozoites secretome, whereas the other 5, 1 elongation factor 1-α (EF-1α), 1 ATP-binding cassette protein 5, 1 CxC-rich protein, 1 translation initiation inhibitor, and a peptide methionine sulfoxide reductase MsrB, were present in the top 55 proteins of assemblage A (WB strain) trophozoites secretomes. Interestingly, A-type flavoprotein lateral transfer candidate was also present in the top 50 supernatant proteins by assemblage A trophozoites; however, its low supernatant enrichment ratio (<0.2) suggests that this protein is unlikely to be secreted by assemblage A trophozoites.

**Table 2:** Assemblage specific component proteins of the *Giardia* secretome.

				Protein abundance							
				Assemblage A			Assemblage B			Abundance ranking in secretome	
Protein description	GI number assemblage A	GI number assemblage B	Signal peptide^a^	Pellet (P) iBAQ	Supernatant (SP) iBAQ	SP/P ratio	Pellet (P) iBAQ	Supernatant (SP) iBAQ	SP/P ratio	Assemblage A	Assemblage B
*A-type flavoprotein lateral candidate*	*GL50803_10358*	*GL50581_1626*	*NP* ^c^	*2.11E+07*	*3.70E+06*	*0.17546*	*4.35E+06*	*8.70E+06*	*2.001741*	*47*	*10*
*Sentrin*	*GL50803_7760*	*GL50581_3210*	*NP* ^c^	*1.44E+05*	*ND* ^c^	*ND* ^c^	*ND* ^c^	*3.67E+04*	∞	–	*31*
**EF-1α**	**GL50803_112312**	–	**NP^c^**	**ND^c^**	**2.22E+06**	∞	–	–	–	**17**	–
**ATP-binding cassette** **protein 5**	**GL50803_8227**	**GL50581_3399**	**NP^c^**	**2.93E+06**	**2.89E+06**	**0.98546**	**2.01E+05**	**1.43E+04**	**0.071052**	**15**	**897**
**CxC rich protein**	**GL50803_17476**	**GL50581_4509**	**1**	**4.80E+05**	**2.83E+05**	**0.58945**	**4.35E+04**	**3.59E+04**	**0.823376**	**43**	**819**
**Peptide methionine sulfoxide reducast MsrB**	**GL50803_5180**	**GL50581_3084**	**NP^c^**	**1.24E+05**	**8.80E+04**	**0.70952**	**1.09E+06**	**1.30E+06**	**1.19568**	**53**	**331**
**Translation initiation inhibitor**	**GL50803_480**	**GL50581_4017**	**NP^c^**	**9.47E+06**	**4.08E+06**	**0.43038**	**1.06E+07**	**9.76E+06**	**0.920148**	**15**	**95**

Human infective *Giardia* trophozoites of assemblage A and B secrete a small set of different proteins. Seven proteins were identified as the most likely to be secreted by either GS or WB isolates. Two are the most likely to be secreted by GS isolate (shown in italics), and 5 are the most likely to be secreted by WB isolate (shown in bold). One GS isolate secreted and 2 WB isolates secreted were identified only via Q-Exactive MS in the other assemblage's dataset (shown in underline). The abundance ranking represents the protein ranking within the secretome of both assemblages according to their abundances in the supernatant.

^a^Probability of N-terminal signal peptide using SignalP.

^b^Not predicted.

^c^Not detected.

### *Giardia* soluble mediators disrupt intestinal cell functions

Soluble and diffusible agents, able to disrupt gut function, could potentially mediate more diffuse and profound pathology for giardiasis than close range interactions between the trophozoites and the gastrointestinal epithelium alone. To determine whether *Giardia*-secreted virulence factors could induce changes in the behaviour of the intestinal epithelium, short-circuit current (Isc) was continuously measured across polarized CaCo-2 epithelial cells that had either been cultured without any additions, co-cultured with *Giardia* trophozoites, or co-cultured with diluted (1:1000) *Giardia* supernatants (Fig. 2B). Further experiments demonstrated that either after 24-hour co-culture with *Giardia* (Fig. 2C) or 24-hour co-culture with diluted *Giardia* supernatants (Fig. 2D), both experimental conditions dramatically inhibit both the cAMP-stimulated Isc (basolateral application of 10 μM of forskolin) and the calcium-activated Isc (basolateral application of 100 μM of UTP). In order to identify which ion channels were being affected, the CFTR chloride ion channel inhibitor, GlyH101 (50 μM), and the calcium-activated chloride ion channel inhibitor, DIDS (100 μM), were added to the apical side of the Ussing chamber. The cAMP-stimulated Isc is predominantly due to activation of CFTR chloride channels as it is inhibited by GlyH101 (Fig. 2B–D). The calcium-activated Isc is predominantly due to activation of calcium-activated chloride channels as it is inhibited by DIDS (Fig. 2B–D).

## Discussion

In this study, we have identified proteins secreted by trophozoites of both human-infecting assemblages. Contaminating host serum proteins (mainly bovine albumin) in the supernatant samples were a concern, as previously described by others [20]. Such serum proteins bind to the parasite's surface and are continuously released, which interferes with the characterization of *Giardia* secretome. To overcome this issue, parasites were cleansed from the serum proteins and incubated in serum-free DMEM before collecting supernatants and pellets. To increase the coverage and robustness of the analysis, 2 mass spectrometers (Orbitrap and Q-Exactive MS) were used on the same replicates, and proteins identified by both MS were included in the analysis.

Previous studies have focused on protein secretion during *Giardia* trophozoite encystation; or protein secretion upon interaction with (or attachment to) host cells. Here instead, we chose to provide a detailed baseline from cultured *Giardia* trophozoites secreting proteins under a steady state *in vitro*. Nevertheless, our results are strongly supportive of a recent proteomic study looking at the effect of host attachment on the profile of *Giardia*-secreted proteins [15]. Prior to that study, several metabolic enzymes had been proposed to be released by *Giardia* trophozoites upon interaction with intestinal epithelial cells (IECs) [13], such as arginine deiminase (ADI), enolase, and ornithine carbamoyltransferase (OCT), which we were also able to identify from the culture supernatants of both assemblages.

Our study does confirm the previously observed enrichment of EF-1α in assemblage A culture supernatants (Table 2; Table S4) [20]. EF-1α is a key enzyme in the protein synthesis process in eukaryotic cells [21], but many organisms have been shown to express EF-1α in excess, which suggests that this protein may have some other functions [21]. In the context of pathogenicity and virulence, the secreted *Leishamnia* EF-1α has been shown to downregulate host inflammatory cell signalling [22]. In *Giardia*, EF-1α has been shown to be an immunoreactive protein recognized by antibodies from patients who have previously had giardiasis [20]. Yet its role as putatively secreted virulence factor in *Giardia* pathogenesis remains elusive. That this protein is only released by assemblage A trophozoites raises the possibility of associating its function with observable differences in pathogenesis or host range between the 2 human infective assemblages.

Our study shows some other differences in secretions between assemblage A and B trophozoites (Table 2). A-type flavoprotein lateral transfer candidate and sentrin were present in assemblage B (GS strain) trophozoites secretome; ATP-binding cassette (ABC) protein 5, CxC-rich protein, translation initiation inhibitor, and peptide methionine sulfoxide reductase (MsrB) were present in assemblage A (WB strain) secretome.

A-type flavoprotein lateral transfer candidate has a high oxygen reductase activity during *Giardia* infection, suggesting an O_2_ scavenging function upon release in the host intestinal environment [23], thus potentially affording increased resilience to *Giardia* trophozoites in the small intestine and manipulating the parasites’ immediate microenvironment. Whether assemblage B trophozoites require A-type flavoprotein lateral transfer candidate throughout the infection or just in its early stage remains unclear. Sentrin is involved in the ubiquitination of proteins to render them resistant to degradation [24]. Sentrin is evolutionarily conserved and has been identified in prokaryotic and eukaryotic organisms such as *S. cerevisiae, A. thaliana*, and *Homo sapiens*, which suggests a conserved specialized function in cell metabolism [24]. With its ubiquitination function, sentrin was expected to be only present in *Giardia* proteome but not in its secretome. Why this protein would be secreted or released by *Giardia* trophozoites remains unclear and raises the question of the advantages, for the parasite, of releasing sentrin into the host environment upon infection.

ABC proteins are a large and diverse canonical group of membrane proteins typically resident in the plasma membrane and associated, in eukaryotes, with the ATP-dependent egress of metabolites and toxins; they can be determinants of virulence and drug resistance [25]. Here 1 *Giardia* ABC protein shows enrichment in the supernatant of WB but not of GS, and it will be interesting to see if a functional correlation can be found. The CxC-rich protein belongs to the HCMP superfamily, which also includes VSPs, tenascins, and HCMPs. The presence of orthologs in both strains is consistent with it not being a VSP protein. As with several other HCMPs, this CxC-rich protein had a very high signal and only 1 TM domain suggesting that it may be a labile surface protein in WB, but its specific role and why it is much more abundant in the WB supernatant than the GS supernatant is not clear. Translation initiation inhibitors are proteins inhibiting the initiation of the translation of messenger RNA (mRNA) into proteins and are mainly located in the cell cytosol [26]. Yet, 1 translation initiation inhibitor is over-represented in the assemblage A trophozoite secretome (top 20 secreted proteins), probably due to its high solubility and stability. Peptide methionine sulfoxide reductase (MsrB) catalyzes the reduction of free- and protein-bound methionine sulfoxides to corresponding methionines, which constitutes a mechanism for the scavenging of reactive oxygen species (ROS) responsible for a fundamental innate defence against pathogens in various host organisms [27]. MsrB is an antioxidant protein protecting organisms from the cytotoxic effects of ROS and therefore from cell death. This protein is crucial for the virulence of *S. typhimurium* and the immune evasion of *Schistosoma mansoni* [28, 29]. Whether msrB has a similar role in *Giardia* assemblage A pathogenicity remains unclear.

The difference in secretion between the 2 human infective assemblages observed in this study may also go some way to explaining the differences in pathogenesis, symptoms, and host range previously observed between assemblage A and B.

The most abundant proteins, in both human isolates, primarily belong to 4 families of proteins: GCATB, high-cysteine membrane proteins, variant surface proteins, and tenascins.

**Figure 1:**
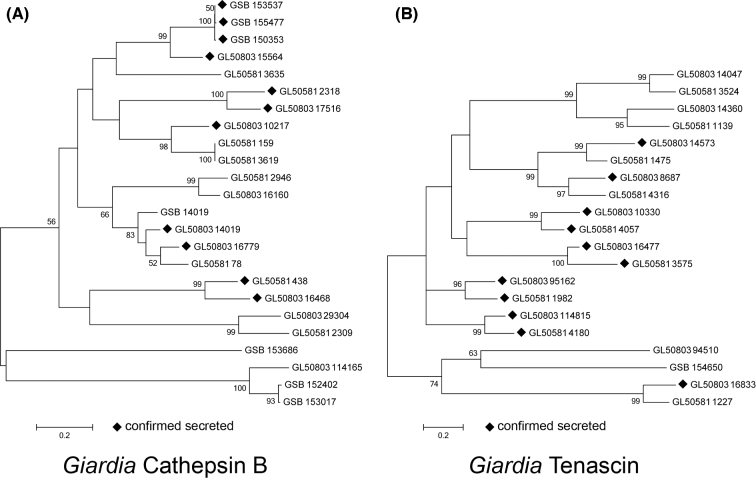
Neighbour joining tree showing clustering of (A) cathepsin B and (B) tenascin gene families. Genes were retrieved by gene name search on *Giardia*DB. Gene sequences were downloaded and aligned using ClustalW generated with the MEGA 6 software package. Maximum composite likelihood method was used, with 2000 bootstrap replicates. Bootstrap values greater than 50% are shown above the branches. ♦Proteins confirmed to be secreted using our proteomic analysis.

The cathepsin B family of *Giardia* are confirmed virulence factors involved in many of the parasite's processes such as encystation and excystation [6]; secreted GCATBs degrade host IL-8 and inhibit neutrophil chemotaxis [3]. GCATB contains secreted and nonsecreted trophozoite-expressed proteins; the orthologues of which are predominantly common to GS (B) and WB (A) assemblages (Fig. 1). Expression of 16 *G*CATBs was proteomically confirmed, of which 11 were shown by our proteomic analysis to be secreted. These 11 fell into 6 orthologous groups, and for 3 of these groups, all group members were shown to be secreted. Secreted *G*CTAB GL50803_15564 (WB) and its ortholog GL50581_2036 (GS) show dN/dS values of >26, indicative of strong positive selective pressure. Interestingly, when GS was resequenced, GL50803_15564 was found to comprise 3 recently diverged orthologs (GSB_153537, GSB_155477, GSB_150353), and it may be that the positive selection pressure observed has been generated as a result of recent gene duplications in the assemblage B strain. GL50803_16779, an assemblage A (WB) GCATB, has previously been shown to be upregulated and involved in trophozoite motility in the early pathogenesis of *Giardia* [15]. In this study, this protein was found to be in WB’s top 5 secreted proteins (Table S4); its GS ortholog (GL50581_78) was also present but at a considerably lower level, suggesting that for this GCATB may play a more significant role in assemblage A than assemblage B.

HCMPs are an enigmatic group of proteins with few associated functional studies. They may protect trophozoites against proteolysis [30, 31] and oxidative damage [32]. In *Giardia*, it appears that 1 lineage of HCMPs has given rise to the VSPs, while another has given rise to a group with high homology to mammalian tenascins. Tenascin, VSPs, and HCMPs are then related multigene families that together form the largest group of proteins enriched in the *Giardia* supernatants. Interestingly, when aligned and analysed phylogenetically, the secreted tenascins segregate into a monophyletic group (Fig. S4). Both WB and GS orthologs of 5 tenascin gene products were secreted, and in WB, 2 other secreted tenascins were also detected that were not detected for the GS strain (Fig. 1B).

VSPs are well-characterized surface glycoproteins with transmembrane domains, which are expressed one at a time by *Giardia* trophozoites through an RNAi-regulated mechanism. They are quintessential virulence factors, responsible for antigenic variation. VSPs are hypervariable by nature, and thus it is to be expected that they do not form orthologous pairs. This was the case for most we observed; intriguingly though, a few proteins annotated as VSPs were conserved between isolates, suggesting that they are not actually VSPs and would not be subject to “one at a time” controlled expression—but are actually misannotated HCMPs, which may have a conserved function in both GS and WB isolates. This study was not able to resolve whether the enrichment of such proteins in the supernatant observed is due to clipping or shedding from the parasite surface or whether the proteins are also secreted.

Tenascins are characterized by the presence of EGF repeats and are able to act as ligands for EGF receptors. Mammalian tenascins are extracellular matrix proteins, which modulate cell adhesion and migration [33]. They appear to have evolved from a group of proteins specific to vertebrates, presumably co-evolving with the EGF receptor, and so the presence of homologous proteins in *Giardia* evolving independently from HCMPs is a clear example of the kind of convergent evolution best described as molecular mimicry. Interestingly, a *Giardia* tenascin (WB-GL50803_8687/GS-GL50581_4316), secreted by both strains, and *Giardia* tenascin (WB-GL50803_14573/GS-GL50581_1475), secreted only by the WB strain (Table 1; Table S4), were found to be induced by host soluble factors and implicated in the regulation of trophozoite attachment [15], supporting the case for secreted tenascins acting as virulence factors in *Giardia* pathogenesis.

**Figure 2:**
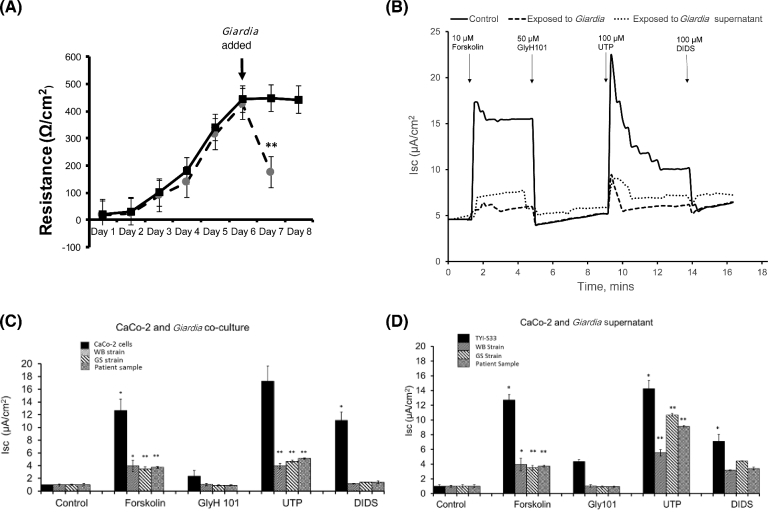
The effect of co-culture with *Giardia* or *Giardia* supernatants on the electrophysiological properties of CaCo-2 monolayers. (A) Transepithelial electrical resistance in CaCo-2 monolayers following seeding on permeable supports. Data show an increase in TEER as the monolayer develops. Confluence occurred around day 6. *Giardia* were added on day 6 after the confluent monolayer formed and co-cultured with the Caco-2 monolayer for 24 hours. TEER was measured after 24 hours and compared with TEER in monolayers that had not been exposed to *Giardia* (n = 6). (B) A representative short circuit current (Isc) against time recording from single monolayers of CaCo-2 cells in an Ussing chamber. The trace shows the activation of CFTR chloride channels (basolateral application of 10 μM of forskolin) and calcium-activated chloride channels (basolateral application of 100 μM of UTP). Specificity of activation is confirmed by inhibition of Isc by the specific CFTR channel blocker, GlyH101; and specific calcium-activated chloride channel blocker, DIDS. The effect on Isc of 24-hour co-incubation of CaCo-2 monolayers with *Giardia* or with *Giardia* supernatant (1:1000 dilution) is also shown. (C) Effect of 24-hour co-incubation of CaCo-2 monolayers with different strains of *Giardia* (WB, GS, and patient samples) on forskolin-stimulated and UTP-stimulated Isc (n = 3). (D) Effect of supernatant co-incubation from different strains of *Giardia* (WB, GS, and patient samples) on forskolin-stimulated and UTP-stimulated Isc (n = 3) from Caco-2 monolayers. The results were analysed by the Student *t* test and expressed as mean values ± standard error mean (SEM). Significant difference expressed as *P < 0.05, **P < 0.01 compared with control.

Most published studies concerning host cell–*Giardia* interactions have focused on the effects on the host intestinal epithelia upon attachment of the trophozoites to the cells. In this study, we have shown that diluted supernatant obtained from the steady growth of *Giardia* trophozoites *in vitro* has an effect on the intestinal cell function. The effect observed on chloride secretion by *Giardia* supernatants indicates that *Giardia* secretes a soluble factor, which is likely affecting secretion across the intestinal epithelial cells. Physiologically, cultured intestinal cells show sensitivity to *Giardia* proteins released by the parasite even at high dilution. Fig. 2D demonstrates that intestinal epithelial cells when acutely exposed to such *Giardia* proteins lose the ability to stimulate CFTR and calcium-activated chloride channels, the clear implication being that virulence determinants released from *Giardia* trophozoites interact with epithelial cell receptors and ion channels.

In this analysis, we have identified the proteins that are secreted by human infective *Giardia* trophozoites. Just 2 groups form the majority of these proteins, *G*CATBs and the HCMP superfamily, encoding known virulence factors in addition to an abundant extracellular nuclease and an oxygen-fixing enzyme. The elucidation of this group of proteins dramatically increases our understanding of the pathogenic mechanisms underlying giardiasis at a molecular level. The genes encoding GCATBs and HCMP superfamily proteins are among the most heterogeneous of all genes between assemblages. Their probable role in interaction with the host and luminal environment is supported by the very high dN/dS values of some family members. Correlation of variation within assemblages at these loci with strain virulence is the essential next step for their use in the diagnosis of virulent strains, risk assessment, and disease prognosis.

**Figure 3:**
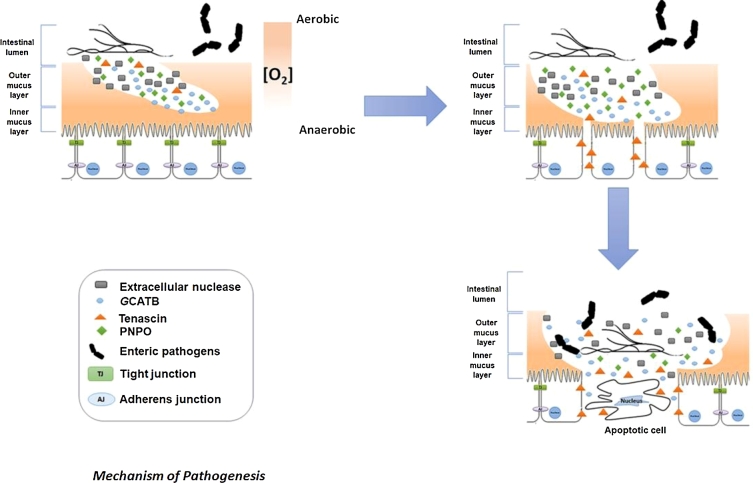
Proposed novel mechanism of pathogenicity for *Giardia* involving PNPO, extracellular nuclease, *G*CATB, tenascin. PNPO (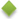) renders the intestinal environment more favourable to trophozoite's growth. Once a new *Giardia* colony is established, trophozoites release extracellular nuclease (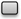), GCATB (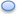), and tenascin (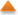). Extracellular nuclease may contribute to reducing the viscosity of the intestinal outer mucus layer, while *G*CATB may degrade mucins and disrupt intracellular junction. Finally, tenascins may maintain intestinal cells apart by attaching to the EGF receptors present at the surface of intestinal cells that could, over time, lead to the apoptosis of these isolated intestinal cells.

Our results indicate that *Giardia* secretions are sufficient to disable normal function in enteric epithelial cells, making them less able to extract fluids from the lumen. In particular, they implicate PNPO, an extracellular nuclease, *G*CATBs, and tenascins. The fact that both extracellular nuclease and *G*CATBs can be involved in the degradation of the intestinal mucus layer and that both *G*CATBs and tenascins can be associated with intestinal intracellular junction disruption suggests collaboration between these proteins. Therefore, we propose a pathogenic mechanism (Fig. 3) whereby PNPO produces a reducing environment favouring growth of trophozoites and the extracellular nuclease degrades the outer layer of the intestinal mucus, improving access for *G*CATBs for further degradation of the protective mucous barrier and subsequent disruption of intestinal intracellular junctions. Lastly, tenascins are involved in maintaining intestinal cell separation by ligation of EGF receptors present at the surface of intestinal cells and exacerbation of epithelial damage via increased levels of apoptosis amongst these more detached cells. Once the intestinal barrier is breached by the actions of *Giardia*-secreted virulence factors, the sites of damage become prone to secondary infection by other opportunist microbes resident in the intestinal lumen and sensitive to irritation by allergens in foodstuffs, leading to further inflammation and to the characteristic symptoms of the disease. Further investigations are necessary to verify this proposed mechanism of the pathogenesis of giardiasis.

## Methods

### Proteomic analysis

#### Sample preparation

*Giardia* trophozoites from the genome reference strains WB (assemblage A, ATCC_50803) and GS (assemblage B, ATCC_50581) were cultured in TYI-S-33 under standard conditions (5% CO_2_) [34] and harvested during the mid–log phase of their *in vitro* growth curves. The total trophozoites (adhered and nonadhered) were washed ×3 in phosphate buffer saline (PBS) and then incubated in nonsupplemented DMEM, with antibiotics to conserve an axenic milieu, for 45 minutes at 37°C (Fig. S5A) [38]. After incubation, an aliquot was analysed by flow cytometry to evaluate the viability of the *Giardia* samples. Trophozoites and supernatant were separated by centrifugation, and both trophozoite pellet and supernatant were harvested. Proteins contained in supernatant were concentrated in Vivaspin columns (3000 MWCO) with 25 mM of ammonium bicarbonate (Ambic) (Fig. S5 B) [39]. Supernatants were analysed by SDS PAGE [40] and were tested on cultured epithelial cells (Caco-2) to ensure the presence of proteins and biological activity (see below). Supernatants and pellets were sent to the Institute of Infection and Global Health at the University of Liverpool for mass spectrometry analysis (Fig. S3) [41].

Protein samples were dispensed into low protein-binding microcentrifuge tubes (Sarstedt, Leicester, UK) and made up to 160 μl by addition of 25 mM of Ambic. The proteins were denatured using 10 μl of 1% (w/v) RapiGest^TM^ (Waters MS Technologies, Manchester, UK) in 25 mM of Ambic, followed by 3 cycles of freeze-thaw and 2 cycles of 10 minutes’ sonication in water bath. The sample was then incubated at 80°C for 10 minutes and reduced (addition of 10 μl of 60-mM DTT and incubation at 65°C for 10 minutes) and alkylated (addition of 10 μl of 180-mM iodoacetamide and incubation at room temperature for 30 minutes in the dark). Trypsin (Sigma-Aldrich, Dorset, UK) was reconstituted in 50 mM of acetic acid to a concentration of 0.2 μg/μl. Digestion was performed by the addition of 10 μl of trypsin to the sample, followed by incubation at 37°C overnight. The RapiGest^TM^ was removed from the sample by acidification (1 μl of trifluoroacetic acid and incubation at 37°C for 45 minutes) and centrifugation (15 000 × *g* for 15 minutes) [41]. After protein digestion, 1 μg of digest was injected into both the Orbitrap Velos and the Q-Exactive MS for all samples.

#### Orbitrap Velos

Peptide mixtures were analysed by online nanoflow liquid chromatography using the nanoACQUITY-nLC system (Waters MS technologies, Manchester, UK) coupled to an LTQ-Orbitrap Velos (ThermoFisher Scientific, Bremen, Germany) mass spectrometer equipped with the manufacturer's nanospray ion source. The analytical column (nanoACQUITY UPLC^TM^ BEH130 C18 15 cm × 75 μm, 1.7-μm capillary column) was maintained at 35°C and a flow rate of 300 nl/min. The gradient consisted of 3%–40% acetonitrile in 0.1% formic acid for 90 minutes, then a ramp of 40%–85% acetonitrile in 0.1% formic acid for 3 minutes. Full scan MS spectra (m/z range 300–2000) were acquired by the Orbitrap at a resolution of 30 000. Analysis was performed in data-dependant mode. The top 20 most intense ions from the MS1 scan (full MS) were selected for tandem MS by collision-induced dissociation (CID), and all product spectra were acquired in the LTQ ion trap. Ion trap and orbitrap maximal injection times were set to 50 ms and 500 ms, respectively.

#### Q-Exactive MS

Digests (2 μl) were analysed on a 50-cm Easy-Spray column with an internal diameter of 75 μm, packed with 2-μm C18 particles, fused to a silica nano-electrospray emitter (Thermo Fisher Scientific). Reversed phase liquid chromatography was performed using the Ultimate 3000 nano system with a binary buffer system consisting of 0.1% formic acid (buffer A) and 80% acetonitrile in 0.1% formic acid (buffer B). The peptides were separated by a linear gradient of 5%–40% buffer B over 110 minutes at a flow rate of 300 nl/min. The column was operated at a constant temperature of 35°C, and the LC system coupled to a Q-Exactive mass spectrometer (Thermo Fisher Scientific). The Q-Exactive was operated in data-dependent mode with survey scans acquired at a resolution of 70 000 at m/z 200. Up to the top 10 most abundant isotope patterns with charge states +2, +3, and/or +4 from the survey scan were selected with an isolation window of 2.0 Th and fragmented by higher-energy collisional dissociation with normalized collision energies of 30. The maximum ion injection times for the survey scan and the MS/MS scans were 250 and 100 ms, respectively, and the ion target value was set to 1E6 for survey scans and 1E4 for the MS/MS scans. Repetitive sequencing of peptides was minimized through dynamic exclusion of the sequenced peptides for 20 seconds.

#### Data analysis

Thermo RAW files were imported into Progenesis LC–MS (version 4.1, Nonlinear Dynamics, Newcastle upon Tyne, UK). Replicate runs were time-aligned using default settings and an auto-selected run as a reference. Peaks were picked by the software using default settings and filtered to include only peaks with a charge state of between +2 and +6. Peptide intensities of replicates were normalized against the reference run by Progenesis LC-MS. Spectral data were transformed to .mgf files with Progenesis LC-MS (Liquid Chromatography-Mass Spectrometry) and exported for peptide identification using the PEAKS Studio 7 (Bioinformatics Solutions Inc., Waterloo, Canada) search engine. A multiple–search engine platform provided by PEAKS Studio named inChorus was used, which combines searching results from PEAKS DB (Bioinformatics Solutions Inc.), Mascot (Matrix Science, London, UK), OMSSA (National Center for Biotechnology Information, Bethesda, USA), and X! Tandem (Global Proteome Machine Organization). Tandem MS data were searched against a custom database that contained the common contamination and internal standards GiardiaDB-3.1_GintestinalisAssemblageA_AnnotatedProteins and GiardiaDB-3.1_GintestinalisAssemblageB_AnnotatedProteins. The search parameters for Orbitrap-Velos were as follows; precursor mass tolerance was set to 10 ppm, and fragment mass tolerance was set to 0.5 Da. One missed tryptic cleavage was permitted. Carbamidomethylation was set as a fixed modification, and oxidation (M) set as a variable modification. The search parameters for Q Exactive were as follows; precursor mass tolerance was set to 10 ppm, and fragment mass tolerance was set to 0.01 Da. One missed tryptic cleavage was permitted. Carbamidomethylation was set as a fixed modification, and oxidation (M) set as a variable modification. The false discovery rates (FDRs) were set at 1%, and at least 2 unique peptides were required for reporting protein identifications. Protein abundance (iBAQ) was calculated as the sum of all the peak intensities (from Progenesis output) divided by the number of theoretically observable tryptic peptides [16]. Protein abundance was normalized by dividing the protein iBAQ value by the summed iBAQ values for that sample. The reported abundance is the mean of the biological replicates.

The mass spectrometry proteomics data have been deposited to the ProteomeXchange Consortium via the PRIDE partner repository [24] with the dataset identifier PXD004398 and 10.6019/PXD004398.

### Electrophysiology

#### Giardia trophozoites culture

*Giardia lamblia* WB and GS strains as well as the patients’ strains (obtained from 3 patients with giardiasis from the NNUH) were grown in filter-sterilized, modified TYI-S-33 medium with 10% adult bovine serum and 0.05% bovine bile [28] at 37°C in microaerophilic conditions and subcultured when confluent. To collect parasites for experiments, the medium was removed from the culture to eliminate unattached or dead parasites. The tube was refilled with cold, sterile medium, and trophozoites detached by chilling on ice for 15 minutes.

Parasites were collected by centrifugation (1500 × g for 5 minutes at 4°C) and washed once with the plating medium of 90% complete DMEM/10% *Giardia* medium. Parasites were then counted using a haemocytometer and diluted to the appropriate number.

To collect *Giardia* supernatant for experiments, the *Giardia* culture bottle was placed on ice for 15 minutes. The bottle then underwent centrifugation (1500 × g for 5 minutes at 4°C). The supernatant was then collected and filtered 3 times using 15-mm diameter syringe filters (0.2-μm pore size). Subsequently, the postfiltered *Giardia* supernatant was diluted 1:1000 and saved in a –20°C freezer until required.

#### Mammalian cell line (CaCo-2) preparation

CaCo-2 cells (passages 20–25) were grown in DMEM supplemented with nonessential amino acids, penicillin (12 IU/ml), streptomycin (12 μg/ml), gentamycin (47 μg/ml), and 20% (vol/vol) heat-inactivated fetal calf serum (all from AMIMED, Bioconcept). The cells were seeded at a density of 6 × 10^4^ cells/cm^2^ in 6-well Transwell filters (0.4-μm pore size) and cultured for 7–15 days until confluent. Confluent monolayers were then used for electrophysiological experiments, for co-culture experiments with *Giardia* parasites, or for culture with *Giardia* supernatants [42].

#### CaCo-2 co-culture experiments with Giardia or Giardia supernatant

Confluent CaCo-2 monolayers were taken, and the CaCo-2 cell media was removed and replenished with a combination of 90% complete DMEM/10% *Giardia* medium plus or minus *Giardia* trophozoites (100 000 total parasites per insert). Control cultures were maintained in a separate plate to prevent parasite contamination. Control inserts were inspected under the microscope to ensure there was no *Giardia* cross-contamination. The co-cultures were incubated at 37°C and 5% CO_2_ for 24 hours, after which the *Giardia* parasites were removed [42].

Confluent Caco-2 monolayers were also cultured with diluted (1:1000) *Giardia* supernatants for 24 hours. Briefly, the culture media was removed from the insert, and Caco-2 cell media was replaced with a combination of 99.9% complete DMEM/0.1% *Giardia* medium plus or minus *Giardia* supernatant [42].

#### Transepithelial electrical resistance assay

Monolayers of CaCo-2 cells were grown on 6-well Transwell filters (0.4-μm pore size) for 7–15 days until confluent. The development of the polarized monolayer was assessed by measuring the transepithelial electrical resistance (TEER) over a 7–15-day period. Once confluent, *Giardia* were added to the apical side of the Transwell filter and incubated for 24 hours. The integrity of the confluent polarized monolayer was assessed by measuring the TEER before and/or after apical infection by *Giardia* [42].

#### Electrophysiology assay

Monolayers of CaCo-2 cells on Transwell filters were mounted into a Physiological Instruments EM-CSYS-2 Ussing chamber setup after establishment of a confluent monolayer, and the short circuit current (I_SC_) across the monolayer was continuously measured [42].

Both sides of the epithelium were bathed in 5 ml of Krebs Henseleit solution, which was continuously circulated through the half chambers, maintained at 37°C, and continuously bubbled with 95% O_2_/5% CO_2_. The composition of the Krebs Henseleit bath solution used was similar to that used by Cuthbert [35] and had the following composition (in mM): NaCl 118, KCl 4.7, CaCl_2_ 2.5, MgCl_2_ 1.2, NaHCO_3_ 25, KH_2_PO_4_ 1.2, and glucose 11.1 (pH 7.4). The permeable supports were left for 30 minutes to equilibrate before experiments were started. All filters were treated with 10 μM of amiloride apically to eliminate electrogenic sodium absorption through epithelial sodium channels [42].

#### Data analysis

I_SC_ was continuously monitored across the monolayers by a Physiological Instruments Multichannel Voltage/Current Clamp (VCC MC6) through 3M KCl/agar, Ag/AgCl_2_ cartridge electrodes (Physiologic Instruments), and the raw data for I_sc_, transepithelial resistance, and transepithelial voltage were recorded using Acquire and Analyse version 1.3 software (Physiological Instruments). Data were exported to Microsoft Excel initially and then into the GraphPad Prism version 5.0 for Windows package for data representation and statistical analysis.

#### Chemicals and Inhibitors

Forskolin (10 μM), UTP (100 μM), Amiloride (10 μM), and DIDS (100 μM) were obtained from Sigma Aldrich, and GlyH-101 (50 μM) was obtained from Merck Chemicals. Stock solutions of Amiloride (10 mM) and GlyH-101 (50 mM) were made by dissolving in DMSO. Final concentrations of drugs are as indicated in the text or figures and where produced by adding the appropriate volume of stock concentration to 5 ml of either the basolateral or apical bathing solution.

### Phylogeny

To look for sequence similarities between proteins of interest from the same protein family, the coding sequences of these proteins were retrieved from *Giardia*DB (v 3.1, 4.0, and 5.0), aligned, and compared using ClustalW.

Phylogenetic trees were built for these proteins via the maximum likelihood approach using MEGA software (v. 6.06).

## Availability of supporting data

All proteomic datasets are held by and can be accessed for free at the European Bioinformatics PRoteomics IDEntifications (PRIDE) database (accession number PXD004398). Free integrated functionality with other *Giardia* large datasets is hosted at EupathDB [36]. Supporting data, including raw data in .csv format, alignments, and phylogenetic analyses, are also available via the *GigaScience* repository, *Giga*DB [37]. All protocols used in this study are available and can be accessed at the protocols.io database [38–43].

## Additional files

Table S1: List of the *Giardia* assemblage A (WB strain) lineage-specific proteins identified via Orbitrap and Q-Exactive MS. Protein sequences were compared with their coding sequence and matched to their orthologs in assemblage B (GS strain) using the *Giardia* database: *Giardia*DB.org. Annotated proteins are highlighted in red, and hypothetical proteins in blue. Proteins were ranked according to Q-Exactive S supernatant (S) abundance, from most to least abundant.

Table S2: List of the *Giardia* assemblage B (GS strain) lineage-specific proteins identified via Orbitrap and Q-Exactive MS. Protein sequences were compared with their coding sequence and matched to their orthologs in assemblage A (WB strain) using *Giardia*DB.org. Annotated proteins are highlighted in red, and hypothetical proteins in blue. Proteins were ranked according to Q-Exactive abundance (S), from most to least abundant.

Table S3: List of the 86 proteins most likely to be secreted by *Giardia* GS strain trophozoites. Thirty-one proteins were identified via Orbitrap and Q-Exactive MS (in bold), and 55 proteins were identified via Q-Exactive MS only (in italics). Fifty-nine proteins are annotated (shown in red), and 27 are hypothetical proteins (shown in blue). Ten proteins are lineage-specific. The 15 proteins identified as conserved between the 2 isolates are highlighted in grey. Only proteins identified via both techniques were considered secreted and were included in the final analysis. Proteins are ranked according to Q-Exactive SP abundance from most to least abundant.

Table S4: List of the 61 proteins most likely to be secreted by *Giardia* WB strain trophozoites. Forty-4 proteins were identified via Orbitrap and Q-Exactive MS (in bold), and 16 proteins were identified via Q-Exactive MS only (in italics). Fifty-three proteins are annotated (shown in red), and 8 are hypothetical proteins (shown in blue). Twelve proteins are lineage-specific. The 15 proteins identified as conserved between the 2 isolates are highlighted in grey. Only proteins identified via both techniques were considered secreted and were included in the final analysis. Proteins are ranked according to Q-Exactive supernatant (S) abundance from most to least abundant.

Table S5: List of the 1553 proteins identified in the pellet of *Giardia* GS strain trophozoites via Q-Exactive and Orbitrap MS. Hypothetical and annotated proteins are shown in blue and black, respectively. Proteins are ranked according to their Q-Exactive protein abundance–iBAQ values from least to most abundant.

Tables S6: List of the 996 proteins identified in the supernatant of *Giardia* GS strain trophozoites via Q-Exactive and Orbitrap MS. Hypothetical and annotated proteins are shown in blue and black, respectively. Proteins are ranked according to their Q-Exactive protein abundance–iBAQ values, from least to most abundant.

Table S7: List of the 1657 proteins identified in the pellet of *Giardia* WB strain trophozoites via Q-Exactive and Orbitrap MS. Hypothetical and annotated proteins are shown in blue and black, respectively. Proteins are ranked according to their Q-Exactive protein abundance–iBAQ values from least to most abundant.

Table S8: List of the 558 proteins identified in the supernatant of *Giardia* WB strain trophozoites via Q-Exactive and Orbitrap MS. Hypothetical and annotated proteins are shown in blue and black, respectively. Proteins are ranked according to their Q-Exactive protein abundance–iBAQ values from least to most abundant.

Figure S1: *Giardia* trophozoites are viable after incubation in nonsupplemented DMEM. Parasites were chilled on ice for 15 minutes, washed 3 times in prewarmed PBS, centrifuged 10 minutes at 3000 rotations per minute (rpm) between each wash; and then incubated in prewarmed nonsupplemented DMEM for 45 minutes at 37°C. After 45 minutes’ incubation, parasites were chilled on ice for 5 minutes and centrifuged for 10 minutes at 3000 rpm. Pellets were collected and respuspended in PBS (A2 and B2). Trophozoites collected from culture and respuspended in either PBS (A3 and B3) or 2% trigene (detergent; A3 and B3) were used as life and death controls, respectively. Proportion of living/dead trophozoites by flow cytometry; 5 μl of propidium iodide (PI) was added in each sample to stain DNA liberated in the milieu after cell death. Flow cytometry was performed using the BD Accuri™ C6 flow cytometer, with a blue laser (λ = 488 nm) and an optical filter 585/40. Gates P2 and P3 represent living and dead trophozoites, respectively. (A) Flow cytometry analysis for GS isolate B. Flow cytometry analysis for WB isolate. Data were analysed using BD Accuri C-flow software (version 1.0.227.4).

Figure S2: Protein expression profile for *Giardia* assemblage A and B obtained with both MS platforms. Both GS and WB pellets (P) and supernatant (S) replicates were analysed via Orbitrap and Q-Exactive MS. Supernatant protein expression profiles are similar to each other within each assemblage; so are pellet protein expression profiles (graphs). A total of 1690 and 1587 proteins were identified for assemblage B and A, respectively (Venn diagrams), via both MS techniques. For assemblage A (WB isolate), 1170 proteins were present in both datasets, and 49 and 471 proteins were identified only in the Orbitrap MS and Q-Exactive MS datasets, respectively. For assemblage B (GS isolate), 1106 proteins were present in both datasets, and 42 and 439 were identified only via Orbitrap Ms and Q-Exactive, respectively, for assemblage B.

Figure S3: *Giardia* proteins identified by Orbitrap and Q Exactive MS for assemblage A (WB isolate) and B (GS isolate). The Orbitrap MS analysis showed 639 and 426 proteins identified in both supernatant and pellet for assemblage B (GS isolate) and assemblage A (WB isolate), respectively, but also 51 (GS isolate) and 35 (WB isolate) in supernatant only and 461 (GS isolate) and 758 proteins (WB isolate) in pellet only, respectively. The Q Exactive MS showed 946 and 490 proteins identified in both supernatant and pellet for assemblage B (GS isolate) and assemblage A (WB isolate), respectively, but also 27 (GS isolate) and 24 (WB isolate) in supernatant only and 569 (GS isolate) and 1227 proteins (WB isolate) in pellet only, respectively. Proteins are ranked according to assemblage B Q-Exactive supernatant (SP) expression from most to least abundant.

Figure S4: Neighbour joining tree showing clustering of tenascins in the superfamily of High Cysteine Membrane Proteins (HCMP). Tenascin genes are highlighted in yellow. Genes were retrieved by gene name search on *Giardia*DB. Gene sequences were downloaded and aligned using ClustalW generated with the MEGA 6 software package. The maximum composite likelihood method was used, with 2000 bootstrap replicates. Bootstrap values greater than 50% are shown. ♦ indicates secreted proteins, as confirmed by our proteomic analysis. Proteins are ranked according to assemblage A Q-Exactive supernatant (SP) abundance from most to least abundant.

Figure S5: Protocol to harvest *Giardia* pellet and supernatant for proteomic analysis. (A) Preparation of *Giardia* supernatant and pellet samples for proteomic assay. Parasites were chilled for 20 minutes, transferred into 15-ml falcon tubes, and centrifuged at 3000 rpm for 10 minutes. Supernatants were discarded, and pellets were washed 3 times in warmed 1xPBS (4 ml, 2 ml, and 1 ml, respectively). Pellets were then incubated, under standard growth conditions and in filtered glass tubes, for 45 minutes at 37°C either in (1) nonsupplemented DMEM containing phenol red or (2) nonsupplemented phenol red-free DMEM. After 45 minutes’ incubation, parasites were chilled for 5 minutes, transferred in 15-ml falcon tubes, and centrifuged at 3000 rpm for 10 minutes. Pellets were stored at –20°C. Proteins present in supernatant samples were concentrated prior to performing the proteomic assay. (B) Protocol to concentrate proteins contained in *Giardia* supernatant samples prior to proteomic assay. Supernatants were transferred in Vivaspin columns with a 3000 MWCO and centrifuged at 12 000 relative centrifugal force (rcf) for 30 minutes. Proteins were washed up to 3 times with 25 mM of Ambic (depending on the presence of phenol red within DMEM) and centrifuged at 12 000 rcf for 30 minutes. Then, 50 μl of 25 mM Ambic was added, and samples were left at room temperature for 1 hour; there was a final spin at 3000 rcf for 2 minutes to recover proteins using BCA.

Figure S6: *Giardia* supernatant protein profile and protein concentration after incubation in nonsupplemented DMEM. Parasites were chilled on ice for 15 minutes, washed 3 times in prewarmed PBS, centrifuged for 10 minutes at 3000 rpm between each wash, and then incubated in prewarmed nonsupplemented DMEM for 45 minutes at 37°C. After 45 minutes’ incubation, parasites were chilled on ice for 5 minutes and centrifuged for 10 minutes at 3000 rpm. Supernatants were collected, and SDS-PAGE on 12% agarose gels, using a SYPRO staining, and BCA assay were performed—a representative gel is shown. Lane: MW: molecular weight; 2: TYI-S-33, 1:500 dilution; 3: GS supernatant; 4: WB supernatant; 5: nonsupplemented DMEM; 6: TYI-S-33, 1:100 dilution.

## Abbreviations

ABC: ATP-binding cassette; ADI: arginine deiminase; Ambic: ammonium bicarbonate; ATP: adenosine triphosphate; CaCo-2: human colonic adenocarcinoma derived epithelial cell line-2; DIDS: 4,4΄-disothiocyanatostibene-2,2΄-sulfonic acid; DMEM: Dulbecco's Modified Eagle Medium; EF-1α: elongation factor 1-alpha; EGF: epidermal growth factor; FDR: false discovery rate; *G*CATB: *Giardia* cathepsin B; GlyH101; HCMP: high-cysteine membrane protein; iBAQ: intensity-based absolute quantification; IEC: intestinal epithelial cells; IL: interleukine; Isc: short-circuit current; mRNA: messenger RNA; msrB: peptide methionine sulfoxide reductase B; OCT: ornithine carbamoyltransferase; P: pellet; PNPO: ryridoxamine 5΄-phosphate oxidase; PRIDE: PRoteomics IDEntifications; rcf: relative centrifugal force; RNA: ribonucleic acid; ROS: reductive oxygen species; rpm: rotations per minute; SP: supernatant; TEER: transepithelial electrical resistance; VSP: variant surface protein.

## Conflicts of interest

The authors declare that they have no competing interests.

## Authors’ contributions

K.T., J.M.W., J.P.W., and P.H. conceived and designed the studies. K.T. and A.D. coordinated the experiments. A.D. and S.A.N. performed the electrophysiology with J.P.W. A.D. performed the flow cytometry with D.S. A.D. prepared the proteomic samples. D.X. performed the proteomic experiments. A.D. and M.B. performed the phylogenetic analysis. All authors contributed to the analysis of the datasets obtained and preparation of figures and tables. The manuscript was drafted by A.D. and K.T. and improved and approved prior to submission by all co-authors.

## Supplementary Material

GIGA-D-17-00132_Original_Submission.pdf

GIGA-D-17-00132_Revision_1.pdf

GIGA-D-17-00132_Revision_2.pdf

Response_to_Reviewer_Comments_Original_Submission.pdf

Response_to_Reviewer_Comments_Revision_1.pdf

Reviewer_1_Report_(Original_Submission) -- Samantha Emery 09 Jul 2017 Reviewed

Reviewer_1_Report_(Revision_1) -- Samantha Emery 05 Nov 2017 Reviewed

Reviewer_2_Report_(Original_Submission) -- Feng Xue 14 Jul 2017 Reviewed

Reviewer_2_Report_(Revision_1) -- Feng Xue 05 Nov 2017 Reviewed

Reviewer_3_Report_(Original_Submission) -- Sebastien Charneau 21 Jul 2017 Reviewed

Reviewer_3_Report_(Revision_1) -- Sebastien Charneau 02 Nov 2017 Reviewed

Additional Files
