# Supplementary material for: Giardia secretome highlights secreted tenascins as a key component of pathogenesis
Source: Gigascience. 2018 Jan 29;7(3):1–13. doi: 10.1093/gigascience/giy003 (PMC5887430; doi:10.1093/gigascience/giy003)
Supplement: GIGA-D-17-00132_Original_Submission.pdf [file giy003_giga-d-17-00132_original_submission.pdf]

# GigaScience

# Giardia Secretome Highlights Secreted Tenascins as a Key Component of Pathogenesis

--Manuscript Draft--

| | |
|---|---|
| **Manuscript Number:** | GIGA-D-17-00132 |
| **Full Title:** | Giardia Secretome Highlights Secreted Tenascins as a Key Component of Pathogenesis |
| **Article Type:** | Research |
| **Funding Information:** | FP7 Science in Society (311846) — Prof Paul Raymond Hunter |
| **Abstract:** | Background: Giardia is a protozoan parasite of public health relevance that causes gastroenteritis in a wide range of hosts. Two genetically distinct lineages (assemblages A and B) are responsible for the human disease. Although it is clear that differences in virulence occur, pathogenesis and virulence of Giardia remains poorly understood.<br>Findings: The genome of Giardia is believed to contain ORFs which could encode as many as 6,000 proteins. By successfully applying quantitative proteomic analyses to the whole parasite and to the supernatants derived from parasite culture of assemblages A and B, we confirm expression of ~1,600 proteins from each assemblage, the vast majority of which being common to both lineages. To look for signature enrichment of secreted proteins, we considered the ratio of proteins in the supernatant compared with the pellet which defined a small group of enriched proteins, putatively secreted at a steady state by cultured growing trophozoites of both assemblages. This secretome contains a high proportion of proteins annotated to have N' terminus signal peptides. The most abundant secreted proteins include known virulence factors such as Cathepsin B cysteine proteases and members of a Giardia superfamily of Cysteine Rich Proteins which comprises VSPs, HCMPs and a new class of virulence factors, the Giardia Tenascins. We demonstrate that physiological function of human enteric epithelial cells is disrupted by such soluble factors even in the absence of the trophozoites.<br>Conclusions: We are able to propose a straightforward model of Giardia pathogenesis incorporating key roles for the major Giardia derived soluble mediators. |
| **Corresponding Author:** | Kevin Tyler<br>University of East Anglia<br>Norwich, Norfolk UNITED KINGDOM |
| **Corresponding Author Secondary Information:** | |
| **Corresponding Author's Institution:** | University of East Anglia |
| **Corresponding Author's Secondary Institution:** | |
| **First Author:** | Audrey Dubourg, PhD |
| **First Author Secondary Information:** | |
| **Order of Authors:** | Audrey Dubourg, PhD |
| | Dong Xia, PhD |
| | John P Winpenny, PhD |
| | Suha Al-Naimi, MBChB PhD |
| | Maha Bouzid, PhD |
| | Darren Sexton, PhD |
| | Jonathan M Wastling, PhD |
| | Paul Raymond Hunter, MBChB PhD |
| | Kevin Tyler |

Powered by Editorial Manager® and ProduXion Manager® from Aries Systems Corporation

| | |
|---|---|
| **Order of Authors Secondary Information:** | |
| **Opposed Reviewers:** | |
| **Additional Information:** | |
| **Question** | **Response** |
| Are you submitting this manuscript to a special series or article collection? | No |
| **Experimental design and statistics**<br><br>Full details of the experimental design and statistical methods used should be given in the Methods section, as detailed in our Minimum Standards Reporting Checklist. Information essential to interpreting the data presented should be made available in the figure legends.<br><br>Have you included all the information requested in your manuscript? | Yes |
| **Resources**<br><br>A description of all resources used, including antibodies, cell lines, animals and software tools, with enough information to allow them to be uniquely identified, should be included in the Methods section. Authors are strongly encouraged to cite Research Resource Identifiers (RRIDs) for antibodies, model organisms and tools, where possible.<br><br>Have you included the information requested as detailed in our Minimum Standards Reporting Checklist? | Yes |
| **Availability of data and materials**<br><br>All datasets and code on which the conclusions of the paper rely must be either included in your submission or deposited in publicly available repositories (where available and ethically appropriate), referencing such data using a unique identifier in the references and in the "Availability of Data and Materials" section of your manuscript.<br><br>Have you have met the above requirement as detailed in our Minimum Standards Reporting Checklist? | Yes |

Powered by Editorial Manager® and ProduXion Manager® from Aries Systems Corporation

Manuscript
Click here to download Manuscript Secretome GigaScience.docx

**Title:** *Giardia* **Secretome Highlights Secreted Tenascins as a Key Component of Pathogenesis**

**Running Title:** *Giardia* Secretome

**Authors: Audrey Dubourg[a], Dong Xia[b], John P. Winpenny[a], Suha Al Naimi[a,c], Maha Bouzid[a], Darren W. Sexton[a,d], Jonathan M. Wastling[b], Paul R. Hunter[a] and Kevin M. Tyler*[a]**

**Authors' affiliations:**

a. NIHR Health Protection Research Unit in Gastrointestinal Infections, Norwich Medical School, University of East Anglia, Norwich, UK.

b. Department of Infection Biology, Institute of Infection and Global Health, Faculty of Health & Life Sciences, University of Liverpool, UK.

c. School of Science, Technology and Health, University Campus Suffolk, UK

d. Liverpool Moore's University, Liverpool, UK.

Authors email:

AD: adubourg@g.ucla.edu

DX: D.Xia@Liverpool.ac.uk

JPW: John.Winpenny@uea.ac.uk

SAN: s.al-naimi@ucs.ac.uk

MB: M.Bouzid@uea.ac.uk

DWS: D.W.Sexton@ljmu.ac.uk

JMW: j.wastling@keele.ac.uk

PRH: Paul.Hunter@uea.ac.uk

KT: k.tyler@uea.ac.uk

*Correspondence: k.tyler@uea.ac.uk,

Tel +44 (0)1603-591225,

Fax: +44 (0) 1603 591750.

# Abstract

**Background:** *Giardia* is a protozoan parasite of public health relevance that causes gastroenteritis in a wide range of hosts. Two genetically distinct lineages (assemblages A and B) are responsible for the human disease. Although it is clear that differences in virulence occur, pathogenesis and virulence of *Giardia* remains poorly understood.

**Findings:** The genome of *Giardia* is believed to contain ORFs which could encode as many as 6,000 proteins. By successfully applying quantitative proteomic analyses to the whole parasite and to the supernatants derived from parasite culture of assemblages A and B, we confirm expression of ~1,600 proteins from each assemblage, the vast majority of which being common to both lineages. To look for signature enrichment of secreted proteins, we considered the ratio of proteins in the supernatant compared with the pellet which defined a small group of enriched proteins, putatively secreted at a steady state by cultured growing trophozoites of both assemblages. This secretome is enriched with proteins annotated to have N' terminus signal peptides. The most abundant secreted proteins include known virulence factors such as Cathepsin B cysteine proteases and members of a Giardia superfamily of Cysteine Rich Proteins which comprises VSPs, HCMPs and a new class of virulence factors, the Giardia Tenascins. We demonstrate that physiological function of human enteric epithelial cells is disrupted by such soluble factors even in the absence of the trophozoites.

**Conclusions**: We are able to propose a straightforward model of *Giardia* pathogenesis incorporating key roles for the major *Giardia* derived soluble mediators.


**Keywords:** *Giardia*, Secretion, Proteomics, Quantitative Proteomics, Tenascin, Cysteine Protease, Enteric Pathogen.

## Background

With some 280 million symptomatic cases, giardiasis causes more bouts of human illness than any other parasitic disease [1]. The mechanism and mediators of pathogenesis by *Giardia*, however, remain largely unknown. Thanks to human volunteer studies, the association of *Giardia* infection itself and the significance of the virulence of the infecting *Giardia* strain, is experimentally unambiguous [2]. The molecular definition associated with strain virulence is though largely unexplored. It is clear that the majority of *Giardia* infections are asymptomatic. It is also clear, that infection is primarily localized to the duodenum and that some localized damage close to the sites of colonization cause villus atrophy and apoptosis of surrounding cells. However, this localized damage cannot be the sole cause of the profound diarrhoea which is often characteristic of the disease and which appears to affect absorption over a much wider area of the digestive tract than the site of infection alone.

One of the secreted mediators of damage to the duodenum is believed to be Cathepsin B protease [3]. Cathepsin B-like proteases compose one of the superfamilies belonging to the CA clan of cysteine peptidases [4]. Compared to other cathepsins, Cathepsin B proteases possess an additional 20 amino acid insertion named the occluding loop that enables their function as an endo- or exopeptidase [5]. Although twenty-seven genes encoding Cathepsin proteases have been identified in *Giardia*, for the majority of these proteases, functions still remain elusive[6]. While some parasites may secrete Cathepsin B proteases to either evade or modulate their hosts immune responses [7], a recent study has demonstrated that *Giardia* trophozoites secrete Cathepsin B-like proteases, degrading intestinal IL-8 and thereby reducing the inflammation reaction by the host [3]. Secreted *Giardia* Cathepsin B protease *(G*CATB*)* may also facilitate trophozoites attachment to intestinal epithelia [8] and contribute to degradation of intestinal mucin [9].

Most of the proteomic studies so far reported for *Giardia* were undertaken in trophozoites undergoing encystation[10-12]. Only a few studies have focused on proteins secreted by *Giardia* and their role in the host-pathogen interaction[3,13-15]. These studies were focused on parasite interaction with intestinal cell lines. No studies have yet attempted to quantify proteins which are the product of steady state secretion by healthy, growing *Giardia* trophozoites and which we hypothesize as the primary mediators of *Giardia*sis pathology. In this study we have identified, to the limit of existing technology, the proteins expressed by populations of healthy, growing human infective *Giardia* trophozoites. We have provided quantitation of the relative abundance of retained and released trophozoite proteins from two human infective assemblages, affording calculation of the specific enrichment of released proteins and thereby the description of which are most likely to be secreted by trophozoites of each assemblage. Thereafter, we compared the profile of enrichment between the two assemblages in order to identify conserved as well as assemblage-specific secreted proteins. We provide electrophysiological analysis which confirms that trophozoite secreted molecules adversely affect the homeostasis of enteric epithelia and our analysis of the heterogeneity of encoding genes between lineages demonstrates the direct selective pressure on these virulence factors and affords their use

in discriminating clinically important strains and outbreaks. Finally, the discovery of Tenascins as a highly represented and variable group of proteins secreted by trophozoites strongly implicates this new class of virulence factors in a novel model for the mechanism of *Giardia* pathogenesis. We propose that their role follows degradation of the protective mucous afforded by the action of a secreted nuclease and *G*CATB, and following damage to cellular junctions by *G*CATB. Tenascins acting to prevent repair to those damaged junctions as a result of the EGF receptor ligation.

## Data description

Soluble and cytosolic fractions from assemblage A and B trophozoites, the aetiologic agents of human giardiasis, were extracted in order to establish which proteins are secreted in the steady state by healthy, growing trophozoite populations. We reasoned that secreted proteins would be overrepresented in the medium in which parasites were incubated compared with the trophozoites that produced them. This apparently straightforward assessment being reliant on the sensitive, specific and quantitative detection of the proteins expressed by *Giardia* trophozoites in whole cells, compared with extracts of *Giardia* proteins from the medium in which the trophozoites were incubated.

The WB (assemblage A) and GS (assemblage B) reference strains were utilized to facilitate ease of comparison between genetically divergent human infective isolates with the available reference genomes. For each experiment trophozoites were harvested from mid log growth and incubated in non-supplemented DMEM for 45 minutes at 37°C before supernatants and pellets were collected for proteomic and other analyses including validation of their viability by flow cytometry (Additional file 1: Fig S1). Proteomic analyses were based on samples from 3 distinct biological replicates. Each sample was analysed using two quantitative proteomic platforms the Orbitrap MS and the Q-Exactive MS. Thus, in total the results from 24 (2 × 2 × 2 × 3) proteomic analyses are reported.

The identification of abundant, secreted, *Giardia* virulence factors led us to consider whether the secretions from *Giardia* alone could effect changes in the behaviour of enteric epithelia - even in the absence of the trophozoites themselves. Thus, in order to determine the effect of *Giardia* trophozoite secreted factors on the intestinal epithelia, chopstick type electrodes connected to a voltmeter were used to measure the trans-epithelial electrical resistance (TEER) of polarised CaCo-2 epithelial cells grown on permeable supports. CaCo-2 cells were cultured over 6 days until confluent. TEER across the developing CaCo-2 monolayer was measured on a daily basis as shown in Figure 2A. Once confluence was established *Giardia* trophozoites were added to the apical side of the confluent epithelium and after 24 hours incubation the trophozoites were washed from the apical surface. In order to determine whether or not co-cultures of *Giardia* trophozoites or diluted *Giardia* supernatants of both human assemblages affected ion channels responsible for secretory movement across the epithelium, an Ussing chamber system was utilised with different chloride secretion inhibitor and activators.

Further details about sample collection, secretome analysis and electrophysiology can be found in the methods section.

# Analyses

### Protein expression in *Giardia* trophozoites

To obtain *Giardia* secretome with high confidence and robustness of the data, two MS techniques were used: Q-Exactive and Orbitrap MS. The Q-Exactive MS identified almost all of the proteins identified by use of the Orbitrap MS, and in total the two techniques identified 1,587 GS proteins and 1,690 WB proteins (Additional File 1: Fig S2). This represents over a quarter of the open reading frames (ORFs) predicted by the respective genomes in this single life-cycle stage under this steady state set of in vitro culture conditions and compares favourably with other recent proteomic analyses of *Giardia* [16,17]. Lists of proteins detected in only one of the two assemblages are provided (Additional file 2: Table S1 and S2). Expression from two of the 8 predicted assemblage-specific genes previously identified by comparative genomics was detected[18].

Overall, both assemblages gave comparable and consistent results by both platforms with the sensitivity of detection being greater for Q-Exactive MS; which provided a range of detection spanning 5 logs. In total, Q-Exactive MS identified 1,542 GS proteins and 1,641 WB proteins (Fig S3). Of these, 946 GS proteins were present in both pellet and supernatant, 27 in the supernatant only and 569 GS proteins in pellet only. By comparison 490 WB proteins were identified in supernatant and pellet and 24 in the supernatant only with 1,127 WB proteins in pellet only.

### *Giardia* secretome

To evaluate supernatant enrichment, proteins identified in the supernatant (SP) datasets were gathered and compared to their concentration in the pellet (P) to provide a ratio using the following formula: $\frac{SP\ Expression}{P\ Expression}$. These proteins were then ranked from highest to lowest by ratiometric value and an arbitrary cut-off invoked such that the top 50 were considered as the most likely to be secreted. Proteins identified only in SP were also included in the analysis as most likely to be secreted. All the proteins selected as “of interest” were ranked according to their SP expression from most to least abundant to obtain a quantitative enrichment profile for each isolate and this was performed for each platform. Orbitrap and Q-Exactive enrichment profiles were compared and proteins were considered as most likely to be enriched in the supernatant when identified as such by Q-Exactive MS and confirmed by Orbitrap MS. The different enrichment profiles were then also compared between assemblages.

The results showed a set of 15 orthologous proteins that were identified in both isolates by both techniques (Table 1). Eleven of these were predicted to possess an N’ terminal signal sequence. Just two of these were of unknown function and two groups dominated the annotated genes encoding the rest of these proteins, five were annotated as Tenascins and three as Cathepsin B cysteine proteases. The most abundant enriched protein was found to be pyridoxamine 5'-phosphate oxidase (PNPO), an FMN dependent enzyme capable of fixing molecular oxygen that lacks a signal peptide and which was also recently identified as a secreted *Giardia* trophozoite protein upregulated during interaction with epithelial cells[15].

An extracellular nuclease was also present, along with a high cysteine membrane protein and a protein annotated as a VSP but which was well conserved between assemblages.

We considered that where proteins were shown to be enriched in the supernatant using both platforms and in both assemblages and possessed an N' terminal signal sequence that they were truly secreted proteins. Secreted proteins involved in adapting *Giardia* to the host environment of the human gut might be expected to be engaged in Red Queen evolution and have dN/dS indicative of positive selection. While amino-acid divergence between orthologs of secreted proteins varied considerably from 67% for the HCMP to 83% for (e.g. for the extracellular nuclease), only three proteins showed evidence of positive selections, two Tenascins and one of the Cathepsins. One Cathepsin and one Tenascin in particular showed evidence of evolution under a very high degree of selective pressure (Table 1). Although, interestingly, some Cathepsins and some Tenascins with similar levels of amino-acid identity between the assemblages to those under high selective pressure showed little or no evidence of positive selection.

We considered whether lineage specific soluble mediators might also be present and identified by this method. Comparing those proteins identified by both methods as having the highest relative expression in the supernatant (Tables S3 and S4). The five most abundant conserved secreted proteins from Table 1 were also present in the top 10 secreted proteins from each assemblage amongst other VSPs, Tenascins, and Cathepsin B, and this regardless of the MS technique or the isolate. Not unexpectedly VSPs were the primary proteins enriched in supernatants that were lineage-specific. Amongst the multigene families, however, there were also differences in the Cathepsin B and Tenascins/HCMP repertoires. No other proteins with N' terminal peptides were encoded in either assemblage except for one CxC-rich protein. Interestingly none of the proteins encoded by assemblage-specific genes and identified by comparative genomics were found to be enriched in the supernatants.

### ***Giardia* soluble mediators disrupt intestinal cell functions**

Soluble and diffusible agents, able to disrupt gut function, could potentially mediate more diffuse and profound pathology for giardiasis than close range interactions between the trophozoites and the gastrointestinal epithelium alone. To determine whether *Giardia* secreted virulence factors could induce changes in the behaviour of intestinal epithelium, short-circuit current (Isc) was continuously measured across polarised CaCo-2 epithelial cells that had either been grown alone, co-cultured with *Giardia* trophozoites or exposed to diluted (1:1000) *Giardia* supernatants (Figure 2B). Further experiments demonstrated that either after 24 hour co-culture with *Giardia* (Fig 2C) or exposure to diluted *Giardia* supernatants alone (Fig 2D) both experimental conditions dramatically inhibit both the cAMP-stimulated Isc (basolateral application of Forskolin) and the calcium-activated Isc (basolateral application of UTP). In order to identify what ion channels where being affected, the CFTR chloride ion channel inhibitor, GlyH101, and the calcium-activated chloride ion channel inhibitor, DIDS, were added to the apical side of the Ussing chamber. The cAMP-stimulated Isc is predominantly due to activation of CFTR chloride channels as it

is inhibited by GlyH101 (Figure 2B-D). The calcium-activated Isc is predominantly due to activation of calcium-activated chloride channels as it is inhibited by DIDS (Figure 2B-D).

**Discussion**

Previous studies have either focused on protein secretion during *Giardia* trophozoite encystation[10-12] or protein secreted upon interaction with or attachment to host cells[3, 13-15]. Here, we have demonstrated that *Giardia* trophozoites secrete proteins under steady state of growth in vitro. The most abundant proteins, in both human isolates, mainly belong to four families of protein: *Giardia* Cathepsin B family, High Cysteine Membrane proteins (HCMPs), Variant surface proteins (VSPs) and Tenascins.

The Cathepsin B family of *Giardia (GCATB)* contains secreted and non-secreted trophozoite expressed proteins; the orthologues of which are predominantly common to GS and WB assemblages (Fig 1). Expression of sixteen *G*CATBs was proteomically confirmed, of which eleven were shown by our proteomic analysis to be secreted. These eleven fell into six orthologous groups and for three of these groups all group members were shown to be secreted. Secreted *G*CTAB GL50803_15564 (WB) and its ortholog GL50581_2036 (GS) show a dN/dS values of >26 indicative of strong positive selective pressure. Interestingly when GS was resequenced GL50803_15564 was found to comprise three recently diverged orthologs (GSB_153537, GSB_155477, GSB_150353) and it may be that the positive selection pressure observed has been generated as a result of recent gene duplications in the assemblage B strain.

HCMPs are an enigmatic group of proteins with few associated functional studies. They may protect trophozoites against proteolysis [19,20] and oxidative damage [21]. In *Giardia*, it appears that one lineage of HCMPs has given rise to the VSPs, whilst another has given rise to a group with high homology to mammalian Tenascins. Tenascin, VSPs and HCMPs are then related multi-gene families which together form the largest group of proteins enriched in the *Giardia* supernatants. Interestingly, when aligned and analysed phylogenetically the secreted Tenascins segregate into a monophyletic group (Figure S4). Both WB and GS orthologs of five tenascin gene products were secreted and in WB two other secreted Tenascins were also detected that were not detected for the GS strain (Figure 1B).

VSPs are well-characterised surface glycoproteins with transmembrane domains, which are expressed one at a time by *Giardia* trophozoites through an RNAi regulated mechanism. They are quintessential virulence factors, responsible for antigenic variation. VSPs are hypervariable by nature and thus it is to be expected that they do not form orthologous pairs. This was the case for most we observed, intriguingly though, a few proteins annotated as VSPs were conserved between isolates suggesting that they are not actually VSPs and would not be subject to “one at a time” control expression - but are actually misannotated HCMPs which may have a conserved function in both GS and WB isolates.

Tenascins are characterised by the presence of epidermal growth factor (EGF) repeats and are able to act as ligands for EGF receptors. Mammalian Tenascins are extracellular matrix proteins that modulate cell adhesion and migration [22]. They appear to have evolved from a

group of proteins specific to vertebrates, presumably co-evolving with the EGF receptor and so the presence of homologous proteins in *Giardia* evolving independently from HCMPs is a clear example of the kind of convergent evolution best described as molecular mimicry.

Most published studies concerning host cell-*Giardia* interactions have focused on the effects on the host intestinal epithelia upon attachment of the trophozoites to the cells. In this study, we have shown that diluted supernatant obtained from the steady growth of *Giardia* trophozoites in vitro has an effect on the intestinal cell function. The effect observed on chloride secretion by *Giardia* supernatants indicates that *Giardia* secretes a soluble factor which is likely affecting secretion across the intestinal epithelial cells. Physiologically, cultured intestinal cells show sensitivity to *Giardia* proteins released by the parasite even at high dilution. Fig2D demonstrates that intestinal epithelial cells when acutely exposed to such *Giardia* proteins lose the ability to stimulate CFTR and calcium-activated chloride channels. The clear implication being that virulence determinants released from *Giardia* trophozoites interact with epithelial cell receptors and ion channels.

In this analysis, we have identified the proteins which are secreted by human infective *Giardia* trophozoites. Just two groups form the majority of these proteins: *G*CATBs and the HCMP superfamily encoding known virulence factors in addition to an abundant extracellular nuclease and an oxygen fixing enzyme. The elucidation of this group of proteins dramatically increases our understanding of the pathogenic mechanisms underlying *Giardia*sis at a molecular level. The genes encoding GCATBs and HCMP superfamily proteins are among the most heterogeneous of all genes between assemblages. Their probable role in interaction with the host and luminal environment is supported by the very high dN/dS values of some family members. Correlation of variation within assemblages at these loci with strain virulence is the essential next step for their use in diagnosis of virulent strains, risk assessment and disease prognosis.

Our results indicate that *Giardia* secretions are sufficient to disable normal function in enteric epithelial making cells less able to extract fluids from the lumen. In particular, they implicate PNPO, an extracellular nuclease, *G*CATBs and Tenascins. The fact that both extracellular nuclease and *G*CATBs can be involved in the degradation of the intestinal mucus layer and that both *G*CATBs and Tenascins can be associated with intestinal intracellular junction disruption suggests collaboration between these proteins. Therefore, we propose a pathogenic mechanism (Fig 3) whereby PNPO produces a reducing environment optimal for trophozoite growth, the extracellular nuclease degrades the outer layer of the intestinal mucus improving access for *G*CATBs for further degrading the protective mucous barrier and then disrupting the intestinal intracellular junctions. Lastly, Tenascins are involved in maintaining intestinal cell separation by attaching to the EGF receptors present at the surface of intestinal cells and exacerbate epithelial damage by increasing the level apoptosis amongst these more detached cells. Once the intestinal barrier is breached by these mechanisms the sites of damage become prone to secondary infection by other opportunist microbes resident in the intestinal lumen and sensitive to irritation by allergens in foodstuffs leading to further inflammation and to the characteristic

symptoms of the disease. Further investigations are necessary to verify this potential mechanism of pathogenesis of *Giardia*sis.

METHODS

**Proteomic Analysis.** *Giardia* trophozoites from the genome reference strains WB (assemblage A) and GS (assemblage B), were cultured in TYI-S-33 under standard conditions[23] and harvested during the midlog phase of their growth curves. Trophozoites were washed 3 x in PBS and then incubated in non-supplemented DMEM for 45 minutes at 37°C. After incubation, an aliquot was analysed by flow cytometry to evaluate the viability of the *Giardia* samples. Trophozoites and supernatant were separated by centrifugation and both trophozoite pellet and supernatant were harvested. Supernatants were analysed by SDS page electrophoresis and were tested on cultured epithelial cells (Caco-2) to ensure the presence of proteins and biological activity (see below). Supernatants and pellets were sent to the Institute of Infection and Global Health at the University of Liverpool for mass spectrometry analysis (Figure S3). Proteins contained in supernatant were concentrated in Vivaspin columns with 25 mM Ambic. Peptide mixtures were analysed by on-line nanoflow liquid chromatography using the nanoACQUITY-nLC system (Waters MS technologies) coupled to an LTQ-Orbitrap Velos (Thermo Fisher Scientific) mass spectrometer or an Ultimate 3000 nano system coupled to a Q-Exactive mass spectrometer (Thermo Fisher Scientific). Once samples had been run through both platforms, and peptides matched to their proteins on *Giardia*DB.org at 1% FDR with a minimum of two unique peptides, datasets were then further analysed bioinformatically. The mass spectrometry proteomics data have been deposited to the ProteomeXchange Consortium via the PRIDE partner repository[24] with the dataset identifier PXD004398 and 10.6019/PXD004398.

**Electrophysiology**. CaCo-2 cells (passages 20-25) were grown in Dulbecco's Modified Eagle medium supplemented with nonessential amino acids, penicillin (12 IU/ml), streptomycin (12µg/ml), gentamycin (47 µg/ml) and 20% (vol/vol) heat inactivated fetal calf serum (all from AMIMED, Bioconcept). The cells were seeded at a density of 6 x $10^6$ cells/$cm^2$ in 6-well Transwell filters (0.4 µm pore size) and cultured for 7-15 days until confluent.

Monolayers of CaCo-2 cells were mounted into a Physiological Instruments EM-CSYS-2 Ussing chamber set-up 7-15 days after establishment of a confluent monolayer and the short circuit current ($I_{SC}$) across the monolayer was continuously measured.

Both sides of the epithelium were bathed in 5mls of Krebs Henseleit solution that was continuously circulated through the half chambers, maintained at 37°C and continuously bubbled with 95% $O_2$ / 5% $CO_2$. The composition of the Krebs Henseleit bath solution used was similar to that used by Cuthbert [24] and had the following composition (in mM): NaCl 118, KCl 4.7, $CaCl_2$ 2.5, $MgCl_2$ 1.2, $NaHCO_3$ 25, $KH_2PO_4$ 1.2 and glucose 11.1 (pH 7.4). The permeable supports were left for 30 mins to equilibrate before experiments were started.

$I_{SC}$ was continuously monitored across the monolayers by a Physiological Instruments Multichannel Voltage/Current Clamp (VCC MC6) through 3M KCl/agar, Ag/$AgCl_2$ cartridge electrodes (Physiologic Instruments), and the raw data for $I_{sc}$, transepithelial resistance and transepithelial voltage were recorded using Acquire and Analyse version 1.3 software (Physiological Instruments). Data was exported to Microsoft Excel initially and then into

GraphPad Prism version 5.0 for Windows package for data representation and statistical analysis. All filters were treated with 10µM amiloride apically to eliminate electrogenic sodium absorption through epithelial sodium channels (ENaC).

**Chemicals and Inhibitors.** Forskolin (10µM), UTP (100µM), Amiloride (10mM), and DIDS (100µM) were obtained from Sigma Aldrich, and GlyH-101 (50 µM) and was obtained from Merck Chemicals. Stock solutions of Amiloride (10mM), GlyH-101 (50mM) were created by dissolving in DMSO. Final concentrations of drugs are as indicated in the text or figures and where produced by adding the appropriate volume of stock concentration to 5mls of either the basolateral or apical Ussing chamber.

**Phylogeny.** To look for sequence similarities between proteins of interest from a same protein family, the coding sequences of these proteins were retrieved from *Giardia*DB, aligned and compared using ClustalW.

Phylogenetic trees were built for these proteins, via Maximum likelihood approach using MEGA software (v. 6.06).

**Availability of Supporting Data**

All proteomic datasets are held by and can be accessed for free at the European Bioinformatics PRoteomics IDEntifications (PRIDE) database https://www.ebi.ac.uk/pride/archive/. Free Integrated functionality with other Giardia large datasets hosted at eupathDB http://eupathdb.org/eupathdb/ is scheduled for the next release.

**Abbreviations:** CaCo-2 (Human colonic adenocarcinoma derived epithelial cell line-2); DMEM (Dulbecco's Modified Eagle Medium); EGF (Epidermal growth factor); *G*CATB (*Giardia* Cathepsin B); HCMP (High cysteine membrane protein); IL (Interleukine); Isc (Short-circuit current); ORF (Open reading frame); TEER (*Trans*-epithelial electrical resistance)

**Conflicts of Interest**

The authors declare that they have no competing interests

**Authors' Contributions**

K.T., J.M.W., J.P.W and P.H. conceived and designed the studies. K. T. and A. D. co-ordinated the experiments. A.D. and S.A.N. performed the electrophysiology with J.P.W. A.D. performed the Flow Cytometry with D.S. A.D. prepared the proteomic samples. D.X. performed the proteomic experiments. A.D. and M.B. performed the phylogenetic analysis. All authors contributed to the analysis of the data sets obtained and preparation of Figures and Tables. The manuscript was drafted by A.D. and K.T. and improved and approved prior to submission by all co-authors.

**Acknowledgements**

The research leading to these results was primarily funded from the European Union Seventh Framework Programme ([FP7/2007-2013] [FP7/2007-2011]) under Grant agreement no:

311846. PRH is supported by the National Institute for Health Research Health Protection Research Unit (NIHR HPRU) in Gastrointestinal Infections at the University of Liverpool in partnership with Public Health England (PHE), and in collaboration with University of East Anglia, University of Oxford and the Institute of Food Research. Professor Hunter is based at University of East Anglia. The views expressed are those of the author(s) and not necessarily those of the NHS, the NIHR, the Department of Health or Public Health England.

## REFERENCES


1. Esch, K.J. & Petersen, C.A. Transmission and epidemiology of zoonotic protozoal diseases of companion animals. *Clinical microbiology reviews* **26**, 58-85 (2013).
2. Nash, T.E., Herrington, D.A., Losonsky, G.A. & Levine, M.M. Experimental human infections with *Giardia* lamblia. *The Journal of infectious diseases* **156**, 974-984 (1987).
3. Cotton, J.A., *et al. Giardia* duodenalis cathepsin B proteases degrade intestinal epithelial interleukin-8 and attenuate interleukin-8-induced neutrophil chemotaxis. *Infect Immun* (2014).
4. Turk, V., *et al.* Cysteine cathepsins: from structure, function and regulation to new frontiers. *Biochim Biophys Acta* **1824**, 68-88 (2012).
5. Musil, D., *et al.* The refined 2.15 A X-ray crystal structure of human liver cathepsin B: the structural basis for its specificity. *EMBO J* **10**, 2321-2330 (1991).
6. DuBois, K.N., *et al.* Identification of the major cysteine protease of *Giardia* and its role in encystation. *J Biol Chem* **283**, 18024-18031 (2008).
7. Sajid, M. & McKerrow, J.H. Cysteine proteases of parasitic organisms. *Molecular and biochemical parasitology* **120**, 1-21 (2002).
8. Rodriguez-Fuentes, G.B., *et al. Giardia* duodenalis: analysis of secreted proteases upon trophozoite-epitheial cell interaction in vitro. *Mem Inst Oswaldo Cruz* **101**, 693-696 (2006).
9. Paget, T.A. & James, S.L. The mucolytic activity of polyamines and mucosal invasion. *Biochem Soc Trans* **22**, 394S (1994).
10. Wampfler, P.B., Tosevski, V., Nanni, P., Spycher, C. & Hehl, A.B. Proteomics of Secretory and Endocytic Organelles in *Giardia* lamblia. *PLoS One* **9**, e94089 (2014).
11. Faso, C., Bischof, S. & Hehl, A.B. The proteome landscape of *Giardia* lamblia encystation. *PLoS One* **8**, e83207 (2013).
12. Lingdan, L., *et al.* Differential dissolved protein expression throughout the life cycle of *Giardia* lamblia. *Exp Parasitol* **132**, 465-469 (2012).
13. Ringqvist, E., *et al.* Release of metabolic enzymes by *Giardia* in response to interaction with intestinal epithelial cells. *Molecular and biochemical parasitology* **159**, 85-91 (2008).
14. Roxstrom-Lindquist, K., Palm, D., Reiner, D., Ringqvist, E. & Svard, S.G. *Giardia* immunity--an update. *Trends Parasitol* **22**, 26-31 (2006).
15. Emery, S.J., *et al.* Induction of virulence factors in *Giardia* duodenalis independent of host attachment. *Scientific reports* **6**, 20765 (2016).
16. Emery, S.J., Lacey, E. & Haynes, P.A. Data from a proteomic baseline study of Assemblage A in *Giardia* duodenalis. *Data in brief* **5**, 23-27 (2015).

17. Emery, S.J., Lacey, E. & Haynes, P.A. Quantitative proteomic analysis of *Giardia* duodenalis assemblage A: A baseline for host, assemblage, and isolate variation. *Proteomics* **15**, 2281-2285 (2015).
18. Jerlstrom-Hultqvist, J., Ankarklev, J. & Svard, S.G. Is human *Giardia*sis caused by two different *Giardia* species? *Gut Microbes* **1**, 379-382 (2010).
19. Davids, B.J*., et al.* A new family of *Giardia*l cysteine-rich non-VSP protein genes and a novel cyst protein. *PLoS One* **1**, e44 (2006).
20. Nash, T.E. Surface antigenic variation in *Giardia* lamblia. *Mol Microbiol* **45**, 585-590 (2002).
21. Requejo, R., Hurd, T.R., Costa, N.J. & Murphy, M.P. Cysteine residues exposed on protein surfaces are the dominant intramitochondrial thiol and may protect against oxidative damage. *The FEBS journal* **277**, 1465-1480 (2010).
22. Chiquet-Ehrismann, R. & Chiquet, M. Tenascins: regulation and putative functions during pathological stress. *The Journal of pathology* **200**, 488-499 (2003).
23. Keister, D.B. Axenic culture of *Giardia* lamblia in TYI-S-33 medium supplemented with bile. *Trans R Soc Trop Med Hyg* **77**, 487-488 (1983).
24. Vizcaíno, J.A., *et al.* 2016 update of the PRIDE database and related tools. *Nucleic Acids Res* 44(D1): D447-D456 (2016).
25. Cuthbert, A.W. Assessment of CFTR chloride channel openers in intact normal and cystic fibrosis murine epithelia. *Br J Pharmacol* **132**, 659-668 (2001).

# Figures Legends:

**Table 1: The secretome of human infective *Giardia* trophozoites of assemblage A and B have a conserved repertoire of abundant secreted factors identified by both Orbitrap MS and Q-Exactive MS.** 15 proteins were identified as most likely to be secreted by both GS and WB isolates. 12 are annotated proteins and 3 are hypothetical proteins. Proteins are ranked according to GS Q-Exactive Supernatant (SP) expression from most to least abundant. Of the 12 annotated proteins, 5 are tenascins and 3 are related high cysteine membrane proteins or VSP and three are Cathepsin Bs. The other annotated abundant secreted protein is an extracellular nuclease. Protein ranking represents the proteins rank within this table, from most to least abundant. Detailed breakdown of the secretome for each assemblage by each method are provided in Supplemental tables 1-4.

**Figure 1: Neighbour joining tree showing clustering of A) Cathepsin B and B) Tenascin gene families.** Genes were retrieved by gene name search on *Giardia*DB. Gene sequences were downloaded and aligned using ClustalW generated with MEGA 6 software package. Maximum composite likelihood method was used, with 2000 bootstrap replicates. Bootstrap values greater than 50% are shown above the branches. ◆ proteins confirmed to be secreted using our proteomic analysis.

**Figure 2: The effect of *Giardia* co-culture and *Giardia* supernatants on the electrophysiological properties of CaCo-2 monolayers. A)** Transepithelial electrical resistance (TEER) in CaCo-2 monolayers following seeding on permeable supports. Data shows increase in TEER as monolayer develops. Confluence occurred around Day 6. *Giardia* were added on Day 6 after confluent monolayer formed and co-cultured with the Caco-2 monolayer for 24 hours. TEER was measured after 24 hours and compared to TEER in monolayers that had not been exposed to *Giardia* (n=6). **B)** A representative short circuit current (Isc) against time recording from a single monolayer of CaCo-2 cells in an Ussing chamber. The trace shows the activation of CFTR chloride channels (basolateral application of 10 µM Forskolin) and calcium-activated chloride channels (basolateral application of 100 µM UTP). Specificity of activation is confirmed by inhibition of Isc by the specific CFTR channel blocker, GlyH101; and specific calcium-activated chloride channel blocker, DIDS. The effect on Isc of incubation of CaCo-2 monolayers with *Giardia* or with *Giardia* supernatants is also shown. **C)** Effect of 24 hour co-incubation of CaCo-2 monolayers with different strains of *Giardia* (WB, GS and patient samples) on forskolin-stimulated and UTP-stimulated Isc (n=3). **D)** Effect of acute addition of supernatants from different strains of *Giardia* (WB, GS and patient samples) on forskolin-stimulated and UTP-stimulated Isc (n=3) from Caco-2 monolayers. The results were analysed by student's t-test and expressed as mean values ± standard error mean (SEM). Significant difference expressed as *P<0.05, **P<0.01 compared to control.

**Figure 3: Proposed novel mechanism of pathogenicity for *Giardia* involving PNPO, extracellular nuclease, *G*CATB, Tenascin.** PNPO (◆) renders the intestinal environment more favourable to trophozoite's growth. Once a new *Giardia* colony is established, trophozoites release extracellular nuclease (▬), GCATB (●) and Tenascin (▲). Extracellular nuclease may contribute to reducing the viscosity of the intestinal outer mucus layer, while *G*CATB may degrade mucins and disrupt intracellular junction. Finally, Tenascins may maintain intestinal cells apart by attaching to the EGF receptors present at the surface of intestinal cells which could over time lead to the apoptosis of these isolated intestinal cells.

**Extended data legends:**

**Table S1:** List of the *Giardia* assemblage A (WB strain) lineage-specific proteins identified via Orbitrap and Q-Exactive MS.
Protein sequences were compared to their coding sequence and matched to their orthologs in assemblage B (GS strain) using *Giardia* database: *Giardia*DB.org. Annotated proteins are highlighted in red and hypothetical proteins in blue. Proteins were ranked according to Q-Exactive S Supernatant (S) abundance, from most to least abundant.

**TableS2:** List of the *Giardia* assemblage B (GS strain) lineage-specific proteins identified via Orbitrap and Q-Exactive MS.
Protein sequences were compared to their coding sequence and matched to their orthologs in assemblage A (WB strain) using *Giardia*DB.org. Annotated proteins are highlighted in red and hypothetical proteins in blue. Proteins were ranked according to Q-Exactive Supernatant (S) abundance, from most to least abundant.

**Table S3:** List of the 31 proteins identified via Orbitrap and Q-Exactive MS as proteins most likely secreted by *Giardia* GS strain trophozoites.
24 proteins are annotated (shown in red) and 7 are hypothetical proteins (shown in blue). 8 proteins are lineage-specific. The 15 proteins identified as conserved between the two isolates are highlighted in grey. Only proteins identified via both techniques were considered as secreted and shown in this table. Proteins are ranked according to Q-Exactive SP expression from most to least abundant.

**Table S4:** List of the 44 proteins identified via Orbitrap and Q-Exactive MS as proteins most likely secreted by *Giardia* WB strain trophozoites.
38 proteins are annotated (shown in red) and 6 are hypothetical proteins (shown in blue). 10 proteins are lineage-specific. The 15 proteins identified as conserved between the two isolates are highlighted in grey. Only proteins identified via both techniques were considered as secreted and shown in this table. Proteins are ranked according to Q-Exactive Supernatant (S) expression from most to least abundant.

**Figure S1**: ***Giardia* trophozoites are viable after incubation in non-supplemented DMEM.** Parasites were chilled on ice for 15 min, washed 3 times in pre-warmed PBS, centrifuged 10 min at 3,000 rpm between each wash; and then incubated in pre-warmed non-supplemented DMEM for 45 min. at 37°C. After 45 min incubation, parasites were chilled on ice for 5 min and centrifuged 10 min at 3,000 rpm. Pellets were collected and respuspended in PBS (A2 and B2). Trophozoites collected from culture and respuspended in either PBS (A3 and B3) or 2% trigene (detergent) (A3 and B3) were used as live and death control respectively.
Proportion of living/dead trophozoites by flow cytometry. 5 µl of propidium iodide (PI) were added in each sample to stain DNA liberated in the milieu after cell death. Flow cytometry was performed using the BD Accuri™ C6 flow cytometer, with a blue laser (λ= 488 nm) and an optical filter 585/40. Gate P2 and P3 represent alive and dead trophozoites respectively. **A.** Flow cytometry analysis for GS isolate **B.** Flow cytometry analysis for WB isolate.
Data were analysed using the BD Accuri C-flow software (version 1.0.227.4).

**Figure S2: Protein expression profile for *Giardia* assemblage A and B obtained with both MS platforms.** Both GS and WB pellet (P) and supernatant (S) replicates were analysed via Orbitrap and Q-Exactive MS. Supernatant protein expression profile are similar to each other within each assemblage, so are pellet protein expression profiles (*Graph charts*). A total of 1,690 and 1,587

proteins were identified for assemblage B and A respectively (*Venn diagrams*) via both MS techniques. For assemblage A (WB isolate), 1,170 proteins were present in both dataset, 49 and 471 proteins were identified only in Orbitrap MS dataset and Q-Exactive MS datasets respectively. For assemblage B (GS isolate) 1,106 proteins were present in both datasets, 42 and 439 were identified only via Orbitrap Ms and Q-Exactive respectively for assemblage B.

**Figure S3: *Giardia* proteins identified by Orbitrap and Q Exactive MS for assemblage A (WB isolate) and B (GS isolate).** The Orbitrap MS analysis showed 639 and 426 proteins identified in both supernatant and pellet for assemblage B (GS isolate) and assemblage A (WB isolate) respectively, but also 51 (GS isolate) and 35 (WB isolate) in supernatant only and 461 (GS isolate) and 758 proteins (WB isolate) in pellet only respectively. The Q Exactive MS showed 946 and 490 proteins identified in both supernatant and pellet for assemblage B (GS isolate) and assemblage A (WB isolate) respectively, but also 27 (GS isolate) and 24 (WB isolate) in supernatant only and 569 (GS isolate) and 1,227 proteins (WB isolate) in pellet only respectively.
Proteins are ranked according to assemblage B Q-Exactive Supernatant (SP) expression from most to least abundant

**Figure S4: Neighbor joining tree showing clustering of Tenascins in the superfamily of High Cysteine Membrane Proteins (HCMP).** Tenascin genes are highlighted in yellow. Genes were retrieved by gene name search on *Giardia*DB. Gene sequences were downloaded and aligned using ClustalW generated with MEGA 6 software package. Maximum composite likelihood method was used, with 2000 bootstrap replicates. Bootstrap values greater than 50% are shown. ◆ indicates secreted proteins as confirmed by our proteomic analysis.
Proteins are ranked according to assemblage A Q-Exactive Supernatant (SP) expression from most to least abundant

**Table 1:**

| Protein Description | GI number Assemblage A | GI number Assemblage B | A:B Identity | dN/dS | Signal peptide[b] | P protein iBAQ | SP protein iBAQ | SP/P ratio | Abundance Ranking |
|---|---|---|---|---|---|---|---|---|---|
| PNPO | GL50803_5810 | GL50581_4133 | 99.2 | 0.038 | NP | 5.71E+07 | 1.18E+08 | 2.063091141 | 1 |
| Tenascin | GL50803_55162 | GL50581_1982 | 76.7 | **1.597**[a] | 0.99 | 2.97E+07 | 4.77E+07 | 1.607023941 | 2 |
| Tenascin | GL50803_10330 | GL50581_4057 | 73.5 | 0.347 | 0.99 | 4.66E+06 | 2.02E+07 | 4.342293266 | 3 |
| Cathepsin B | GL50803_16468 | GL50581_438 | 83.6 | 0.1072 | 0.78 | 9.63E+06 | 1.79E+07 | 1.861308975 | 4 |
| Tenascin | GL50803_8687 | GL50581_4316 | 77.6 | **44.176**[a] | 0.98 | 6.22E+06 | 1.10E+07 | 1.770525826 | 5 |
| Uncharacterized | GL50803_5258 | GL50581_2767 | 91.2 | 0.029 | NP[c] | 3.93E+06 | 1.09E+07 | 2.780917516 | 6 |
| Extracellular nuclease | GL50803_8742 | GL50581_3607 | 83.1 | 0.234 | 1 | 1.03E+06 | 4.57E+06 | 4.436192622 | 7 |
| Tenascin | GL50803_16477 | GL50581_3575 | 79.8 | 0.1256 | 0.99 | 8.35E+05 | 4.03E+06 | 4.830955632 | 8 |
| Cathepsin B | GL50803_15564 | GL50581_2036 | 79.1 | **26.5782**[a] | 1 | 1.10E+06 | 3.95E+06 | 3.589608246 | 9 |
| CKS1 | GL50803_2661 | GL50581_3484 | 100 | 0.001 | NP[c] | 1.14E+06 | 3.20E+06 | 2.803061521 | 10 |
| Tenascin | GL50803_113038 | GL50581_4180 | 79 | 0.0949 | 1 | 1.20E+06 | 3.14E+06 | 2.620931138 | 11 |
| HCMP | GL50803_7215 | GL50581_727 | 67 | 0.1821 | 0.99 | ND[d] | 2.54E+06 | ∞ | 12 |
| Uncharacterized | GL50803_16522 | GL50581_352 | 76 | 0.1591 | NP[c] | 1.15E+06 | 2.21E+06 | 1.928833128 | 13 |
| HCMP | GL50803_12063 | GL50581_2622 | 83 | 0.246 | 1 | 3.62E+05 | 1.94E+06 | 5.354665277 | 14 |
| Cathepsin B | GL50803_17516 | GL50581_2318 | 72.8 | 0.2056 | 1 | ND[d] | 7.81E+05 | ∞ | 15 |

[a] dN/dS in bold indicate proteins show evidence of positive selective pressure during divergence from a common ancestor.

[b] Probability of an N-terminal signal peptide using SignalP.

[c]NP – Not predicted.

[d]ND – Not Detected.

Figure 1 Click here to download Figure Figure 1.pdf

**Figure 1**

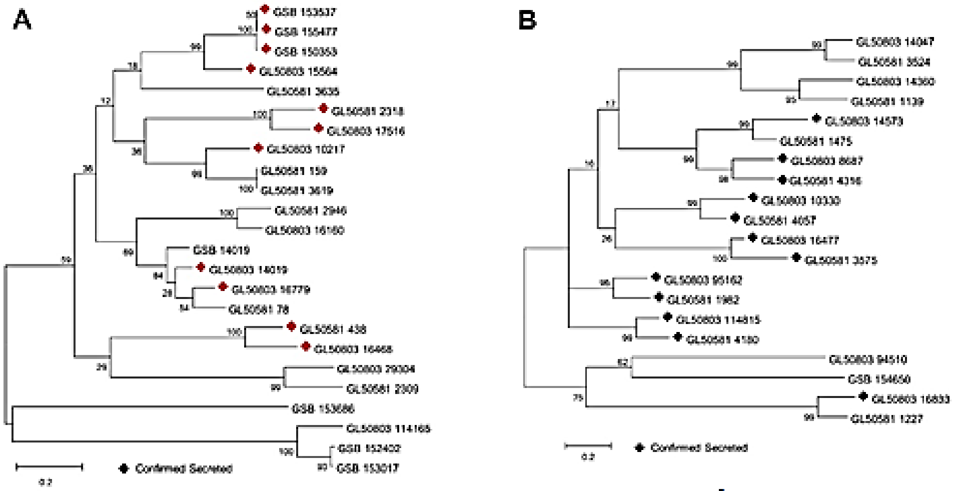


*Giardia* Cathepsin B

*Giardia* Tenascin

 Click here to download Figure Figure 2.pdf

**Figure 2**

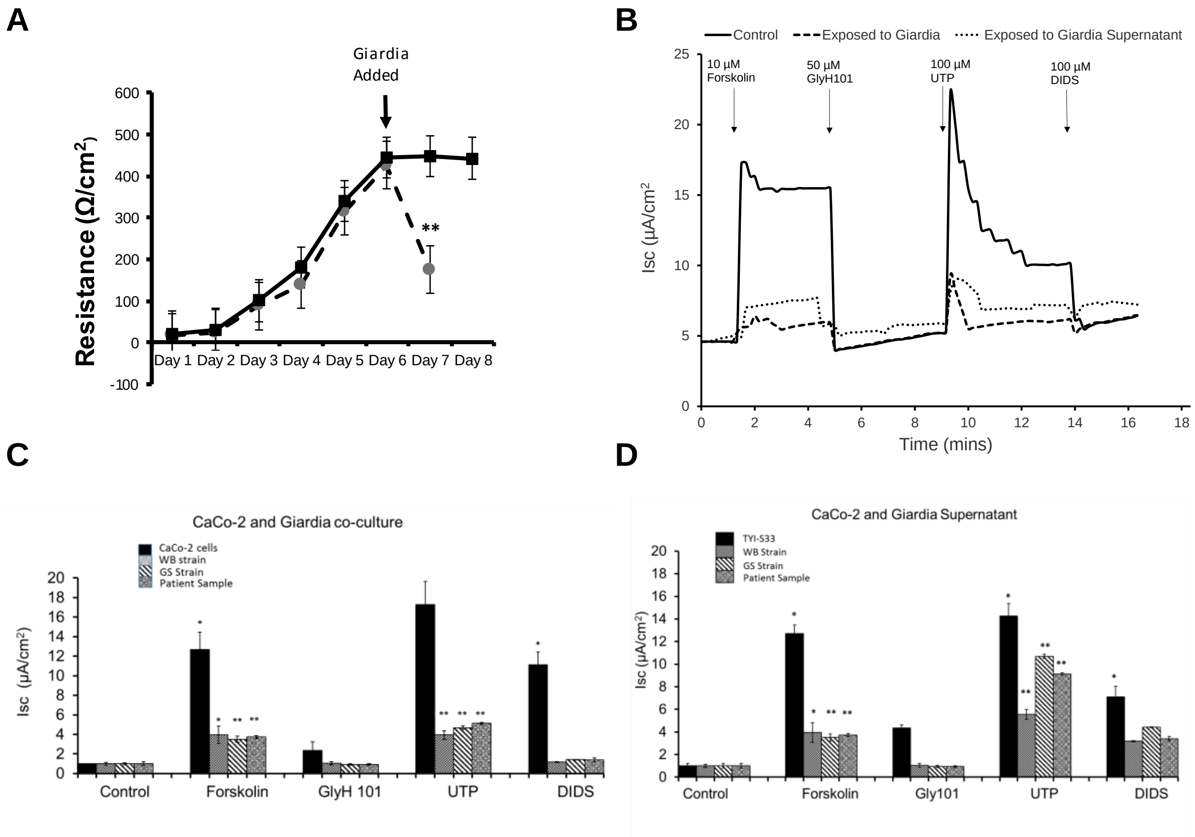

Figure 3

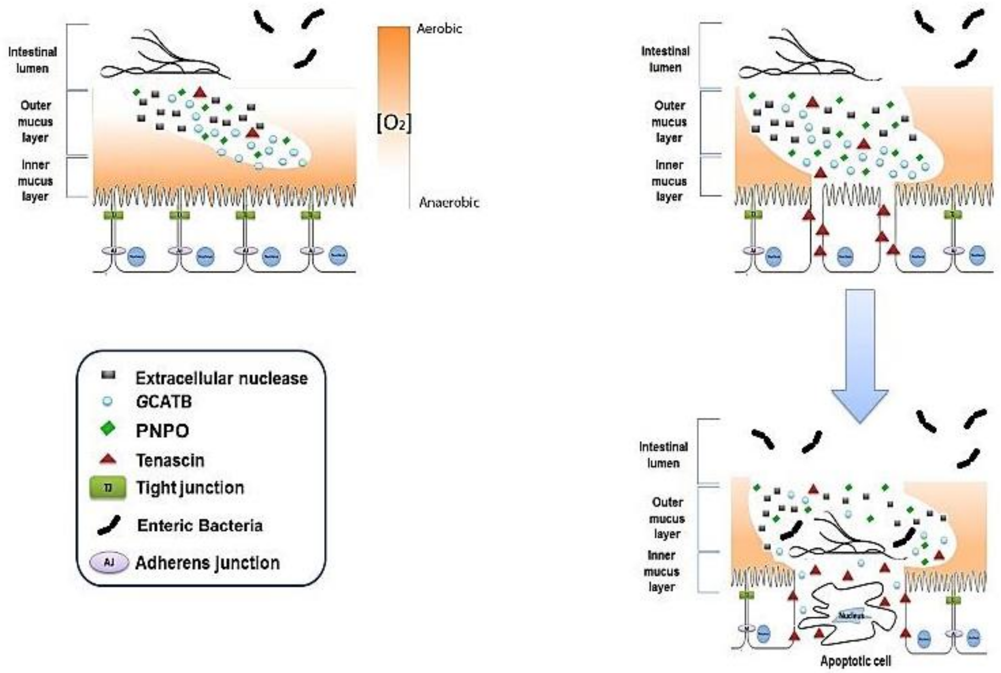


*Mechanism of pathogenesis*

Figure S1

Click here to access/download
**Supplementary Material**
Additional Figure S1.pdf

Figure S2

Click here to access/download
**Supplementary Material**
Additional File Figure S2.pdf

Figure S3

Click here to access/download
**Supplementary Material**
Additional File Figure S3.pdf

Figure S4

Click here to access/download
**Supplementary Material**
Additional File Figure S4.pdf

Table S1

Click here to access/download
**Supplementary Material**
Additional file Table S1.pdf

Table S3

Click here to access/download
**Supplementary Material**
Additional file Table S3.pdf

Table S4

Click here to access/download
**Supplementary Material**
Additional file Table S4.pdf

Table S2

Click here to access/download
**Supplementary Material**
Additional Table S2.pdf

Cover letter
Click here to download Personal Cover SecretomeCoverLetter.docx

Faculty of Medicine and Health Sciences

Dr K. M. Tyler
University of East Anglia
Norwich Medical School
Norwich, Norfolk NR4 7TJ
http://www.uea.ac.uk/~wm077/
01144(0)1603-591225 (Direct Line)
k.tyler@uea.ac.uk

UEA University of East Anglia | NORWICH MEDICAL SCHOOL

Dear Dr Goodman,

It is my pleasure to submit our latest discoveries for your consideration at Gigascience.

Although Giardia was the first microscopic pathogen to be visualized on van Leeuwenhoek's microscope (1681), it remains the cause of more episodes of human illness than any parasite. 335 years on we still don't know what mediates giardiasis pathology. Here we provide a data set which transforms our understanding of how secreted Giardia virulence factors can elicit diahorreal pathology. The study is enabled by the use of the latest proteomic technology which affords quantitation as well as exquisite sensitivity for identification of pathogen proteins. By wielding this technology we were able to describe the building blocks of Giardia itself as large data sets and in unprecedented detail and to provide this through public databases as a resource which we believe will be welcomed by the wider community for providing proof of expression for a large number of hitherto hypothetical proteins.

By further applying these proteomic technologies to culture supernatants from multiple parasite lineages, we were able to determine which proteins were being selectively transported out of the parasites - providing a quantitative secretome for the first time. The results are remarkable and surprising, indicating that the pathogenic form of the parasite secretes few proteins which fall predominantly into just a two families of virulence factors. One well known (Cathepsin Bs) damage the mucosa directly, but the other (Tenescins) are mimics of a mammalian class of extracellular matrix protein and almost entirely uncharacterized. This reflects what is likely to be a novel mechanism by which enteric pathogens can use soluble virulence factors to perpetuate damage to the gastric mucosa. The profile of secreted proteins also helps to cement our understanding of other aspects of giardiasis pathology and we demonstrate using electrophysiological assays that soluble components can exert effects on gut mucosa similar to that of whole living parasites, resolving what has long been observed as a contradiction - that the profound and generalised dysfunction of the gut causing severe diahorrea could be mediated by localized infections in the duodenum. Overall, the manuscript provides long sought mechanistic insight and new intervention targets for one of the world's most important diseases.

Suggestions -

Editorial board:
K. Nelson

Reviewers:
staffan.svard@icm.uu.se
fgillin@ucsd.edu
Bjorn.Andersson@ki.se

A reviewer account for the proteomic data prior to publication can be accessed at https://www.ebi.ac.uk/pride/archive/ with the following login details:
Username: reviewer90411@ebi.ac.uk
Password: 24ksbhjN

Yours sincerely,

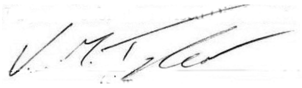

P.S. EupathDB (http://eupathdb.org/eupathdb/ ) are also in receipt of the dataset and will provide for the dataset integrated with existing genomic and transcriptomic data in the October release if the article has been accepted by that point.
