# Supplementary material for: Giardia secretome highlights secreted tenascins as a key component of pathogenesis
Source: Gigascience. 2018 Jan 29;7(3):1–13. doi: 10.1093/gigascience/giy003 (PMC5887430; doi:10.1093/gigascience/giy003)
Supplement: GIGA-D-17-00132_Revision_1.pdf [file giy003_giga-d-17-00132_revision_1.pdf]

GigaScience
Giardia Secretome Highlights Secreted Tenascins as a Key Component of Pathogenesis
--Manuscript Draft--

**Manuscript Number:**	GIGA-D-17-00132R1
**Full Title:**	Giardia Secretome Highlights Secreted Tenascins as a Key Component of Pathogenesis
**Article Type:**	Research
**Funding Information:**	FP7 Science in Society (311846) — Prof Paul Raymond Hunter
**Abstract:**	Background: Giardia is a protozoan parasite of public health relevance that causes gastroenteritis in a wide range of hosts. Two genetically distinct lineages (assemblages A and B) are responsible for the human disease. Although it is clear that differences in virulence occur, pathogenesis and virulence of Giardia remains poorly understood.<br>Findings: The genome of Giardia is believed to contain ORFs which could encode as many as 6,000 proteins. By successfully applying quantitative proteomic analyses to the whole parasite and to the supernatants derived from parasite culture of assemblages A and B, we confirm expression of ~1,600 proteins from each assemblage, the vast majority of which being common to both lineages. To look for signature enrichment of secreted proteins, we considered the ratio of proteins in the supernatant compared with the pellet which defined a small group of enriched proteins, putatively secreted at a steady state by cultured growing trophozoites of both assemblages. This secretome is enriched with proteins annotated to have N-terminal signal peptide. The most abundant secreted proteins include known virulence factors such as cathepsin B cysteine proteases and members of a Giardia superfamily of cysteine rich proteins which comprises VSPs, HCMPs and a new class of virulence factors, the Giardia tenascins. We demonstrate that physiological function of human enteric epithelial cells is disrupted by such soluble factors even in the absence of the trophozoites.<br>Conclusions: We are able to propose a straightforward model of Giardia pathogenesis incorporating key roles for the major Giardia derived soluble mediators.
**Corresponding Author:**	Kevin Tyler<br>University of East Anglia<br>Norwich, Norfolk UNITED KINGDOM
**Corresponding Author Secondary Information:**	
**Corresponding Author's Institution:**	University of East Anglia
**Corresponding Author's Secondary Institution:**	
**First Author:**	Audrey Dubourg, PhD
**First Author Secondary Information:**	
**Order of Authors:**	Audrey Dubourg, PhD<br>Dong Xia, PhD<br>John P Winpenny, PhD<br>Suha Al-Naimi, MBChB PhD<br>Maha Bouzid, PhD<br>Darren Sexton, PhD<br>Jonathan M Wastling, PhD<br>Paul Raymond Hunter, MBChB PhD

Powered by Editorial Manager® and ProduXion Manager® from Aries Systems Corporation

	Kevin Tyler
**Order of Authors Secondary Information:**	
**Response to Reviewers:**	Responses to the Reviewers and Editor's comments Regarding: "Giardia Secretome Highlights Secreted Tenascins as a Key Component of Pathogenesis", GIGA-D-17-00132<br><br>Summary:<br>Thank you to you and your reviewers for a set of incisive comments and thorough suggestions on the manuscript. We have incorporated as far as it was possible for us to do so and as a result, we hope that you will find the paper much improved in general. The methods section in particular has been significantly improved and detailed. Fig 1 and 3 are now improved and of higher resolution, all the tables (supplemental tables included) were also updated to read protein abundance instead of protein expression and typos were corrected.<br><br>Response to Reviewer#1: (Text of Reviewer with authors' responses in italic)<br>The authors have used proteomic technologies to fill in an important gap in the research regarding the secretome of Giardia. While this is a very important and very valuable venture, the manuscript fails to adequately report on the methods used to generate their data. The core of manuscript is a quantitative proteomic experiment, therefore it must describe the methods of acquiring proteomic data, how these data were searched and analysed quantitatively, and their consistency and reproducibility. Moreover, as this experiment involves transporting Giardia from normal optimised media to co-culture conditions and non-parasite media, more information is needed on these aspects in order to verify they were adequately controlled. I have provided some overarching comments that must be addressed before further evaluation, most of which are centralised on amending the methods so they adequately report the experiment. (Thank you for your suggestions, all those questions will be answered below in your comments)<br>a) Proteomics:<br>- You have stated you have used an LTQ-Velos Orbitrap and a Q-Exactive. While these are two mass spectrometers capable of generating the high-resolution data for quantitative analyses, they aren't necessarily complementary or orthogonal platforms. What is the rationale for doing technical injections on these machines?<br>The aim was not to assess the technical differences between the two techniques but to have a better coverage and to increase the robustness of the data as preliminary data showed a huge discrepancy in the quantification when both MS were used on different replicates. Only the proteins identified by both techniques were included in the quantitative analysis to increase the robustness of the data. This was added in the results section to explain the need to use both MS techniques here.<br>The methods for the mass spectrometry, including chromatography, fragmentation regime, MS and MS2 conditions, data acquisition are missing from the methods, so it isn't possible to tell if they were run in ways to acquire different types of data. Were there differences in the way the gradient was run, or ions were acquired to justify using both?<br>Both MS were similarly set technically to have conserved parameters and three independent replicates were run on both MS as we only wanted to increase coverage and not compare both MS techniques. All the conditions and protocol were added into the proteomics section of the Methods to make it clear for the reader.<br>Were the ratios generated by each method averaged for each identification? Were they in agreement?<br>Ratios were generated from the averaged iBAQ abundance for each identification and this for each method. Ratios were usually in agreement, when ranking and analysing the quantitative data, only the Q-Exactive value used to rank proteins as the technique has been shown to be more sensitive.<br>The authors need to justify why they have used both mass spectrometers, in what way they combined the data for quantitative analyses? This needs to be discussed in the manuscript. Given that the QExactive identified nearly all the proteins in the LTQ Orbitrap runs, was there a benefit for keeping both datasets and if so this needs to be discussed.<br>See above for explanations, it was added in the results section to explain the benefit for using both techniques<br>- As stated above, the mass spectrometry methods are absent in the methods. The

Powered by Editorial Manager® and ProduXion Manager® from Aries Systems Corporation

injection conditions, chromatography gradients, fragmentation, MS and MS2 conditions, data acquisition must be included.
As stated above, all those conditions have been added to the Methods section.

- Sample preparation is not described. Methods say the trophs and s/n were separated and proteins harvested. How were the secreted proteins harvested/precipitated from the media? Was this done at Liverpool? No conditions for protein-peptide digestion are given. More information needed.
As stated above, all those conditions have been added to the Methods section. The protocol to prepare supernatant and pellet samples prior to MS was done on site before being sent to Liverpool for the MS analysis. Protein denaturation and peptide mixture preparation as well as the MS and peptides analysis and matching were done in Liverpool. Once all quantification and protein matching were done, the datasets were sent back to UEA for further analysis of Giardia secretome.
This has now been specified in the methods sections

- Please reference the genomes used for database searching, the release number from giardiadb.org and also what search software/algorithm was used for matching peptides. Please state the search conditions, such as variable and fixed modifications, tolerance, missed cleavages etc etc. This, like the mass spec, needs to be in the manuscript. I have noticed that some of these are given in the description for the PRIDE data deposit, but these also need to be in a supplementary methods or the methods themselves.
All genome references (ATCC), release number of giardiadb.org and software/algorithm used for matching peptides as well as search conditions were added in the methods section in the proteomics sub-section, under Data analysis.

- Judging from the tables the method of quantitation is iBAQ. There is no description of this in the methods or the results. The method for generating these abundance values must be in the methods and results. Some readers may not be familiar with iBAQ, so some explanation of it in general could be valuable to the readership.
Thank you for pointing that out, a brief explanation has been added in the results section and a more developed description in the methods section.

- As above, if you are choosing to keep both datasets from the mass spectrometers in the manuscript, then please start their consistency and agreement at the quantitative level of protein abundance as the manuscript only states their agreement at the identification level.
Prior to perform any proteome or secretome analysis, all quantitative datasets were compared by running a Spearman correlation test. This showed that the datasets were exploitable for a quantitative analysis of the secretome and proteome. However, to avoid any bias in the analysis, datasets were first analysed separately. Only the quantitative data from the replicates run on one or the other MS were merged. Secretion profiles were obtained for both techniques and then compared to see any discrepancy in the protein ranking between the two techniques. Apart from a slight variation in values (which is expected as the techniques are different and also the Q-Ex is more sensitive than the Velos-Orb) the ranking were the same regardless of the MS technique. We decided to put the protein abundance for both technique on the supplemental tables and to simplify Table 1 with only the Q-Ex values as it is more sensitive.

- The top 50 proteins were considered to be the most likely candidates as secreted proteins. What was the ratio cutoff that this included (min/max)?
The top50 cut-off included proteins with a ratio and some proteins with only an abundance value in their supernatant dataset suggesting that the latter were most likely secreted as their abundance in the proteome was potentially too low to be picked on any of the MSs. For the proteins with a ratio, the range between the top50 proteins varies greatly from one assemblage to the other. E.g.: from 0.3 up to 21 for WB isolate and from 1.5 to 10. Therefore, to simplify this analysis we decided to just focus on the top50 for now.

What were the ratios of currently known secreted proteins in Giardia, and could these be used (or at least discussed) as internal standards considered as an alternative method to filter candidates?

Powered by Editorial Manager® and ProduXion Manager® from Aries Systems Corporation

Since at the time of the work there was no existing gold standard study for providing "known secreted proteins" of giardia trophozoites, because many of the studies which have looked at trophozoite secretion did so in the presence of intestinal epithelial cells not from steady state supernatants and because there did not exist a consensus for which proteins are secreted and which are not we undertook the analysis "de novo" without reference to "known positive controls" which we considered might be wrong and bias any result - except insofar as we did evaluate the number of proteins predicted to have a secretion signal. Our results do however strongly support and substantiate work published in scientific reports by this reviewer in the past few years and we have now expanded the discussion with regard to known secreted proteins in Giardia highlighting this point.

- You used three biological replicates, were these used in order to filter for only reproducibly identified proteins? All the supplementary files have data at the average level? How were replicates used in the identification and quantification of proteins? You say minimum 2 peptides were needed, but what if that was found in only one of the replicates?
As the reviewer suggests we used three independent biological replicates primarily to filter for reproducibly identified proteins but also robustness of the data. If a protein was identified and quantified in only one of the three replicates, it was excluded from our final analyses.

- You need to provide a supplementary table of your proteomic identifications and quantification for your datasets of the ~1600 proteins.
We can provide these in this way, but believe that the integration of our complete dataset with EuPathDB which will be available in the next release will provide this in a far more accessible and elegant way and make any such spreadsheet appear cumbersome and redundant. The problem with accessing with the PRIDE files no double exacerbated the reviewer concern here which has now been fixed (below).

- The PRIDE deposit contains files, but there are no titles on these files? What are these? They have different numbers for accession but no titles? These are not the raw files? To what level have they been processed? Please clarify what these files are.
Thanks for looking into the PRIDE files, it appears that some of the raw files had been mixed up during the download onto the database. We contacted PRIDE IT team and got this sorted. The names of the raw files were also updated by adding the technique into the names. S stand for supernatant and P for pellet. The number 1, 2, and 3 represent the replicate number for each MS.
The Progenesis (peaks) files with only the access number refer to the Velos data as we used it as the standard technique which created files without the technique into the title.

b)Culture conditions:
- Please state throughout if you used/harvested adhered or non-adhered or total trophozoites.
Thank for you spotting that missing bit, the total trophozoites were harvested to run the experiments. This was added in both the results and methods sections.

- You say the 'reference isolates', please cite the ATCC numbers.
ATTC numbers were added in the Data description and the Methods sections

- As you are moving isolates to separate media or co-culture conditions, please state whether the trophozoites were in aerobic or anaerobic conditions, including during co-culture.
For all experiments and the standard in vitro culture of the trophozoites, parasites were under aerobic conditions with 5% CO_2. This was added into the methods section.

- There needs to be more information in the methods in regards to the experiment regarding Giardia/Giardia soluble products and the CaCO2 cells and the electrophysiology. In the Data description it says Giardia were added, in the results on page 6 is states cells were grown alone, with Giardia or with diluted supernatants. None of the information about these different conditions, their time frames or experiment design are given in the detailed methods regarding 'electrophysiology'. Not only does this need to be added, but the description of the experiment between Data

description, results and methods needs to be made consistent throughout otherwise is it too confusing.

The full methodology for the culture of the Giardia parasites, the Caco-2 cells and the electrophysiological assays has now been given in the Methods section (p12-14) of the manuscript. We apologise for the confusion caused by our description of the experimental conditions. Clarification has now been given for the different experimental conditions used for the electrophysiological assays in the Methods section (p12-14), Figure 2 legend (p18) and Analyses section (p5-7).

c)Other
- Given that VSPs are known membrane proteins, and you are looking at media fractions, have you considered the possibility they may be cleaved protein products? What sequence coverage are you getting for your VSPs, including the termini? Can you say they are indeed secreted, or could they be VSPs cleaved from the membrane during turnover?
We agree with the reviewer that this is likely, our data is consistent with at least some exterior (outward facing) plasma membrane proteins being clipped or shed from the surface rather than being actively secreted per se. We were unable to discriminate exact mechanism by which surface proteins were entering the supernatants during this study and have deliberately avoided describing them as secreted, but we are seeking funds to do exactly this using a more targeted approach and employing cell biological and transgenic as MS based methodologies.

Response to Reviewer#2: (Text of Reviewer with authors' responses in italic)
This manuscript revealed Giardia secretome under steady state of growth in vitro by proteomics methods and also confirmed that the secreted molecules adversely affect the homeostasis of enteric epithelia. The authors also concluded that Tenascins was a new class of virulence factors. This research provided a comprehensive proteomic data on Giardia secretome, introduced a novel pathogenicity mechanism, therefore is worthy of publication. However, there are still some problems needed to be revised before acceptance.
Thank you for your comments. We have modified the manuscript and hopefully improved it following your suggestions (see below)
1. More comparison between this research and previous Giardia proteomics research should be discussed, especially on the topic of whether the parasite-host cell interaction can induce the proteomic changes of the parasite.
This was indeed lacking in the discussion section, thank you for pointing it out. Comparison between our findings and the known Giardia secreted proteins has been added in the discussion section. We have in particular discussed our findings on the metabolic proteins previously shown to be secreted upon interaction with host cells. Our data shows that these specific proteins are present in both secretome and proteome but at a very low abundance in secretome and they are therefore not in the top50 cut-off. This suggests an up-regulation of their secretion upon host-cell interaction as Svard and his team suggested. The other known to be secreted proteins were also discussed.
2. The major advantage of this research is that Tenascins was defined as a new class of virulence factor of Giardia. Considering that this is the major innovation and proposed novel mechanism of pathogenicity for Giardia, the authors should try to express them in 293T cells, insect cells or in vitro none-cell cell lysis system to analyze the pathological function of the recombinant proteins. This results will strongly support the Figure 3 novel mechanism proposal.
We agree entirely with the reviewer that just this type of in vitro experiment would indeed strongly support our findings about a novel pathogenesis mechanism. However, as much as we would love to research more and add more analysis to support our findings, we cannot do that at the moment due to lack of funding. We have included just such experiment in our current grant application along with the provision of transgenic mutants which will enable structure/function analysis.
3. Figure 1 is not necessary and can be transferred as a supplement figure. Other analysis, such as alignment of protein function domains, signalling peptide prediction also can be included in this figure. To support the proposed novel mechanism of pathogenicity, the authors should pay more attention on the protein functions, such as function domain, 2D and 3D structure, etc.
We thanks the reviewer for these ideas, we have of course begun to look closely at the

Powered by Editorial Manager® and ProduXion Manager® from Aries Systems Corporation

tenascins which are an interesting new family of mimics as well as the cathepsins and in particular and their recent evolutionary history. We think the key findings about the protein are adequately incorporated in the figure and will aid the understanding of the general reader. Simple structural analysis does not show yield much more than we have described unfortunately – the proteins homology to actual tenascins are limited to the EGF domains where are fully intact in most cases. The proteins do appear to have emerged under selective pressure from common ancestors with HCMPs and VSP proteins and they are polyphyletic. In the future we hope that by expressing recombinant versions of the proteins we can deduce and evidence more about their structure of these proteins but this is I think well beyond the scope of this paper.

Response to Reviewer#3: (Text of Reviewer with authors' responses in italic)
From the biological point of view, the results are sounding and interesting. The manuscript is well presented. The choices of methods and accuracy of the procedure are globally good; the comprehensive of description of methods must be detailed. The authors definitively identify many proteins using mass spectrometry, some of which may be important for the interaction of Giardia lamblia with host cell. The manuscript is lacking any confirmation of these secreted proteins by another biological technique; however, that may not be trivial given the potential lack of antibodies.
However, I have pointed out below several concerns I have regarding this publication. The scientific content of this manuscript is interesting. It is advised to correct all the typing errors (Some corrections that I found, I indicated) and to have a last editing of the manuscript. I suggest improving the quality of the figures (as 1, 3). Please do not write protein name with uppercase (for example: replace some Cathepsin by some cathepsin).
Considering all together I can state that several points need clarification or revision before final decision.
Thank you for your comments and suggestions. We have modified the manuscript and hopefully improved it following your suggestions (see below)

ABSTRACT
Please change "N' terminus signal peptides" by "N-terminal signal peptide" and in the main text as well.
This was modified throughout the text and in the abstract.
ANALYSES
5/3: The use of expression is not appropriate here, in the main text and in the suppl. table. It is not "expression" but "protein abundance". Moreover, the term "SP expression" does not sound good to explain the more abundant proteins identified from the supernatant.
This changed throughout the text as well as in all the tables (including supplemental. tables)
The authors should explain what they used to do the ratio SP/P to compare the "abundance" of proteins. What did the value considered? Did they use the PSMs? In fact, you used iBAQ algorithm but it is not mentioned in the main text.
The iBAQ algorithm and its meaning were indeed missing from the main text, thank you for letting us know. We have added an explanation for our abundance value and iBAQ in the results section as well as the Methods section. The formula for the ratio was modified to state Sp or P abundance-iBAQ as it is what we used for the analysis. We hypothesised that proteins with a ratio ≥ 1 would be more likely secreted as enriched in the supernatant and would therefore be included in the analysis if the proteins were part of our top50 cut-off.
DISCUSSION
The authors could discuss about the high number of proteins from the supernatants; why they identified some proteins only from the supernatants since proteins are synthetized in the cell; and why they used two different mass spectrometers and they could justify the choice of these 2 spectrometers in function of the different results.
Not many proteins were identified only in the supernatant, between 10 to 20 proteins which is fairly low compared to the 1,600 proteins identified in total. However, this could be surprising and yet expected if those proteins are mainly secreted, their abundance in the cytosol would be so low compared to other proteins that it would not be picked on by the MS with the default settings, this show the limitations of the MS techniques.
We used two different MS due to preliminary data showing major discrepancy between replicates, which is why we decided to use both MS to increase coverage and

Powered by Editorial Manager® and ProduXion Manager® from Aries Systems Corporation

	robustness of the analysis. This has now been explained in the results and discussion sections.<br>The authors discussed the function of the more abundant secreted proteins, but did they find other proteins known secreted that could be confirm these results?<br>This was missing in our analysis and our discussion. Thank you for suggesting it. We have now added comparison between our dataset and the known secreted proteins in Giardia.<br>METHODS<br>it should be indicated the origin of the strains, and if the parasites are subjected or not to regular passages in animal (for example mice).<br>This was added into the Methods section, parasites were not passed through any animals, only in vitro in glass tubes. This was specified in the methods and results.<br>The Proteomic Analysis must be detailed: Cell lysis; Protein trypsin digestion; Peptide separation by reverse-phase chromatography; Mass spectra acquisition for both mass spectrometers; data analysis (.raw and protein interpretation).<br>The proteomics protocol was expanded and greatly improved to include all the processes in the Methods section<br>Did the authors work with the same number of cells from each strain? Quantity of proteins and peptides? How did the authors quantify the proteins and peptides?<br>As we wanted to have a protein concentration (determined by BCA assay) as high as possible and the "cleansing" of the supernatants by incubation in serum-free DMEM before collecting Pellet and supernatant samples may have caused stress onto the trophozoites, we decided to not work with a conserved number of cells but with the total trophozoites in mid-log phase growth. However, after protein digestion, 1 µg of digest was injected in each MS and this for all replicates and each strain.<br><br>9/15: should change "SDS page electrophoresis and..." by "SDS PAGE and..."<br>This was changed<br>9/21: What the cut-off of the vivaspin filter? 5000 MWCO? change Ambic by ammonium bicarbonate<br>The cut-off of the columns was 3000 MWCO, this was added in the manuscript. Ambic was modified as well and then the abbreviation added<br>9/28 and other place: should change giardiaDB.org by giardiaDB.org<br>Not sure what needed to be changed here if it was GiardiaDB to giardiaDB or vice-versa.<br>9/47 and 10/10: should change "mls" by "ml".<br>This has been done<br>10/6: Consider the following sentence: "...GlyH-101 (50mM) and" what?<br>We have extended and clarified the sentence to read, "Stock solutions of Amiloride (10mM), GlyH-101 (50mM) were made by dissolving in DMSO."<br>The sentence was corrected<br>10/27: should write Giardia in italic<br>This has been done<br>10/32: Abbreviations are incomplete, for example FDR<br>All abbreviations were added in the abbreviations section and also in the text when required<br>14/7: Delete S in the sentence: "…Q-Exactive S Supernatant (S) abundance, from most to least abundant…"<br>This has been done.
**Additional Information:**	
**Question**	**Response**
Are you submitting this manuscript to a special series or article collection?	No
**Experimental design and statistics**<br><br>Full details of the experimental design and statistical methods used should be given in the Methods section, as detailed in our Minimum Standards Reporting Checklist. Information essential to interpreting the data presented should be made available	Yes

Powered by Editorial Manager® and ProduXion Manager® from Aries Systems Corporation

in the figure legends.<br><br>Have you included all the information requested in your manuscript?	
**Resources**<br><br>A description of all resources used, including antibodies, cell lines, animals and software tools, with enough information to allow them to be uniquely identified, should be included in the Methods section. Authors are strongly encouraged to cite Research Resource Identifiers (RRIDs) for antibodies, model organisms and tools, where possible.<br><br>Have you included the information requested as detailed in our Minimum Standards Reporting Checklist?	Yes
**Availability of data and materials**<br><br>All datasets and code on which the conclusions of the paper rely must be either included in your submission or deposited in publicly available repositories (where available and ethically appropriate), referencing such data using a unique identifier in the references and in the “Availability of Data and Materials” section of your manuscript.<br><br>Have you have met the above requirement as detailed in our Minimum Standards Reporting Checklist?	Yes

Manuscript

Click here to download Manuscript resubmission of giardia secretome changes tracked .docx

Title: ***Giardia*** **Secretome Highlights Secreted Tenascins as a Key Component of Pathogenesis**

Running Title: *Giardia* Secretome

Authors: **Audrey Dubourg[a], Dong Xia[b], John P. Winpenny[a], Suha Al Naimi[a,c], Maha Bouzid[a], Darren W. Sexton[a,d], Jonathan M. Wastling[b], Paul R. Hunter[a] and Kevin M. Tyler*[a]**

Authors' affiliations:

a. NIHR Health Protection Research Unit in Gastrointestinal Infections, Norwich Medical School, University of East Anglia, Norwich, UK.

b. Department of Infection Biology, Institute of Infection and Global Health, Faculty of Health & Life Sciences, University of Liverpool, UK.

c. c. Department of Science and Technology, Faculty of Health and Science, University of Suffolk, Ipswich, UK

Commented [SA1]:

School of Science, Technology and Health, University Campus Suffolk, UK

d. Liverpool Moore's University, Liverpool, UK.

Authors email:

AD: adubourg@g.ucla.edu

DX: D.Xia@Liverpool.ac.uk

JPW: jJohn.wWinpenny@uea.ac.uk

Field Code Changed

SAN: s.al-naimi@ucs.ac.uk

MB: M.Bouzid@uea.ac.uk

DWS: D.W.Sexton@ljmu.ac.uk

JMW: j.wastling@keele.ac.uk

PRH: Paul.Hunter@uea.ac.uk

KT: k.tyler@uea.ac.uk

*Correspondence: k.tyler@uea.ac.uk,

Tel +44 (0)1603-591225,

Fax: +44 (0) 1603 591750.

Abstract

Background: *Giardia* is a protozoan parasite of public health relevance that causes gastroenteritis in a wide range of hosts. Two genetically distinct lineages (assemblages A and B) are responsible for the human disease. Although it is clear that differences in virulence occur, pathogenesis and virulence of *Giardia* remains poorly understood.

Findings: The genome of *Giardia* is believed to contain ORFs which could encode as many as 6,000 proteins. By successfully applying quantitative proteomic analyses to the whole parasite and to the supernatants derived from parasite culture of assemblages A and B, we confirm expression of ~1,600 proteins from each assemblage, the vast majority of which being common to both lineages. To look for signature enrichment of secreted proteins, we considered the ratio of proteins in the supernatant compared with the pellet which defined a small group of enriched proteins, putatively secreted at a steady state by cultured growing trophozoites of both assemblages. This secretome is enriched with proteins annotated to have ~~N' terminus signal peptides~~N-terminal signal peptide. The most abundant secreted proteins include known virulence factors such as cathepsin B cysteine proteases and members of a *Giardia* superfamily of cysteine rich proteins which comprises VSPs, HCMPs and a new class of virulence factors, the *Giardia* tenascins. We demonstrate that physiological function of human enteric epithelial cells is disrupted by such soluble factors even in the absence of the trophozoites.

Conclusions: We are able to propose a straightforward model of *Giardia* pathogenesis incorporating key roles for the major *Giardia* derived soluble mediators.

Keywords: *Giardia*, Secretion, Proteomics, Quantitative Proteomics, Tenascin, Cysteine protease, Enteric Pathogen.

Formatted: Font: Italic

Formatted: Font: Italic

Background

With some 280 million symptomatic cases, giardiasis causes more bouts of human illness than any other parasitic disease [1]. The mechanism and mediators of pathogenesis by *Giardia*, however, remain largely unknown. Thanks to human volunteer studies, the association of *Giardia* infection itself and the significance of the virulence of the infecting *Giardia* strain, is experimentally unambiguous [2]. The molecular definition associated with strain virulence is though largely unexplored. It is clear that the majority of *Giardia* infections are asymptomatic. It is also clear, that infection is primarily localized to the duodenum and that some localized damage close to the sites of colonization cause villus atrophy and apoptosis of surrounding cells. However, this localized damage cannot be the sole cause of the profound diarrhoea which is often characteristic of the disease and which appears to affect absorption over a much wider area of the digestive tract than the site of infection alone.

One of the secreted mediators of damage to the duodenum is believed to be cathepsin B protease [3]. Cathepsin B-like proteases compose one of the superfamilies belonging to the CA clan of cysteine peptidases [4]. Compared to other cathepsins, cathepsin B proteases possess an additional 20 amino acid insertion named the occluding loop that enables their function as an endo- or exopeptidase [5]. Although twenty-seven genes encoding cathepsin proteases have been identified in *Giardia*, for the majority of these proteases, functions still remain elusive [6]. While some parasites may secrete cathepsin B proteases to either evade or modulate their hosts immune responses [7], a recent study has demonstrated that *Giardia* trophozoites secrete cathepsin B-like proteases, degrading intestinal IL-8 and thereby reducing the inflammation reaction by the host [3]. Secreted *Giardia* cathepsin B protease *(G*CATB*)* may also facilitate trophozoites attachment to intestinal epithelia [8] and contribute to degradation of intestinal mucin [9].

Most of the proteomic studies so far reported for *Giardia* were undertaken in trophozoites undergoing encystation [10-12]. Only a few studies have focused on proteins secreted by *Giardia* and their role in the host-pathogen ~~interaction~~interaction [3, 13-15]. These studies were focused on parasite interaction with intestinal cell lines. No studies have yet attempted to quantify proteins which are the product of steady state secretion by healthy, growing *Giardia* trophozoites and which we hypothesize as the primary mediators of giardiasis pathology. In this study, we have identified, to the limit of existing technology, the proteins expressed by populations of healthy, growing human infective *Giardia* trophozoites. We have provided quantitation of the relative abundance of retained and released trophozoite proteins from two human infective assemblages, affording calculation of the specific enrichment of released proteins and thereby the description of which proteins are most likely to be secreted by trophozoites of each assemblage. Thereafter, we compared the profile of enrichment between the two assemblages in order to identify conserved as well as assemblage-specific secreted proteins. We provide electrophysiological analysis which confirms that trophozoite secreted molecules adversely affect the homeostasis of enteric epithelia and our analysis of the heterogeneity of encoding genes between lineages demonstrates the direct selective pressure on these

virulence factors and affords their use in discriminating clinically important strains and outbreaks. Finally, the discovery of tenascins as a highly represented and variable group of proteins secreted by trophozoites strongly implicates this new class of virulence factors in a novel model for the mechanism of *Giardia* pathogenesis. We propose that their role follows degradation of the protective mucous afforded by the action of a secreted nuclease and *G*CATB, and following damage to cellular junctions by *G*CATB. Tenascins acting to prevent repair to those damaged junctions as a result of the EGF receptor ligation.

Data description

Soluble and cytosolic fractions from *in vitro* grown assemblage A and B trophozoites, the aetiologic agents of human giardiasis, were extracted in order to establish which proteins are secreted in the steady state by healthy, growing trophozoite populations. We reasoned that secreted proteins would be overrepresented in the medium in which parasites were incubated compared with the trophozoites that produced them. This ~~apparently~~ ostensibly straightforward assessment being reliant on the sensitive, specific and quantitative detection of the proteins expressed by *Giardia* trophozoites in whole cells~~, compared with extracts of *Giardia* proteins from~~ and in the medium in which the trophozoites were incubated.

The WB (assemblage A – ATCC_50803) and GS (assemblage B – ATCC_50581) reference strains were utilized to facilitate ease of comparison between genetically divergent human infective isolates with the available reference genomes. For each experiment trophozoites were harvested from mid log growth and incubated in non-supplemented Dulbecco's Modified Eagle medium (DMEM) for 45 minutes at 37°C before supernatants and pellets were collected for proteomic and other analyses including validation of their viability by flow cytometry (Additional file 1: Fig S1). Proteomic analyses were based on samples from 3 distinct biological replicates. Each sample was analysed using two quantitative proteomic platforms the Orbitrap MS and the Q-Exactive MS. Thus, in total the results from 24 (2 × 2 × 2 × 3) proteomic analyses are reported.

Formatted: Font color: Auto

The identification of abundant, secreted, *Giardia* virulence factors led us to consider whether the secretions from *Giardia* alone could effect changes in the behaviour of enteric epithelia - even in the absence of the trophozoites themselves. I~~Thus, i~~n order to determine the effect of *Giardia* trophozoite secreted factors on the intestinal epithelia, chopstick type electrodes connected to a voltmeter were used to measure the trans-epithelial electrical resistance (TEER) of polarised CaCo-2 epithelial cells grown on permeable supports. CaCo-2 cells were cultured over 6 days until confluent. TEER across the developing CaCo-2 monolayer was measured on a daily basis as shown in Figure 2A. Once confluence was established *Giardia* trophozoites were added to the apical side of the confluent epithelium and after 24 hours incubation the trophozoites were washed from the apical surface. In order to determine whether or not co-cultures of *Giardia* trophozoites or diluted *Giardia* supernatants of both human assemblages affected ion channels responsible for secretory movement across the epithelium, an Ussing chamber system was utilised with different chloride secretion inhibitor and activators.

Further details about sample collection, secretome analysis and electrophysiology can be found in the methods section.

Analyses

Protein expression in *Giardia* trophozoites

~~To obtain *Giardia* secretome with high confidence and robustness of the data, two MS techniques were used: Q-Exactive and Orbitrap MS. Preliminary data obtained from different replicates using either Q-Exactive or Orbitrap MS techniques separately showed very low correlation between the datasets and high discrepancy in the absolute abundances of the proteins (*data not shown*). T~~Therefore, ~~t~~o ~~obtain~~describe definitive *Giardia* secretomes under a standard set of conditions with high confidence and based on a robust data set ~~and robustness of the data~~and two reduce the potential for technical artifact~~,~~ the two MS techniques: Q-Exactive and Orbitrap MS were used with similar settings on the same three independent replicates to increase coverage. Only proteins identified by both techniques within the three replicates datasets were included in the analysis to increase the robustness of the data. The protein quantification was ~~done~~ performed using a label-free method: iBAQ (intensity based absolute quantification) which calculates the sum of parent ion intensities of identified peptides per proteins ~~(~~[16]. The average normalised abundance was divided by the iBAQ values giving the "Abundance-iBAQ". The quantitative datasets from both MS techniques and for each independent replicate were shown to be strongly correlated by a Spearman correlation test (Data not shown) and therefore exploitable for proteomics analysis.

Formatted: Not Highlight

The Q-Exactive MS identified almost all of the proteins identified by use of the Orbitrap MS, and in total the two techniques identified 1,587 GS proteins and 1,690 WB proteins (Additional File 1: Fig S2). This represents over a quarter of the open reading frames (ORFs) predicted by the respective genomes in this single life-cycle stage under this steady state set of in vitro culture conditions and compares favourably with other recent proteomic analyses of *Giardia* [17, 18]. Lists of proteins detected in only one of the two assemblages are provided (Additional file 2: Table S1 and S2). ~~Expression~~ Protein abundance from two of the 8 predicted assemblage-specific genes previously identified by comparative genomics was detected [19].

Overall, both assemblages gave comparable and consistent results by both platforms with the sensitivity of detection being greater for Q-Exactive MS; which provided a range of detection spanning 5 logs. In total, Q-Exactive MS identified 1,542 GS proteins and 1,641 WB proteins (Fig S3). Of these, 946 GS proteins were present in both pellet and supernatant, 27 in the supernatant only and 569 GS proteins in pellet only. By comparison 490 WB proteins were identified in supernatant and pellet and 24 in the supernatant only with 1,127 WB proteins in pellet only.

Giardia secretome

To evaluate supernatant enrichment, proteins identified in the supernatant (SP) datasets were gathered and compared to their concentration in the pellet (P) to provide a ratio

using the following formula: $\frac{SP\ abundance-iBAQ\ \text{~~Expression~~}}{P\ abundance-iBAQ\ \text{~~Expression~~}}$. These proteins were then ranked from highest to lowest by ratiometric value and an arbitrary cut-off invoked such that the top 50 were considered as the most likely to be secreted. Proteins identified only in SP were also included in the analysis as most likely to be secreted. All the proteins selected as "of interest" were ranked according to their SP expression from most to least abundant to obtain a quantitative enrichment profile for each isolate and this was performed for each platform. Orbitrap and Q-Exactive enrichment profiles were compared and proteins were considered as most likely to be enriched in the supernatant when identified as such by Q-Exactive MS and confirmed by Orbitrap MS. The different enrichment profiles were then also compared between assemblages.

The results showed a set of 15 orthologous proteins that were identified in both isolates by both techniques (Table 1). Eleven of these were predicted to possess an N-terminal signal sequence. Just two of these were of unknown function and two groups dominated the annotated genes encoding the rest of these proteins, five were annotated as tenascins and three as cathepsin B cysteine proteases. The most abundant enriched protein was found to be pyridoxamine 5'-phosphate oxidase (PNPO), an FMN dependent enzyme capable of fixing molecular oxygen that lacks a signal peptide and which was also recently identified as a secreted *Giardia* trophozoite protein upregulated during interaction with epithelial cells[15]. An extracellular nuclease was also present, along with a high cysteine membrane protein and a protein annotated as a VSP but which was well conserved between assemblages.

We considered that where proteins were shown to be enriched in the supernatant using both platforms and in both assemblages and possessed an N-terminal signal sequence that they were truly secreted proteins. Secreted proteins involved in adapting *Giardia* to the host environment of the human gut might be expected to be engaged in Red Queen evolution and have dN/dS indicative of positive selection. While amino-acid divergence between orthologs of secreted proteins varied considerably from 67% for the HCMP to 83% for (e.g. for the extracellular nuclease), only three proteins showed evidence of positive selections, two tenascins and one of the cathepsins. One cathepsin and one tenascin in particular showed evidence of evolution under a very high degree of selective pressure (Table 1). Although, interestingly, some cathepsins and some tenascins with similar levels of amino-acid identity between the assemblages to those under high selective pressure showed little or no evidence of positive selection.

We considered whether lineage specific soluble mediators might also be present and identified by this method. Comparing those proteins identified by both methods as having the highest relative expression in the supernatant (Tables S3 and S4). The five most abundant conserved secreted proteins from Table 1 were also present in the top 10 secreted proteins from each assemblage amongst other VSPs, tenascins, and cathepsin B, and this regardless of the MS technique or the isolate. Not unexpectedly VSPs were the primary proteins enriched in supernatants that were lineage-specific. Amongst the multigene families, however, there were also differences in the cathepsin B and tenascins/HCMP repertoires. No other proteins with N-terminal peptides were encoded in

either assemblage except for one CxC-rich protein. Interestingly none of the proteins encoded by assemblage-specific genes and identified by comparative genomics were found to be enriched in the supernatants.

Giardia soluble mediators disrupt intestinal cell functions

Soluble and diffusible agents, able to disrupt gut function, could potentially mediate more diffuse and profound pathology for giardiasis than close range interactions between the trophozoites and the gastrointestinal epithelium alone. To determine whether *Giardia* secreted virulence factors could induce changes in the behaviour of intestinal epithelium, short-circuit current (Isc) was continuously measured across polarised CaCo-2 epithelial cells that had either been cultured without any additions, co-cultured with *Giardia* trophozoites or co-cultured with diluted (1:1000) *Giardia* supernatants (Figure 2B). Further experiments demonstrated that either after 24 hour co-culture with *Giardia* (Fig 2C) or 24 hour co-culture with diluted *Giardia* supernatants (Fig 2D) both experimental conditions dramatically inhibit both the cAMP-stimulated Isc (basolateral application of 10 µM Forskolin) and the calcium-activated Isc (basolateral application of 100 µM UTP). In order to identify what ion channels where being affected, the CFTR chloride ion channel inhibitor, GlyH101 (50 µM), and the calcium-activated chloride ion channel inhibitor, DIDS (100 µM), were added to the apical side of the Ussing chamber. The cAMP-stimulated Isc is predominantly due to activation of CFTR chloride channels as it is inhibited by GlyH101 (Figure 2B-D). The calcium-activated Isc is predominantly due to activation of calcium-activated chloride channels as it is inhibited by DIDS (Figure 2B-D).

Soluble and diffusible agents, able to disrupt gut function, could potentially mediate more diffuse and profound pathology for giardiasis than close range interactions between the trophozoites and the gastrointestinal epithelium alone. To determine whether *Giardia* secreted virulence factors could induce changes in the behaviour of intestinal epithelium, short-circuit current (Isc) was continuously measured across polarised CaCo-2 epithelial cells that had either been grown alone, co-cultured with *Giardia* trophozoites or exposed to diluted (1:1000) *Giardia* supernatants (Figure 2B). Further experiments demonstrated that either after 24 hour co-culture with *Giardia* (Fig 2C) or exposure to diluted *Giardia* supernatants alone (Fig 2D) both experimental conditions dramatically inhibit both the cAMP-stimulated Isc (basolateral application of Forskolin) and the calcium-activated Isc (basolateral application of UTP). In order to identify what ion channels where being affected, the CFTR chloride ion channel inhibitor, GlyH101, and the calcium-activated chloride ion channel inhibitor, DIDS, were added to the apical side of the Ussing chamber. The cAMP-stimulated Isc is predominantly due to activation of CFTR chloride channels as it is inhibited by GlyH101 (Figure 2B-D). The calcium-activated Isc is predominantly due to activation of calcium-activated chloride channels as it is inhibited by DIDS (Figure 2B-D).

Discussion

In this study, we have identified many proteins as secreted by trophozoites and thus forof both human infecting assemblagese A and B. Contaminating host However, one problem encountered was the high extent of ssserum proteins (mainly bovine albumin) in the supernatant samples were a concern,, as previously described by others ([20]). Such serum

Formatted: Not Highlight

proteins bind to the parasite's surface and are continuously released which interfere with the characterisation of *Giardia* secretome. To overcome this issue, parasites were cleansed from the serum proteins and incubated in serum-free DMEM before collecting supernatants and pellets. To increase coverage and robustness of the analysis, two mass spectrometers (Orbitrap and Q-Exactive MS) were used on the same replicates and proteins identified by both MS were including in the analysis.

Previous studies have either focused on protein secretion during *Giardia* trophozoite encystation~~[10-12]~~ or protein secreted upon interaction with or attachment to host cells~~[3, 13-15]~~~~.~~ ~~Here~~, but we have chosen here to provide a detailed baseline from cultured~~demonstrated that~~ *Giardia* trophozoites secretinge~~e~~ proteins under a steady state~~ of growth~~ *in vitro*. Nevertheless, our results are strongly supportive of recent proteomic study looking at the effect of host attachment on the profile of giardia secreted proteins [15]. Prior to that study, several metabolic enzymes had been proposed to~~Interestingly, the proteins previously identified as~~ be released by giardia trophozoites~~ secreted~~ upon interaction with intestinal epithelial cells (IEC) [13]: e.g. arginine deiminase (ADI), enolase, and ornithine carbamoyltransferase (OCT) which were all identified ~~as most likely to be secreted in~~ from ~~trophozoites~~ culture supernatants of both assemblages.

~~Yet, their protein abundances were below the top50 cut-off used here which strongly supports their up-regulation upon interaction with host cells (Data not shown). Furthermore, the fact that those metabolic enzymes have been identified in the secretion profile of both human assemblages trophozoites suggests that this up-regulation may be a conserved pathogenic mechanism involved in the colonisation of a host intestinal environment.~~Our study does confirm the previously observed enrichment of

~~Prior to this study, only one protein,~~ elongation factor 1-alpha (EF-1α), ~~had been identified as secreted in the *in vitro* growth serum of~~in assemblage A culture supernatants~~trophozoites~~ [20]~~. One lineage-specific EF-1α was also identified as secreted in high abundance in assemblage A (WB) trophozoites only suggesting an assemblage-specific mechanism~~ (Table S4). EF-1α is a key enzyme in the protein synthesis process in eukaryotic cells [21] but many ~~. Many~~ organisms have been shown to express EF-1α in excess which suggests that this protein may have some other functions [21]. In the context of pathogenicity and virulence, the secreted *Leishamnia* EF-1α was shown to down-regulate the host inflammatory cell signalling [22]. In *H. pylori*, virulence factor CagA was shown to be translocated into the host cell and disrupt the epithelial cell polarity [23]. In *Giardia*, EF-1α has been shown to be an immunoreactive protein recognised by antibodies from patients who have previously had giardiasis [20]. Yet, its role as secreted virulence factor in *Giardia* pathogenesis remains still elusive. ~~The fact T~~that this protein is only secreted by assemblage A trophozoites ~~also~~ raises the ~~question of~~possibility of explaining any observable differences in pathogenesis or host range between the ~~different~~two human infective assemblages~~ known to day and more importantly between the two assemblages causing human giardiasis.~~

Formatted: Font: Italic

Formatted: Font: Italic

Formatted: Font: Italic

Formatted: Font: Italic

Formatted: Font: Italic

The most abundant proteins, in both human isolates, ~~mainly~~ primarily belong to four families of proteins: *Giardia* cathepsin B family *(*GCATB*)*, high cysteine membrane proteins (HCMPs), variant surface proteins (VSPs) and tenascins.

The cathepsin B family of *Giardia* are confirmed virulence factors involved in many of the parasite's processes such as encystation and excystation [6]; secreted GCATBs degrade host IL-8 and inhibit neutrophil chemotaxis ~~(~~[3]~~)~~. GCATB contains secreted and non-secreted trophozoite expressed proteins; the orthologues of which are predominantly common to GS (B) and WB (A) assemblages (Fig 1). Expression of sixteen *G*CATBs was proteomically confirmed, of which eleven were shown by our proteomic analysis to be secreted. These eleven fell into six orthologous groups and for three of these groups all group members were shown to be secreted. Secreted *G*CTAB GL50803_15564 (WB) and its ortholog GL50581_2036 (GS) show a dN/dS values of >26 indicative of strong positive selective pressure. Interestingly when GS was resequenced GL50803_15564 was found to comprise three recently diverged orthologs (GSB_153537, GSB_155477, GSB_150353) and it may be that the positive selection pressure observed has been generated as a result of recent gene duplications in the assemblage B strain. GL50803_16779, an assemblage A (WB) GCATB, has previously been shown to be up-regulated and involved in trophozoite motility in early pathogenesis of *Giardia* ~~(~~[15]~~)~~. In this study, this protein was found to be in WB top 5 secreted proteins (Table S4); ~~however~~i~~, i~~ts GS ortholog (GL50581_78) was also present but at a considerably lower level suggesting that for this~~ but not part of the cut-off. This suggests that this specific~~ GCATB may play a more significant role~~play a more important role in the trophozoite motility fo~~in~~r~~ assemblage A than assemblage B~~ in early pathogenesis~~.

HCMPs are an enigmatic group of proteins with few associated functional studies. They may protect trophozoites against proteolysis [24, 25] and oxidative damage [26]. In *Giardia*, it appears that one lineage of HCMPs has given rise to the VSPs, whilst another has given rise to a group with high homology to mammalian tenascins. Tenascin, VSPs and HCMPs are then related multi-gene families which together form the largest group of proteins enriched in the *Giardia* supernatants. Interestingly, when aligned and analysed phylogenetically the secreted tenascins segregate into a monophyletic group (Figure S4). Both WB and GS orthologs of five tenascin gene products were secreted and in WB two other secreted tenascins were also detected that were not detected for the GS strain (Figure 1B).

VSPs are well-characterised surface glycoproteins with transmembrane domains, which are expressed one at a time by *Giardia* trophozoites through an RNAi regulated mechanism. They are quintessential virulence factors, responsible for antigenic variation. VSPs are hypervariable by nature and thus it is to be expected that they do not form orthologous pairs. This was the case for most we observed, intriguingly though, a few proteins annotated as VSPs were conserved between isolates suggesting that they are not actually VSPs and would not be subject to "one at a time" control expression - but are actually misannotated HCMPs which may have a conserved function in both GS and WB isolates. This study does was not able to resolve whether the enrichment of such proteins in the supernatant observed is as a result of clipping or shedding from the parasite surface or whether the proteins are also secreted.

Formatted: Not Highlight

Formatted: Font: Italic

Formatted: Not Highlight

Tenascins are characterised by the presence of epidermal growth factor (EGF) repeats and are able to act as ligands for EGF receptors. Mammalian tenascins are extracellular matrix proteins that modulate cell adhesion and migration [27]. They appear to have evolved from a group of proteins specific to vertebrates, presumably co-evolving with the EGF receptor and so the presence of homologous proteins in *Giardia* evolving independently from HCMPs is a clear example of the kind of convergent evolution best described as molecular mimicry. Interestingly, one secreted giardia tenascin (WB-GL50803_8687/GS-GL50581_4316) identified as conserved between the two human assemblages along with another giardia tenascin (WB-GL50803_14573/GS-GL50581_1475) identified as secreted in assemblage A only (Table 1 and S4) ~~have recently been shown to be involved in~~were found to be induced by host soluble factors and implicated in regulation of~~ (i) the determent of~~ trophozoites attachment ~~and (ii) the induction of virulence factors expression upon contact with host soluble factors~~ [15] supporting the case for~~. This suggests that~~ secreted tenascins ~~may be~~acting as virulence factors ~~involved in~~in *Giardia* pathogenesis.

Formatted: Font: Italic

Most published studies concerning host cell-*Giardia* interactions have focused on the effects on the host intestinal epithelia upon attachment of the trophozoites to the cells. In this study, we have shown that diluted supernatant obtained from the steady growth of *Giardia* trophozoites in vitro has an effect on the intestinal cell function. The effect observed on chloride secretion by *Giardia* supernatants indicates that *Giardia* secretes a soluble factor which is likely affecting secretion across the intestinal epithelial cells. Physiologically, cultured intestinal cells show sensitivity to *Giardia* proteins released by the parasite even at high dilution. Fig2D demonstrates that intestinal epithelial cells when acutely exposed to such *Giardia* proteins lose the ability to stimulate CFTR and calcium-activated chloride channels. The clear implication being that virulence determinants released from *Giardia* trophozoites interact with epithelial cell receptors and ion channels.

In this analysis, we have identified the proteins which are secreted by human infective *Giardia* trophozoites. Just two groups form the majority of these proteins: *G*CATBs and the HCMP superfamily encoding known virulence factors in addition to an abundant extracellular nuclease and an oxygen fixing enzyme. The elucidation of this group of proteins dramatically increases our understanding of the pathogenic mechanisms underlying giardiasis at a molecular level. The genes encoding GCATBs and HCMP superfamily proteins are among the most heterogeneous of all genes between assemblages. Their probable role in interaction with the host and luminal environment is supported by the very high dN/dS values of some family members. Correlation of variation within assemblages at these loci with strain virulence is the essential next step for their use in diagnosis of virulent strains, risk assessment and disease prognosis.

Our results indicate that *Giardia* secretions are sufficient to disable normal function in enteric epithelial making cells less able to extract fluids from the lumen. In particular, they implicate PNPO, an extracellular nuclease, *G*CATBs and tenascins. The fact that both extracellular nuclease and *G*CATBs can be involved in the degradation of the intestinal mucus layer and that both *G*CATBs and tenascins can be associated with intestinal intracellular junction disruption suggests collaboration between these proteins. Therefore, we propose a pathogenic mechanism (Fig 3) whereby PNPO produces a reducing

environment optimal for trophozoite growth, the extracellular nuclease degrades the outer layer of the intestinal mucus improving access for *G*CATBs for further degrading the protective mucous barrier and then disrupting the intestinal intracellular junctions. Lastly, tenascins are involved in maintaining intestinal cell separation by attaching to the EGF receptors present at the surface of intestinal cells and exacerbate epithelial damage by increasing the level apoptosis amongst these more detached cells. Once the intestinal barrier is breached by these mechanisms the sites of damage become prone to secondary infection by other opportunist microbes resident in the intestinal lumen and sensitive to irritation by allergens in foodstuffs leading to further inflammation and to the characteristic symptoms of the disease. Further investigations are necessary to verify this potential mechanism of pathogenesis of giardiasis.

METHODS

Formatted: Font: Bold

Proteomic Analysis.

Samples preparation

Formatted: Font: Not Italic

Giardia trophozoites from the genome reference strains WB (assemblage A,ATCC 50803) and GS (assemblage B, ATCC 50581), were cultured in TYI-S-33 under standard conditions (5% CO_2)[28] and harvested during the midlog phase of their *in vitro* growth curves. The total trophozoites (adhered and non-adhered) were washed 3 x in phosphate buffer saline (PBS) and then incubated in non-supplemented DMEM, with antibiotics to conserve an axenic milieu, for 45 minutes at 37°C. After incubation, an aliquot was analysed by flow cytometry to evaluate the viability of the *Giardia* samples. Trophozoites and supernatant were separated by centrifugation and both trophozoite pellet and supernatant were harvested. Proteins contained in supernatant were concentrated in Vivaspin columns (3,000 MWCO) with 25 mM ammonium bicarbonate (Ambic). Supernatants were analysed by SDS PAGE and were tested on cultured epithelial cells (Caco-2) to ensure the presence of proteins and biological activity (see below). Supernatants and pellets were sent to the Institute of Infection and Global Health at the University of Liverpool for mass spectrometry analysis (Figure S3).

Field Code Changed

Protein samples were dispensed into low protein-binding microcentrifuge tubes (Sarstedt, Leicester, UK) and made up to 160 µl by addition of 25 mM Ambic. The proteins were denatured using 10 µl of 1% (w/v) RapiGest^{TM} (Waters MS Technologies, Manchester, UK) in 25 mM Ambic followed by three cycles of freeze-thaw, and two cycles of 10 min sonication in water bath. Sample was then incubated at 80 °C for 10 min and reduced (addition of 10 µl of 60 mM DTT and incubation at 65 °C for 10 min) and alkylated (addition of 10 µl of 180 mM iodoacetamide and incubation at room temperature for 30 min in the dark). Trypsin (Sigma-Aldrich, Dorset, UK) was reconstituted in 50 mM acetic acid to a concentration of 0.2 µg/µl. Digestion was performed by the addition of 10 µl of trypsin to the sample followed by incubation at 37 °C overnight. The RapiGest^{TM} was removed from the sample by acidification (1 µl of trifluoroacetic acid and incubation at 37 °C for 45 min) and centrifugation (15,000 × *g* for 15 min). After protein digestion, 1 µg of digest were injected into both the Velos-Orbitrap and the Q-Exactive MS, for all samples.

Velos

Peptide mixtures were analysed by on-line nanoflow liquid chromatography using the nanoACQUITY-nLC system (Waters MS technologies, Manchester, UK) coupled to an LTQ-Orbitrap Velos (ThermoFisher Scientific, Bremen, Germany) mass spectrometer equipped with the manufacturer's nanospray ion source. The analytical column (nanoACQUITY UPLC^{TM} BEH130 C18 15cm x 75µm, 1.7µm capillary column) was maintained at 35°C and a flow-rate of 300nl/min. The gradient consisted of 3-40% acetonitrile in 0.1% formic acid for 90min then a ramp of 40-85% acetonitrile in 0.1% formic acid for 3 min. Full scan MS spectra (m/z range 300-2000) were acquired by the Orbitrap at a resolution of 30,000. Analysis was performed in data dependant mode. The top 20 most intense ions from MS1 scan (full MS) were selected for tandem MS by collision induced dissociation (CID) and all product spectra were acquired in the LTQ ion trap. Ion trap and orbitrap maximal injection times were set to 50ms and 500ms, respectively.

Q-Exactive MS

Digests (2 µl) were analysed on a 50cm Easy-Spray column with an internal diameter of 75µm, packed with 2µm C18 particles, fused to a silica nano-electrospray emitter (Thermo Fisher Scientific). Reversed phase liquid chromatography was performed using the Ultimate 3000 nano system with a binary buffer system consisting of 0.1% formic acid (buffer A) and 80% acetonitrile in 0.1% formic acid (buffer B). The peptides were separated by a linear gradient of 5-40% buffer B over 110 min at a flow rate of 300nl/min. The column was operated at a constant temperature of 35°C and the LC system coupled to a Q-Exactive mass spectrometer (Thermo Fisher Scientific). The Q-Exactive was operated in data-dependent mode with survey scans acquired at a resolution of 70,000 at m/z 200. Up to the top 10 most abundant isotope patterns with charge states +2, +3 and/or +4 from the survey scan were selected with an isolation window of 2.0Th and fragmented by higher energy collisional dissociation with normalized collision energies of 30. The maximum ion injection times for the survey scan and the MS/MS scans were 250 and 100ms, respectively, and the ion target value was set to 1E6 for survey scans and 1E4 for the MS/MS scans. Repetitive sequencing of peptides was minimized through dynamic exclusion of the sequenced peptides for 20s.

Data analysis

Thermo RAW files were imported into Progenesis LC–MS (version 4.1, Nonlinear Dynamics). Replicate runs were time-aligned using default settings and an auto-selected run as a reference. Peaks were picked by the software using default settings and filtered to include only peaks with a charge state of between +2 and +6. Peptide intensities of replicates were normalised against the reference run by Progenesis LC-MS. Spectral data were transformed to .mgf files with Progenesis LC–MS and exported for peptide identification using the PEAKS Studio 7 (Bioinformatics Solutions Inc.) search engine. Multiple search engine platform provided by PEAKS Studio named inChorus was used, which combines searching results from PEAKS DB (Bioinformatics Solutions Inc.), Mascot (Matrix Science), OMSSA (National Center for Biotechnology Information) and X!Tandem (Global Proteome Machine Organization). Tandem MS data were searched against a custom database that contained

the common contamination and internal standards, GiardiaDB-3.1_GintestinalisAssemblageA_AnnotatedProteins or GiardiaDB-3.1_GintestinalisAssemblageB_AnnotatedProteins. The search parameters for Orbitrap-Velos were as follows; precursor mass tolerance was set to 10ppm and fragment mass tolerance was set to 0.5 Da. One missed tryptic cleavage was permitted. Carbamidomethylation was set as a fixed modification and oxidation (M) set as a variable modification. The search parameters for Q Exactive were as follows; precursor mass tolerance was set to 10ppm and fragment mass tolerance was set to 0.01 Da. One missed tryptic cleavage was permitted. Carbamidomethylation was set as a fixed modification and oxidation (M) set as a variable modification. The false discovery rates (FDR) were set at 1% and at least two unique peptides were required for reporting protein identifications. Protein abundance (iBAQ) was calculated as the sum of all the peak intensities (from Progenesis output) divided by the number of theoretically observable tryptic peptides (Schwanhausser *et al* 2011, PMID: 21593866). Protein abundance was normalised by dividing the protein iBAQ (intensity based absolute quantification) value by the summed iBAQ values for that sample. The reported abundance is the mean of the biological replicates.

~~Peptide mixtures were analysed by on-line nanoflow liquid chromatography using the nanoACQUITY-nLC system (Waters MS technologies) coupled to an LTQ-Orbitrap Velos (Thermo Fisher Scientific) mass spectrometer or an Ultimate 3000 nano system coupled to a Q-Exactive mass spectrometer (Thermo Fisher Scientific). Once samples had been run through both platforms, and peptides matched to their proteins on GiardiaDB.org at 1% FDR with a minimum of two unique peptides, datasets were then further analysed bioinformatically.~~ The mass spectrometry proteomics data have been deposited to the ProteomeXchange Consortium via the PRIDE partner repository[24] with the dataset identifier PXD004398 and 10.6019/PXD004398.

Electrophysiology.

Giardia trophozoites culture

Giardia lamblia -WB and GS strain as well as the patients' strains (obtained from 3 patients with Giardiasis from the NNUH) were grown in filter sterilized, modified TYI-S-33 medium with 10% adult bovine serum and 0.05% bovine bile [28] at 37°C in microaerophilic conditions and sub-cultured when confluent. To collect parasites for experiments, the medium was removed from the culture to eliminate unattached or dead parasites. The tube was refilled with cold, sterile medium and trophozoites detached by chilling on ice for 15 minutes.

Parasites were collected by centrifugation (1500 x g for 5 minutes at 4 °C) and washed once with the plating medium of 90% complete DMEM/10% *Giardia* medium. Parasites were then counted using a haemocytometer and diluted to the appropriate number.

To collect *Giardia* supernatant for experiments, the Giardia culture bottle was placed on ice for 15 minutes. The bottle then underwent centrifugation (1500 x g for 5 minutes at 4 °C). The supernatant was then collected and filtered 3 times using a 15mm diameter syringe

Formatted: Font: (Default) +Body (Calibri)

Formatted: Font color: Auto

Commented [JW(-S2]: Need to add reference 28 here.

Formatted: Font color: Auto

Formatted: Font color: Auto

Formatted: Font color: Auto

filters (0.2µm pore size). Subsequently the post-filtered Giardia supernatant was diluted 1:1000 and saved in -20°C freezer until required.

Formatted: Font color: Auto

Mammalian cell line (CaCo-2) preparation

CaCo-2 cells (passages 20-25) were grown in DMEM supplemented with nonessential amino acids, penicillin (12 IU/ml), streptomycin (12µg/ml), gentamycin (47 µg/ml) and 20% (vol/vol) heat inactivated fetal calf serum (all from AMIMED, Bioconcept). The cells were seeded at a density of 6 x 10^4 cells/cm^2 in 6-well Transwell filters (0.4 µm pore size) and cultured for 7-15 days until confluent. Confluent monolayers were then used for electrophysiological experiments, for co-culture experiments with *Giardia* parasites or for culture with *Giardia* supernatants.

CaCo-2 co-culture experiments with *Giardia* or *Giardia* supernatant

Confluent CaCo-2 monolayers were taken and the CaCo-2 cell media was removed and replenished with a combination of 90% complete DMEM/10% *Giardia* medium plus or minus *Giardia* trophozoites (100,000 total parasites per insert). Control cultures were maintained in a separate plate to prevent parasite contamination. Control inserts were inspected under the microscope to ensure there was no *Giardia* cross contamination. The co-cultures were incubated at 37°C and 5% CO_2 for 24 hours, after which the *Giardia* parasites were removed.

Formatted: Font color: Auto

Confluent Caco-2 monolayers were also cultured with diluted (1:1000) *Giardia* supernatants for 24 hours. Briefly, the culture media was removed from the insert and Caco-2 cell media was replaced with a combination of 99.9% complete DMEM/ 0.1% *Giardia* medium plus or minus *Giardia* supernatant.

Formatted: Font: Not Italic, Font color: Auto

Formatted: Font color: Auto

Formatted: Left

Transepithelial electrical resistance- (TEER) Assay

Monolayers of CaCo-2 cells were grown on 6-well Transwell filters (0.4 µm pore size) for 7-15 days until confluent. The development of the polarised monolayer was assessed by measuring the TEER over a 7-15 day period. Once confluent, Giardia were added to the apical side of the Transwell filter and incubated for 24 hours. The integrity of the confluent polarised monolayer was assessed by measuring the TEER before and/or after apical infection by *Giardia*.

Electrophysiology Assay

Monolayers of CaCo-2 cells on Transwell filters were mounted into a Physiological Instruments EM-CSYS-2 Ussing chamber set-up, after establishment of a confluent

monolayer and the short circuit current (I_{SC}) across the monolayer was continuously measured.

Both sides of the epithelium were bathed in 5ml of Krebs Henseleit solution that was continuously circulated through the half chambers, maintained at 37°C and continuously bubbled with 95% O_2 / 5% CO_2. The composition of the Krebs Henseleit bath solution used was similar to that used by Cuthbert [29] and had the following composition (in mM): NaCl 118, KCl 4.7, $CaCl_2$ 2.5, $MgCl_2$ 1.2, $NaHCO_3$ 25, KH_2PO_4 1.2 and glucose 11.1 (pH 7.4). The permeable supports were left for 30 mins to equilibrate before experiments were started. All filters were treated with 10µM amiloride apically to eliminate electrogenic sodium absorption through epithelial sodium channels (ENaC).Data analysis

I_{SC} was continuously monitored across the monolayers by a Physiological Instruments Multichannel Voltage/Current Clamp (VCC MC6) through 3M KCl/agar, Ag/$AgCl_2$ cartridge electrodes (Physiologic Instruments), and the raw data for I_{sc}, transepithelial resistance and transepithelial voltage were recorded using Acquire and Analyse version 1.3 software (Physiological Instruments). Data were exported to Microsoft Excel initially and then into GraphPad Prism version 5.0 for Windows package for data representation and statistical analysis.

Chemicals and Inhibitors.

Forskolin (10µM), UTP (100µM), Amiloride (10µM), and DIDS (100µM) were obtained from Sigma Aldrich, and GlyH-101 (50 µM) was obtained from Merck Chemicals. Stock solutions of Amiloride (10mM), GlyH-101 (50mM) were made by dissolving in DMSO. Final concentrations of drugs are as indicated in the text or figures and where produced by adding the appropriate volume of stock concentration to 5ml of either the basolateral or apical bathing solution.

~~Mammalian cell line preparation~~

~~CaCo-2 cells (passages 20-25) were grown in Dulbecco's Modified Eagle medium~~DMEM ~~supplemented with nonessential amino acids, penicillin (12 IU/ml), streptomycin (12µg/ml), gentamycin (47 µg/ml) and 20% (vol/vol) heat inactivated fetal calf serum (all from AMIMED, Bioconcept). The cells were seeded at a density of 6 x 10^6 cells/cm² in 6-well Transwell filters (0.4 µm pore size) and cultured for 7-15 days until confluent.~~

~~Monolayers of CaCo-2 cells were mounted into a Physiological Instruments EM-CSYS-2 Ussing chamber set-up 7-15 days after establishment of a confluent monolayer and the short circuit current (I_{SC}) across the monolayer was continuously measured.~~

~~Both sides of the epithelium were bathed in 5mls of Krebs Henseleit solution that was continuously circulated through the half chambers, maintained at 37°C and continuously bubbled with 95% O_2 / 5% CO_2. The composition of the Krebs Henseleit bath solution used was similar to that used by Cuthbert [29] and had the following composition (in mM): NaCl 118, KCl 4.7, $CaCl_2$ 2.5, $MgCl_2$ 1.2, $NaHCO_3$ 25, KH_2PO_4 1.2 and glucose 11.1 (pH 7.4). The permeable supports were left for 30 mins to equilibrate before experiments were started.~~

Electrophiology assay

I_{SC} was continuously monitored across the monolayers by a Physiological Instruments Multichannel Voltage/Current Clamp (VCC MC6) through 3M KCl/agar, Ag/$AgCl_2$ cartridge electrodes (Physiologic Instruments), and the raw data for I_{SC}, transepithelial resistance and transepithelial voltage were recorded using Acquire and Analyse version 1.3 software (Physiological Instruments). Data was exported to Microsoft Excel initially and then into GraphPad Prism version 5.0 for Windows package for data representation and statistical analysis. All filters were treated with 10µM amiloride apically to eliminate electrogenic sodium absorption through epithelial sodium channels (ENaC).

Chemicals and Inhibitors.

Formatted: Font: Not Bold

Forskolin (10µM), UTP (100µM), Amiloride (10mM), and DIDS (100µM) were obtained from Sigma Aldrich, and GlyH-101 (50 µM) and was obtained from Merck Chemicals. Stock solutions of Amiloride (10mM), GlyH-101 (50mM) were created by dissolving in DMSO. Final concentrations of drugs are as indicated in the text or figures and where produced by adding the appropriate volume of stock concentration to 5mls of either the basolateral or apical Ussing chamber.

Data analysis

I_{SC} was continuously monitored across the monolayers by a Physiological Instruments Multichannel Voltage/Current Clamp (VCC MC6) through 3M KCl/agar, Ag/$AgCl_2$ cartridge electrodes (Physiologic Instruments), and the raw data for I_{SC}, transepithelial resistance and transepithelial voltage were recorded using Acquire and Analyse version 1.3 software (Physiological Instruments). Data was exported to Microsoft Excel initially and then into GraphPad Prism version 5.0 for Windows package for data representation and statistical analysis. All filters were treated with 10µM amiloride apically to eliminate electrogenic sodium absorption through epithelial sodium channels (ENaC).

Phylogeny

To look for sequence similarities between proteins of interest from a same protein family, the coding sequences of these proteins were retrieved from *Giardia*DB (v 3.1, 4.0 and 5.0?), aligned and compared using ClustalW.

Commented [AJ3]: Which version did Maha use for her analysis?

Phylogenetic trees were built for these proteins, via Maximum likelihood approach using MEGA software (v. 6.06).

Availability of Supporting Data

All proteomic datasets are held by and can be accessed for free at the European Bioinformatics PRoteomics IDEntifications (PRIDE) database https://www.ebi.ac.uk/pride/archive/. Free Integrated functionality with other *Giardia*

large datasets hosted at EupathDB http://eupathdb.org/eupathdb/ is scheduled for the next release.

Abbreviations: ADI (Arginine Deiminase); Ambic (Ammonium bicarbonate); CaCo-2 (Human colonic adenocarcinoma derived epithelial cell line-2); DMEM (Dulbecco's Modified Eagle Medium); DIDS (4,4'-disothiocyanatostibene-2,2'-sulfonic acid); EF-1α (Elongation Factor 1-alpha); EGF (Epidermal growth factor); ENaC (Epithelial Sodium Channel); FDR (False discovery rate); FMN (Flavin mononucleotide); *GCATB* (*Giardia* cathepsin B); GlyH101; HCMP (High cysteine membrane protein); iBAQ (Intensity based absolute quantification); IEC (Intestinal Epithelial Cells); IL (Interleukine); Isc (Short-circuit current); OCT (Ornithine Carbamoyltransferase); ORF (Open reading frame); P (Pellet); PNPO (Pyridoxamine 5'-phosphate oxidase); SP (Supernatant); PRIDE (PRoteomics IDEntifications); TEER (Transepithelial electrical resistance), VSP (Variant surface protein)

Abbreviations: ADI (Agirnine Deiminase); Ambic (Ammonium bicarbonate); CaCo-2 (Human colonic adenocarcinoma derived epithelial cell line-2); DMEM (Dulbecco's Modified Eagle Medium); DIDS (4,4'-disothiocyanatostibene-2,2'-sulfonic acid); EF-1α (Elongation Factor 1-alpha); EGF (Epidermal growth factor); ENaC (Epithelial Sodium Channel); FDR (False discovery rate); FMN (Flavin mononucleotide); *GCATB* (*Giardia* cathepsin B); GlyH101 (Glybenclamide); HCMP (High cysteine membrane protein); iBAQ (Intensity based absolute quantification); IEC (Intestinal Epithelial Cells); IL (Interleukine); Isc (Short-circuit current); OCT (Ornithine Carbamoyltransferase); ORF (Open reading frame); P (Pellet); PNPO (Pyridoxamine 5'-phosphate oxidase); SP (Supernatant); PRIDE (PRoteomics IDEntifications); TEER (*Trans*-epithelial electrical resistance), VSP (Variant surface protein)

Conflicts of Interest

The authors declare that they have no competing interests

Authors' Contributions

K.T., J.M.W., J.P.W and P.H. conceived and designed the studies. K. T. and A. D. co-ordinated the experiments. A.D. and S.A.N. performed the electrophysiology with J.P.W. A.D. performed the Flow Cytometry with D.S. A.D. prepared the proteomic samples. D.X. performed the proteomic experiments. A.D. and M.B. performed the phylogenetic analysis. All authors contributed to the analysis of the data sets obtained and preparation of Figures and Tables. The manuscript was drafted by A.D. and K.T. and improved and approved prior to submission by all co-authors.

Acknowledgements

The research leading to these results was primarily funded from the European Union Seventh Framework Programme ([FP7/2007-2013] [FP7/2007-2011]) under Grant agreement no: 311846. PRH is supported by the National Institute for Health Research Health Protection Research Unit (NIHR HPRU) in Gastrointestinal Infections at the University of Liverpool in partnership with Public Health England (PHE), and in collaboration with University of East Anglia, University of Oxford and the Institute of Food Research. Professor Hunter is based

at University of East Anglia. The views expressed are those of the author(s) and not necessarily those of the NHS, the NIHR, the Department of Health or Public Health England.

Formatted: Line spacing: Multiple 1.15 li

REFERENCES

1. Esch KJ and Petersen CA. Transmission and epidemiology of zoonotic protozoal diseases of companion animals. Clinical microbiology reviews. 2013;26 1:58-85. doi:10.1128/CMR.00067-12.
2. Nash TE, Herrington DA, Losonsky GA and Levine MM. Experimental human infections with Giardia lamblia. The Journal of infectious diseases. 1987;156 6:974-84.
3. Cotton JA, Bhargava A, Ferraz JG, Yates RM, Beck PL and Buret AG. Giardia duodenalis cathepsin B proteases degrade intestinal epithelial interleukin-8 and attenuate interleukin-8-induced neutrophil chemotaxis. Infect Immun. 2014; doi:10.1128/IAI.01771-14.
4. Turk V, Stoka V, Vasiljeva O, Renko M, Sun T, Turk B, et al. Cysteine cathepsins: from structure, function and regulation to new frontiers. Biochim Biophys Acta. 2012;1824 1:68-88. doi:10.1016/j.bbapap.2011.10.002.
5. Musil D, Zucic D, Turk D, Engh RA, Mayr I, Huber R, et al. The refined 2.15 A X-ray crystal structure of human liver cathepsin B: the structural basis for its specificity. EMBO J. 1991;10 9:2321-30.
6. DuBois KN, Abodeely M, Sakanari J, Craik CS, Lee M, McKerrow JH, et al. Identification of the major cysteine protease of Giardia and its role in encystation. J Biol Chem. 2008;283 26:18024-31. doi:M802133200 [pii]

10.1074/jbc.M802133200.

7. Sajid M and McKerrow JH. Cysteine proteases of parasitic organisms. Molecular and biochemical parasitology. 2002;120 1:1-21. doi:S0166685101004388 [pii].
8. Rodriguez-Fuentes GB, Cedillo-Rivera R, Fonseca-Linan R, Arguello-Garcia R, Munoz O, Ortega-Pierres G, et al. Giardia duodenalis: analysis of secreted proteases upon trophozoite-epitheial cell interaction in vitro. Mem Inst Oswaldo Cruz. 2006;101 6:693-6.
9. Paget TA and James SL. The mucolytic activity of polyamines and mucosal invasion. Biochem Soc Trans. 1994;22 4:394S.
10. Wampfler PB, Tosevski V, Nanni P, Spycher C and Hehl AB. Proteomics of Secretory and Endocytic Organelles in Giardia lamblia. PLoS One. 2014;9 4:e94089. doi:10.1371/journal.pone.0094089.
11. Faso C, Bischof S and Hehl AB. The proteome landscape of Giardia lamblia encystation. PLoS One. 2013;8 12:e83207. doi:10.1371/journal.pone.0083207.
12. Lingdan L, Pengtao G, Wenchao L, Jianhua L, Ju Y, Chengwu L, et al. Differential dissolved protein expression throughout the life cycle of Giardia lamblia. Exp Parasitol. 2012;132 4:465-9. doi:10.1016/j.exppara.2012.09.014.
13. Ringqvist E, Palm JE, Skarin H, Hehl AB, Weiland M, Davids BJ, et al. Release of metabolic enzymes by Giardia in response to interaction with intestinal epithelial cells. Molecular and biochemical parasitology. 2008;159 2:85-91. doi:S0166-6851(08)00056-X [pii]

10.1016/j.molbiopara.2008.02.005.

14. Roxstrom-Lindquist K, Palm D, Reiner D, Ringqvist E and Svard SG. Giardia immunity--an update. Trends Parasitol. 2006;22 1:26-31. doi:S1471-4922(05)00311-9 [pii]

10.1016/j.pt.2005.11.005.

15. Emery SJ, Mirzaei M, Vuong D, Pascovici D, Chick JM, Lacey E, et al. Induction of virulence factors in Giardia duodenalis independent of host attachment. Scientific reports. 2016;6:20765. doi:10.1038/srep20765.

16. Schwanhausser B, Busse D, Li N, Dittmar G, Schuchhardt J, Wolf J, et al. Global quantification of mammalian gene expression control. Nature. 2011;473 7347:337-42. doi:10.1038/nature10098.

17. Emery SJ, Lacey E and Haynes PA. Data from a proteomic baseline study of Assemblage A in Giardia duodenalis. Data in brief. 2015;5:23-7. doi:10.1016/j.dib.2015.08.003.

18. Emery SJ, Lacey E and Haynes PA. Quantitative proteomic analysis of Giardia duodenalis assemblage A: A baseline for host, assemblage, and isolate variation. Proteomics. 2015;15 13:2281-5. doi:10.1002/pmic.201400434.

19. Jerlstrom-Hultqvist J, Ankarklev J and Svard SG. Is human giardiasis caused by two different Giardia species? Gut Microbes. 2010;1 6:379-82. doi:10.4161/gmic.1.6.13608.

20. Skarin H, Ringqvist E, Hellman U and Svard SG. Elongation factor 1-alpha is released into the culture medium during growth of Giardia intestinalis trophozoites. Exp Parasitol. 2011;127 4:804-10. doi:S0014-4894(11)00017-8 [pii]

10.1016/j.exppara.2011.01.006.

21. Condeelis J. Elongation factor 1 alpha, translation and the cytoskeleton. Trends Biochem Sci. 1995;20 5:169-70. doi:S0968000400889987 [pii].

22. Nandan D and Reiner NE. Leishmania donovani engages in regulatory interference by targeting macrophage protein tyrosine phosphatase SHP-1. Clinical immunology. 2005;114 3:266-77. doi:10.1016/j.clim.2004.07.017.

23. Tan S, Tompkins LS and Amieva MR. Helicobacter pylori usurps cell polarity to turn the cell surface into a replicative niche. PLoS pathogens. 2009;5 5:e1000407. doi:10.1371/journal.ppat.1000407.

24. Davids BJ, Reiner DS, Birkeland SR, Preheim SP, Cipriano MJ, McArthur AG, et al. A new family of giardial cysteine-rich non-VSP protein genes and a novel cyst protein. PLoS One. 2006;1:e44. doi:10.1371/journal.pone.0000044.

25. Nash TE. Surface antigenic variation in Giardia lamblia. Mol Microbiol. 2002;45 3:585-90. doi:3029 [pii].

26. Requejo R, Hurd TR, Costa NJ and Murphy MP. Cysteine residues exposed on protein surfaces are the dominant intramitochondrial thiol and may protect against oxidative damage. The FEBS journal. 2010;277 6:1465-80. doi:10.1111/j.1742-4658.2010.07576.x.

27. Chiquet-Ehrismann R and Chiquet M. Tenascins: regulation and putative functions during pathological stress. The Journal of pathology. 2003;200 4:488-99. doi:10.1002/path.1415.

28. Keister DB. Axenic culture of Giardia lamblia in TYI-S-33 medium supplemented with bile. Trans R Soc Trop Med Hyg. 1983;77 4:487-8.

29. Cuthbert AW. Assessment of CFTR chloride channel openers in intact normal and cystic fibrosis murine epithelia. Br J Pharmacol. 2001;132 3:659-68. doi:10.1038/sj.bjp.0703859.

Figures Legends:

Table 1: The secretome of human infective *Giardia* trophozoites of assemblage A and B have a conserved repertoire of abundant secreted factors identified by both Orbitrap MS and Q-Exactive MS. 15 proteins were identified as most likely to be secreted by both GS and WB isolates. 12 are annotated proteins and 3 are hypothetical proteins. Proteins are ranked according to GS Q-Exactive Supernatant (SP) ~~expression~~ protein abundance, from most to least abundant. Of the 12 annotated proteins, 5 are tenascins and 3 are related high cysteine membrane proteins or VSP and three are ~~C~~cathepsin Bs. The other annotated abundant secreted protein is an extracellular nuclease. Protein ranking represents the proteins rank within this table, from most to least abundant. Detailed breakdown of the secretome for each assemblage by each method are provided in Supplemental tables 1-4.

Figure 1: Neighbour joining tree showing clustering of A) Cathepsin B and B) Tenascin gene families. Genes were retrieved by gene name search on *Giardia*DB. Gene sequences were downloaded and aligned using ClustalW generated with MEGA 6 software package. Maximum composite likelihood method was used, with 2000 bootstrap replicates. Bootstrap values greater than 50% are shown above the branches. ◆ proteins confirmed to be secreted using our proteomic analysis.

Figure 2: The effect of co-culture with *Giardia* or *Giardia* supernatants on the electrophysiological properties of CaCo-2 monolayers. A) Transepithelial electrical resistance (TEER) in CaCo-2 monolayers following seeding on permeable supports. Data shows increase in TEER as monolayer develops. Confluence occurred around Day 6. *Giardia* were added on Day 6 after confluent monolayer formed and co-cultured with the Caco-2 monolayer for 24 hours. TEER was measured after 24 hours and compared to TEER in monolayers that had not been exposed to *Giardia* (n=6). **B)** A representative short circuit current (Isc) against time recording from single monolayers of CaCo-2 cells in an Ussing chamber. The trace shows the activation of CFTR chloride channels (basolateral application of 10 µM Forskolin) and calcium-activated chloride channels (basolateral application of 100 µM UTP). Specificity of activation is confirmed by inhibition of Isc by the specific CFTR channel blocker, GlyH101; and specific calcium-activated chloride channel blocker, DIDS. The effect on Isc of 24 hour co-incubation of CaCo-2 monolayers with *Giardia* or with *Giardia* supernatant (1:1000 dilution) is also shown. **C)** Effect of 24 hour co-incubation of CaCo-2 monolayers with different strains of *Giardia* (WB, GS and patient samples) on forskolin-stimulated and UTP-stimulated Isc (n=3). **D)** Effect of supernatant co-incubation from different strains of *Giardia* (WB, GS and patient samples) on forskolin-stimulated and UTP-stimulated Isc (n=3) from Caco-2 monolayers. The results were analysed by student's t-test and expressed as mean values ± standard error mean (SEM). Significant difference expressed as *P<0.05, **P<0.01 compared to control.

Figure 3: Proposed novel mechanism of pathogenicity for *Giardia* involving PNPO, extracellular nuclease, *G*CATB, Tenascin. PNPO (◆) renders the intestinal environment more favourable to trophozoite's growth. Once a new *Giardia* colony is established, trophozoites release extracellular nuclease (■), GCATB (●) and Tenascin (▲). Extracellular nuclease may contribute to reducing the viscosity of the intestinal outer mucus layer, while *G*CATB may degrade mucins and disrupt intracellular junction. Finally, Tenascins may maintain intestinal cells apart by attaching to the EGF receptors present at the surface of intestinal cells which could over time lead to the apoptosis of these isolated intestinal cells.

Figure 2: The effect of *Giardia* co-culture and *Giardia* supernatants on the electrophysiological properties of CaCo-2 monolayers. A) Transepithelial electrical resistance (TEER) in CaCo-2 monolayers following seeding on permeable supports. Data shows increase in TEER as monolayer develops. Confluence occurred around Day 6. *Giardia* were added on Day 6 after confluent monolayer formed and co-cultured with the Caco-2 monolayer for 24 hours. TEER was measured after 24 hours and compared to TEER in monolayers that had not been exposed to *Giardia* (n=6). **B)** A representative short circuit current (Isc) against time recording from a single monolayer of CaCo-2 cells in an Ussing chamber. The trace shows the activation of CFTR chloride channels (basolateral application of 10 µM Forskolin) and calcium-activated chloride channels (basolateral application of 100 µM UTP). Specificity of activation is confirmed by inhibition of Isc by the specific CFTR channel blocker, GlyH101; and specific calcium activated chloride channel blocker, DIDS. The effect on Isc of incubation of CaCo-2 monolayers with *Giardia* or with *Giardia* supernatants is also shown. **C)** Effect of 24 hour co-incubation of CaCo-2 monolayers with different strains of *Giardia* (WB, GS and patient samples) on forskolin-stimulated and UTP-stimulated Isc (n=3). **D)** Effect of acute addition of supernatants from different strains of *Giardia* (WB, GS and patient samples) on forskolin-stimulated and UTP-stimulated Isc (n=3) from Caco-2 monolayers. The results were analysed by student's t-test and expressed as mean values ± standard error mean (SEM). Significant difference expressed as *P<0.05, **P<0.01 compared to control.

Figure 3: Proposed novel mechanism of pathogenicity for *Giardia* involving PNPO, extracellular nuclease, GCATB, Tenascin. PNPO ( ) renders the intestinal environment more favourable to trophozoite's growth. Once a new *Giardia* colony is established, trophozoites release extracellular nuclease ( ), GCATB ( ) and Tenascin ( ). Extracellular nuclease may contribute to reducing the viscosity of the intestinal outer mucus layer, while GCATB may degrade mucins and disrupt intracellular junction. Finally, Tenascins may maintain intestinal cells apart by attaching to the EGF receptors present at the surface of intestinal cells which could over time lead to the apoptosis of these isolated intestinal cells.

Extended data legends:

Table S1: List of the *Giardia* assemblage A (WB strain) lineage-specific proteins identified via Orbitrap and Q-Exactive MS.

Protein sequences were compared to their coding sequence and matched to their orthologs in assemblage B (GS strain) using *Giardia* database: *Giardia*DB.org. Annotated proteins are highlighted in red and hypothetical proteins in blue. Proteins were ranked according to Q-Exactive S Supernatant (S) abundance, from most to least abundant.

TableS2: List of the *Giardia* assemblage B (GS strain) lineage-specific proteins identified via Orbitrap and Q-Exactive MS.

Protein sequences were compared to their coding sequence and matched to their orthologs in assemblage A (WB strain) using *Giardia*DB.org. Annotated proteins are highlighted in red and hypothetical proteins in blue. Proteins were ranked according to Q-Exactive Supernatant (S) abundance, from most to least abundant.

Table S3: List of the 31 proteins identified via Orbitrap and Q-Exactive MS as proteins most likely secreted by *Giardia* GS strain trophozoites.

24 proteins are annotated (shown in red) and 7 are hypothetical proteins (shown in blue). 8 proteins are lineage-specific. The 15 proteins identified as conserved between the two isolates are highlighted in grey. Only proteins identified via both techniques were considered as secreted and shown in this table. Proteins are ranked according to Q-Exactive SP expression from most to least abundant.

Table S4: List of the 44 proteins identified via Orbitrap and Q-Exactive MS as proteins most likely secreted by *Giardia* WB strain trophozoites.
38 proteins are annotated (shown in red) and 6 are hypothetical proteins (shown in blue). 10 proteins are lineage-specific. The 15 proteins identified as conserved between the two isolates are highlighted in grey. Only proteins identified via both techniques were considered as secreted and shown in this table. Proteins are ranked according to Q-Exactive Supernatant (S) expression from most to least abundant.

Figure S1: ***Giardia* trophozoites are viable after incubation in non-supplemented DMEM.** Parasites were chilled on ice for 15 min, washed 3 times in pre-warmed PBS, centrifuged 10 min at 3,000 rpm between each wash; and then incubated in pre-warmed non-supplemented DMEM for 45 min. at 37°C. After 45 min incubation, parasites were chilled on ice for 5 min and centrifuged 10 min at 3,000 rpm. Pellets were collected and respuspended in PBS (A2 and B2). Trophozoites collected from culture and respuspended in either PBS (A3 and B3) or 2% trigene (detergent) (A3 and B3) were used as live and death control respectively.
Proportion of living/dead trophozoites by flow cytometry. 5 µl of propidium iodide (PI) were added in each sample to stain DNA liberated in the milieu after cell death. Flow cytometry was performed using the BD Accuri™ C6 flow cytometer, with a blue laser (λ= 488 nm) and an optical filter 585/40. Gate P2 and P3 represent alive and dead trophozoites respectively. **A.** Flow cytometry analysis for GS isolate **B.** Flow cytometry analysis for WB isolate.
Data were analysed using the BD Accuri C-flow software (version 1.0.227.4).

Formatted: Justified

Figure S2: Protein expression profile for *Giardia* assemblage A and B obtained with both MS platforms. Both GS and WB pellet (P) and supernatant (S) replicates were analysed via Orbitrap and Q-Exactive MS. Supernatant protein expression profile are similar to each other within each assemblage, so are pellet protein expression profiles (*Graph charts*). A total of 1,690 and 1,587 proteins were identified for assemblage B and A respectively (*Venn diagrams*) via both MS techniques. For assemblage A (WB isolate), 1,170 proteins were present in both dataset, 49 and 471 proteins were identified only in Orbitrap MS dataset and Q-Exactive MS datasets respectively. For assemblage B (GS isolate) 1,106 proteins were present in both datasets, 42 and 439 were identified only via Orbitrap Ms and Q-Exactive respectively for assemblage B.

Figure S3: *Giardia* proteins identified by Orbitrap and Q Exactive MS for assemblage A (WB isolate) and B (GS isolate). The Orbitrap MS analysis showed 639 and 426 proteins identified in both supernatant and pellet for assemblage B (GS isolate) and assemblage A (WB isolate) respectively, but also 51 (GS isolate) and 35 (WB isolate) in supernatant only and 461 (GS isolate) and 758 proteins (WB isolate) in pellet only respectively. The Q Exactive MS showed 946 and 490 proteins identified in both supernatant and pellet for assemblage B (GS isolate) and assemblage A (WB isolate) respectively, but also 27 (GS isolate) and 24 (WB isolate) in supernatant only and 569 (GS isolate) and 1,227 proteins (WB isolate) in pellet only respectively.
Proteins are ranked according to assemblage B Q-Exactive Supernatant (SP) expression from most to least abundant

Figure S4: Neighbour joining tree showing clustering of Tenascins in the superfamily of High Cysteine Membrane Proteins (HCMP). Tenascin genes are highlighted in yellow. Genes were retrieved by gene name search on *Giardia*DB. Gene sequences were downloaded and aligned using ClustalW generated with MEGA 6 software package. Maximum composite likelihood method was used, with 2000 bootstrap replicates. Bootstrap values greater than 50% are shown. ◆ indicates secreted proteins as confirmed by our proteomic analysis.

Proteins are ranked according to assemblage A Q-Exactive Supernatant (SP) ~~expression~~ abundance from most to least abundant

Table 1:

Protein Description	GI number Assemblage A	GI number Assemblage B	A:B Identity	dN/dS	Signal peptide[b]	P protein iBAQ	SP protein iBAQ	SP/P ratio	Abundance Ranking
PNPO	GL50803_5810	GL50581_4133	99.2	0.038	NP	5.71E+07	1.18E+08	2.063091141	1
Tenascin	GL50803_55162	GL50581_1982	76.7	**1.597**[a]	0.99	2.97E+07	4.77E+07	1.607023941	2
Tenascin	GL50803_10330	GL50581_4057	73.5	0.347	0.99	4.66E+06	2.02E+07	4.342293266	3
Cathepsin B	GL50803_16468	GL50581_438	83.6	0.1072	0.78	9.63E+06	1.79E+07	1.861308975	4
Tenascin	GL50803_8687	GL50581_4316	77.6	**44.176**[a]	0.98	6.22E+06	1.10E+07	1.770525826	5
Uncharacterized	GL50803_5258	GL50581_2767	91.2	0.029	NP[c]	3.93E+06	1.09E+07	2.780917516	6
Extracellular nuclease	GL50803_8742	GL50581_3607	83.1	0.234	1	1.03E+06	4.57E+06	4.436192622	7
Tenascin	GL50803_16477	GL50581_3575	79.8	0.1256	0.99	8.35E+05	4.03E+06	4.830955632	8
Cathepsin B	GL50803_15564	GL50581_2036	79.1	**26.5782**[a]	1	1.10E+06	3.95E+06	3.589608246	9
CKS1	GL50803_2661	GL50581_3484	100	0.001	NP[c]	1.14E+06	3.20E+06	2.803061521	10
Tenascin	GL50803_113038	GL50581_4180	79	0.0949	1	1.20E+06	3.14E+06	2.620931138	11
HCMP	GL50803_7215	GL50581_727	67	0.1821	0.99	ND[d]	2.54E+06	∞	12
Uncharacterized	GL50803_16522	GL50581_352	76	0.1591	NP[c]	1.15E+06	2.21E+06	1.928833128	13
HCMP	GL50803_12063	GL50581_2622	83	0.246	1	3.62E+05	1.94E+06	5.354665277	14
Cathepsin B	GL50803_17516	GL50581_2318	72.8	0.2056	1	ND[d]	7.81E+05	∞	15

[a] dN/dS in bold indicate proteins show evidence of positive selective pressure during divergence from a common ancestor.

[b] Probability of an N-terminal signal peptide using SignalP.

[c]NP – Not predicted.

[d]ND – Not Detected.

Protein description	GI Number Assemblage A	GI Number Assemblage B	A:B Identity	dN/dS	Signal Peptide[b]	Protein Abundance Pellet (P) iBAQ	Protein Abundance Supernatant (SP) iBAQ	SP/P Ratio	Abundance ranking
PNPO	GL50803_5810	GL50581_4133	99.2	0.038	NP[c]	5.71E+07	1.18E+08	2.063091	1
Tenascin	GL50803_95162	GL50581_1982	76.2	**1.597**[a]	0.99	2.97E+07	4.77E+07	1.607024	2
Tenascin	GL50803_10330	GL50581_4057	73.5	0.347	0.99	4.66E+06	2.02E+07	4.342293	3
Cathepsin B	GL50803_16468	GL50581_438	83.6	0.1072	0.78	9.63E+06	1.79E+07	1.861309	4
Tenascin	GL50803_8687	GL50581_4316	77.6	**44.176**[a]	0.98	6.22E+06	1.10E+07	1.770526	5
Uncharacterised	GL50803_5258	GL50581_2767	91.2	0.029	NP[c]	3.93E+06	1.09E+07	2.780918	6
Extracellular nuclease	GL50803_8742	GL50581_3607	83.1	0.234	1	1.03E+06	4.57E+06	4.436193	7
Tenascin-37	GL50803_16477	GL50581_3575	79.8	0.1256	0.99	8.35E+05	4.03E+06	4.830956	8
Cathepsin B	GL50803_15564	GL50581_2036	79.1	**26.5782**[a]	1	1.10E+06	3.95E+06	3.589608	9
CKS1	GL50803_2661	GL50581_3484	100	0.001	NP[c]	1.14E+06	3.20E+06	2.803062	10
Tenascin	GL50803_113038	GL50581_4180	79	0.0949	1	1.20E+06	3.14E+06	2.620931	11
HCMP Group 1	GL50803_7715	GL50581_727	67	0.1821	0.99	ND[d]	2.54E+06	∞	12
Uncharacterised	GL50803_16522	GL50581_352	76	0.1591	NP[c]	1.15E+06	2.21E+06	1.928833	13
HCMP	GL50803_12063	GL50581_2622	83	0.246	1	3.62E+05	1.94E+06	5.354665	14
Cathepsin B	GL50803_17516	GL50581_2318	72.8	0.2056	1	ND[d]	7.81E+05	∞	15

[a] dN/dS in bold indicate protein show evidence of positive selective pressure during divergence from a common ancestor

[b] Probability of N-terminal signal petptide using SignalP

[c] Not predicted

[d] Not detected

Manuscript

Click here to download Manuscript resubmission of giardia secretome clean .docx

Title: ***Giardia*** **Secretome Highlights Secreted Tenascins as a Key Component of Pathogenesis**

Running Title: *Giardia* Secretome

Authors: **Audrey Dubourg[a], Dong Xia[b], John P. Winpenny[a], Suha Al Naimi[a,c], Maha Bouzid[a], Darren W. Sexton[a,d], Jonathan M. Wastling[b], Paul R. Hunter[a] and Kevin M. Tyler*[a]**

Authors' affiliations:

a. NIHR Health Protection Research Unit in Gastrointestinal Infections, Norwich Medical School, University of East Anglia, Norwich, UK.

b. Department of Infection Biology, Institute of Infection and Global Health, Faculty of Health & Life Sciences, University of Liverpool, UK.

c. Department of Science and Technology, Faculty of Health and Science, University of Suffolk, Ipswich, UK

d. Liverpool Moore's University, Liverpool, UK.

Authors email:

AD: adubourg@g.ucla.edu

DX: D.Xia@Liverpool.ac.uk

JPW: john.winpenny@uea.ac.uk

SAN: s.al-naimi@ucs.ac.uk

MB: M.Bouzid@uea.ac.uk

DWS: D.W.Sexton@ljmu.ac.uk

JMW: j.wastling@keele.ac.uk

PRH: Paul.Hunter@uea.ac.uk

KT: k.tyler@uea.ac.uk

*Correspondence: k.tyler@uea.ac.uk,

Tel +44 (0)1603-591225,

Fax: +44 (0) 1603 591750.

Abstract

Background: *Giardia* is a protozoan parasite of public health relevance that causes gastroenteritis in a wide range of hosts. Two genetically distinct lineages (assemblages A and B) are responsible for the human disease. Although it is clear that differences in virulence occur, pathogenesis and virulence of *Giardia* remains poorly understood.

Findings: The genome of *Giardia* is believed to contain ORFs which could encode as many as 6,000 proteins. By successfully applying quantitative proteomic analyses to the whole parasite and to the supernatants derived from parasite culture of assemblages A and B, we confirm expression of ~1,600 proteins from each assemblage, the vast majority of which being common to both lineages. To look for signature enrichment of secreted proteins, we considered the ratio of proteins in the supernatant compared with the pellet which defined a small group of enriched proteins, putatively secreted at a steady state by cultured growing trophozoites of both assemblages. This secretome is enriched with proteins annotated to have N-terminal signal peptide. The most abundant secreted proteins include known virulence factors such as cathepsin B cysteine proteases and members of a *Giardia* superfamily of cysteine rich proteins which comprises VSPs, HCMPs and a new class of virulence factors, the *Giardia* tenascins. We demonstrate that physiological function of human enteric epithelial cells is disrupted by such soluble factors even in the absence of the trophozoites.

Conclusions: We are able to propose a straightforward model of *Giardia* pathogenesis incorporating key roles for the major *Giardia* derived soluble mediators.

Keywords: *Giardia*, Secretion, Proteomics, Quantitative Proteomics, Tenascin, Cysteine protease, Enteric Pathogen.

Background

With some 280 million symptomatic cases, giardiasis causes more bouts of human illness than any other parasitic disease [1]. The mechanism and mediators of pathogenesis by *Giardia*, however, remain largely unknown. Thanks to human volunteer studies, the association of *Giardia* infection itself and the significance of the virulence of the infecting *Giardia* strain, is experimentally unambiguous [2]. The molecular definition associated with strain virulence is though largely unexplored. It is clear that the majority of *Giardia* infections are asymptomatic. It is also clear, that infection is primarily localized to the duodenum and that some localized damage close to the sites of colonization cause villus atrophy and apoptosis of surrounding cells. However, this localized damage cannot be the sole cause of the profound diarrhoea which is often characteristic of the disease and which appears to affect absorption over a much wider area of the digestive tract than the site of infection alone.

One of the secreted mediators of damage to the duodenum is believed to be cathepsin B protease [3]. Cathepsin B-like proteases compose one of the superfamilies belonging to the CA clan of cysteine peptidases [4]. Compared to other cathepsins, cathepsin B proteases possess an additional 20 amino acid insertion named the occluding loop that enables their function as an endo- or exopeptidase [5]. Although twenty-seven genes encoding cathepsin proteases have been identified in *Giardia*, for the majority of these proteases, functions still remain elusive [6]. While some parasites may secrete cathepsin B proteases to either evade or modulate their hosts immune responses [7], a recent study has demonstrated that *Giardia* trophozoites secrete cathepsin B-like proteases, degrading intestinal IL-8 and thereby reducing the inflammation reaction by the host [3]. Secreted *Giardia* cathepsin B protease *(G*CATB*)* may also facilitate trophozoites attachment to intestinal epithelia [8] and contribute to degradation of intestinal mucin [9].

Most of the proteomic studies so far reported for *Giardia* were undertaken in trophozoites undergoing encystation [10-12]. Only a few studies have focused on proteins secreted by *Giardia* and their role in the host-pathogen interaction [3, 13-15]. These studies were focused on parasite interaction with intestinal cell lines. No studies have yet attempted to quantify proteins which are the product of steady state secretion by healthy, growing *Giardia* trophozoites and which we hypothesize as the primary mediators of giardiasis pathology. In this study, we have identified, to the limit of existing technology, the proteins expressed by populations of healthy, growing human infective *Giardia* trophozoites. We have provided quantitation of the relative abundance of retained and released trophozoite proteins from two human infective assemblages, affording calculation of the specific enrichment of released proteins and thereby the description of which proteins are most likely to be secreted by trophozoites of each assemblage. Thereafter, we compared the profile of enrichment between the two assemblages in order to identify conserved as well as assemblage-specific secreted proteins. We provide electrophysiological analysis which confirms that trophozoite secreted molecules adversely affect the homeostasis of enteric epithelia and our analysis of the heterogeneity of encoding genes between lineages demonstrates the direct selective pressure on these virulence factors and affords their use

in discriminating clinically important strains and outbreaks. Finally, the discovery of tenascins as a highly represented and variable group of proteins secreted by trophozoites strongly implicates this new class of virulence factors in a novel model for the mechanism of *Giardia* pathogenesis. We propose that their role follows degradation of the protective mucous afforded by the action of a secreted nuclease and *G*CATB, and following damage to cellular junctions by *G*CATB. Tenascins acting to prevent repair to those damaged junctions as a result of the EGF receptor ligation.

Data description

Soluble and cytosolic fractions from *in vitro* grown assemblage A and B trophozoites, the aetiologic agents of human giardiasis, were extracted in order to establish which proteins are secreted in the steady state by healthy, growing trophozoite populations. We reasoned that secreted proteins would be overrepresented in the medium in which parasites were incubated compared with the trophozoites that produced them. This ostensibly straightforward assessment being reliant on the sensitive, specific and quantitative detection of the proteins expressed by *Giardia* trophozoites in whole cells and in the medium in which the trophozoites were incubated.

The WB (assemblage A – ATCC_50803) and GS (assemblage B – ATCC_50581) reference strains were utilized to facilitate ease of comparison between genetically divergent human infective isolates with the available reference genomes. For each experiment trophozoites were harvested from mid log growth and incubated in non-supplemented Dulbecco's Modified Eagle medium (DMEM) for 45 minutes at 37°C before supernatants and pellets were collected for proteomic and other analyses including validation of their viability by flow cytometry (Additional file 1: Fig S1). Proteomic analyses were based on samples from 3 distinct biological replicates. Each sample was analysed using two quantitative proteomic platforms the Orbitrap MS and the Q-Exactive MS. Thus, in total the results from 24 (2 × 2 × 2 × 3) proteomic analyses are reported.

The identification of abundant, secreted, *Giardia* virulence factors led us to consider whether the secretions from *Giardia* alone could effect changes in the behaviour of enteric epithelia - even in the absence of the trophozoites themselves. In order to determine the effect of *Giardia* trophozoite secreted factors on the intestinal epithelia, chopstick type electrodes connected to a voltmeter were used to measure the trans-epithelial electrical resistance (TEER) of polarised CaCo-2 epithelial cells grown on permeable supports. CaCo-2 cells were cultured over 6 days until confluent. TEER across the developing CaCo-2 monolayer was measured on a daily basis as shown in Figure 2A. Once confluence was established *Giardia* trophozoites were added to the apical side of the confluent epithelium and after 24 hours incubation the trophozoites were washed from the apical surface. In order to determine whether or not co-cultures of *Giardia* trophozoites or diluted *Giardia* supernatants of both human assemblages affected ion channels responsible for secretory movement across the epithelium, an Ussing chamber system was utilised with different chloride secretion inhibitor and activators.

Further details about sample collection, secretome analysis and electrophysiology can be found in the methods section.

Analyses

Protein expression in *Giardia* trophozoites

To describe definitive *Giardia* secretomes under a standard set of conditions with high confidence and based on a robust data set and two reduce the potential for technical artifact the two MS techniques: Q-Exactive and Orbitrap MS were used with similar settings on the same three independent replicates to increase coverage. Only proteins identified by both techniques within the three replicates datasets were included in the analysis to increase the robustness of the data. The protein quantification was performed using a label-free method: iBAQ (intensity based absolute quantification) which calculates the sum of parent ion intensities of identified peptides per proteins [16]. The average normalised abundance was divided by the iBAQ values giving the "Abundance-iBAQ". The quantitative datasets from both MS techniques and for each independent replicate were shown to be strongly correlated by a Spearman correlation test (Data not shown) and therefore exploitable for proteomics analysis.

The Q-Exactive MS identified almost all of the proteins identified by use of the Orbitrap MS, and in total the two techniques identified 1,587 GS proteins and 1,690 WB proteins (Additional File 1: Fig S2). This represents over a quarter of the open reading frames (ORFs) predicted by the respective genomes in this single life-cycle stage under this steady state set of in vitro culture conditions and compares favourably with other recent proteomic analyses of *Giardia* [17, 18]. Lists of proteins detected in only one of the two assemblages are provided (Additional file 2: Table S1 and S2). Protein abundance from two of the 8 predicted assemblage-specific genes previously identified by comparative genomics was detected [19].

Overall, both assemblages gave comparable and consistent results by both platforms with the sensitivity of detection being greater for Q-Exactive MS; which provided a range of detection spanning 5 logs. In total, Q-Exactive MS identified 1,542 GS proteins and 1,641 WB proteins (Fig S3). Of these, 946 GS proteins were present in both pellet and supernatant, 27 in the supernatant only and 569 GS proteins in pellet only. By comparison 490 WB proteins were identified in supernatant and pellet and 24 in the supernatant only with 1,127 WB proteins in pellet only.

Giardia secretome

To evaluate supernatant enrichment, proteins identified in the supernatant (SP) datasets were gathered and compared to their concentration in the pellet (P) to provide a ratio using the following formula: $\frac{SP\ abundance - iBAQ}{P\ abundance - iBAQ}$. These proteins were then ranked from highest to lowest by ratiometric value and an arbitrary cut-off invoked such that the top 50 were considered as the most likely to be secreted. Proteins identified only in SP were also included in the analysis as most likely to be secreted. All the proteins selected as "of interest" were ranked according to their SP expression from most to least abundant to

obtain a quantitative enrichment profile for each isolate and this was performed for each platform. Orbitrap and Q-Exactive enrichment profiles were compared and proteins were considered as most likely to be enriched in the supernatant when identified as such by Q-Exactive MS and confirmed by Orbitrap MS. The different enrichment profiles were then also compared between assemblages.

The results showed a set of 15 orthologous proteins that were identified in both isolates by both techniques (Table 1). Eleven of these were predicted to possess an N-terminal signal sequence. Just two of these were of unknown function and two groups dominated the annotated genes encoding the rest of these proteins, five were annotated as tenascins and three as cathepsin B cysteine proteases. The most abundant enriched protein was found to be pyridoxamine 5'-phosphate oxidase (PNPO), an FMN dependent enzyme capable of fixing molecular oxygen that lacks a signal peptide and which was also recently identified as a secreted *Giardia* trophozoite protein upregulated during interaction with epithelial cells[15]. An extracellular nuclease was also present, along with a high cysteine membrane protein and a protein annotated as a VSP but which was well conserved between assemblages.

We considered that where proteins were shown to be enriched in the supernatant using both platforms and in both assemblages and possessed an N-terminal signal sequence that they were truly secreted proteins. Secreted proteins involved in adapting *Giardia* to the host environment of the human gut might be expected to be engaged in Red Queen evolution and have dN/dS indicative of positive selection. While amino-acid divergence between orthologs of secreted proteins varied considerably from 67% for the HCMP to 83% for (e.g. for the extracellular nuclease), only three proteins showed evidence of positive selections, two tenascins and one of the cathepsins. One cathepsin and one tenascin in particular showed evidence of evolution under a very high degree of selective pressure (Table 1). Although, interestingly, some cathepsins and some tenascins with similar levels of amino-acid identity between the assemblages to those under high selective pressure showed little or no evidence of positive selection.

We considered whether lineage specific soluble mediators might also be present and identified by this method. Comparing those proteins identified by both methods as having the highest relative expression in the supernatant (Tables S3 and S4). The five most abundant conserved secreted proteins from Table 1 were also present in the top 10 secreted proteins from each assemblage amongst other VSPs, tenascins, and cathepsin B, and this regardless of the MS technique or the isolate. Not unexpectedly VSPs were the primary proteins enriched in supernatants that were lineage-specific. Amongst the multigene families, however, there were also differences in the cathepsin B and tenascins/HCMP repertoires. No other proteins with N-terminal peptides were encoded in either assemblage except for one CxC-rich protein. Interestingly none of the proteins encoded by assemblage-specific genes and identified by comparative genomics were found to be enriched in the supernatants.

Giardia soluble mediators disrupt intestinal cell functions

Soluble and diffusible agents, able to disrupt gut function, could potentially mediate more diffuse and profound pathology for giardiasis than close range interactions between the trophozoites and the gastrointestinal epithelium alone. To determine whether *Giardia* secreted virulence factors could induce changes in the behaviour of intestinal epithelium, short-circuit current (Isc) was continuously measured across polarised CaCo-2 epithelial cells that had either been cultured without any additions, co-cultured with *Giardia* trophozoites or co-cultured with diluted (1:1000) *Giardia* supernatants (Figure 2B). Further experiments demonstrated that either after 24 hour co-culture with *Giardia* (Fig 2C) or 24 hour co-culture with diluted *Giardia* supernatants (Fig 2D) both experimental conditions dramatically inhibit both the cAMP-stimulated Isc (basolateral application of 10 µM Forskolin) and the calcium-activated Isc (basolateral application of 100 µM UTP). In order to identify what ion channels where being affected, the CFTR chloride ion channel inhibitor, GlyH101 (50 µM), and the calcium-activated chloride ion channel inhibitor, DIDS (100 µM), were added to the apical side of the Ussing chamber. The cAMP-stimulated Isc is predominantly due to activation of CFTR chloride channels as it is inhibited by GlyH101 (Figure 2B-D). The calcium-activated Isc is predominantly due to activation of calcium-activated chloride channels as it is inhibited by DIDS (Figure 2B-D).

Discussion

In this study, we have identified proteins secreted by trophozoites of both human infecting assemblages. Contaminating host serum proteins (mainly bovine albumin) in the supernatant samples were a concern, as previously described by others [20]. Such serum proteins bind to the parasite's surface and are continuously released which interfere with the characterisation of *Giardia* secretome. To overcome this issue, parasites were cleansed from the serum proteins and incubated in serum-free DMEM before collecting supernatants and pellets. To increase coverage and robustness of the analysis, two mass spectrometers (Orbitrap and Q-Exactive MS) were used on the same replicates and proteins identified by both MS were including in the analysis.

Previous studies have either focused on protein secretion during *Giardia* trophozoite encystation or protein secreted upon interaction with or attachment to host cells, but we have chosen here to provide a detailed baseline from cultured *Giardia* trophozoites secreting proteins under a steady state *in vitro*. Nevertheless, our results are strongly supportive of recent proteomic study looking at the effect of host attachment on the profile of giardia secreted proteins [15]. Prior to that study, several metabolic enzymes had been proposed to be released by giardia trophozoites upon interaction with intestinal epithelial cells (IEC) [13]: e.g. arginine deiminase (ADI), enolase, and ornithine carbamoyltransferase (OCT) which were all identified from culture supernatants of both assemblages.

Our study does confirm the previously observed enrichment of elongation factor 1-alpha (EF-1α), in assemblage A culture supernatants [20] (Table S4). EF-1α is a key enzyme in the protein synthesis process in eukaryotic cells [21] but many organisms have been shown to express EF-1α in excess which suggests that this protein may have some other functions [21]. In the context of pathogenicity and virulence, the secreted *Leishamnia* EF-1α was

shown to down-regulate the host inflammatory cell signalling [22]. In *H. pylori*, virulence factor CagA was shown to be translocated into the host cell and disrupt the epithelial cell polarity [23]. In *Giardia*, EF-1α has been shown to be an immunoreactive protein recognised by antibodies from patients who have previously had giardiasis [20]. Yet, its role as secreted virulence factor in *Giardia* pathogenesis remains still elusive. That this protein is only secreted by assemblage A trophozoites raises the possibility of explaining any observable differences in pathogenesis or host range between the two human infective assemblages.

The most abundant proteins, in both human isolates, primarily belong to four families of proteins: *Giardia* cathepsin B family *(*GCATB*)*, high cysteine membrane proteins (HCMPs), variant surface proteins (VSPs) and tenascins.

The cathepsin B family of *Giardia* are confirmed virulence factors involved in many of the parasite's processes such as encystation and excystation [6]; secreted GCATBs degrade host IL-8 and inhibit neutrophil chemotaxis [3]. GCATB contains secreted and non-secreted trophozoite expressed proteins; the orthologues of which are predominantly common to GS (B) and WB (A) assemblages (Fig 1). Expression of sixteen *G*CATBs was proteomically confirmed, of which eleven were shown by our proteomic analysis to be secreted. These eleven fell into six orthologous groups and for three of these groups all group members were shown to be secreted. Secreted *G*CTAB GL50803_15564 (WB) and its ortholog GL50581_2036 (GS) show a dN/dS values of >26 indicative of strong positive selective pressure. Interestingly when GS was resequenced GL50803_15564 was found to comprise three recently diverged orthologs (GSB_153537, GSB_155477, GSB_150353) and it may be that the positive selection pressure observed has been generated as a result of recent gene duplications in the assemblage B strain. GL50803_16779, an assemblage A (WB) GCATB, has previously been shown to be up-regulated and involved in trophozoite motility in early pathogenesis of *Giardia* [15]. In this study, this protein was found to be in WB top 5 secreted proteins (Table S4); its GS ortholog (GL50581_78) was also present but at a considerably lower level suggesting that for this GCATB may play a more significant role in assemblage A than assemblage B.

HCMPs are an enigmatic group of proteins with few associated functional studies. They may protect trophozoites against proteolysis [24, 25] and oxidative damage [26]. In *Giardia*, it appears that one lineage of HCMPs has given rise to the VSPs, whilst another has given rise to a group with high homology to mammalian tenascins. Tenascin, VSPs and HCMPs are then related multi-gene families which together form the largest group of proteins enriched in the *Giardia* supernatants. Interestingly, when aligned and analysed phylogenetically the secreted tenascins segregate into a monophyletic group (Figure S4). Both WB and GS orthologs of five tenascin gene products were secreted and in WB two other secreted tenascins were also detected that were not detected for the GS strain (Figure 1B).

VSPs are well-characterised surface glycoproteins with transmembrane domains, which are expressed one at a time by *Giardia* trophozoites through an RNAi regulated mechanism. They are quintessential virulence factors, responsible for antigenic variation. VSPs are

hypervariable by nature and thus it is to be expected that they do not form orthologous pairs. This was the case for most we observed, intriguingly though, a few proteins annotated as VSPs were conserved between isolates suggesting that they are not actually VSPs and would not be subject to “one at a time” control expression - but are actually misannotated HCMPs which may have a conserved function in both GS and WB isolates. This study does was not able to resolve whether the enrichment of such proteins in the supernatant observed is as a result of clipping or shedding from the parasite surface or whether the proteins are also secreted.

Tenascins are characterised by the presence of epidermal growth factor (EGF) repeats and are able to act as ligands for EGF receptors. Mammalian tenascins are extracellular matrix proteins that modulate cell adhesion and migration [27]. They appear to have evolved from a group of proteins specific to vertebrates, presumably co-evolving with the EGF receptor and so the presence of homologous proteins in *Giardia* evolving independently from HCMPs is a clear example of the kind of convergent evolution best described as molecular mimicry. Interestingly, one secreted giardia tenascin (WB-GL50803_8687/GS-GL50581_4316) identified as conserved between the two human assemblages along with another giardia tenascin (WB-GL50803_14573/GS-GL50581_1475) identified as secreted in assemblage A only (Table 1 and S4) were found to be induced by host soluble factors and implicated in regulation of trophozoites attachment [15] supporting the case for secreted tenascins acting as virulence factors in *Giardia* pathogenesis.

Most published studies concerning host cell-*Giardia* interactions have focused on the effects on the host intestinal epithelia upon attachment of the trophozoites to the cells. In this study, we have shown that diluted supernatant obtained from the steady growth of *Giardia* trophozoites in vitro has an effect on the intestinal cell function. The effect observed on chloride secretion by *Giardia* supernatants indicates that *Giardia* secretes a soluble factor which is likely affecting secretion across the intestinal epithelial cells. Physiologically, cultured intestinal cells show sensitivity to *Giardia* proteins released by the parasite even at high dilution. Fig2D demonstrates that intestinal epithelial cells when acutely exposed to such *Giardia* proteins lose the ability to stimulate CFTR and calcium-activated chloride channels. The clear implication being that virulence determinants released from *Giardia* trophozoites interact with epithelial cell receptors and ion channels.

In this analysis, we have identified the proteins which are secreted by human infective *Giardia* trophozoites. Just two groups form the majority of these proteins: *G*CATBs and the HCMP superfamily encoding known virulence factors in addition to an abundant extracellular nuclease and an oxygen fixing enzyme. The elucidation of this group of proteins dramatically increases our understanding of the pathogenic mechanisms underlying giardiasis at a molecular level. The genes encoding GCATBs and HCMP superfamily proteins are among the most heterogeneous of all genes between assemblages. Their probable role in interaction with the host and luminal environment is supported by the very high dN/dS values of some family members. Correlation of variation within assemblages at these loci with strain virulence is the essential next step for their use in diagnosis of virulent strains, risk assessment and disease prognosis.

Our results indicate that *Giardia* secretions are sufficient to disable normal function in enteric epithelial making cells less able to extract fluids from the lumen. In particular, they implicate PNPO, an extracellular nuclease, *G*CATBs and tenascins. The fact that both extracellular nuclease and *G*CATBs can be involved in the degradation of the intestinal mucus layer and that both *G*CATBs and tenascins can be associated with intestinal intracellular junction disruption suggests collaboration between these proteins. Therefore, we propose a pathogenic mechanism (Fig 3) whereby PNPO produces a reducing environment optimal for trophozoite growth, the extracellular nuclease degrades the outer layer of the intestinal mucus improving access for *G*CATBs for further degrading the protective mucous barrier and then disrupting the intestinal intracellular junctions. Lastly, tenascins are involved in maintaining intestinal cell separation by attaching to the EGF receptors present at the surface of intestinal cells and exacerbate epithelial damage by increasing the level apoptosis amongst these more detached cells. Once the intestinal barrier is breached by these mechanisms the sites of damage become prone to secondary infection by other opportunist microbes resident in the intestinal lumen and sensitive to irritation by allergens in foodstuffs leading to further inflammation and to the characteristic symptoms of the disease. Further investigations are necessary to verify this potential mechanism of pathogenesis of giardiasis.

METHODS

Proteomic Analysis.

Samples preparation

Giardia trophozoites from the genome reference strains WB (assemblage A,ATCC_50803) and GS (assemblage B, ATCC_50581), were cultured in TYI-S-33 under standard conditions (5% CO_2)[28] and harvested during the midlog phase of their *in vitro* growth curves. The total trophozoites (adhered and non-adhered) were washed 3 x in phosphate buffer saline (PBS) and then incubated in non-supplemented DMEM, with antibiotics to conserve an axenic milieu, for 45 minutes at 37°C. After incubation, an aliquot was analysed by flow cytometry to evaluate the viability of the *Giardia* samples. Trophozoites and supernatant were separated by centrifugation and both trophozoite pellet and supernatant were harvested. Proteins contained in supernatant were concentrated in Vivaspin columns (3,000 MWCO) with 25 mM ammonium bicarbonate (Ambic). Supernatants were analysed by SDS PAGE and were tested on cultured epithelial cells (Caco-2) to ensure the presence of proteins and biological activity (see below). Supernatants and pellets were sent to the Institute of Infection and Global Health at the University of Liverpool for mass spectrometry analysis (Figure S3).

Protein samples were dispensed into low protein-binding microcentrifuge tubes (Sarstedt, Leicester, UK) and made up to 160 µl by addition of 25 mM Ambic. The proteins were denatured using 10 µl of 1% (w/v) RapiGest^{TM} (Waters MS Technologies, Manchester, UK) in 25 mM Ambic followed by three cycles of freeze-thaw, and two cycles of 10 min sonication in water bath. Sample was then incubated at 80 °C for 10 min and reduced (addition of 10 µl of 60 mM DTT and incubation at 65 °C for 10 min) and alkylated (addition of 10 µl of 180 mM iodoacetamide and incubation at room temperature for 30 min in the dark). Trypsin

(Sigma-Aldrich, Dorset, UK) was reconstituted in 50 mM acetic acid to a concentration of 0.2 µg/µl. Digestion was performed by the addition of 10 µl of trypsin to the sample followed by incubation at 37 °C overnight. The RapiGest™ was removed from the sample by acidification (1 µl of trifluoroacetic acid and incubation at 37 °C for 45 min) and centrifugation (15,000 × *g* for 15 min). After protein digestion, 1 µg of digest were injected into both the Velos-Orbitrap and the Q-Exactive MS, for all samples.

Velos

Peptide mixtures were analysed by on-line nanoflow liquid chromatography using the nanoACQUITY-nLC system (Waters MS technologies, Manchester, UK) coupled to an LTQ-Orbitrap Velos (ThermoFisher Scientific, Bremen, Germany) mass spectrometer equipped with the manufacturer's nanospray ion source. The analytical column (nanoACQUITY UPLC™ BEH130 C18 15cm x 75µm, 1.7µm capillary column) was maintained at 35°C and a flow-rate of 300nl/min. The gradient consisted of 3-40% acetonitrile in 0.1% formic acid for 90min then a ramp of 40-85% acetonitrile in 0.1% formic acid for 3 min. Full scan MS spectra (m/z range 300-2000) were acquired by the Orbitrap at a resolution of 30,000. Analysis was performed in data dependant mode. The top 20 most intense ions from MS1 scan (full MS) were selected for tandem MS by collision induced dissociation (CID) and all product spectra were acquired in the LTQ ion trap. Ion trap and orbitrap maximal injection times were set to 50ms and 500ms, respectively.

Q-Exactive MS

Digests (2 µl) were analysed on a 50cm Easy-Spray column with an internal diameter of 75µm, packed with 2µm C18 particles, fused to a silica nano-electrospray emitter (Thermo Fisher Scientific). Reversed phase liquid chromatography was performed using the Ultimate 3000 nano system with a binary buffer system consisting of 0.1% formic acid (buffer A) and 80% acetonitrile in 0.1% formic acid (buffer B). The peptides were separated by a linear gradient of 5-40% buffer B over 110 min at a flow rate of 300nl/min. The column was operated at a constant temperature of 35°C and the LC system coupled to a Q-Exactive mass spectrometer (Thermo Fisher Scientific). The Q-Exactive was operated in data-dependent mode with survey scans acquired at a resolution of 70,000 at m/z 200. Up to the top 10 most abundant isotope patterns with charge states +2, +3 and/or +4 from the survey scan were selected with an isolation window of 2.0Th and fragmented by higher energy collisional dissociation with normalized collision energies of 30. The maximum ion injection times for the survey scan and the MS/MS scans were 250 and 100ms, respectively, and the ion target value was set to 1E6 for survey scans and 1E4 for the MS/MS scans. Repetitive sequencing of peptides was minimized through dynamic exclusion of the sequenced peptides for 20s.

Data analysis

Thermo RAW files were imported into Progenesis LC–MS (version 4.1, Nonlinear Dynamics). Replicate runs were time-aligned using default settings and an auto-selected run as a reference. Peaks were picked by the software using default settings and filtered to include only peaks with a charge state of between +2 and +6. Peptide intensities of replicates were

normalised against the reference run by Progenesis LC-MS. Spectral data were transformed to .mgf files with Progenesis LC–MS and exported for peptide identification using the PEAKS Studio 7 (Bioinformatics Solutions Inc.) search engine. Multiple search engine platform provided by PEAKS Studio named inChorus was used, which combines searching results from PEAKS DB (Bioinformatics Solutions Inc.), Mascot (Matrix Science), OMSSA (National Center for Biotechnology Information) and X!Tandem (Global Proteome Machine Organization). Tandem MS data were searched against a custom database that contained the common contamination and internal standards, GiardiaDB-3.1_GintestinalisAssemblageA_AnnotatedProteins or GiardiaDB-3.1_GintestinalisAssemblageB_AnnotatedProteins. The search parameters for Orbitrap-Velos were as follows; precursor mass tolerance was set to 10ppm and fragment mass tolerance was set to 0.5 Da. One missed tryptic cleavage was permitted. Carbamidomethylation was set as a fixed modification and oxidation (M) set as a variable modification. The search parameters for Q Exactive were as follows; precursor mass tolerance was set to 10ppm and fragment mass tolerance was set to 0.01 Da. One missed tryptic cleavage was permitted. Carbamidomethylation was set as a fixed modification and oxidation (M) set as a variable modification. The false discovery rates (FDR) were set at 1% and at least two unique peptides were required for reporting protein identifications. Protein abundance (iBAQ) was calculated as the sum of all the peak intensities (from Progenesis output) divided by the number of theoretically observable tryptic peptides (Schwanhausser *et al* 2011, PMID: 21593866). Protein abundance was normalised by dividing the protein iBAQ (intensity based absolute quantification) value by the summed iBAQ values for that sample. The reported abundance is the mean of the biological replicates.

The mass spectrometry proteomics data have been deposited to the ProteomeXchange Consortium via the PRIDE partner repository[24] with the dataset identifier PXD004398 and 10.6019/PXD004398.

Electrophysiology.

Giardia trophozoites culture

Giardia lamblia WB and GS strain as well as the patients' strains (obtained from 3 patients with Giardiasis from the NNUH) were grown in filter sterilized, modified TYI-S-33 medium with 10% adult bovine serum and 0.05% bovine bile [28] at 37°C in microaerophilic conditions and sub-cultured when confluent. To collect parasites for experiments, the medium was removed from the culture to eliminate unattached or dead parasites. The tube was refilled with cold, sterile medium and trophozoites detached by chilling on ice for 15 minutes.

Parasites were collected by centrifugation (1500 x g for 5 minutes at 4 °C) and washed once with the plating medium of 90% complete DMEM/10% *Giardia* medium. Parasites were then counted using a haemocytometer and diluted to the appropriate number.

To collect *Giardia* supernatant for experiments, the Giardia culture bottle was placed on ice for 15 minutes. The bottle then underwent centrifugation (1500 x g for 5 minutes at 4 °C).

The supernatant was then collected and filtered 3 times using a 15mm diameter syringe filters (0.2µm pore size). Subsequently the post-filtered Giardia supernatant was diluted 1:1000 and saved in -20°C freezer until required.

Mammalian cell line (CaCo-2) preparation

CaCo-2 cells (passages 20-25) were grown in DMEM supplemented with nonessential amino acids, penicillin (12 IU/ml), streptomycin (12µg/ml), gentamycin (47 µg/ml) and 20% (vol/vol) heat inactivated fetal calf serum (all from AMIMED, Bioconcept). The cells were seeded at a density of 6 x 10^4 cells/cm^2 in 6-well Transwell filters (0.4 µm pore size) and cultured for 7-15 days until confluent. Confluent monolayers were then used for electrophysiological experiments, for co-culture experiments with *Giardia* parasites or for culture with *Giardia* supernatants.

CaCo-2 co-culture experiments with *Giardia* or *Giardia* supernatant

Confluent CaCo-2 monolayers were taken and the CaCo-2 cell media was removed and replenished with a combination of 90% complete DMEM/10% *Giardia* medium plus or minus *Giardia* trophozoites (100,000 total parasites per insert). Control cultures were maintained in a separate plate to prevent parasite contamination. Control inserts were inspected under the microscope to ensure there was no *Giardia* cross contamination. The co-cultures were incubated at 37°C and 5% CO_2 for 24 hours, after which the *Giardia* parasites were removed.

Confluent Caco-2 monolayers were also cultured with diluted (1:1000) *Giardia* supernatants for 24 hours. Briefly, the culture media was removed from the insert and Caco-2 cell media was replaced with a combination of 99.9% complete DMEM/ 0.1% *Giardia* medium plus or minus *Giardia* supernatant.

Transepithelial electrical resistance (TEER) Assay

Monolayers of CaCo-2 cells were grown on 6-well Transwell filters (0.4 µm pore size) for 7-15 days until confluent. The development of the polarised monolayer was assessed by measuring the TEER over a 7-15 day period. Once confluent, Giardia were added to the apical side of the Transwell filter and incubated for 24 hours. The integrity of the confluent polarised monolayer was assessed by measuring the TEER before and/or after apical infection by *Giardia*.

Electrophysiology Assay

Monolayers of CaCo-2 cells on Transwell filters were mounted into a Physiological Instruments EM-CSYS-2 Ussing chamber set-up, after establishment of a confluent monolayer and the short circuit current (I_{SC}) across the monolayer was continuously measured.

Both sides of the epithelium were bathed in 5ml of Krebs Henseleit solution that was continuously circulated through the half chambers, maintained at 37°C and continuously bubbled with 95% O_2 / 5% CO_2. The composition of the Krebs Henseleit bath solution used

was similar to that used by Cuthbert [29] and had the following composition (in mM): NaCl 118, KCl 4.7, $CaCl_2$ 2.5, $MgCl_2$ 1.2, $NaHCO_3$ 25, KH_2PO_4 1.2 and glucose 11.1 (pH 7.4). The permeable supports were left for 30 mins to equilibrate before experiments were started. All filters were treated with 10µM amiloride apically to eliminate electrogenic sodium absorption through epithelial sodium channels (ENaC).Data analysis

I_{SC} was continuously monitored across the monolayers by a Physiological Instruments Multichannel Voltage/Current Clamp (VCC MC6) through 3M KCl/agar, Ag/$AgCl_2$ cartridge electrodes (Physiologic Instruments), and the raw data for I_{sc}, transepithelial resistance and transepithelial voltage were recorded using Acquire and Analyse version 1.3 software (Physiological Instruments). Data were exported to Microsoft Excel initially and then into GraphPad Prism version 5.0 for Windows package for data representation and statistical analysis.

Chemicals and Inhibitors

Forskolin (10µM), UTP (100µM), Amiloride (10µM), and DIDS (100µM) were obtained from Sigma Aldrich, and GlyH-101 (50 µM) was obtained from Merck Chemicals. Stock solutions of Amiloride (10mM), GlyH-101 (50mM) were made by dissolving in DMSO. Final concentrations of drugs are as indicated in the text or figures and where produced by adding the appropriate volume of stock concentration to 5ml of either the basolateral or apical bathing solution.

Phylogeny

To look for sequence similarities between proteins of interest from a same protein family, the coding sequences of these proteins were retrieved from *Giardia*DB (v 3.1, 4.0 and 5.0), aligned and compared using ClustalW.

Phylogenetic trees were built for these proteins, via Maximum likelihood approach using MEGA software (v. 6.06).

Availability of Supporting Data

All proteomic datasets are held by and can be accessed for free at the European Bioinformatics PRoteomics IDEntifications (PRIDE) database https://www.ebi.ac.uk/pride/archive/. Free Integrated functionality with other *Giardia* large datasets hosted at EupathDB http://eupathdb.org/eupathdb/ is scheduled for the next release.

Abbreviations: ADI (Arginine Deiminase); Ambic (Ammonium bicarbonate); CaCo-2 (Human colonic adenocarcinoma derived epithelial cell line-2); DMEM (Dulbecco's Modified Eagle Medium); DIDS (4,4'-disothiocyanatostibene-2,2'-sulfonic acid); EF-1α (Elongation Factor 1-alpha); EGF (Epidermal growth factor); ENaC (Epithelial Sodium Channel); FDR (False discovery rate); FMN (Flavin mononucleotide); *G*CATB (*Giardia* cathepsin B); GlyH101; HCMP (High cysteine membrane protein); iBAQ (Intensity based absolute quantification); IEC (Intestinal Epithelial Cells); IL (Interleukine); Isc (Short-circuit current); OCT (Ornithine Carbamoyltransferase); ORF (Open reading frame); P (Pellet); PNPO (Pyridoxamine 5'-

phosphate oxidase); SP (Supernatant); PRIDE (PRoteomics IDEntifications); TEER (Transepithelial electrical resistance), VSP (Variant surface protein)

Conflicts of Interest

The authors declare that they have no competing interests

Authors' Contributions

K.T., J.M.W., J.P.W and P.H. conceived and designed the studies. K. T. and A. D. co-ordinated the experiments. A.D. and S.A.N. performed the electrophysiology with J.P.W. A.D. performed the Flow Cytometry with D.S. A.D. prepared the proteomic samples. D.X. performed the proteomic experiments. A.D. and M.B. performed the phylogenetic analysis. All authors contributed to the analysis of the data sets obtained and preparation of Figures and Tables. The manuscript was drafted by A.D. and K.T. and improved and approved prior to submission by all co-authors.

Acknowledgements

The research leading to these results was primarily funded from the European Union Seventh Framework Programme ([FP7/2007-2013] [FP7/2007-2011]) under Grant agreement no: 311846. PRH is supported by the National Institute for Health Research Health Protection Research Unit (NIHR HPRU) in Gastrointestinal Infections at the University of Liverpool in partnership with Public Health England (PHE), and in collaboration with University of East Anglia, University of Oxford and the Institute of Food Research. Professor Hunter is based at University of East Anglia. The views expressed are those of the author(s) and not necessarily those of the NHS, the NIHR, the Department of Health or Public Health England.

REFERENCES

1. Esch KJ and Petersen CA. Transmission and epidemiology of zoonotic protozoal diseases of companion animals. Clinical microbiology reviews. 2013;26 1:58-85. doi:10.1128/CMR.00067-12.
2. Nash TE, Herrington DA, Losonsky GA and Levine MM. Experimental human infections with Giardia lamblia. The Journal of infectious diseases. 1987;156 6:974-84.
3. Cotton JA, Bhargava A, Ferraz JG, Yates RM, Beck PL and Buret AG. Giardia duodenalis cathepsin B proteases degrade intestinal epithelial interleukin-8 and attenuate interleukin-8-induced neutrophil chemotaxis. Infect Immun. 2014; doi:10.1128/IAI.01771-14.
4. Turk V, Stoka V, Vasiljeva O, Renko M, Sun T, Turk B, et al. Cysteine cathepsins: from structure, function and regulation to new frontiers. Biochim Biophys Acta. 2012;1824 1:68-88. doi:10.1016/j.bbapap.2011.10.002.
5. Musil D, Zucic D, Turk D, Engh RA, Mayr I, Huber R, et al. The refined 2.15 A X-ray crystal structure of human liver cathepsin B: the structural basis for its specificity. EMBO J. 1991;10 9:2321-30.
6. DuBois KN, Abodeely M, Sakanari J, Craik CS, Lee M, McKerrow JH, et al. Identification of the major cysteine protease of Giardia and its role in encystation. J Biol Chem. 2008;283 26:18024-31. doi:M802133200 [pii]

10.1074/jbc.M802133200.

7. Sajid M and McKerrow JH. Cysteine proteases of parasitic organisms. Molecular and biochemical parasitology. 2002;120 1:1-21. doi:S0166685101004388 [pii].
8. Rodriguez-Fuentes GB, Cedillo-Rivera R, Fonseca-Linan R, Arguello-Garcia R, Munoz O, Ortega-Pierres G, et al. Giardia duodenalis: analysis of secreted proteases upon trophozoite-epitheial cell interaction in vitro. Mem Inst Oswaldo Cruz. 2006;101 6:693-6.
9. Paget TA and James SL. The mucolytic activity of polyamines and mucosal invasion. Biochem Soc Trans. 1994;22 4:394S.
10. Wampfler PB, Tosevski V, Nanni P, Spycher C and Hehl AB. Proteomics of Secretory and Endocytic Organelles in Giardia lamblia. PLoS One. 2014;9 4:e94089. doi:10.1371/journal.pone.0094089.
11. Faso C, Bischof S and Hehl AB. The proteome landscape of Giardia lamblia encystation. PLoS One. 2013;8 12:e83207. doi:10.1371/journal.pone.0083207.
12. Lingdan L, Pengtao G, Wenchao L, Jianhua L, Ju Y, Chengwu L, et al. Differential dissolved protein expression throughout the life cycle of Giardia lamblia. Exp Parasitol. 2012;132 4:465-9. doi:10.1016/j.exppara.2012.09.014.
13. Ringqvist E, Palm JE, Skarin H, Hehl AB, Weiland M, Davids BJ, et al. Release of metabolic enzymes by Giardia in response to interaction with intestinal epithelial cells. Molecular and biochemical parasitology. 2008;159 2:85-91. doi:S0166-6851(08)00056-X [pii]

10.1016/j.molbiopara.2008.02.005.
14. Roxstrom-Lindquist K, Palm D, Reiner D, Ringqvist E and Svard SG. Giardia immunity--an update. Trends Parasitol. 2006;22 1:26-31. doi:S1471-4922(05)00311-9 [pii]

10.1016/j.pt.2005.11.005.
15. Emery SJ, Mirzaei M, Vuong D, Pascovici D, Chick JM, Lacey E, et al. Induction of virulence factors in Giardia duodenalis independent of host attachment. Scientific reports. 2016;6:20765. doi:10.1038/srep20765.
16. Schwanhausser B, Busse D, Li N, Dittmar G, Schuchhardt J, Wolf J, et al. Global quantification of mammalian gene expression control. Nature. 2011;473 7347:337-42. doi:10.1038/nature10098.
17. Emery SJ, Lacey E and Haynes PA. Data from a proteomic baseline study of Assemblage A in Giardia duodenalis. Data in brief. 2015;5:23-7. doi:10.1016/j.dib.2015.08.003.
18. Emery SJ, Lacey E and Haynes PA. Quantitative proteomic analysis of Giardia duodenalis assemblage A: A baseline for host, assemblage, and isolate variation. Proteomics. 2015;15 13:2281-5. doi:10.1002/pmic.201400434.
19. Jerlstrom-Hultqvist J, Ankarklev J and Svard SG. Is human giardiasis caused by two different Giardia species? Gut Microbes. 2010;1 6:379-82. doi:10.4161/gmic.1.6.13608.
20. Skarin H, Ringqvist E, Hellman U and Svard SG. Elongation factor 1-alpha is released into the culture medium during growth of Giardia intestinalis trophozoites. Exp Parasitol. 2011;127 4:804-10. doi:S0014-4894(11)00017-8 [pii]

10.1016/j.exppara.2011.01.006.
21. Condeelis J. Elongation factor 1 alpha, translation and the cytoskeleton. Trends Biochem Sci. 1995;20 5:169-70. doi:S0968000400889987 [pii].
22. Nandan D and Reiner NE. Leishmania donovani engages in regulatory interference by targeting macrophage protein tyrosine phosphatase SHP-1. Clinical immunology. 2005;114 3:266-77. doi:10.1016/j.clim.2004.07.017.

23. Tan S, Tompkins LS and Amieva MR. Helicobacter pylori usurps cell polarity to turn the cell surface into a replicative niche. PLoS pathogens. 2009;5 5:e1000407. doi:10.1371/journal.ppat.1000407.

24. Davids BJ, Reiner DS, Birkeland SR, Preheim SP, Cipriano MJ, McArthur AG, et al. A new family of giardial cysteine-rich non-VSP protein genes and a novel cyst protein. PLoS One. 2006;1:e44. doi:10.1371/journal.pone.0000044.

25. Nash TE. Surface antigenic variation in Giardia lamblia. Mol Microbiol. 2002;45 3:585-90. doi:3029 [pii].

26. Requejo R, Hurd TR, Costa NJ and Murphy MP. Cysteine residues exposed on protein surfaces are the dominant intramitochondrial thiol and may protect against oxidative damage. The FEBS journal. 2010;277 6:1465-80. doi:10.1111/j.1742-4658.2010.07576.x.

27. Chiquet-Ehrismann R and Chiquet M. Tenascins: regulation and putative functions during pathological stress. The Journal of pathology. 2003;200 4:488-99. doi:10.1002/path.1415.

28. Keister DB. Axenic culture of Giardia lamblia in TYI-S-33 medium supplemented with bile. Trans R Soc Trop Med Hyg. 1983;77 4:487-8.

29. Cuthbert AW. Assessment of CFTR chloride channel openers in intact normal and cystic fibrosis murine epithelia. Br J Pharmacol. 2001;132 3:659-68. doi:10.1038/sj.bjp.0703859.

Figures Legends:

Table 1: The secretome of human infective *Giardia* trophozoites of assemblage A and B have a conserved repertoire of abundant secreted factors identified by both Orbitrap MS and Q-Exactive MS. 15 proteins were identified as most likely to be secreted by both GS and WB isolates. 12 are annotated proteins and 3 are hypothetical proteins. Proteins are ranked according to GS Q-Exactive Supernatant (SP) protein abundance, from most to least abundant. Of the 12 annotated proteins, 5 are tenascins and 3 are related high cysteine membrane proteins or VSP and three are cathepsin Bs. The other annotated abundant secreted protein is an extracellular nuclease. Protein ranking represents the proteins rank within this table, from most to least abundant. Detailed breakdown of the secretome for each assemblage by each method are provided in Supplemental tables 1-4.

Figure 1: Neighbour joining tree showing clustering of A) Cathepsin B and B) Tenascin gene families. Genes were retrieved by gene name search on *Giardia*DB. Gene sequences were downloaded and aligned using ClustalW generated with MEGA 6 software package. Maximum composite likelihood method was used, with 2000 bootstrap replicates. Bootstrap values greater than 50% are shown above the branches. ◆ proteins confirmed to be secreted using our proteomic analysis.

Figure 2: The effect of co-culture with *Giardia* or *Giardia* supernatants on the electrophysiological properties of CaCo-2 monolayers. A) Transepithelial electrical resistance (TEER) in CaCo-2 monolayers following seeding on permeable supports. Data shows increase in TEER as monolayer develops. Confluence occurred around Day 6. *Giardia* were added on Day 6 after confluent monolayer formed and co-cultured with the Caco-2 monolayer for 24 hours. TEER was measured after 24 hours and compared to TEER in monolayers that had not been exposed to *Giardia* (n=6). **B)** A representative short circuit current (Isc) against time recording from single monolayers of CaCo-2 cells in an Ussing chamber. The trace shows the activation of CFTR chloride channels (basolateral application of 10 µM Forskolin) and calcium-activated chloride channels (basolateral application of 100 µM UTP). Specificity of activation is confirmed by inhibition of Isc by the specific CFTR channel blocker, GlyH101; and specific calcium-activated chloride channel blocker, DIDS. The effect on Isc of 24 hour co-incubation of CaCo-2 monolayers with *Giardia* or with *Giardia* supernatant (1:1000 dilution) is also shown. **C)** Effect of 24 hour co-incubation of CaCo-2 monolayers with different strains of *Giardia* (WB, GS and patient samples) on forskolin-stimulated and UTP-stimulated Isc (n=3). **D)** Effect of supernatant co-incubation from different strains of *Giardia* (WB, GS and patient samples) on forskolin-stimulated and UTP-stimulated Isc (n=3) from Caco-2 monolayers. The results were analysed by student's t-test and expressed as mean values ± standard error mean (SEM). Significant difference expressed as *P<0.05, **P<0.01 compared to control.

Figure 3: Proposed novel mechanism of pathogenicity for *Giardia* involving PNPO, extracellular nuclease, *G*CATB, Tenascin. PNPO (◆) renders the intestinal environment more favourable to trophozoite's growth. Once a new *Giardia* colony is established, trophozoites release extracellular nuclease (▢), GCATB (⬭) and Tenascin (▲). Extracellular nuclease may contribute to reducing the viscosity of the intestinal outer mucus layer, while *G*CATB may degrade mucins and disrupt intracellular junction. Finally, Tenascins may maintain intestinal cells apart by attaching to the EGF receptors present at the surface of intestinal cells which could over time lead to the apoptosis of these isolated intestinal cells.

Extended data legends:

Table S1: List of the *Giardia* assemblage A (WB strain) lineage-specific proteins identified via Orbitrap and Q-Exactive MS.
Protein sequences were compared to their coding sequence and matched to their orthologs in assemblage B (GS strain) using *Giardia* database: *Giardia*DB.org. Annotated proteins are highlighted in red and hypothetical proteins in blue. Proteins were ranked according to Q-Exactive S Supernatant (S) abundance, from most to least abundant.

TableS2: List of the *Giardia* assemblage B (GS strain) lineage-specific proteins identified via Orbitrap and Q-Exactive MS.
Protein sequences were compared to their coding sequence and matched to their orthologs in assemblage A (WB strain) using *Giardia*DB.org. Annotated proteins are highlighted in red and hypothetical proteins in blue. Proteins were ranked according to Q-Exactive Supernatant (S) abundance, from most to least abundant.

Table S3: List of the 31 proteins identified via Orbitrap and Q-Exactive MS as proteins most likely secreted by *Giardia* GS strain trophozoites.
24 proteins are annotated (shown in red) and 7 are hypothetical proteins (shown in blue). 8 proteins are lineage-specific. The 15 proteins identified as conserved between the two isolates are highlighted in grey. Only proteins identified via both techniques were considered as secreted and shown in this table. Proteins are ranked according to Q-Exactive SP expression from most to least abundant.

Table S4: List of the 44 proteins identified via Orbitrap and Q-Exactive MS as proteins most likely secreted by *Giardia* WB strain trophozoites.
38 proteins are annotated (shown in red) and 6 are hypothetical proteins (shown in blue). 10 proteins are lineage-specific. The 15 proteins identified as conserved between the two isolates are highlighted in grey. Only proteins identified via both techniques were considered as secreted and shown in this table. Proteins are ranked according to Q-Exactive Supernatant (S) expression from most to least abundant.

Figure S1: ***Giardia* trophozoites are viable after incubation in non-supplemented DMEM.** Parasites were chilled on ice for 15 min, washed 3 times in pre-warmed PBS, centrifuged 10 min at 3,000 rpm between each wash; and then incubated in pre-warmed non-supplemented DMEM for 45 min. at 37°C. After 45 min incubation, parasites were chilled on ice for 5 min and centrifuged 10 min at 3,000 rpm. Pellets were collected and respuspended in PBS (A2 and B2). Trophozoites collected from culture and respuspended in either PBS (A3 and B3) or 2% trigene (detergent) (A3 and B3) were used as live and death control respectively.
Proportion of living/dead trophozoites by flow cytometry. 5 µl of propidium iodide (PI) were added in each sample to stain DNA liberated in the milieu after cell death. Flow cytometry was performed using the BD Accuri™ C6 flow cytometer, with a blue laser (λ= 488 nm) and an optical filter 585/40. Gate P2 and P3 represent alive and dead trophozoites respectively. **A.** Flow cytometry analysis for GS isolate **B.** Flow cytometry analysis for WB isolate.
Data were analysed using the BD Accuri C-flow software (version 1.0.227.4).

Figure S2: Protein expression profile for *Giardia* assemblage A and B obtained with both MS platforms. Both GS and WB pellet (P) and supernatant (S) replicates were analysed via Orbitrap and Q-Exactive MS. Supernatant protein expression profile are similar to each other within each assemblage, so are pellet protein expression profiles (*Graph charts*). A total of 1,690 and 1,587

proteins were identified for assemblage B and A respectively (*Venn diagrams*) via both MS techniques. For assemblage A (WB isolate), 1,170 proteins were present in both dataset, 49 and 471 proteins were identified only in Orbitrap MS dataset and Q-Exactive MS datasets respectively. For assemblage B (GS isolate) 1,106 proteins were present in both datasets, 42 and 439 were identified only via Orbitrap Ms and Q-Exactive respectively for assemblage B.

Figure S3: *Giardia* proteins identified by Orbitrap and Q Exactive MS for assemblage A (WB isolate) and B (GS isolate). The Orbitrap MS analysis showed 639 and 426 proteins identified in both supernatant and pellet for assemblage B (GS isolate) and assemblage A (WB isolate) respectively, but also 51 (GS isolate) and 35 (WB isolate) in supernatant only and 461 (GS isolate) and 758 proteins (WB isolate) in pellet only respectively. The Q Exactive MS showed 946 and 490 proteins identified in both supernatant and pellet for assemblage B (GS isolate) and assemblage A (WB isolate) respectively, but also 27 (GS isolate) and 24 (WB isolate) in supernatant only and 569 (GS isolate) and 1,227 proteins (WB isolate) in pellet only respectively.
Proteins are ranked according to assemblage B Q-Exactive Supernatant (SP) expression from most to least abundant

Figure S4: Neighbour joining tree showing clustering of Tenascins in the superfamily of High Cysteine Membrane Proteins (HCMP). Tenascin genes are highlighted in yellow. Genes were retrieved by gene name search on *Giardia*DB. Gene sequences were downloaded and aligned using ClustalW generated with MEGA 6 software package. Maximum composite likelihood method was used, with 2000 bootstrap replicates. Bootstrap values greater than 50% are shown. ◆ indicates secreted proteins as confirmed by our proteomic analysis.
Proteins are ranked according to assemblage A Q-Exactive Supernatant (SP) abundance from most to least abundant

Table 1:

Protein description	GI Number Assemblage A	GI Number Assemblage B	A:B Identity	dN/dS	Signal Peptide[b]	Protein Abundance: Pellet (P) iBAQ	Protein Abundance: Supernatant (SP) iBAQ	SP/P Ratio	Abundance ranking
PNPO	GL50803_5810	GL50581_4133	99.2	0.038	NP[c]	5.71E+07	1.18E+08	2.063091	1
Tenascin	GL50803_95162	GL50581_1982	76.2	**1.597**[a]	0.99	2.97E+07	4.77E+07	1.607024	2
Tenascin	GL50803_10330	GL50581_4057	73.5	0.347	0.99	4.66E+06	2.02E+07	4.342293	3
Cathepsin B	GL50803_16468	GL50581_438	83.6	0.1072	0.78	9.63E+06	1.79E+07	1.861309	4
Tenascin	GL50803_8687	GL50581_4316	77.6	**44.176**[a]	0.98	6.22E+06	1.10E+07	1.770526	5
Uncharacterised	GL50803_5258	GL50581_2767	91.2	0.029	NP[c]	3.93E+06	1.09E+07	2.780918	6
Extracellular nuclease	GL50803_8742	GL50581_3607	83.1	0.234	1	1.03E+06	4.57E+06	4.436193	7
Tenascin-37	GL50803_16477	GL50581_3575	79.8	0.1256	0.99	8.35E+05	4.03E+06	4.830956	8
Cathepsin B	GL50803_15564	GL50581_2036	79.1	**26.5782**[a]	1	1.10E+06	3.95E+06	3.589608	9
CKS1	GL50803_2661	GL50581_3484	100	0.001	NP[c]	1.14E+06	3.20E+06	2.803062	10
Tenascin	GL50803_113038	GL50581_4180	79	0.0949	1	1.20E+06	3.14E+06	2.620931	11
HCMP Group 1	GL50803_7715	GL50581_727	67	0.1821	0.99	ND[d]	2.54E+06	∞	12
Uncharacterised	GL50803_16522	GL50581_352	76	0.1591	NP[c]	1.15E+06	2.21E+06	1.928833	13
HCMP	GL50803_12063	GL50581_2622	83	0.246	1	3.62E+05	1.94E+06	5.354665	14
Cathepsin B	GL50803_17516	GL50581_2318	72.8	0.2056	1	ND[d]	7.81E+05	∞	15

[a] dN/dS in bold indicate protein show evidence of positive selective pressure during divergence from a common ancestor

[b] Probability of N-terminal signal petptide using SignalP

[c] Not predicted

[d] Not detected

Figure 1 Click here to download Figure Figure 1.tif

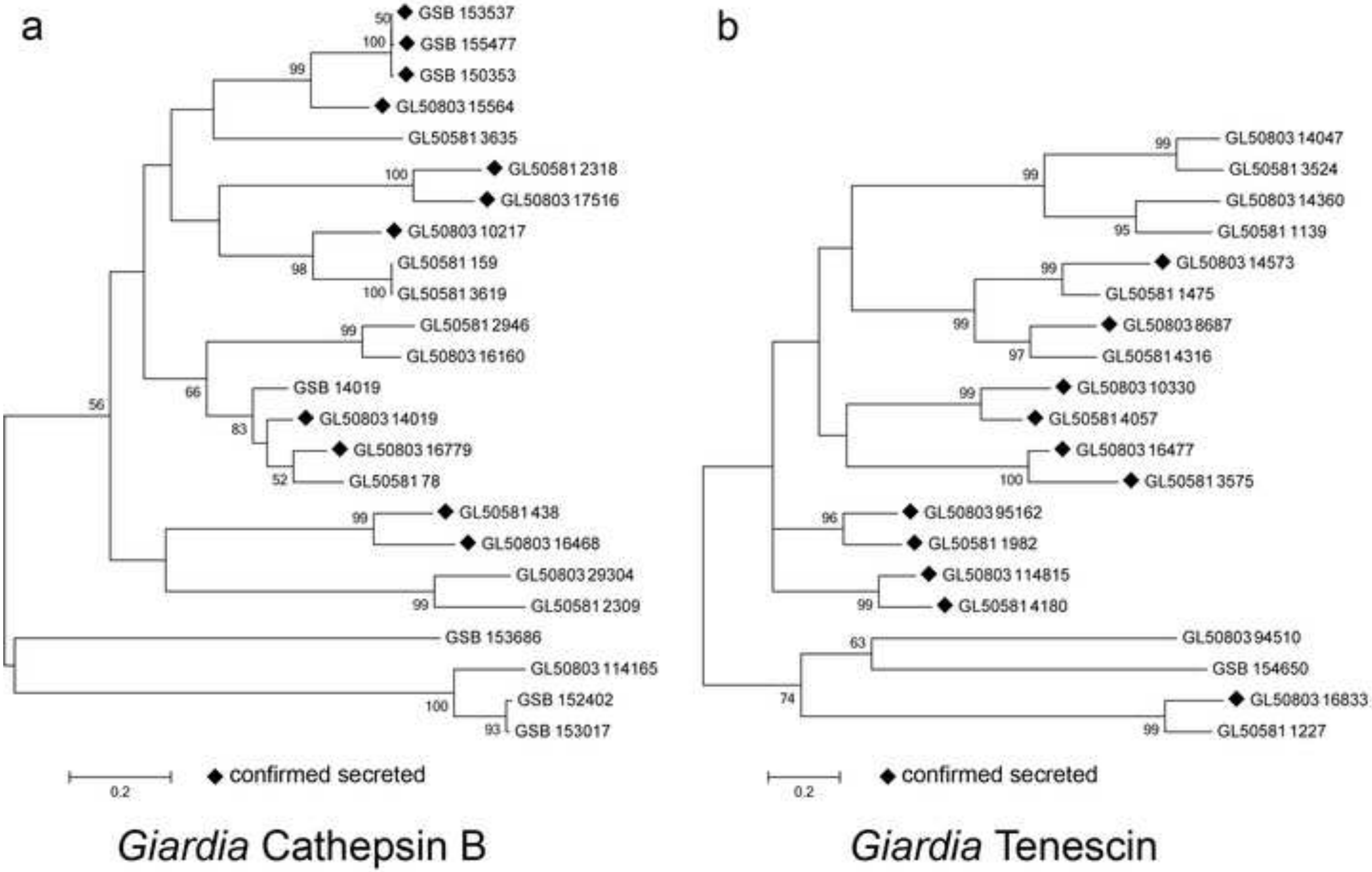

Figure 2

Click here to download Figure Figure 2.tif

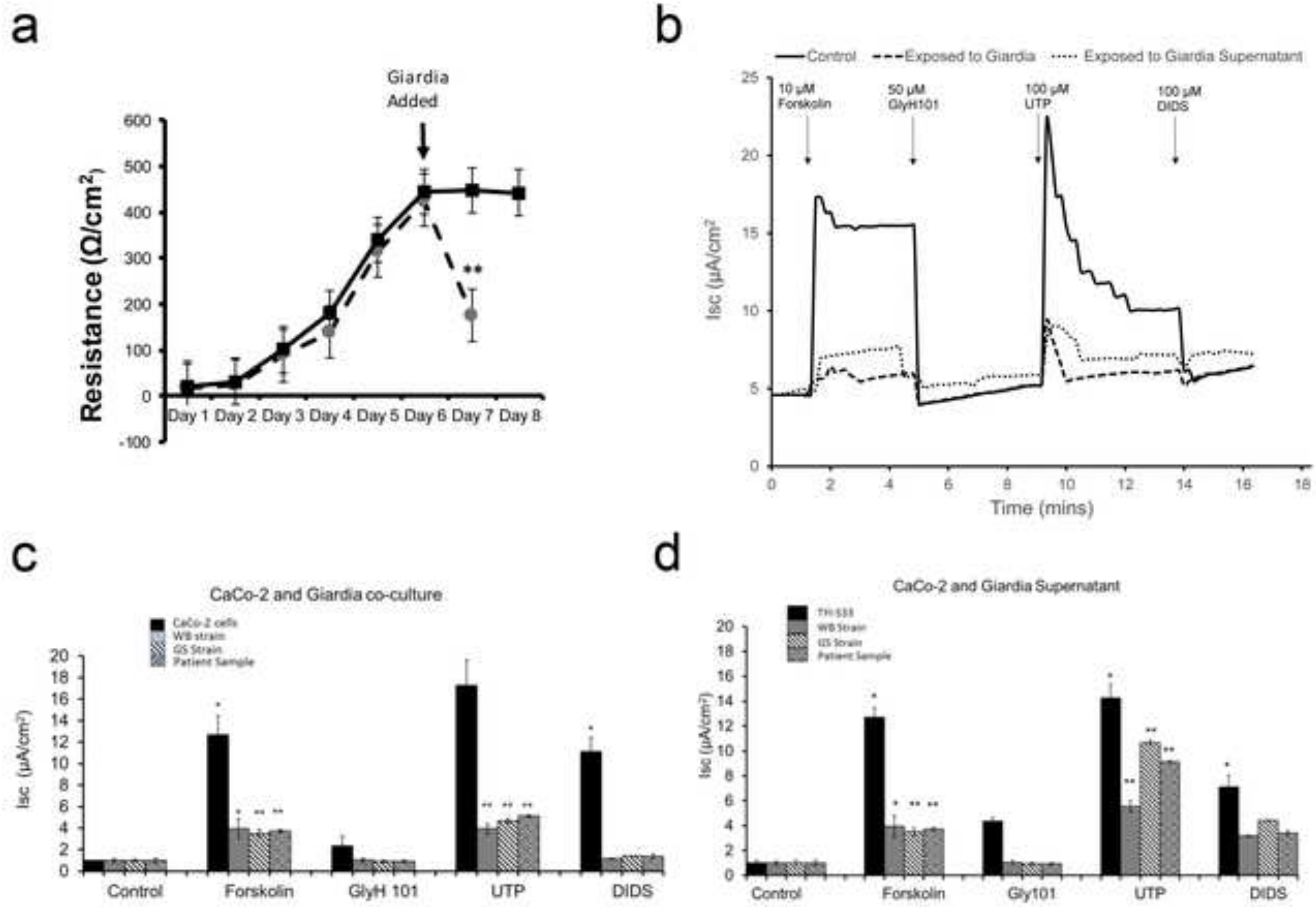

 Click here to download Figure Figure 3.tif

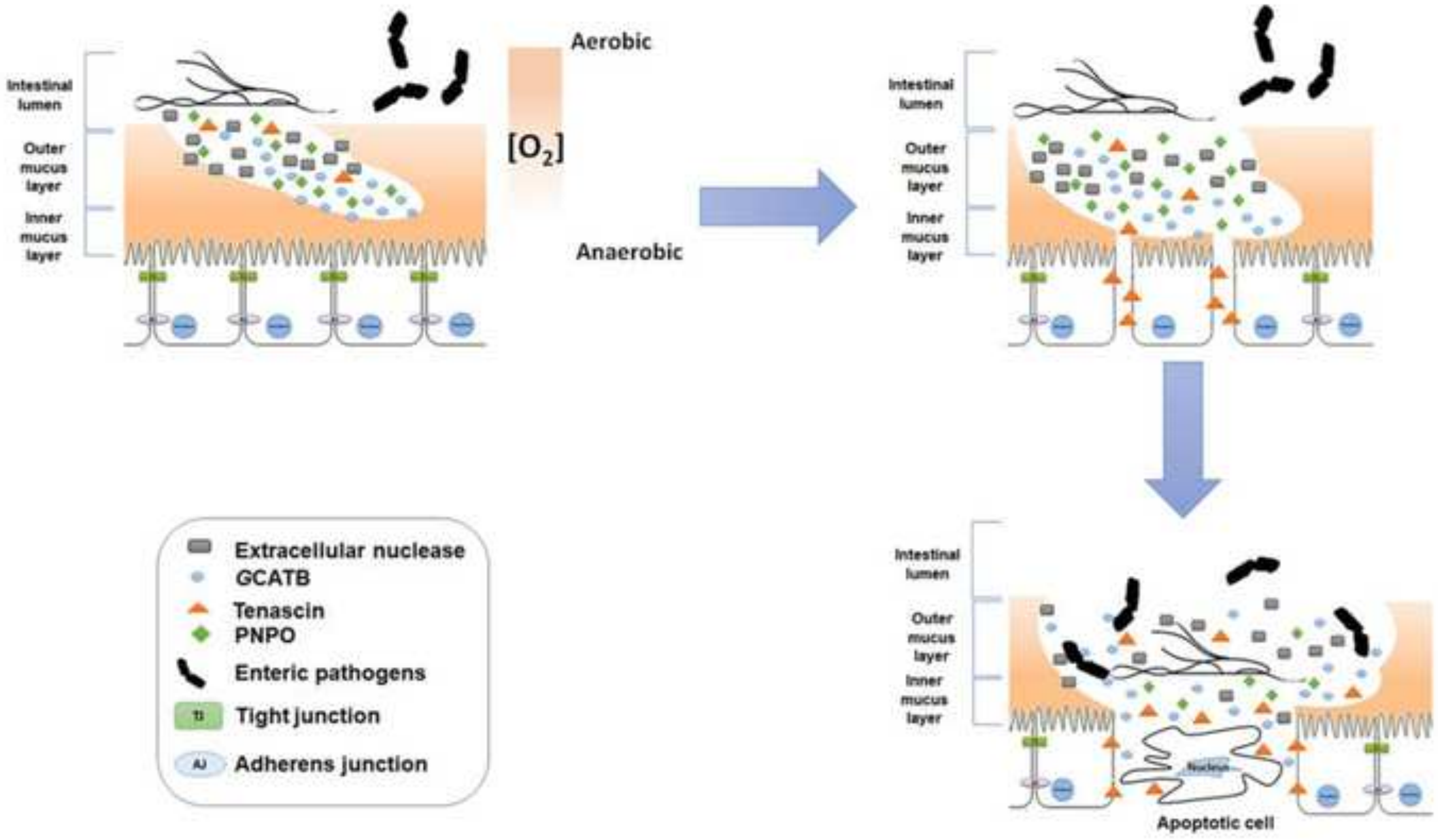

Mechanism of Pathogenesis

Figure S1

Click here to access/download
Supplementary Material
Additional Figure S1.pdf

Figure S2

Click here to access/download
Supplementary Material
Additional File Figure S2.pdf

Figure S3

Click here to access/download
Supplementary Material
Additional File Figure S3.pdf

Figure S4

Click here to access/download
Supplementary Material
Additional File Figure S4.pdf

Table S1

Click here to access/download

Supplementary Material

Additional file Table S1 Amended.docx

Table S2

Click here to access/download
Supplementary Material
Additional Table S2 amended.docx

Table S3

Click here to access/download
Supplementary Material
Additional file Table S3 amended.docx

Table S4

Click here to access/download
Supplementary Material
Additional file Table S4 amended.docx

 Click here to download Personal Cover Improvements from peer review.docx

Responses to the Reviewers and Editor's comments Regarding: "*Giardia* Secretome Highlights Secreted Tenascins as a Key Component of Pathogenesis", GIGA-D-17-00132

Summary:

Thank you to you and your reviewers for a set of incisive comments and thorough suggestions on the manuscript. We have incorporated as far as it was possible for us to do so and as a result, we hope that you will find the paper much improved in general. The methods section in particular has been significantly improved and detailed. Fig 1 and 3 are now improved and of higher resolution, all the tables (supplemental tables included) were also updated to read protein abundance instead of protein expression and typos were corrected.

Response to Reviewer#1: (Text of Reviewer with authors' responses in *italic)*

The authors have used proteomic technologies to fill in an important gap in the research regarding the secretome of *Giardia*. While this is a very important and very valuable venture, the manuscript fails to adequately report on the methods used to generate their data. The core of manuscript is a quantitative proteomic experiment, therefore it must describe the methods of acquiring proteomic data, how these data were searched and analysed quantitatively, and their consistency and reproducibility. Moreover, as this experiment involves transporting Giardia from normal optimised media to co-culture conditions and non-parasite media, more information is needed on these aspects in order to verify they were adequately controlled. I have provided some overarching comments that must be addressed before further evaluation, most of which are centralised on amending the methods so they adequately report the experiment. ***(Thank you for your suggestions, all those questions will be answered below in your comments)***

a) Proteomics:

- You have stated you have used an LTQ-Velos Orbitrap and a Q-Exactive. While these are two mass spectrometers capable of generating the high-resolution data for quantitative analyses, they aren't necessarily complementary or orthogonal platforms. What is the rationale for doing technical injections on these machines?

The aim was not to assess the technical differences between the two techniques but to have a better coverage and to increase the robustness of the data as preliminary data showed a huge discrepancy in the quantification when both MS were used on different replicates. Only the proteins identified by both techniques were included in the quantitative analysis to increase the robustness of the data. This was added in the results section to explain the need to use both MS techniques here.

The methods for the mass spectrometry, including chromatography, fragmentation regime, MS and MS2 conditions, data acquisition are missing from the methods, so it isn't possible to tell if they were run in ways to acquire different types of data. Were there differences in the way the gradient was run, or ions were acquired to justify using both?

Both MS were similarly set technically to have conserved parameters and three independent replicates were run on both MS as we only wanted to increase coverage and not compare both MS techniques. All the conditions and protocol were added into the proteomics section of the Methods to make it clear for the reader.

Were the ratios generated by each method averaged for each identification? Were they in agreement?

Ratios were generated from the averaged iBAQ abundance for each identification and this for each method. Ratios were usually in agreement, when ranking and analysing the quantitative data, only the Q-Exactive value used to rank proteins as the technique has been shown to be more sensitive.

The authors need to justify why they have used both mass spectrometers, in what way they combined the data for quantitative analyses? This needs to be discussed in the manuscript. Given that the QExactive identified nearly all the proteins in the LTQ Orbitrap runs, was there a benefit for keeping both datasets and if so this needs to be discussed.

See above for explanations, it was added in the results section to explain the benefit for using both techniques

- As stated above, the mass spectrometry methods are absent in the methods. The injection conditions, chromatography gradients, fragmentation, MS and MS2 conditions, data acquisition must be included.
As stated above, all those conditions have been added to the Methods section.

- Sample preparation is not described. Methods say the trophs and s/n were separated and proteins harvested. How were the secreted proteins harvested/precipitated from the media? Was this done at Liverpool? No conditions for protein-peptide digestion are given. More information needed.
As stated above, all those conditions have been added to the Methods section. The protocol to prepare supernatant and pellet samples prior to MS was done on site before being sent to Liverpool for the MS analysis. Protein denaturation and peptide mixture preparation as well as the MS and peptides analysis and matching were done in Liverpool. Once all quantification and protein matching were done, the datasets were sent back to UEA for further analysis of Giardia secretome.
This has now been specified in the methods sections

- Please reference the genomes used for database searching, the release number from giardiadb.org and also what search software/algorithm was used for matching peptides. Please state the search conditions, such as variable and fixed modifications, tolerance, missed cleavages etc etc. This, like the mass spec, needs to be in the manuscript. I have noticed that some of these are given in the description for the PRIDE data deposit, but these also need to be in a supplementary methods or the methods themselves.
All genome references (ATCC), release number of giardiadb.org and software/algorithm used for matching peptides as well as search conditions were added in the methods section in the proteomics sub-section, under Data analysis.

- Judging from the tables the method of quantitation is iBAQ. There is no description of this in the methods or the results. The method for generating these abundance values must be in the

methods and results. Some readers may not be familiar with iBAQ, so some explanation of it in general could be valuable to the readership.
Thank you for pointing that out, a brief explanation has been added in the results section and a more developed description in the methods section.

- As above, if you are choosing to keep both datasets from the mass spectrometers in the manuscript, then please start their consistency and agreement at the quantitative level of protein abundance as the manuscript only states their agreement at the identification level.
Prior to perform any proteome or secretome analysis, all quantitative datasets were compared by running a Spearman correlation test. This showed that the datasets were exploitable for a quantitative analysis of the secretome and proteome. However, to avoid any bias in the analysis, datasets were first analysed separately. Only the quantitative data from the replicates run on one or the other MS were merged. Secretion profiles were obtained for both techniques and then compared to see any discrepancy in the protein ranking between the two techniques. Apart from a slight variation in values (which is expected as the techniques are different and also the Q-Ex is more sensitive than the Velos-Orb) the ranking were the same regardless of the MS technique. We decided to put the protein abundance for both technique on the supplemental tables and to simplify Table 1 with only the Q-Ex values as it is more sensitive.

- The top 50 proteins were considered to be the most likely candidates as secreted proteins. What was the ratio cutoff that this included (min/max)?
The top50 cut-off included proteins with a ratio and some proteins with only an abundance value in their supernatant dataset suggesting that the latter were most likely secreted as their abundance in the proteome was potentially too low to be picked on any of the MSs. For the proteins with a ratio, the range between the top50 proteins varies greatly from one assemblage to the other. E.g.: from 0.3 up to 21 for WB isolate and from 1.5 to 10. Therefore, to simplify this analysis we decided to just focus on the top50 for now.

What were the ratios of currently known secreted proteins in Giardia, and could these be used (or at least discussed) as internal standards considered as an alternative method to filter candidates?
Since at the time of the work there was no existing gold standard study for providing "known secreted proteins" of giardia trophozoites, because many of the studies which have looked at trophozoite secretion did so in the presence of intestinal epithelial cells not from steady state supernatants and because there did not exist a consensus for which proteins are secreted and which are not we undertook the analysis "de novo" without reference to "known positive controls" which we considered might be wrong and bias any result - except insofar as we did evaluate the number of proteins predicted to have a secretion signal. Our results do however strongly support and substantiate work published in scientific reports by this reviewer in the past few years and we have now expanded the discussion with regard to known secreted proteins in Giardia highlighting this point.

- You used three biological replicates, were these used in order to filter for only reproducibly identified proteins? All the supplementary files have data at the average level? How were replicates used in the identification and quantification of proteins? You say minimum 2 peptides were needed, but what if that was found in only one of the replicates?

As the reviewer suggests we used three independent biological replicates primarily to filter for reproducibly identified proteins but also robustness of the data. If a protein was identified and quantified in only one of the three replicates, it was excluded from our final analyses.

- You need to provide a supplementary table of your proteomic identifications and quantification for your datasets of the ~1600 proteins.
We can provide these in this way, but believe that the integration of our complete dataset with EuPathDB which will be available in the next release will provide this in a far more accessible and elegant way and make any such spreadsheet appear cumbersome and redundant. The problem with accessing with the PRIDE files no double exacerbated the reviewer concern here which has now been fixed (below).

- The PRIDE deposit contains files, but there are no titles on these files? What are these? They have different numbers for accession but no titles? These are not the raw files? To what level have they been processed? Please clarify what these files are.
Thanks for looking into the PRIDE files, it appears that some of the raw files had been mixed up during the download onto the database. We contacted PRIDE IT team and got this sorted. The names of the raw files were also updated by adding the technique into the names. S stand for supernatant and P for pellet. The number 1, 2, and 3 represent the replicate number for each MS.
The Progenesis (peaks) files with only the access number refer to the Velos data as we used it as the standard technique which created files without the technique into the title.

b) Culture conditions:
- Please state throughout if you used/harvested adhered or non-adhered or total trophozoites.
Thank for you spotting that missing bit, the total trophozoites were harvested to run the experiments. This was added in both the results and methods sections.

- You say the 'reference isolates', please cite the ATCC numbers.
ATTC numbers were added in the Data description and the Methods sections

- As you are moving isolates to separate media or co-culture conditions, please state whether the trophozoites were in aerobic or anaerobic conditions, including during co-culture.
For all experiments and the standard in vitro culture of the trophozoites, parasites were under aerobic conditions with 5% CO_2. This was added into the methods section.

- There needs to be more information in the methods in regards to the experiment regarding Giardia/Giardia soluble products and the CaCO2 cells and the electrophysiology. In the Data description it says Giardia were added, in the results on page 6 is states cells were grown alone, with Giardia or with diluted supernatants. None of the information about these different conditions, their time frames or experiment design are given in the detailed methods regarding 'electrophysiology'. Not only does this need to be added, but the description of the experiment between Data description, results and methods needs to be made consistent throughout otherwise is it too confusing.

The full methodology for the culture of the Giardia parasites, the Caco-2 cells and the electrophysiological assays has now been given in the Methods section (p12-14) of the manuscript. We apologise for the confusion caused by our description of the experimental

conditions. Clarification has now been given for the different experimental conditions used for the electrophysiological assays in the Methods section (p12-14), Figure 2 legend (p18) and Analyses section (p5-7).

c) Other

- Given that VSPs are known membrane proteins, and you are looking at media fractions, have you considered the possibility they may be cleaved protein products? What sequence coverage are you getting for your VSPs, including the termini? Can you say they are indeed secreted, or could they be VSPs cleaved from the membrane during turnover?

We agree with the reviewer that this is likely, our data is consistent with at least some exterior (outward facing) plasma membrane proteins being clipped or shed from the surface rather than being actively secreted per se. We were unable to discriminate exact mechanism by which surface proteins were entering the supernatants during this study and have deliberately avoided describing them as secreted, but we are seeking funds to do exactly this using a more targeted approach and employing cell biological and transgenic as MS based methodologies.

Response to Reviewer#2: (Text of Reviewer with authors' responses in *italic*)

This manuscript revealed Giardia secretome under steady state of growth in vitro by proteomics methods and also confirmed that the secreted molecules adversely affect the homeostasis of enteric epithelia. The authors also concluded that Tenascins was a new class of virulence factors. This research provided a comprehensive proteomic data on Giardia secretome, introduced a novel pathogenicity mechanism, therefore is worthy of publication. However, there are still some problems needed to be revised before acceptance.

Thank you for your comments. We have modified the manuscript and hopefully improved it following your suggestions (see below)

1. More comparison between this research and previous Giardia proteomics research should be discussed, especially on the topic of whether the parasite-host cell interaction can induce the proteomic changes of the parasite.

   This was indeed lacking in the discussion section, thank you for pointing it out.

   Comparison between our findings and the known Giardia secreted proteins has been added in the discussion section. We have in particular discussed our findings on the metabolic proteins previously shown to be secreted upon interaction with host cells. Our data shows that these specific proteins are present in both secretome and proteome but at a very low abundance in secretome and they are therefore not in the top50 cut-off. This suggests an up-regulation of their secretion upon host-cell interaction as Svard and his team suggested. The other known to be secreted proteins were also discussed.

2. The major advantage of this research is that Tenascins was defined as a new class of virulence factor of Giardia. Considering that this is the major innovation and proposed novel mechanism of pathogenicity for Giardia, the authors should try to express them in 293T cells, insect cells or in vitro none-cell cell lysis system to analyze the pathological function of the recombinant proteins. This results will strongly support the Figure 3 novel mechanism proposal.

We agree entirely with the reviewer that just this type of in vitro experiment would indeed strongly support our findings about a novel pathogenesis mechanism. However, as much as we would love to research more and add more analysis to support our findings, we cannot do that at the moment due to lack of funding. We have included just such experiment in our current grant application along with the provision of transgenic mutants which will enable structure/function analysis.

3. Figure 1 is not necessary and can be transferred as a supplement figure. Other analysis, such as alignment of protein function domains, signalling peptide prediction also can be included in this figure. To support the proposed novel mechanism of pathogenicity, the authors should pay more attention on the protein functions, such as function domain, 2D and 3D structure, etc.

We thanks the reviewer for these ideas, we have of course begun to look closely at the tenascins which are an interesting new family of mimics as well as the cathepsins and in particular and their recent evolutionary history. We think the key findings about the protein are adequately incorporated in the figure and will aid the understanding of the general reader. Simple structural analysis does not show yield much more than we have described unfortunately – the proteins homology to actual tenascins are limited to the EGF domains where are fully intact in most cases. The proteins do appear to have emerged under selective pressure from common ancestors with HCMPs and VSP proteins and they are polyphyletic. In the future we hope that by expressing recombinant versions of the proteins we can deduce and evidence more about their structure of these proteins but this is I think well beyond the scope of this paper.

Response to Reviewer#3: (Text of Reviewer with authors' responses in *italic*)

From the biological point of view, the results are sounding and interesting. The manuscript is well presented. The choices of methods and accuracy of the procedure are globally good; the comprehensive of description of methods must be detailed. The authors definitively identify many proteins using mass spectrometry, some of which may be important for the interaction of Giardia lamblia with host cell. The manuscript is lacking any confirmation of these secreted proteins by another biological technique; however, that may not be trivial given the potential lack of antibodies.

However, I have pointed out below several concerns I have regarding this publication. The scientific content of this manuscript is interesting. It is advised to correct all the typing errors (Some corrections that I found, I indicated) and to have a last editing of the manuscript. I suggest improving the quality of the figures (as 1, 3). Please do not write protein name with uppercase (for example: replace some Cathepsin by some cathepsin).

Considering all together I can state that several points need clarification or revision before final decision.

Thank you for your comments and suggestions. We have modified the manuscript and hopefully improved it following your suggestions (see below)

ABSTRACT

Please change "N' terminus signal peptides" by "N-terminal signal peptide" and in the main text as well.

This was modified throughout the text and in the abstract.

ANALYSES
5/3: The use of expression is not appropriate here, in the main text and in the suppl. table. It is not "expression" but "protein abundance". Moreover, the term "SP expression" does not sound good to explain the more abundant proteins identified from the supernatant.

This changed throughout the text as well as in all the tables (including supplemental. tables)

The authors should explain what they used to do the ratio SP/P to compare the "abundance" of proteins. What did the value considered? Did they use the PSMs? In fact, you used iBAQ algorithm but it is not mentioned in the main text.

The iBAQ algorithm and its meaning were indeed missing from the main text, thank you for letting us know. We have added an explanation for our abundance value and iBAQ in the results section as well as the Methods section. The formula for the ratio was modified to state Sp or P abundance-iBAQ as it is what we used for the analysis. We hypothesised that proteins with a ratio ≥ 1 would be more likely secreted as enriched in the supernatant and would therefore be included in the analysis if the proteins were part of our top50 cut-off.

DISCUSSION
The authors could discuss about the high number of proteins from the supernatants; why they identified some proteins only from the supernatants since proteins are synthetized in the cell; and why they used two different mass spectrometers and they could justify the choice of these 2 spectrometers in function of the different results.

Not many proteins were identified only in the supernatant, between 10 to 20 proteins which is fairly low compared to the 1,600 proteins identified in total. However, this could be surprising and yet expected if those proteins are mainly secreted, their abundance in the cytosol would be so low compared to other proteins that it would not be picked on by the MS with the default settings, this show the limitations of the MS techniques.

We used two different MS due to preliminary data showing major discrepancy between replicates, which is why we decided to use both MS to increase coverage and robustness of the analysis. This has now been explained in the results and discussion sections.

The authors discussed the function of the more abundant secreted proteins, but did they find other proteins known secreted that could be confirm these results?

This was missing in our analysis and our discussion. Thank you for suggesting it. We have now added comparison between our dataset and the known secreted proteins in Giardia.

METHODS
it should be indicated the origin of the strains, and if the parasites are subjected or not to regular passages in animal (for example mice).

This was added into the Methods section, parasites were not passed through any animals, only in vitro in glass tubes. This was specified in the methods and results.

The Proteomic Analysis must be detailed: Cell lysis; Protein trypsin digestion; Peptide separation by reverse-phase chromatography; Mass spectra acquisition for both mass spectrometers; data analysis (.raw and protein interpretation).

The proteomics protocol was expanded and greatly improved to include all the processes in the Methods section

Did the authors work with the same number of cells from each strain? Quantity of proteins and peptides? How did the authors quantify the proteins and peptides?

As we wanted to have a protein concentration (determined by BCA assay) as high as possible and the "cleansing" of the supernatants by incubation in serum-free DMEM before collecting Pellet and supernatant samples may have caused stress onto the trophozoites, we decided to not work with a conserved number of cells but with the total trophozoites in mid-log phase growth. However, after protein digestion, 1 µg of digest was injected in each MS and this for all replicates and each strain.

9/15: should change "SDS page electrophoresis and..." by "SDS PAGE and..."

This was changed

9/21: What the cut-off of the vivaspin filter? 5000 MWCO? change Ambic by ammonium bicarbonate

The cut-off of the columns was 3000 MWCO, this was added in the manuscript. Ambic was modified as well and then the abbreviation added

9/28 and other place: should change giardiaDB.org by giardiaDB.org

Not sure what needed to be changed here if it was GiardiaDB to giardiaDB or vice-versa.

9/47 and 10/10: should change "mls" by "ml".

This has been done

10/6: Consider the following sentence: "...GlyH-101 (50mM) and" what?

We have extended and clarified the sentence to read, "Stock solutions of Amiloride (10mM), GlyH-101 (50mM) were made by dissolving in DMSO."

The sentence was corrected

10/27: should write Giardia in italic

This has been done

10/32: Abbreviations are incomplete, for example FDR

All abbreviations were added in the abbreviations section and also in the text when required

14/7: Delete S in the sentence: "…Q-Exactive S Supernatant (S) abundance, from most to least abundant…"

This has been done.
