# Supplementary material for: Giardia secretome highlights secreted tenascins as a key component of pathogenesis
Source: Gigascience. 2018 Jan 29;7(3):1–13. doi: 10.1093/gigascience/giy003 (PMC5887430; doi:10.1093/gigascience/giy003)
Supplement: GIGA-D-17-00132_Revision_2.pdf [file giy003_giga-d-17-00132_revision_2.pdf]

GigaScience

Giardia Secretome Highlights Secreted Tenascins as a Key Component of Pathogenesis

--Manuscript Draft--

**Manuscript Number:**	GIGA-D-17-00132R2
**Full Title:**	Giardia Secretome Highlights Secreted Tenascins as a Key Component of Pathogenesis
**Article Type:**	Research
**Funding Information:**	FP7 Science in Society (311846) — Prof Paul Raymond Hunter
**Abstract:**	Background: Giardia is a protozoan parasite of public health relevance that causes gastroenteritis in a wide range of hosts. Two genetically distinct lineages (assemblages A and B) are responsible for the human disease. Although it is clear that differences in virulence occur, pathogenesis and virulence of Giardia remains poorly understood.<br>Findings: The genome of Giardia is believed to contain ORFs which could encode as many as 6,000 proteins. By successfully applying quantitative proteomic analyses to the whole parasite and to the supernatants derived from parasite culture of assemblages A and B, we confirm expression of ~1,600 proteins from each assemblage, the vast majority of which being common to both lineages. To look for signature enrichment of secreted proteins, we considered the ratio of proteins in the supernatant compared with the pellet which defined a small group of enriched proteins, putatively secreted at a steady state by cultured growing trophozoites of both assemblages. This secretome is enriched with proteins annotated to have N-terminal signal peptide. The most abundant secreted proteins include known virulence factors such as cathepsin B cysteine proteases and members of a Giardia superfamily of cysteine rich proteins which comprises VSPs, HCMPs and a new class of virulence factors, the Giardia tenascins. We demonstrate that physiological function of human enteric epithelial cells is disrupted by such soluble factors even in the absence of the trophozoites.<br>Conclusions: We are able to propose a straightforward model of Giardia pathogenesis incorporating key roles for the major Giardia derived soluble mediators.
**Corresponding Author:**	Kevin Tyler<br>University of East Anglia<br>Norwich, Norfolk UNITED KINGDOM
**Corresponding Author Secondary Information:**	
**Corresponding Author's Institution:**	University of East Anglia
**Corresponding Author's Secondary Institution:**	
**First Author:**	Audrey Dubourg, PhD
**First Author Secondary Information:**	
**Order of Authors:**	Audrey Dubourg, PhD<br>Dong Xia, PhD<br>John P Winpenny, PhD<br>Suha Al-Naimi, MBChB PhD<br>Maha Bouzid, PhD<br>Darren Sexton, PhD<br>Jonathan M Wastling, PhD<br>Paul Raymond Hunter, MBChB PhD

Powered by Editorial Manager® and ProduXion Manager® from Aries Systems Corporation

	Kevin Tyler
**Order of Authors Secondary Information:**	

Response to Reviewers:

Responses to the Reviewers and Editor’s comments Regarding: “Giardia Secretome Highlights Secreted Tenascins as a Key Component of Pathogenesis”, GIGA-D-17-00132

Summary:
Thank you to you and your reviewers for these positive and constructive we have addressed them all as fully as we are able to do so.

Response to Reviewer#1: (Text of Reviewer with authors’ responses in italic)
I would like to congratulate the authors for their extensive revisions. I think this has greatly improved the manuscript and facilitated understanding of their methods. I have a few comments and revisions that I feel the authors should consider prior to final publication.
Firstly, I understand that the reviewers are using both MS platforms (Q-Exactive and LTQ Velos) to increase robustness of their data. I do not think they increase coverage, as they state they are highly correlated, and nearly all of the Velos IDs are also identified by the Q-exactive MS (which is more sensitive and makes sense). However, given there is little discrepancy between the data from both platforms, that the data is already filtered by reproducibility, and the Q-exactive is the more sensitive machine, might the authors think to keep the extra, reproducible IDs from the Q-exactive rather than discarding them totally because they are not seen on the LTQ Velos? As secreted proteins are going to be low abundance (given they are only being produced and secreted over an hour or so incubation in DMEM), the sensitivity of the Q-exactive may provide additional secreted proteins? Maybe these can at least be provided in an additional supplementary table?

Thank you for these very insightful statements and suggestions. Although we agree that the proteins identified only by Q-Exactive should not be completely excluded from the analysis in this work we would prefer to only include the proteins identified by both MS techniques to keep the robustness of our data. However, we do also agree that including the proteins identified as most likely secreted only by Q-Exactive MS may also bring additional information to the reader; therefore; those proteins were added in supplementary Table S3 and S4 in italic to differentiate them from the proteins included in the analysis.

Secondly, given the authors are comparing WB and GS, and that these most likely represent cryptic species with different clinical pathophysiology, maybe the discussion/results could be added to in order discuss differences between assemblages. The authors have already provided a robust set of proteins common between assemblages, and tables of proteins identified exclusively in WB or GS. However, significant differences between assemblages in the proteomics and/or electrophysiology could provide important insights into the differences between these two, human-infective assemblages that is limited in current literature.

This part was indeed missing, thank you for pointing it out. Not many differences in terms of protein identification were found in the dataset but a few proteins had a great variation in their abundance from one assemblage to the other. Those proteins were put in a table (Table 2) and paragraphs were added in the results section and discussion sections.

Thirdly, while the authors do plan to deposit the data in the EuPath DBs, providing excel supplementary tables of protein IDs, peptide counts and IBAQ values for reproducibly identified proteins is a very valuable resource for other researchers looking to compare identifications and expression values. Some of these people may not be familiar or aware of the EuPATH databases and their systems. I strongly advise providing these for future reviewers, as it will greatly affect the accessibility of your data.

We are happy to provide this and have added 4 supplementary tables: Table S5-S8 containing all the proteins identified peptide counts and iBAQ values for each technique and by sample< For example Table S5 represents the list of proteins

Powered by Editorial Manager® and ProduXion Manager® from Aries Systems Corporation

	identified in the pellet of GS strain trophozoites for both Q-Exactive and Orbitrap MS.<br><br>Minor Comments:<br>Methods:<br>1) In the non-supplemented DMEM, what was the oxygen levels? Were these capped, filled flasks?<br><br>Parasites were incubated for 45 min in 1 ml of non-supplemented DMEM under their standard in vitro growth conditions. Pellets were resuspended in DMEM and transferred in filtered glass tube and put at 37°C with 5% O2. To help readers and reviewers understand the protocol, a supplemental figure was created and added with the whole protocol developed and used to cleanse and harvest pellets and supernatants: Figure S5. Reference to figure was added in the Method section under Proteomic analysis, Samples preparation. In addition, culture protocols are now provided.<br><br>2) What were the centrifugation speeds - higher speeds would have been likely to rupture cells, so it might be good to report this.<br><br>The centrifugation speed to separate pellet and supernatant was the speed used to usually pellet down Giardia “safely”: 3,000 rpm. This speed is the standard speed used to pellet down the parasites before freezing cells down and is unlikely to rupture cells. However, as we incubated the, in DMEM before harvesting pellets and supernatants, we performed a flow cytometry viability assay on pellet samples after centrifugation. The parasites were still alive as shown by supplemental Figure S1. But to be sure readers have all the key elements, all the centrifugation speeds used were added onto Figure S5.<br><br>3)In the electrophysiology, what are the patient strains? Are these verified assemblage A or B? Have they been typed?<br><br>The patient strain used in the electrophysiology was not typed. It was a stabilate prepared from a clinical sample at the Norfolk and Norwich University Hospital (NNUH) and was used alongside the lab strains to control for atypical activity (e.g. attenuation) that might have occurred as an artefact of protracted culture of the laboratory strains. Since the results were similar to the laboratory strains it serves to provide support that the results are likely to be generalizable for most human giardia infections.<br><br>Results:<br>1) I think that adding some further comments about viability and harvesting contingencies to ensure cell integrity (i.e. cell not popping) would be valuable when reporting supernatant vs pellet IDs around pg 12, ln 42-49. While contamination of the supernatant with proteins is a very difficult thing to avoid in this type of experiment, hence your experimental design in averaging pellet to supernatant ratios, I think showcasing steps to mitigate contamination would add more credibility to the results.<br><br>To help the reader visualise what was done to obtain the proteomic samples, a supplementary figure: Fig S5 was created and added as well as its legend explaining every step of the protocol<br><br>2) Were the supernatants harvested with protease inhibitors at any stage, or how was protease activity controlled during harvesting? SDS-PAGE was mentioned as a quality control step (which can also show whether there is any evidence of protease activity), but pictures of these gels are not provided in the supplementary.<br><br>No protease was added in the final cleansing protocol. Several protein concentration protocols were tried before deciding to use the Vivaspin columns and we followed the protocol from the manufacturer which did not require the use of proteases. SDS-PAGE were run after the protein concentration to verify that proteins were not degraded, and BCA assay performed to obtain the protein concentration as well. A supplementary figure: Fig S6 is provided with a photo of a representative SDS-PAGE gel.
**Additional Information:**	
**Question**	**Response**

Are you submitting this manuscript to a special series or article collection?	No
**Experimental design and statistics**<br><br>Full details of the experimental design and statistical methods used should be given in the Methods section, as detailed in our Minimum Standards Reporting Checklist. Information essential to interpreting the data presented should be made available in the figure legends.<br><br>Have you included all the information requested in your manuscript?	Yes
**Resources**<br><br>A description of all resources used, including antibodies, cell lines, animals and software tools, with enough information to allow them to be uniquely identified, should be included in the Methods section. Authors are strongly encouraged to cite Research Resource Identifiers (RRIDs) for antibodies, model organisms and tools, where possible.<br><br>Have you included the information requested as detailed in our Minimum Standards Reporting Checklist?	Yes
**Availability of data and materials**<br><br>All datasets and code on which the conclusions of the paper rely must be either included in your submission or deposited in publicly available repositories (where available and ethically appropriate), referencing such data using a unique identifier in the references and in the "Availability of Data and Materials" section of your manuscript.<br><br>Have you have met the above requirement as detailed in our Minimum Standards Reporting Checklist?	Yes

Manuscript
Click here to download Manuscript secretomeresubmissionED8-1-18675clean.DOCX

Title: ***Giardia*** **Secretome Highlights Secreted Tenascins as a Key Component of Pathogenesis**

Running Title: *Giardia* Secretome

Authors: **Audrey Dubourg[a], Dong Xia[b,e], John P. Winpenny[a], Suha Al Naimi[a,c], Maha Bouzid[a], Darren W. Sexton[a,d], Jonathan M. Wastling[b,f], Paul R. Hunter[a] and Kevin M. Tyler*[a]**

Authors' affiliations:

a. NIHR Health Protection Research Unit in Gastrointestinal Infections, Norwich Medical School, University of East Anglia, Norwich, UK.

b. Department of Infection Biology, Institute of Infection and Global Health, Faculty of Health & Life Sciences, University of Liverpool, UK.

c. Department of Science and Technology, Faculty of Health and Science, University of Suffolk, Ipswich, UK

d. School of Pharmacy and Biomolecular Sciences Liverpool John Moores University, Liverpool, UK.

e. Research Support Office, Royal Veterinary College, University of London, UK.

f. Faculty of Natural Sciences, Keele University, Staffordshire, UK.

Authors email:

AD: adubourg@g.ucla.edu

DX: dxia@rvc.ac.uk

JPW: john.winpenny@uea.ac.uk

SAN: s.al-naimi@ucs.ac.uk

MB: M.Bouzid@uea.ac.uk

DWS: D.W.Sexton@ljmu.ac.uk

JMW: j.wastling@keele.ac.uk

PRH: Paul.Hunter@uea.ac.uk

KT: k.tyler@uea.ac.uk

*Correspondence: k.tyler@uea.ac.uk,

Tel +44 (0)1603-591225,

Fax: +44 (0) 1603 591750.

Abstract

Background: *Giardia* is a protozoan parasite of public health relevance that causes gastroenteritis in a wide range of hosts. Two genetically distinct lineages (assemblages A and B) are responsible for the human disease. Although it is clear that differences in virulence occur, pathogenesis and virulence of *Giardia* remains poorly understood.

Findings: The genome of *Giardia* is believed to contain ORFs that could encode as many as 6,000 proteins. By successfully applying quantitative proteomic analyses to the whole parasite and to the supernatants derived from parasite culture of assemblages A and B, we confirm expression of ~1,600 proteins from each assemblage, the vast majority of which being common to both lineages. To look for signature enrichment of secreted proteins, we considered the ratio of proteins in the supernatant compared with the pellet, which defined a small group of enriched proteins, putatively secreted at a steady state by cultured growing trophozoites of both assemblages. This secretome is enriched with proteins annotated to have N-terminal signal peptide. The most abundant secreted proteins include known virulence factors such as cathepsin B cysteine proteases and members of a *Giardia* superfamily of cysteine rich proteins that comprises VSPs, HCMPs and a new class of virulence factors, the *Giardia* tenascins. We demonstrate that physiological function of human enteric epithelial cells is disrupted by such soluble factors even in the absence of the trophozoites.

Conclusions: We are able to propose a straightforward model of *Giardia* pathogenesis incorporating key roles for the major *Giardia* derived soluble mediators.

Keywords: *Giardia*, Secretion, Proteomics, Quantitative Proteomics, Tenascin, Cysteine protease, Enteric Pathogen.

Background

With some 280 million symptomatic cases, giardiasis causes more bouts of human illness than any other parasitic disease [1]. The mechanism and mediators of pathogenesis by *Giardia*, however, remain largely unknown. Thanks to human volunteer studies, the association of *Giardia* infection itself and the significance of the virulence of the infecting *Giardia* strain, is experimentally unambiguous [2]. The molecular definition associated with strain virulence is though largely unexplored. It is clear that the majority of *Giardia* infections are asymptomatic. It is also clear, that infection is primarily localized to the duodenum and that some localized damage, close to the sites of colonization, causes villus atrophy and apoptosis of surrounding cells. However, this localized damage cannot be the sole cause of the profound diarrhoea which is often characteristic of the disease and which appears to affect absorption over a much wider area of the digestive tract than the site of infection alone.

One of the secreted mediators of damage to the duodenum is believed to be cathepsin B protease [3]. Cathepsin B-like proteases compose one of the superfamilies belonging to the CA clan of cysteine peptidases [4]. Compared to other cathepsins, cathepsin B proteases possess an additional 20 amino acid insertion named the occluding loop that enables their function as an endo- or exopeptidase [5]. Although twenty-seven genes encoding cathepsin proteases have been identified in *Giardia*, for the majority of these proteases, function remains elusive [6]. While some parasites may secrete cathepsin B proteases to either evade or modulate their hosts immune responses [7], a recent study has demonstrated that *Giardia* trophozoites secrete cathepsin B-like proteases, degrading intestinal IL-8 and thereby reducing the inflammation reaction by the host [3]. Secreted *Giardia* cathepsin B protease *(G*CATB*)* may also contribute to degradation of intestinal mucin and facilitate trophozoite attachment to intestinal epithelia [8, 9].

Most of the proteomic studies so far reported for *Giardia* were undertaken in trophozoites undergoing encystation [10-12]. Only a few studies have focused on proteins secreted by *Giardia* and their role in the host-pathogen interaction [3, 13-15]. These studies were focused on parasite interaction with intestinal cell lines. No studies have yet attempted to quantify proteins that are the product of steady state secretion by healthy, growing *Giardia* trophozoites and which we hypothesize as the primary mediators of giardiasis pathology. In this study, we have identified, to the limit of existing technology, the proteins expressed by populations of healthy, growing human infective *Giardia* trophozoites. We have provided quantitation of the relative abundance of retained and released trophozoite proteins from two human infective assemblages, affording calculation of the specific enrichment of released proteins and thereby the description of which proteins are most likely to be secreted by trophozoites of each assemblage. Thereafter, we compared the profile of enrichment between the two assemblages in order to identify conserved as well as assemblage-specific secreted proteins. We provide electrophysiological analysis which confirms that trophozoite secreted molecules adversely affect the homeostasis of enteric epithelia and our analysis of the heterogeneity of encoding genes between lineages demonstrates the direct selective pressure on these virulence factors and affords their use

in discriminating clinically important strains and outbreaks. Finally, the discovery of tenascins as a highly represented and variable group of proteins secreted by trophozoites strongly implicates this new class of virulence factors in a novel model for the mechanism of *Giardia* pathogenesis. We propose that tenescin action follows degradation of the protective mucous afforded by the action of a secreted nuclease and *G*CATB, and damage to cellular junctions by *G*CATB. Tenascins acting by means of EGF receptor ligation, to prevent repair to those damaged junctions.

Data description

Soluble and cytosolic fractions from *in vitro* grown assemblage A and B trophozoites, the aetiologic agents of human giardiasis, were extracted in order to establish which proteins are secreted in the steady state by healthy, growing trophozoite populations. We reasoned that secreted proteins would be overrepresented in the medium in which parasites were incubated compared with the trophozoites that produced them. This ostensibly straightforward assessment being reliant on the sensitive, specific and quantitative detection of the proteins expressed by *Giardia* trophozoites in whole cells and in the medium in which the trophozoites were incubated.

The WB (assemblage A – ATCC_50803) and GS (assemblage B – ATCC_50581) reference strains were utilized to facilitate ease of comparison between genetically divergent human infective isolates with the available reference genomes. For each experiment trophozoites were harvested from mid log growth and incubated in non-supplemented Dulbecco's Modified Eagle medium (DMEM) for 45 minutes at 37°C before supernatants and pellets were collected for proteomic and other analyses including validation of their viability by flow cytometry (Additional file 1: Fig S1). Proteomic analyses were based on samples from 3 distinct biological replicates. Each sample was analysed using two quantitative proteomic platforms the Orbitrap MS and the Q-Exactive MS. Thus, in total the results from 24 (2 × 2 × 2 × 3) proteomic analyses are reported.

The identification of abundant, secreted, *Giardia* virulence factors led us to consider whether the secretions from *Giardia* alone could effect changes in the behaviour of enteric epithelia - even in the absence of the trophozoites themselves. In order to determine the effect of *Giardia* trophozoite secreted factors on the intestinal epithelia, chopstick type electrodes connected to a voltmeter were used to measure the trans-epithelial electrical resistance (TEER) of polarised CaCo-2 epithelial cells grown on permeable supports. CaCo-2 cells were cultured over 6 days until confluent. TEER across the developing CaCo-2 monolayer was measured on a daily basis as shown in Figure 2A. Once confluence was established, *Giardia* trophozoites were added to the apical side of the confluent epithelium and after 24 hours incubation the trophozoites were washed from the apical surface. In order to determine whether co-cultures of *Giardia* trophozoites or diluted *Giardia* supernatants affected the ion channels responsible for secretory movement across the epithelium, an Ussing chamber system was utilised with different chloride secretion inhibitor and activators.

Further details about sample collection, secretome analysis and electrophysiology can be found in the methods section and protocols provided.

Analyses

Protein expression in *Giardia* trophozoites

To describe definitive *Giardia* secretomes under a standard set of conditions with high confidence and based on a robust data set and to reduce the potential for technical artefact the two MS techniques: Q-Exactive and Orbitrap MS were used with similar settings on the same three independent replicates to increase coverage. Only proteins identified by both techniques within the three replicates datasets were included in the analysis to increase the robustness of the data. The protein quantification was performed using a label-free method: iBAQ (intensity based absolute quantification) which calculates the sum of parent ion intensities of identified peptides per proteins [16]. The average normalised abundance was divided by the iBAQ values giving the "Abundance-iBAQ". The quantitative datasets from both MS techniques and for each independent replicate were shown to be strongly correlated by a Spearman correlation test (data not shown) and therefore exploitable for proteomic analysis.

The Q-Exactive MS identified almost all of the proteins identified by use of the Orbitrap MS, and in total the two techniques identified 1,587 GS proteins and 1,690 WB proteins (Additional File 1: Fig S2). This represents over a quarter of the open reading frames (ORFs) predicted by the respective genomes in this single life-cycle stage under this steady state set of *in vitro* culture conditions and compares favourably with other recent proteomic analyses of *Giardia* [17, 18]. Lists of proteins detected in only one of the two assemblages are provided (Additional file 2: Table S1 and S2). Protein from two of the eight predicted assemblage-specific genes previously identified by comparative genomics was detected [19].

Overall, both assemblages gave comparable and consistent results using both platforms with the sensitivity of detection being greater for Q-Exactive MS; which provided a range of detection spanning 5 logs. In total, Q-Exactive MS identified 1,542 GS proteins and 1,641 WB proteins (Fig S3). Of these, 946 GS proteins were present in both pellet and supernatant, 27 in the supernatant only and 569 GS proteins in pellet only. By comparison, 490 WB proteins were identified in supernatant and pellet and 24 in the supernatant only with 1,127 WB proteins in pellet only.

Giardia secretome

To evaluate supernatant enrichment, proteins identified in the supernatant (SP) datasets were gathered and compared to their concentration in the pellet (P) to provide a ratio using the following formula: $\frac{SP\ abundance-iBAQ}{P\ abundance-iBAQ}$. These proteins were then ranked from highest to lowest by ratiometric value and an arbitrary cut-off invoked such that the top 50 were considered as the most likely to be secreted. Proteins identified only in SP were also included in the analysis as most likely to be secreted. All the proteins selected as "of interest" were ranked according to their SP expression from most to least abundant to obtain a quantitative enrichment profile for each isolate and this was performed for each platform. Orbitrap and Q-Exactive enrichment profiles were compared and proteins were considered as most likely to be enriched in the supernatant when identified as such by Q-Exactive MS and confirmed

by Orbitrap MS. The different enrichment profiles were then also compared between assemblages.

The results yielded a set of 15 orthologous proteins that were identified in both isolates by both techniques (Table 1). Eleven of these were predicted to possess an N-terminal signal sequence. Just two of these were of unknown function and two groups dominated the annotated genes encoding the rest of these proteins, five were annotated as tenascins and three as cathepsin B cysteine proteases. The most abundant enriched protein was found to be pyridoxamine 5'-phosphate oxidase (PNPO), an FMN dependent enzyme capable of fixing molecular oxygen that lacks a signal peptide and which was also recently identified as a secreted *Giardia* trophozoite protein upregulated during interaction with epithelial cells [15]. An extracellular nuclease was also present, along with a high cysteine membrane protein; as well as a protein product of a gene misannotated as a VSP (since it was well conserved between assemblages).

We considered that where proteins were shown to be enriched in the supernatant by both platforms and in both assemblages and possessed an N-terminal signal sequence that they were truly secreted proteins. Secreted proteins involved in adapting *Giardia* to the host environment of the human gut might be expected to be engaged in Red Queen evolution and have dN/dS indicative of positive selection. While amino-acid divergence between orthologs of secreted proteins varied considerably from 67% for the HCMP to 83% for (e.g. for the extracellular nuclease), only three proteins showed evidence of positive selections, two tenascins and one of the cathepsins. One cathepsin and one tenascin in particular showed evidence of evolution under a very high degree of selective pressure (Table 1). Interestingly, some cathepsins and some tenascins with similar levels of amino-acid identity between the assemblages to those under high selective pressure showed little or no evidence of positive selection.

We considered whether lineage specific soluble mediators might also be present and identified by this method. Comparing those proteins identified by both methods as having the highest relative expression in the supernatant (Tables S3 and S4). The five most abundant conserved secreted proteins from Table 1 were also present in the top 10 secreted proteins from each assemblage amongst other VSPs, tenascins, and cathepsin B, and this regardless of the MS technique or the isolate. Unsurprisingly, VSPs were the primary proteins enriched in supernatants that were lineage-specific. Amongst the multigene families, however, there were also differences in the cathepsin B and tenascins/HCMP repertoires. No other proteins with N-terminal peptides were encoded in either assemblage except for one CxC-rich protein. Interestingly, none of the eight proteins encoded by assemblage-specific genes and identified by comparative genomics were found to be enriched in the supernatants.

When comparing secretion profiles between the two assemblages, seven proteins were over-represented in the supernatants by only one assemblage or only identified by Q-Exactive MS or present at very low abundance in one of the two (Table 2). Only two proteins, sentrin and A-type flavoprotein lateral transfer candidate, were present in the top 50 proteins of assemblage B (GS strain) trophozoites secretome. Whereas, the other five, one elongation factor 1-α (EF-1α), one ATP-binding cassette protein 5, one CxC rich protein, one

translation initiation inhibitor and a peptide methionine sulfoxide reductase MsrB, were present in the top 55 proteins of assemblage A (WB strain) trophozoites secretomes. Interestingly, A-type flavoprotein lateral transfer candidate was also present in the top50 supernatant proteins by assemblage A trophozoites; however, its low supernatant enrichment ratio (< 0.2) suggests that this protein is unlikely to be secreted by assemblage A trophozoites.

Giardia soluble mediators disrupt intestinal cell functions

Soluble and diffusible agents, able to disrupt gut function, could potentially mediate more diffuse and profound pathology for giardiasis than close range interactions between the trophozoites and the gastrointestinal epithelium alone. To determine whether *Giardia* secreted virulence factors could induce changes in the behaviour of intestinal epithelium, short-circuit current (Isc) was continuously measured across polarised CaCo-2 epithelial cells that had either been cultured without any additions, co-cultured with *Giardia* trophozoites or co-cultured with diluted (1:1000) *Giardia* supernatants (Figure 2B). Further experiments demonstrated that either after 24 hour co-culture with *Giardia* (Fig 2C) or 24 hour co-culture with diluted *Giardia* supernatants (Fig 2D) both experimental conditions dramatically inhibit both the cAMP-stimulated Isc (basolateral application of 10 µM Forskolin) and the calcium-activated Isc (basolateral application of 100 µM UTP). In order to identify what ion channels where being affected, the CFTR chloride ion channel inhibitor, GlyH101 (50 µM), and the calcium-activated chloride ion channel inhibitor, DIDS (100 µM), were added to the apical side of the Ussing chamber. The cAMP-stimulated Isc is predominantly due to activation of CFTR chloride channels as it is inhibited by GlyH101 (Figure 2B-D). The calcium-activated Isc is predominantly due to activation of calcium-activated chloride channels as it is inhibited by DIDS (Figure 2B-D).

Discussion

In this study, we have identified proteins secreted by trophozoites of both human infecting assemblages. Contaminating host serum proteins (mainly bovine albumin) in the supernatant samples were a concern, as previously described by others [20]. Such serum proteins bind to the parasite's surface and are continuously released which interfere with the characterisation of *Giardia* secretome. To overcome this issue, parasites were cleansed from the serum proteins and incubated in serum-free DMEM before collecting supernatants and pellets. To increase coverage and robustness of the analysis, two mass spectrometers (Orbitrap and Q-Exactive MS) were used on the same replicates and proteins identified by both MS were included in the analysis.

Previous studies have focused on protein secretion during *Giardia* trophozoite encystation; or protein secretion upon interaction with (or attachment to) host cells. Here instead, we chose to provide a detailed baseline from cultured *Giardia* trophozoites secreting proteins under a steady state *in vitro*. Nevertheless, our results are strongly supportive of a recent proteomic study looking at the effect of host attachment on the profile of *Giardia* secreted proteins [15]. Prior to that study, several metabolic enzymes had been proposed to be released by *Giardia* trophozoites upon interaction with intestinal epithelial cells (IEC) [13]:

such as arginine deiminase (ADI), enolase, and ornithine carbamoyltransferase (OCT) which we were also able to identify from the culture supernatants of both assemblages.

Our study does confirm the previously observed enrichment of EF-1α, in assemblage A culture supernatants [20] (Table 2 and Table S4). EF-1α is a key enzyme in the protein synthesis process in eukaryotic cells [21] but many organisms have been shown to express EF-1α in excess which suggests that this protein may have some other functions [21]. In the context of pathogenicity and virulence, the secreted *Leishamnia* EF-1α has been shown to down-regulate host inflammatory cell signalling [22]. In *Giardia*, EF-1α has been shown to be an immunoreactive protein recognised by antibodies from patients who have previously had giardiasis [20]. Yet, its role as putatively secreted virulence factor in *Giardia* pathogenesis remains elusive. That this protein is only released by assemblage A trophozoites raises the possibility of associating its function with observable differences in pathogenesis or host range between the two human infective assemblages.

Our study shows some other differences in secretions between assemblage A and B trophozoites (Table 2). A-type flavoprotein lateral transfer candidate and sentrin were present in assemblage B (GS strain) trophozoites secretome; and ATP-binding cassette (ABC) protein 5, CxC rich protein, translation initiation inhibitor and peptide methionine sulfoxide reductase (MsrB) were present in assemblage A (WB strain) secretome.

A-type flavoprotein lateral transfer candidate has a high oxygen reductase activity during *Giardia* infection suggesting an O_2 scavenging function upon release in the host intestinal environment [23]; thus, potentially affording increased resilience to *Giardia* trophozoites in the small intestine and manipulating the parasites immediate microenvironment. Whether assemblage B trophozoites require A-type flavoprotein lateral transfer candidate throughout the infection or just in its early stage remains unclear. Sentrin is involved in the ubiquitination of proteins to render them resistant to degradation [24]. Sentrin is evolutionarily conserved and has been identified in prokaryotic and eukaryotic organisms such as *S. cerevisiae*, *A. thaliana* and *Homo sapiens*, which suggests a conserved specialised function in cell metabolism [24]. With its ubiquitination function, sentrin was expected to be only present in *Giardia* proteome but not in its secretome. Why this protein would be secreted or released by *Giardia* trophozoites remains unclear and raises the question of the advantages, for the parasite, of releasing sentrin into the host environment upon infection.

ABC proteins are a large and diverse canonical group of membrane proteins typically resident in the plasma membrane and associated, in eukaryotes, with the ATP dependent egress of metabolites and toxins; they can be determinants of virulence and drug resistance [25]. Here one *Giardia* ABC protein shows enrichment in the supernatant of WB but not of GS and it will be interesting to see if a functional correlation can be found. The CxC rich protein belongs to the HCMP superfamily that also includes VSPs, tenascins and HCMPs. The presence of orthologs in both strains is consistent with it not being a VSP protein. As with several other HCMPs, this CxC rich protein had a very high signal and only one TM domain suggesting that it may be a labile surface protein in WB, but its specific role and why it is much more abundant in the WB supernatant than the GS supernatant is not clear. Translation initiation inhibitors are proteins inhibiting the initiation of the translation of messenger RNA (mRNA)

into proteins and are mainly located in the cell cytosol [26]. Yet, one translation initiation inhibitor is over-represented in the assemblage A trophozoite secretome (Top20 secreted proteins) probably due to its high solubility and stability. Peptide methionine sulfoxide reductase (MsrB) catalyses the reduction of free- and protein-bound methionine sulfoxides to corresponding methionines, which constitutes a mechanism for the scavenging of reactive oxygen species (ROS) responsible for a fundamental innate defence against pathogens in various host organisms [27]. MsrB is an antioxidant protein protecting organisms from the cytotoxic effects of ROS and therefore from cell death. This protein is crucial for the virulence of *S. typhimurium* and the immune evasion of *Schistosoma mansoni* [28, 29]. Whether msrB has a similar role in *Giardia* assemblage A pathogenicity remains unclear.

The difference in secretion between the two human infective assemblages observed in this study may also go some way to explaining the differences in pathogenesis, symptoms and host range previously observed between assemblage A and B.

The most abundant proteins, in both human isolates, primarily belong to four families of proteins: *Giardia* cathepsin B family *(*GCATB*)*, high cysteine membrane proteins (HCMPs), variant surface proteins (VSPs) and tenascins.

The cathepsin B family of *Giardia* are confirmed virulence factors involved in many of the parasite's processes such as encystation and excystation [6]; secreted GCATBs degrade host IL-8 and inhibit neutrophil chemotaxis [3]. GCATB contains secreted and non-secreted trophozoite expressed proteins; the orthologues of which are predominantly common to GS (B) and WB (A) assemblages (Fig 1). Expression of sixteen *G*CATBs was proteomically confirmed, of which eleven were shown by our proteomic analysis to be secreted. These eleven fell into six orthologous groups and for three of these groups all group members were shown to be secreted. Secreted *G*CTAB GL50803_15564 (WB) and its ortholog GL50581_2036 (GS) show a dN/dS values of >26 indicative of strong positive selective pressure. Interestingly when GS was resequenced, GL50803_15564 was found to comprise three recently diverged orthologs (GSB_153537, GSB_155477, GSB_150353) and it may be that the positive selection pressure observed has been generated as a result of recent gene duplications in the assemblage B strain. GL50803_16779, an assemblage A (WB) GCATB, has previously been shown to be up-regulated and involved in trophozoite motility in early pathogenesis of *Giardia* [15]. In this study, this protein was found to be in WB top 5 secreted proteins (Table S4); its GS ortholog (GL50581_78) was also present but at a considerably lower level suggesting that for this GCATB may play a more significant role in assemblage A than assemblage B.

HCMPs are an enigmatic group of proteins with few associated functional studies. They may protect trophozoites against proteolysis [30, 31] and oxidative damage [32]. In *Giardia*, it appears that one lineage of HCMPs has given rise to the VSPs, whilst another has given rise to a group with high homology to mammalian tenascins. Tenascin, VSPs and HCMPs are then related multi-gene families that together form the largest group of proteins enriched in the *Giardia* supernatants. Interestingly, when aligned and analysed phylogenetically the secreted tenascins segregate into a monophyletic group (Figure S4). Both WB and GS

orthologs of five tenascin gene products were secreted and in WB two other secreted tenascins were also detected that were not detected for the GS strain (Figure 1B).

VSPs are well-characterised surface glycoproteins with transmembrane domains, which are expressed one at a time by *Giardia* trophozoites through an RNAi regulated mechanism. They are quintessential virulence factors, responsible for antigenic variation. VSPs are hypervariable by nature and thus it is to be expected that they do not form orthologous pairs. This was the case for most we observed, intriguingly though, a few proteins annotated as VSPs were conserved between isolates suggesting that they are not actually VSPs and would not be subject to "one at a time" controlled expression - but are actually misannotated HCMPs which may have a conserved function in both GS and WB isolates. This study was not able to resolve whether the enrichment of such proteins in the supernatant observed is due to clipping or shedding from the parasite surface or whether the proteins are also secreted.

Tenascins are characterised by the presence of epidermal growth factor (EGF) repeats and are able to act as ligands for EGF receptors. Mammalian tenascins are extracellular matrix proteins, which modulate cell adhesion and migration [33]. They appear to have evolved from a group of proteins specific to vertebrates, presumably co-evolving with the EGF receptor and so the presence of homologous proteins in *Giardia* evolving independently from HCMPs is a clear example of the kind of convergent evolution best described as molecular mimicry. Interestingly, a *Giardia* tenascin (WB-GL50803_8687/GS-GL50581_4316), secreted by both strains and *Giardia* tenascin (WB-GL50803_14573/GS-GL50581_1475) secreted only by WB strain (Table 1 and S4) were found to be induced by host soluble factors and implicated in regulation of trophozoite attachment [15], supporting the case for secreted tenascins acting as virulence factors in *Giardia* pathogenesis.

Most published studies concerning host cell-*Giardia* interactions have focused on the effects on the host intestinal epithelia upon attachment of the trophozoites to the cells. In this study, we have shown that diluted supernatant obtained from the steady growth of *Giardia* trophozoites *in vitro* has an effect on the intestinal cell function. The effect observed on chloride secretion by *Giardia* supernatants indicates that *Giardia* secretes a soluble factor, which is likely affecting secretion across the intestinal epithelial cells. Physiologically, cultured intestinal cells show sensitivity to *Giardia* proteins released by the parasite even at high dilution. Fig2D demonstrates that intestinal epithelial cells when acutely exposed to such *Giardia* proteins lose the ability to stimulate CFTR and calcium-activated chloride channels. The clear implication being that virulence determinants released from *Giardia* trophozoites interact with epithelial cell receptors and ion channels.

In this analysis, we have identified the proteins that are secreted by human infective *Giardia* trophozoites. Just two groups form the majority of these proteins: *G*CATBs and the HCMP superfamily encoding known virulence factors in addition to an abundant extracellular nuclease and an oxygen-fixing enzyme. The elucidation of this group of proteins dramatically increases our understanding of the pathogenic mechanisms underlying giardiasis at a molecular level. The genes encoding GCATBs and HCMP superfamily proteins are among the most heterogeneous of all genes between assemblages. Their probable role in interaction with the host and luminal environment is supported by the very high dN/dS values of some

family members. Correlation of variation within assemblages at these loci with strain virulence is the essential next step for their use in diagnosis of virulent strains, risk assessment and disease prognosis.

Our results indicate that *Giardia* secretions are sufficient to disable normal function in enteric epithelial making cells less able to extract fluids from the lumen. In particular, they implicate PNPO, an extracellular nuclease, *G*CATBs and tenascins. The fact that both extracellular nuclease and *G*CATBs can be involved in the degradation of the intestinal mucus layer and that both *G*CATBs and tenascins can be associated with intestinal intracellular junction disruption suggests collaboration between these proteins. Therefore, we propose a pathogenic mechanism (Fig 3) whereby PNPO produces a reducing environment favouring growth of trophozoites, the extracellular nuclease degrades the outer layer of the intestinal mucus improving access for *G*CATBs for further degradation of the protective mucous barrier and subsequent disruption of intestinal intracellular junctions. Lastly, tenascins are involved in maintaining intestinal cell separation by ligation of EGF receptors present at the surface of intestinal cells and exacerbation of epithelial damage via increased levels of apoptosis amongst these more detached cells. Once the intestinal barrier is breached by the actions of giardia secreted virulence factors, the sites of damage become prone to secondary infection by other opportunist microbes resident in the intestinal lumen and sensitive to irritation by allergens in foodstuffs leading to further inflammation and to the characteristic symptoms of the disease. Further investigations are necessary to verify this proposed mechanism of pathogenesis of giardiasis.

METHODS

Proteomic Analysis.

Samples preparation

Giardia trophozoites from the genome reference strains WB (assemblage A,ATCC_50803) and GS (assemblage B, ATCC_50581), were cultured in TYI-S-33 under standard conditions (5% CO_2)[34] and harvested during the midlog phase of their *in vitro* growth curves. The total trophozoites (adhered and non-adhered) were washed 3 x in phosphate buffer saline (PBS) and then incubated in non-supplemented DMEM, with antibiotics to conserve an axenic milieu, for 45 minutes at 37°C (Figure S5 A) [38]. After incubation, an aliquot was analysed by flow cytometry to evaluate the viability of the *Giardia* samples. Trophozoites and supernatant were separated by centrifugation and both trophozoite pellet and supernatant were harvested. Proteins contained in supernatant were concentrated in Vivaspin columns (3,000 MWCO) with 25 mM ammonium bicarbonate (Ambic) (Figure S5 B) [39]. Supernatants were analysed by SDS PAGE [40] and were tested on cultured epithelial cells (Caco-2) to ensure the presence of proteins and biological activity (see below). Supernatants and pellets were sent to the Institute of Infection and Global Health at the University of Liverpool for mass spectrometry analysis (Figure S3) [41].

Protein samples were dispensed into low protein-binding microcentrifuge tubes (Sarstedt, Leicester, UK) and made up to 160 µl by addition of 25 mM Ambic. The proteins were denatured using 10 µl of 1% (w/v) RapiGest^{TM} (Waters MS Technologies, Manchester, UK) in

25 mM Ambic followed by three cycles of freeze-thaw, and two cycles of 10 min sonication in water bath. Sample was then incubated at 80 °C for 10 min and reduced (addition of 10 µl of 60 mM DTT and incubation at 65 °C for 10 min) and alkylated (addition of 10 µl of 180 mM iodoacetamide and incubation at room temperature for 30 min in the dark). Trypsin (Sigma-Aldrich, Dorset, UK) was reconstituted in 50 mM acetic acid to a concentration of 0.2 µg/µl. Digestion was performed by the addition of 10 µl of trypsin to the sample followed by incubation at 37 °C overnight. The RapiGest™ was removed from the sample by acidification (1 µl of trifluoroacetic acid and incubation at 37 °C for 45 min) and centrifugation (15,000 × *g* for 15 min) [41]. After protein digestion, 1 µg of digest were injected into both the Orbitrap Velos and the Q-Exactive MS, for all samples.

Orbitrap Velos

Peptide mixtures were analysed by on-line nanoflow liquid chromatography using the nanoACQUITY-nLC system (Waters MS technologies, Manchester, UK) coupled to an LTQ-Orbitrap Velos (ThermoFisher Scientific, Bremen, Germany) mass spectrometer equipped with the manufacturer’s nanospray ion source. The analytical column (nanoACQUITY UPLC™ BEH130 C18 15cm x 75µm, 1.7µm capillary column) was maintained at 35°C and a flow-rate of 300nl/min. The gradient consisted of 3-40% acetonitrile in 0.1% formic acid for 90min then a ramp of 40-85% acetonitrile in 0.1% formic acid for 3 min. Full scan MS spectra (m/z range 300-2000) were acquired by the Orbitrap at a resolution of 30,000. Analysis was performed in data dependant mode. The top 20 most intense ions from MS1 scan (full MS) were selected for tandem MS by collision induced dissociation (CID) and all product spectra were acquired in the LTQ ion trap. Ion trap and orbitrap maximal injection times were set to 50ms and 500ms, respectively.

Q-Exactive MS

Digests (2 µl) were analysed on a 50cm Easy-Spray column with an internal diameter of 75µm, packed with 2µm C18 particles, fused to a silica nano-electrospray emitter (Thermo Fisher Scientific). Reversed phase liquid chromatography was performed using the Ultimate 3000 nano system with a binary buffer system consisting of 0.1% formic acid (buffer A) and 80% acetonitrile in 0.1% formic acid (buffer B). The peptides were separated by a linear gradient of 5-40% buffer B over 110 min at a flow rate of 300nl/min. The column was operated at a constant temperature of 35°C and the LC system coupled to a Q-Exactive mass spectrometer (Thermo Fisher Scientific). The Q-Exactive was operated in data-dependent mode with survey scans acquired at a resolution of 70,000 at m/z 200. Up to the top 10 most abundant isotope patterns with charge states +2, +3 and/or +4 from the survey scan were selected with an isolation window of 2.0Th and fragmented by higher energy collisional dissociation with normalized collision energies of 30. The maximum ion injection times for the survey scan and the MS/MS scans were 250 and 100ms, respectively, and the ion target value was set to 1E6 for survey scans and 1E4 for the MS/MS scans. Repetitive sequencing of peptides was minimized through dynamic exclusion of the sequenced peptides for 20s.

Data analysis

Thermo RAW files were imported into Progenesis LC–MS (version 4.1, Nonlinear Dynamics). Replicate runs were time-aligned using default settings and an auto-selected run as a reference. Peaks were picked by the software using default settings and filtered to include only peaks with a charge state of between +2 and +6. Peptide intensities of replicates were normalised against the reference run by Progenesis LC-MS. Spectral data were transformed to .mgf files with Progenesis LC–MS and exported for peptide identification using the PEAKS Studio 7 (Bioinformatics Solutions Inc.) search engine. Multiple search engine platform provided by PEAKS Studio named inChorus was used, which combines searching results from PEAKS DB (Bioinformatics Solutions Inc.), Mascot (Matrix Science), OMSSA (National Center for Biotechnology Information) and X!Tandem (Global Proteome Machine Organization). Tandem MS data were searched against a custom database that contained the common contamination and internal standards, GiardiaDB-3.1_GintestinalisAssemblageA_AnnotatedProteins or GiardiaDB-3.1_GintestinalisAssemblageB_AnnotatedProteins. The search parameters for Orbitrap-Velos were as follows; precursor mass tolerance was set to 10ppm and fragment mass tolerance was set to 0.5 Da. One missed tryptic cleavage was permitted. Carbamidomethylation was set as a fixed modification and oxidation (M) set as a variable modification. The search parameters for Q Exactive were as follows; precursor mass tolerance was set to 10ppm and fragment mass tolerance was set to 0.01 Da. One missed tryptic cleavage was permitted. Carbamidomethylation was set as a fixed modification and oxidation (M) set as a variable modification. The false discovery rates (FDR) were set at 1% and at least two unique peptides were required for reporting protein identifications. Protein abundance (iBAQ) was calculated as the sum of all the peak intensities (from Progenesis output) divided by the number of theoretically observable tryptic peptides [16]. Protein abundance was normalised by dividing the protein iBAQ (intensity based absolute quantification) value by the summed iBAQ values for that sample. The reported abundance is the mean of the biological replicates.

The mass spectrometry proteomics data have been deposited to the ProteomeXchange Consortium via the PRIDE partner repository [24] with the dataset identifier PXD004398 and 10.6019/PXD004398.

Electrophysiology.

Giardia trophozoites culture

Giardia lamblia WB and GS strain as well as the patients' strains (obtained from 3 patients with giardiasis from the NNUH) were grown in filter sterilized, modified TYI-S-33 medium with 10% adult bovine serum and 0.05% bovine bile [28] at 37°C in microaerophilic conditions and sub-cultured when confluent. To collect parasites for experiments, the medium was removed from the culture to eliminate unattached or dead parasites. The tube was refilled with cold, sterile medium and trophozoites detached by chilling on ice for 15 minutes.

Parasites were collected by centrifugation (1500 x g for 5 minutes at 4 °C) and washed once with the plating medium of 90% complete DMEM/10% *Giardia* medium. Parasites were then counted using a haemocytometer and diluted to the appropriate number.

To collect *Giardia* supernatant for experiments, the *Giardia* culture bottle was placed on ice for 15 minutes. The bottle then underwent centrifugation (1500 x g for 5 minutes at 4 °C). The supernatant was then collected and filtered 3 times using a 15mm diameter syringe filters (0.2µm pore size). Subsequently the post-filtered *Giardia* supernatant was diluted 1:1000 and saved in -20°C freezer until required.

Mammalian cell line (CaCo-2) preparation

CaCo-2 cells (passages 20-25) were grown in DMEM supplemented with nonessential amino acids, penicillin (12 IU/ml), streptomycin (12µg/ml), gentamycin (47 µg/ml) and 20% (vol/vol) heat inactivated fetal calf serum (all from AMIMED, Bioconcept). The cells were seeded at a density of 6 x 10^4 cells/cm^2 in 6-well Transwell filters (0.4 µm pore size) and cultured for 7-15 days until confluent. Confluent monolayers were then used for electrophysiological experiments, for co-culture experiments with *Giardia* parasites or for culture with *Giardia* supernatants [42].

CaCo-2 co-culture experiments with *Giardia* or *Giardia* supernatant

Confluent CaCo-2 monolayers were taken and the CaCo-2 cell media was removed and replenished with a combination of 90% complete DMEM/10% *Giardia* medium plus or minus *Giardia* trophozoites (100,000 total parasites per insert). Control cultures were maintained in a separate plate to prevent parasite contamination. Control inserts were inspected under the microscope to ensure there was no *Giardia* cross contamination. The co-cultures were incubated at 37°C and 5% CO_2 for 24 hours, after which the *Giardia* parasites were removed [42].

Confluent Caco-2 monolayers were also cultured with diluted (1:1000) *Giardia* supernatants for 24 hours. Briefly, the culture media was removed from the insert and Caco-2 cell media was replaced with a combination of 99.9% complete DMEM/ 0.1% *Giardia* medium plus or minus *Giardia* supernatant [42].

Transepithelial electrical resistance (TEER) Assay

Monolayers of CaCo-2 cells were grown on 6-well Transwell filters (0.4 µm pore size) for 7-15 days until confluent. The development of the polarised monolayer was assessed by measuring the TEER over a 7-15 day period. Once confluent, *Giardia* were added to the apical side of the Transwell filter and incubated for 24 hours. The integrity of the confluent polarised monolayer was assessed by measuring the TEER before and/or after apical infection by *Giardia* [42].

Electrophysiology Assay

Monolayers of CaCo-2 cells on Transwell filters were mounted into a Physiological Instruments EM-CSYS-2 Using chamber set-up, after establishment of a confluent monolayer and the short circuit current (I_{SC}) across the monolayer was continuously measured [42].

Both sides of the epithelium were bathed in 5ml of Krebs Henseleit solution that was continuously circulated through the half chambers, maintained at 37°C and continuously bubbled with 95% O_2 / 5% CO_2. The composition of the Krebs Henseleit bath solution used was similar to that used by Cuthbert [35] and had the following composition (in mM): NaCl 118, KCl 4.7, $CaCl_2$ 2.5, $MgCl_2$ 1.2, $NaHCO_3$ 25, KH_2PO_4 1.2 and glucose 11.1 (pH 7.4). The permeable supports were left for 30 mins to equilibrate before experiments were started. All filters were treated with 10µM amiloride apically to eliminate electrogenic sodium absorption through epithelial sodium channels (ENaC) [42].

Data analysis

I_{SC} was continuously monitored across the monolayers by a Physiological Instruments Multichannel Voltage/Current Clamp (VCC MC6) through 3M KCl/agar, Ag/$AgCl_2$ cartridge electrodes (Physiologic Instruments), and the raw data for I_{sc}, transepithelial resistance and transepithelial voltage were recorded using Acquire and Analyse version 1.3 software (Physiological Instruments). Data were exported to Microsoft Excel initially and then into GraphPad Prism version 5.0 for Windows package for data representation and statistical analysis.

Chemicals and Inhibitors

Forskolin (10µM), UTP (100µM), Amiloride (10µM), and DIDS (100µM) were obtained from Sigma Aldrich, and GlyH-101 (50 µM) was obtained from Merck Chemicals. Stock solutions of Amiloride (10mM), GlyH-101 (50mM) were made by dissolving in DMSO. Final concentrations of drugs are as indicated in the text or figures and where produced by adding the appropriate volume of stock concentration to 5ml of either the basolateral or apical bathing solution.

Phylogeny

To look for sequence similarities between proteins of interest from a same protein family, the coding sequences of these proteins were retrieved from GiardiaDB (v 3.1, 4.0 and 5.0), aligned and compared using ClustalW.

Phylogenetic trees were built for these proteins, via Maximum likelihood approach using MEGA software (v. 6.06).

Availability of Supporting Data

All proteomic datasets are held by and can be accessed for free at the European Bioinformatics PRoteomics IDEntifications (PRIDE) database (accession number PXD004398). Free Integrated functionality with other *Giardia* large datasets hosted at EupathDB [36]. Supporting data, including raw data in .csv format, alignments and phylogenetic analyses, are also available via the *GigaScience* repository GigaDB [37]. All protocols used in this study are available and can be accessed at protocols.io database [38-43].

Abbreviations: ABC (ATP-binding cassette); ADI (Arginine Deiminase); Ambic (Ammonium bicarbonate); ATP (Adenosine triphosphate); CaCo-2 (Human colonic adenocarcinoma

derived epithelial cell line-2); DMEM (Dulbecco's Modified Eagle Medium); DIDS (4,4'-disothiocyanatostibene-2,2'-sulfonic acid); EF-1α (Elongation Factor 1-alpha); EGF (Epidermal growth factor); ENaC (Epithelial Sodium Channel); FDR (False discovery rate); FMN (Flavin mononucleotide); *G*CATB (*Giardia* cathepsin B); GlyH101; HCMP (High cysteine membrane protein); iBAQ (Intensity based absolute quantification); IEC (Intestinal Epithelial Cells); IL (Interleukine); Isc (Short-circuit current); mRNA (messenger RNA); msrB (peptide methionine sulfoxide reductase B); OCT (Ornithine Carbamoyltransferase); ORF (Open reading frame); P (Pellet); PNPO (Pyridoxamine 5'-phosphate oxidase); rcf (Relative centrifugal force); RNA (Ribonucleic acid); ROS (Reductive oxygen species); rpm (Rotations per minute); SP (Supernatant); PRIDE (PRoteomics IDEntifications); TEER (Transepithelial electrical resistance), VSP (Variant surface protein)

Conflicts of Interest

The authors declare that they have no competing interests

Authors' Contributions

K.T., J.M.W., J.P.W and P.H. conceived and designed the studies. K. T. and A. D. co-ordinated the experiments. A.D. and S.A.N. performed the electrophysiology with J.P.W. A.D. performed the Flow Cytometry with D.S. A.D. prepared the proteomic samples. D.X. performed the proteomic experiments. A.D. and M.B. performed the phylogenetic analysis. All authors contributed to the analysis of the data sets obtained and preparation of Figures and Tables. The manuscript was drafted by A.D. and K.T. and improved and approved prior to submission by all co-authors.

Acknowledgements

The research leading to these results was primarily funded from the European Union Seventh Framework Programme ([FP7/2007-2013] [FP7/2007-2011]) under Grant agreement no: 311846. PRH is supported by the National Institute for Health Research Health Protection Research Unit (NIHR HPRU) in Gastrointestinal Infections at the University of Liverpool in partnership with Public Health England (PHE), and in collaboration with University of East Anglia, University of Oxford and the Institute of Food Research. Professor Hunter is based at University of East Anglia. The views expressed are those of the author(s) and not necessarily those of the NHS, the NIHR, the Department of Health or Public Health England. We thank Susanne Warrenfeltz and the EuPathDB team for invaluable assistance in making the integrated datasets available.

REFERENCES

1. Esch KJ and Petersen CA. Transmission and epidemiology of zoonotic protozoal diseases of companion animals. Clinical microbiology reviews. 2013;26 1:58-85. doi:10.1128/CMR.00067-12.
2. Nash TE, Herrington DA, Losonsky GA and Levine MM. Experimental human infections with Giardia lamblia. The Journal of infectious diseases. 1987;156 6:974-84.
3. Cotton JA, Bhargava A, Ferraz JG, Yates RM, Beck PL and Buret AG. Giardia duodenalis cathepsin B proteases degrade intestinal epithelial interleukin-8 and attenuate

interleukin-8-induced neutrophil chemotaxis. Infect Immun. 2014; doi:10.1128/IAI.01771-14.
4. Turk V, Stoka V, Vasiljeva O, Renko M, Sun T, Turk B, et al. Cysteine cathepsins: from structure, function and regulation to new frontiers. Biochim Biophys Acta. 2012;1824 1:68-88. doi:10.1016/j.bbapap.2011.10.002.
5. Musil D, Zucic D, Turk D, Engh RA, Mayr I, Huber R, et al. The refined 2.15 A X-ray crystal structure of human liver cathepsin B: the structural basis for its specificity. EMBO J. 1991;10 9:2321-30.
6. DuBois KN, Abodeely M, Sakanari J, Craik CS, Lee M, McKerrow JH, et al. Identification of the major cysteine protease of Giardia and its role in encystation. The Journal of biological chemistry. 2008;283 26:18024-31. doi:M802133200 [pii]10.1074/jbc.M802133200.
7. Sajid M and McKerrow JH. Cysteine proteases of parasitic organisms. Molecular and biochemical parasitology. 2002;120 1:1-21. doi:S0166685101004388 [pii].
8. Rodriguez-Fuentes GB, Cedillo-Rivera R, Fonseca-Linan R, Arguello-Garcia R, Munoz O, Ortega-Pierres G, et al. Giardia duodenalis: analysis of secreted proteases upon trophozoite-epitheial cell interaction in vitro. Mem Inst Oswaldo Cruz. 2006;101 6:693-6.
9. Paget TA and James SL. The mucolytic activity of polyamines and mucosal invasion. Biochem Soc Trans. 1994;22 4:394S.
10. Wampfler PB, Tosevski V, Nanni P, Spycher C and Hehl AB. Proteomics of Secretory and Endocytic Organelles in Giardia lamblia. PLoS One. 2014;9 4:e94089. doi:10.1371/journal.pone.0094089.
11. Faso C, Bischof S and Hehl AB. The proteome landscape of Giardia lamblia encystation. PLoS One. 2013;8 12:e83207. doi:10.1371/journal.pone.0083207.
12. Lingdan L, Pengtao G, Wenchao L, Jianhua L, Ju Y, Chengwu L, et al. Differential dissolved protein expression throughout the life cycle of Giardia lamblia. Exp Parasitol. 2012;132 4:465-9. doi:10.1016/j.exppara.2012.09.014.
13. Ringqvist E, Palm JE, Skarin H, Hehl AB, Weiland M, Davids BJ, et al. Release of metabolic enzymes by Giardia in response to interaction with intestinal epithelial cells. Molecular and biochemical parasitology. 2008;159 2:85-91. doi:S0166-6851(08)00056-X [pii]10.1016/j.molbiopara.2008.02.005.
14. Roxstrom-Lindquist K, Palm D, Reiner D, Ringqvist E and Svard SG. Giardia immunity--an update. Trends Parasitol. 2006;22 1:26-31. doi:S1471-4922(05)00311-9 [pii]10.1016/j.pt.2005.11.005.
15. Emery SJ, Mirzaei M, Vuong D, Pascovici D, Chick JM, Lacey E, et al. Induction of virulence factors in Giardia duodenalis independent of host attachment. Scientific reports. 2016;6:20765. doi:10.1038/srep20765.
16. Schwanhausser B, Busse D, Li N, Dittmar G, Schuchhardt J, Wolf J, et al. Global quantification of mammalian gene expression control. Nature. 2011;473 7347:337-42. doi:10.1038/nature10098.
17. Emery SJ, Lacey E and Haynes PA. Data from a proteomic baseline study of Assemblage A in Giardia duodenalis. Data in brief. 2015;5:23-7. doi:10.1016/j.dib.2015.08.003.
18. Emery SJ, Lacey E and Haynes PA. Quantitative proteomic analysis of Giardia duodenalis assemblage A: A baseline for host, assemblage, and isolate variation. Proteomics. 2015;15 13:2281-5. doi:10.1002/pmic.201400434.
19. Jerlstrom-Hultqvist J, Ankarklev J and Svard SG. Is human giardiasis caused by two different Giardia species? Gut Microbes. 2010;1 6:379-82. doi:10.4161/gmic.1.6.13608.

20. Skarin H, Ringqvist E, Hellman U and Svard SG. Elongation factor 1-alpha is released into the culture medium during growth of Giardia intestinalis trophozoites. Exp Parasitol. 2011;127 4:804-10. doi:S0014-4894(11)00017-8 [pii]10.1016/j.exppara.2011.01.006.
21. Condeelis J. Elongation factor 1 alpha, translation and the cytoskeleton. Trends Biochem Sci. 1995;20 5:169-70. doi:S0968000400889987 [pii].
22. Nandan D and Reiner NE. Leishmania donovani engages in regulatory interference by targeting macrophage protein tyrosine phosphatase SHP-1. Clinical immunology. 2005;114 3:266-77. doi:10.1016/j.clim.2004.07.017.
23. Di Matteo A, Scandurra FM, Testa F, Forte E, Sarti P, Brunori M, et al. The O2-scavenging flavodiiron protein in the human parasite Giardia intestinalis. The Journal of biological chemistry. 2008;283 7:4061-8. doi:10.1074/jbc.M705605200.
24. Kamitani T, Kito K, Nguyen HP, Fukuda-Kamitani T and Yeh ET. Characterization of a second member of the sentrin family of ubiquitin-like proteins. The Journal of biological chemistry. 1998;273 18:11349-53.
25. Sauvage V, Aubert D, Escotte-Binet S and Villena I. The role of ATP-binding cassette (ABC) proteins in protozoan parasites. Molecular and biochemical parasitology. 2009;167 2:81-94. doi:10.1016/j.molbiopara.2009.05.005.
26. Malys N and McCarthy JE. Translation initiation: variations in the mechanism can be anticipated. Cellular and molecular life sciences : CMLS. 2011;68 6:991-1003. doi:10.1007/s00018-010-0588-z.
27. Kim HY and Gladyshev VN. Methionine sulfoxide reduction in mammals: characterization of methionine-R-sulfoxide reductases. Molecular biology of the cell. 2004;15 3:1055-64. doi:10.1091/mbc.E03-08-0629.
28. Denkel LA, Horst SA, Rouf SF, Kitowski V, Bohm OM, Rhen M, et al. Methionine sulfoxide reductases are essential for virulence of Salmonella typhimurium. PloS one. 2011;6 11:e26974. doi:10.1371/journal.pone.0026974.
29. Oke TT, Moskovitz J and Williams DL. Characterization of the methionine sulfoxide reductases of Schistosoma mansoni. The Journal of parasitology. 2009;95 6:1421-8. doi:10.1645/GE-2062.1.
30. Davids BJ, Reiner DS, Birkeland SR, Preheim SP, Cipriano MJ, McArthur AG, et al. A new family of giardial cysteine-rich non-VSP protein genes and a novel cyst protein. PLoS One. 2006;1:e44. doi:10.1371/journal.pone.0000044.
31. Nash TE. Surface antigenic variation in Giardia lamblia. Mol Microbiol. 2002;45 3:585-90. doi:3029 [pii].
32. Requejo R, Hurd TR, Costa NJ and Murphy MP. Cysteine residues exposed on protein surfaces are the dominant intramitochondrial thiol and may protect against oxidative damage. The FEBS journal. 2010;277 6:1465-80. doi:10.1111/j.1742-4658.2010.07576.x.
33. Chiquet-Ehrismann R and Chiquet M. Tenascins: regulation and putative functions during pathological stress. The Journal of pathology. 2003;200 4:488-99. doi:10.1002/path.1415.
34. Keister DB. Axenic culture of Giardia lamblia in TYI-S-33 medium supplemented with bile. Trans R Soc Trop Med Hyg. 1983;77 4:487-8.
35. Cuthbert AW. Assessment of CFTR chloride channel openers in intact normal and cystic fibrosis murine epithelia. Br J Pharmacol. 2001;132 3:659-68. doi:10.1038/sj.bjp.0703859.
36 EuPathDB: Eukaryotic Pathogen Database Resources : Giardia DB 35 http://giardiadb.org/giardiadb/showXmlDataContent.do?name=XmlQuestions.News#giardiadb12_17_release accessed 11 Jan 2018

37 Dubourg A, Xia D, Winpenny JP, Al-Naimi S, Bouzid M, Sexton DW, et al. Supporting data for "Giardia Secretome Highlights Secreted Tenascins as a Key Component of Pathogenesis" *GigaScience* Database. 2018. http://dx.doi.org/10.5524/100381

38 Dubourg A, Xia D, Winpenny JP, Al Naimi S, Bouzid M, Sexton DW, et al. *Giardia* supernatant cleansing protocol. Protocols.io.2018. dx.doi.org/10.17504/protocols.io.k96cz9e

39 Dubourg A, Xia D, Winpenny JP, Al Naimi S, Bouzid M, Sexton DW, et al. Concentration of *Giardia* supernatant proteins. Protocols.io.2018. dx.doi.org/10.17504/protocols.io.mccc2sw

40 Dubourg A, Xia D, Winpenny JP, Al Naimi S, Bouzid M, Sexton DW, et al. Sodium Dodecyl Sulfate PolyAcrylamide Gel Electrophoresis (SDS-PAGE) with Sypro straining. Protocols.io.2018. dx.doi.org/10.17504/protocols.io.mcdc2s6

41 Dubourg A, Xia D, Winpenny JP, Al Naimi S, Bouzid M, Sexton DW, et al. Proteomics Analysis. Protocols.io.2018. dx.doi.org/10.17504/protocols.io.mcec2te

42 Dubourg A, Xia D, Winpenny JP, Al Naimi S, Bouzid M, Sexton DW, et al. *Giardia* electrophysiological assays. Protocols.io.2018. dx.doi.org/10.17504/protocols.io.mcfc2tn

43 Dubourg A, Xia D, Winpenny JP, Al Naimi S, Bouzid M, Sexton DW, et al. *Giardia* Secretome Highlights Secreted Tenascins as a Key Component of Pathogenesis Protocols.io.2018. dx.doi.org/10.17504/protocols.io.k97cz9n

Figures Legends:

Table 1: The secretome of human infective *Giardia* trophozoites of assemblage A and B have a conserved repertoire of abundant secreted factors identified by both Orbitrap MS and Q-Exactive MS. 15 proteins were identified as most likely to be secreted by both GS and WB isolates. 12 are annotated proteins and 3 are hypothetical proteins. Proteins are ranked according to GS Q-Exactive Supernatant (SP) protein abundance, from most to least abundant. Of the 12 annotated proteins, 5 are tenascins and 3 are related high cysteine membrane proteins or VSP and three are cathepsin Bs. The other annotated abundant secreted protein is an extracellular nuclease. Protein ranking represents the proteins rank within this table, from most to least abundant. Detailed breakdown of the secretome for each assemblage by each method are provided in Supplemental tables 1-4.

Table 2: Human infective *Giardia* trophozoites of assemblage A and B secrete a small set of different proteins. Seven proteins were identified as most likely to be secreted by either GS or WB isolates. Two are most likely to be secreted by GS isolate (shown in *italic*) and five are most likely to be secreted by WB isolate (shown in **bold**). One GS isolate- and two WB isolate-secreted were identified only via Q-Exactive MS in the other assemblage's dataset (shown in red). The abundance ranking represents the protein ranking within the secretome of both assemblages according to their abundances in the supernatant.

Figure 1: Neighbour joining tree showing clustering of A) Cathepsin B and B) Tenascin gene families. Genes were retrieved by gene name search on GiardiaDB. Gene sequences were downloaded and aligned using ClustalW generated with MEGA 6 software package. Maximum composite likelihood method was used, with 2000 bootstrap replicates. Bootstrap values greater than 50% are shown above the branches. ◆ proteins confirmed to be secreted using our proteomic analysis.

Figure 2: The effect of co-culture with *Giardia* or *Giardia* supernatants on the electrophysiological properties of CaCo-2 monolayers. A) Transepithelial electrical resistance (TEER) in CaCo-2 monolayers following seeding on permeable supports. Data shows increase in TEER as monolayer develops. Confluence occurred around Day 6. *Giardia* were added on Day 6 after confluent monolayer formed and co-cultured with the Caco-2 monolayer for 24 hours. TEER was measured after 24 hours and compared to TEER in monolayers that had not been exposed to *Giardia* (n=6). **B)** A representative short circuit current (Isc) against time recording from single monolayers of CaCo-2 cells in an Ussing chamber. The trace shows the activation of CFTR chloride channels (basolateral application of 10 µM Forskolin) and calcium-activated chloride channels (basolateral application of 100 µM UTP). Specificity of activation is confirmed by inhibition of Isc by the specific CFTR channel blocker, GlyH101; and specific calcium-activated chloride channel blocker, DIDS. The effect on Isc of 24 hour co-incubation of CaCo-2 monolayers with *Giardia* or with *Giardia* supernatant (1:1000 dilution) is also shown. **C)** Effect of 24 hour co-incubation of CaCo-2 monolayers with different strains of *Giardia* (WB, GS and patient samples) on forskolin-stimulated and UTP-stimulated Isc (n=3). **D)** Effect of supernatant co-incubation from different strains of *Giardia* (WB, GS and patient samples) on forskolin-stimulated and UTP-stimulated Isc (n=3) from Caco-2 monolayers. The results were analysed by student's t-test and expressed as mean values ± standard error mean (SEM). Significant difference expressed as *P<0.05, **P<0.01 compared to control.

Figure 3: Proposed novel mechanism of pathogenicity for *Giardia* involving PNPO, extracellular nuclease, *G*CATB, Tenascin. PNPO ( ) renders the intestinal environment more favourable to trophozoite's growth. Once a new *Giardia* colony is established, trophozoites release extracellular nuclease ( ), GCATB ( ) and Tenascin ( ). Extracellular nuclease may contribute to reducing the viscosity of the intestinal outer mucus layer, while *G*CATB may degrade mucins and disrupt intracellular junction. Finally, Tenascins may maintain intestinal cells apart by attaching to the EGF receptors present at the surface of intestinal cells that could over time lead to the apoptosis of these isolated intestinal cells.

Extended data legends:

Table S1: List of the *Giardia* assemblage A (WB strain) lineage-specific proteins identified via Orbitrap and Q-Exactive MS.
Protein sequences were compared to their coding sequence and matched to their orthologs in assemblage B (GS strain) using *Giardia* database: *Giardia*DB.org. Annotated proteins are highlighted in red and hypothetical proteins in blue. Proteins were ranked according to Q-Exactive S Supernatant (S) abundance, from most to least abundant.

Table S2: List of the *Giardia* assemblage B (GS strain) lineage-specific proteins identified via Orbitrap and Q-Exactive MS.
Protein sequences were compared to their coding sequence and matched to their orthologs in assemblage A (WB strain) using *Giardia*DB.org. Annotated proteins are highlighted in red and hypothetical proteins in blue. Proteins were ranked according to Q-Exactive abundance (S) abundance, from most to least abundant.

Table S3: List of the 86 proteins most likely to be secreted by *Giardia* GS strain trophozoites. 31 proteins were identified via Orbitrap and Q-Exactive MS (**in bold)**.and 55 proteins were identified via Q-Exactive MS only (*in italic*).
59 proteins are annotated (shown in red) and 27 are hypothetical proteins (shown in blue). 10 proteins are lineage-specific. The 15 proteins identified as conserved between the two isolates are highlighted in grey. Only proteins identified via both techniques were considered as secreted and were included in the final analysis. Proteins are ranked according to Q-Exactive SP abundance from most to least abundant.

Table S4: List of the 61 proteins most likely to be secreted by *Giardia* WB strain trophozoites. 44 proteins were identified via Orbitrap and Q-Exactive MS (**in bold**) and 16 proteins were identified via Q-Exactive MS only (*in italic*)
53 proteins are annotated (shown in red) and 8 are hypothetical proteins (shown in blue). 12 proteins are lineage-specific. The 15 proteins identified as conserved between the two isolates are highlighted in grey. Only proteins identified via both techniques were considered as secreted and were included in the final analysis. Proteins are ranked according to Q-Exactive Supernatant (S) abundance from most to least abundant.

Table S5: List of the 1,553 proteins identified in the pellet of *Giardia* GS strain trophozoites via Q-Exactive and Orbitrap MS. Hypothetical and annotated proteins are shown in blue and black respectively. Proteins are ranked according to their Q-Exactive protein abundance-iBAQ values, from least to most abundant.

Tables S6: List of the 996 proteins identified in the supernatant of *Giardia* GS strain trophozoites via Q-Exactive and Orbitrap MS. Hypothetical and annotated proteins are shown in blue and black

respectively. Proteins are ranked according to their Q-Exactive protein abundance-iBAQ values, from least to most abundant.

Table S7: List of the 1,657 proteins identified in the pellet of *Giardia* WB strain trophozoites via Q-Exactive and Orbitrap MS. Hypothetical and annotated proteins are shown in blue and black respectively. Proteins are ranked according to their Q-Exactive protein abundance-iBAQ values, from least to most abundant.

Table S8: List of the 558 proteins identified in the supernatant of *Giardia* WB strain trophozoites via Q-Exactive and Orbitrap MS. Hypothetical and annotated proteins are shown in blue and black respectively. Proteins are ranked according to their Q-Exactive protein abundance-iBAQ values, from least to most abundant.

Figure S1: ***Giardia* trophozoites are viable after incubation in non-supplemented DMEM.** Parasites were chilled on ice for 15 min, washed 3 times in pre-warmed PBS, centrifuged 10 min at 3,000 rpm between each wash; and then incubated in pre-warmed non-supplemented DMEM for 45 min. at 37°C. After 45 min incubation, parasites were chilled on ice for 5 min and centrifuged 10 min at 3,000 rpm. Pellets were collected and respuspended in PBS (A2 and B2). Trophozoites collected from culture and respuspended in either PBS (A3 and B3) or 2% trigene (detergent) (A3 and B3) were used as live and death control respectively.
Proportion of living/dead trophozoites by flow cytometry. 5 µl of propidium iodide (PI) were added in each sample to stain DNA liberated in the milieu after cell death. Flow cytometry was performed using the BD Accuri™ C6 flow cytometer, with a blue laser (λ= 488 nm) and an optical filter 585/40. Gate P2 and P3 represent alive and dead trophozoites respectively. **A.** Flow cytometry analysis for GS isolate **B.** Flow cytometry analysis for WB isolate.
Data were analysed using the BD Accuri C-flow software (version 1.0.227.4).

Figure S2: Protein expression profile for *Giardia* assemblage A and B obtained with both MS platforms. Both GS and WB pellet (P) and supernatant (S) replicates were analysed via Orbitrap and Q-Exactive MS. Supernatant protein expression profile are similar to each other within each assemblage, so are pellet protein expression profiles (*Graph charts*). A total of 1,690 and 1,587 proteins were identified for assemblage B and A respectively (*Venn diagrams*) via both MS techniques. For assemblage A (WB isolate), 1,170 proteins were present in both dataset, 49 and 471 proteins were identified only in Orbitrap MS dataset and Q-Exactive MS datasets respectively. For assemblage B (GS isolate) 1,106 proteins were present in both datasets, 42 and 439 were identified only via Orbitrap Ms and Q-Exactive respectively for assemblage B.

Figure S3: *Giardia* proteins identified by Orbitrap and Q Exactive MS for assemblage A (WB isolate) and B (GS isolate). The Orbitrap MS analysis showed 639 and 426 proteins identified in both supernatant and pellet for assemblage B (GS isolate) and assemblage A (WB isolate) respectively, but also 51 (GS isolate) and 35 (WB isolate) in supernatant only and 461 (GS isolate) and 758 proteins (WB isolate) in pellet only respectively. The Q Exactive MS showed 946 and 490 proteins identified in both supernatant and pellet for assemblage B (GS isolate) and assemblage A (WB isolate) respectively, but also 27 (GS isolate) and 24 (WB isolate) in supernatant only and 569 (GS isolate) and 1,227 proteins (WB isolate) in pellet only respectively.
Proteins are ranked according to assemblage B Q-Exactive Supernatant (SP) expression from most to least abundant

Figure S4: Neighbour joining tree showing clustering of tenascins in the superfamily of High Cysteine Membrane Proteins (HCMP). Tenascin genes are highlighted in yellow. Genes were retrieved by gene name search on GiardiaDB. Gene sequences were downloaded and aligned using ClustalW generated

with MEGA 6 software package. Maximum composite likelihood method was used, with 2000 bootstrap replicates. Bootstrap values greater than 50% are shown. ◆ indicates secreted proteins as confirmed by our proteomic analysis.
Proteins are ranked according to assemblage A Q-Exactive Supernatant (SP) abundance from most to least abundant

Figure S5: Protocol to harvest *Giardia* pellet and supernatant for proteomic analysis.

A. **Preparation of *Giardia* supernatant and pellet samples for proteomic assay.** Parasites were chilled for 20 minutes, transferred into 15 ml falcon tubes and centrifuged at 3,000 rotations per minute (rpm) for 10 minutes. Supernatants were discarded and pellets were washed three times in warmed 1xPBS (4 ml, 2 ml and 1 ml respectively). Pellets were then incubated, under standard growth conditions and in filtered glass tubes, for 45 minutes at 37°C either in **1.** non-supplemented DMEM containing phenol red or **2.** non-supplemented phenol red-free DMEM. After 45 minutes incubation, parasites were chilled for 5 minutes, transferred in 15 ml falcon tubes and centrifuged at 3,000 rpm for 10 minutes. Pellets were stored at -20°C. Proteins present in supernatant samples were concentrated prior to perform Proteomic assay.

B. **Protocol to concentrate proteins contained in *Giardia* supernatant samples prior to Proteomic assay.** Supernatants were transferred in Vivaspin columns with a 3,000 MWCO and centrifuged at 12,000 relative centrifugal force (rcf) for 30 min. Proteins were washed up to 3 times with 25 mM Ambic (depending on presence of phenol red within DMEM) and centrifuged at 12,000 rcf for 30 min. Then, 50 µl of 25 mM Ambic were added and samples were left at room temperature for one hour; final spin at 3,000 rcf for 2 min to recover proteins. BCA.

Figure S6: *Giardia* supernatant protein profile and protein concentration after incubation in non-supplemented DMEM. Parasites were chilled on ice for 15 min, washed 3 times in pre-warmed PBS, centrifuged 10 min at 3,000 rpm between each wash; and then were incubated in pre-warmed non-supplemented DMEM for 45 min. at 37°C. After 45 min incubation, parasites were chilled on ice for 5 min and centrifuged 10 min at 3,000 rpm. Supernatants were collected and SDS-PAGE on 12% agarose gels, using a SYPRO staining, and BCA assay were performed – a representative gel is shown.

__Lane:__ __MW__: Molecular weight; **__2__**: TYI-S-33, 1:500 dilution; **__3__**: GS supernatant; **__4__**: WB supernatant; **__5__**: non-supplemented DMEM; **__6__**: TYI-S-33, 1:100 dilution.

Table 1:

Protein description	GI Number Assemblage A	GI Number Assemblage B	A:B Identity	dN/dS	Signal Peptide[b]	Protein Abundance: Pellet (P) iBAQ	Protein Abundance: Supernatant (SP) iBAQ	SP/P Ratio	Abundance ranking
PNPO	GL50803_5810	GL50581_4133	99.2	0.038	NP[c]	5.71E+07	1.18E+08	2.063091	1
Tenascin	GL50803_95162	GL50581_1982	76.2	**1.597**[a]	0.99	2.97E+07	4.77E+07	1.607024	2
Tenascin	GL50803_10330	GL50581_4057	73.5	0.347	0.99	4.66E+06	2.02E+07	4.342293	3
Cathepsin B	GL50803_16468	GL50581_438	83.6	0.1072	0.78	9.63E+06	1.79E+07	1.861309	4
Tenascin	GL50803_8687	GL50581_4316	77.6	**44.176**[a]	0.98	6.22E+06	1.10E+07	1.770526	5
Uncharacterised	GL50803_5258	GL50581_2767	91.2	0.029	NP[c]	3.93E+06	1.09E+07	2.780918	6
Extracellular nuclease	GL50803_8742	GL50581_3607	83.1	0.234	1	1.03E+06	4.57E+06	4.436193	7
Tenascin-37	GL50803_16477	GL50581_3575	79.8	0.1256	0.99	8.35E+05	4.03E+06	4.830956	8
Cathepsin B	GL50803_15564	GL50581_2036	79.1	**26.5782**[a]	1	1.10E+06	3.95E+06	3.589608	9
CKS1	GL50803_2661	GL50581_3484	100	0.001	NP[c]	1.14E+06	3.20E+06	2.803062	10
Tenascin	GL50803_113038	GL50581_4180	79	0.0949	1	1.20E+06	3.14E+06	2.620931	11
HCMP Group 1	GL50803_7715	GL50581_727	67	0.1821	0.99	ND[d]	2.54E+06	∞	12
Uncharacterised	GL50803_16522	GL50581_352	76	0.1591	NP[c]	1.15E+06	2.21E+06	1.928833	13
HCMP	GL50803_12063	GL50581_2622	83	0.246	1	3.62E+05	1.94E+06	5.354665	14
Cathepsin B	GL50803_17516	GL50581_2318	72.8	0.2056	1	ND[d]	7.81E+05	∞	15

[a] **dN/dS in bold indicate protein show evidence of positive selective pressure during divergence from a common ancestor**

[b] **Probability of N-terminal signal peptide using SignalP**

[c] **Not predicted**

[d] **Not detected**

Table 2:

				Protein Abundance							
				Assemblage A			Assemblage B			Abundance ranking in secretome	
**Protein description**	**GI Number Assemblage A**	**GI Number Assemblage B**	**Signal Peptide[a]**	**Pellet (P) iBAQ**	**Supernatant (SP) iBAQ**	**SP/P Ratio**	**Pellet (P) iBAQ**	**Supernatant (SP) iBAQ**	**SP/P Ratio**	**Assemblage A**	**Assemblage B**
A-type flavoprotein lateral candidate	*GL50803_10358*	*GL50581_1626*	*NP[c]*	*2.11E+07*	*3.70E+06*	*0.17546*	*4.35E+06*	*8.70E+06*	*2.001741*	*47*	*10*
Sentrin	*GL50803_7760*	*GL50581_3210*	*NP[c]*	*1.44E+05*	*ND[c]*	*ND[c]*	*ND[c]*	*3.67E+04*	∞	*-*	*31*
**EF-1α**	**GL50803_112312**	**-**	**NP[c]**	**ND[c]**	**2.22E+06**	∞	**-**	**-**	**-**	**17**	**-**
**ATP-binding cassette protein 5**	**GL50803_8227**	**GL50581_3399**	**NP[c]**	**2.93E+06**	**2.89E+06**	**0.98546**	**2.01E+05**	**1.43E+04**	**0.071052**	**15**	**897**
**CxC rich protein**	**GL50803_17476**	**GL50581_4509**	**1**	**4.80E+05**	**2.83E+05**	**0.58945**	**4.35E+04**	**3.59E+04**	**0.823376**	**43**	**819**
**Peptide methionine sulfoxide reducast MsrB**	**GL50803_5180**	**GL50581_3084**	**NP[c]**	**1.24E+05**	**8.80E+04**	**0.70952**	**1.09E+06**	**1.30E+06**	**1.19568**	**53**	**331**
**Translation initiation inhibitor**	**GL50803_480**	**GL50581_4017**	**NP[c]**	**9.47E+06**	**4.08E+06**	**0.43038**	**1.06E+07**	**9.76E+06**	**0.920148**	**15**	**95**

[a] **Probability of N-terminal signal peptide using SignalP**

[b] **Not predicted**

[c] **Not detected**

Figure 1

Click here to download Figure Figure 1.tif

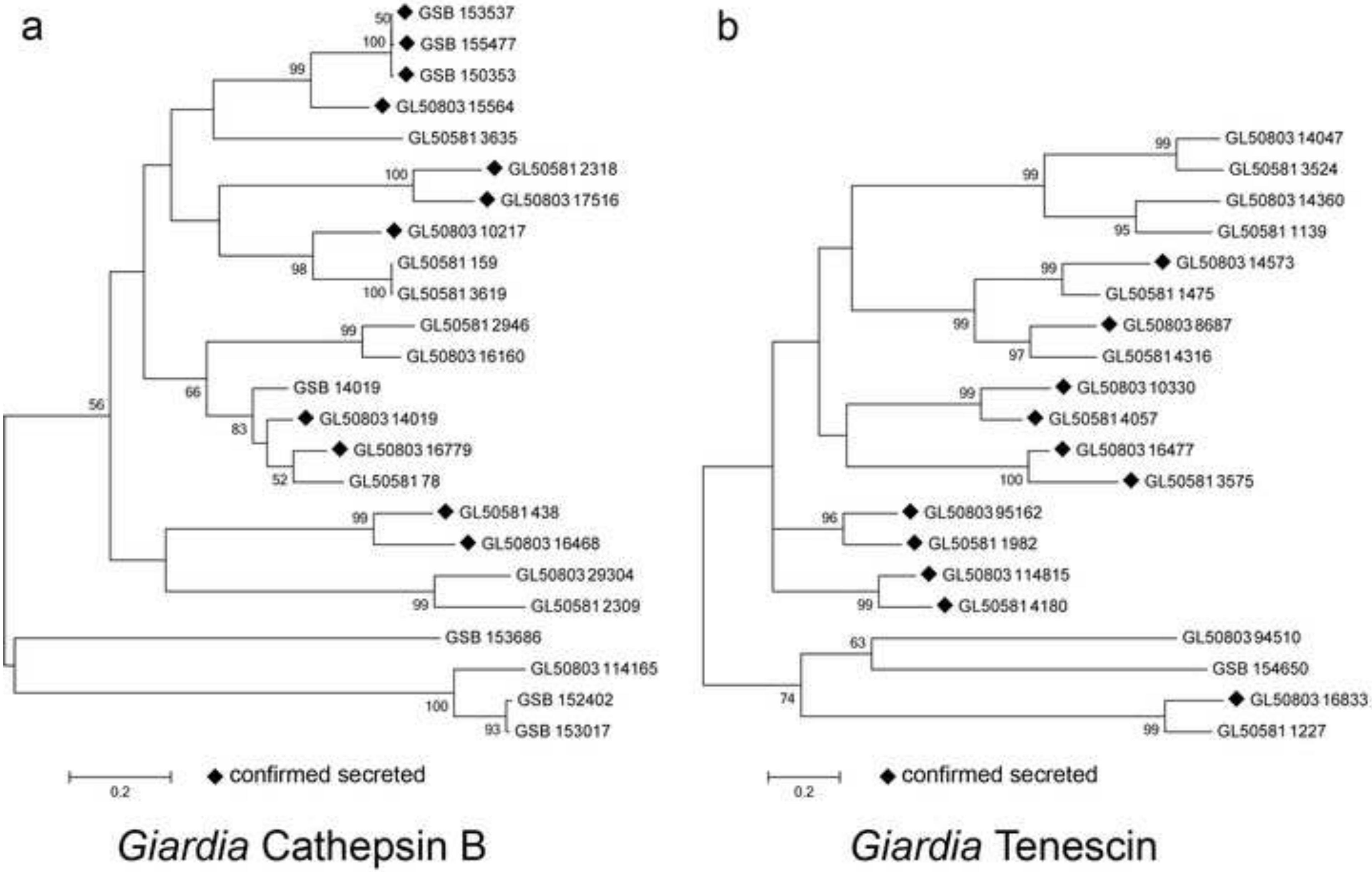

Figure 2

Click here to download Figure Figure 2.tif

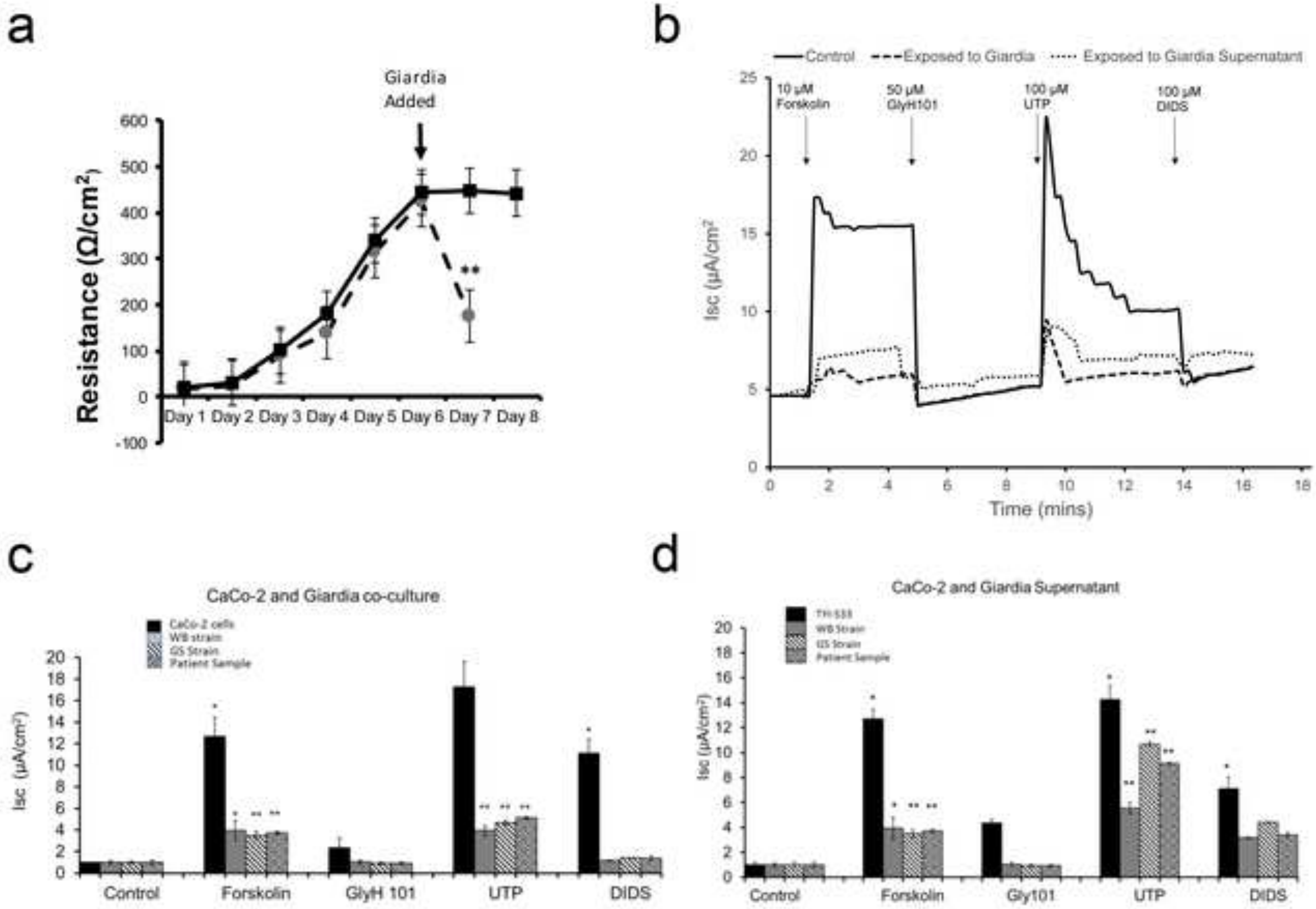

Click here to download Figure Figure 3.tif

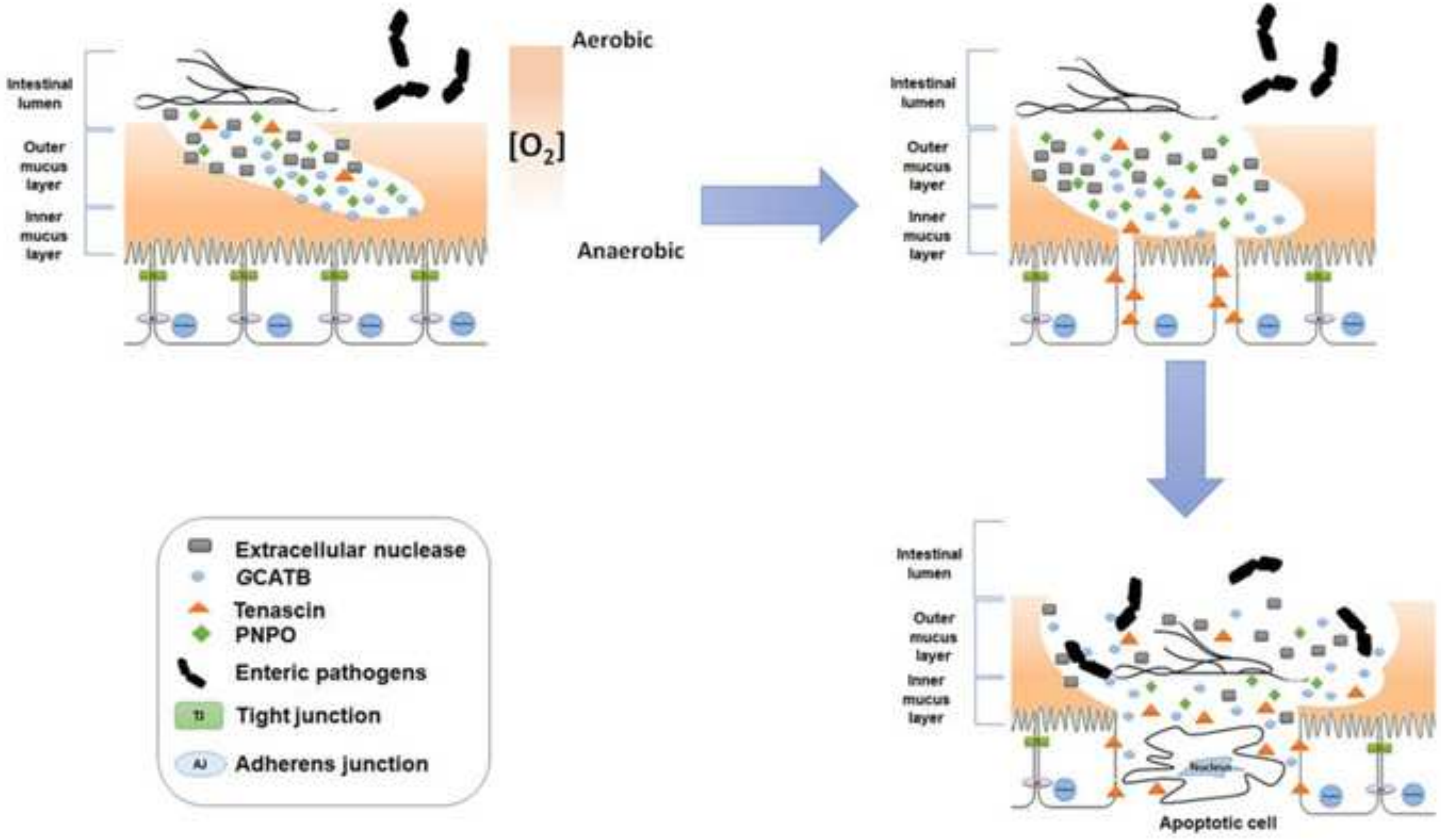

Mechanism of Pathogenesis

Figure S1

Click here to access/download
Supplementary Material
Additional Figure S1.pdf

Figure S2

Click here to access/download
Supplementary Material
Additional File Figure S2.pdf

Figure S3

Click here to access/download
Supplementary Material
Additional File Figure S3.pdf

Figure S4

Click here to access/download
Supplementary Material
Additional File Figure S4.pdf

Figure S5

Click here to access/download
Supplementary Material
Additional file Figure S5.pdf

Figure S6

Click here to access/download

Supplementary Material

Additional file Figure S6.docx

Table S1

Click here to access/download
Supplementary Material
Additional file Table S1 Amended.docx

Table S2

Click here to access/download
Supplementary Material
Additional Table S2 amended.docx

Table S3

Click here to access/download
Supplementary Material
Additional file Table S3 amended.docx

Table S4

Click here to access/download
Supplementary Material
Additional file Table S4 amended.docx

Table S5

Click here to access/download
Supplementary Material
Additional file Table S5.docx

Table S6

Click here to access/download
Supplementary Material
Additional file Table S6.docx

Table S7

Click here to access/download
Supplementary Material
Additional file Table S7.docx

Table S8

Click here to access/download
Supplementary Material
Additional file Table S8.docx

Click here to download Personal Cover Minor Improvements from peer review 2017Dec04.docx

Responses to the Reviewers and Editor's comments Regarding: "*Giardia* Secretome Highlights Secreted Tenascins as a Key Component of Pathogenesis", GIGA-D-17-00132

Summary:

Thank you to you and your reviewers for these positive and constructive we have addressed them all as fully as we are able to do so.

Response to Reviewer#1: (Text of Reviewer with authors' responses in *italic)*

I would like to congratulate the authors for their extensive revisions. I think this has greatly improved the manuscript and facilitated understanding of their methods. I have a few comments and revisions that I feel the authors should consider prior to final publication.

Firstly, I understand that the reviewers are using both MS platforms (Q-Exactive and LTQ Velos) to increase robustness of their data. I do not think they increase coverage, as they state they are highly correlated, and nearly all of the Velos IDs are also identified by the Q-exactive MS (which is more sensitive and makes sense). However, given there is little discrepancy between the data from both platforms, that the data is already filtered by reproducibility, and the Q-exactive is the more sensitive machine, might the authors think to keep the extra, reproducible IDs from the Q-exactive rather than discarding them totally because they are not seen on the LTQ Velos? As secreted proteins are going to be low abundance (given they are only being produced and secreted over an hour or so incubation in DMEM), the sensitivity of the Q-exactive may provide additional secreted proteins? Maybe these can at least be provided in an additional supplementary table?

Thank you for these very insightful statements and suggestions. Although we agree that the proteins identified only by Q-Exactive should not be completely excluded from the analysis in this work we would prefer to only include the proteins identified by both MS techniques to keep the robustness of our data. However, we do also agree that including the proteins identified as most likely secreted only by Q-Exactive MS may also bring additional information to the reader; therefore; those proteins were added in supplementary Table S3 and S4 in italic to differentiate them from the proteins included in the analysis.

Secondly, given the authors are comparing WB and GS, and that these most likely represent cryptic species with different clinical pathophysiology, maybe the discussion/results could be added to in order discuss differences between assemblages. The authors have already provided a robust set of proteins common between assemblages, and tables of proteins identified exclusively in WB or GS. However, significant differences between assemblages in the proteomics and/or electrophysiology could provide important insights into the differences between these two, human-infective assemblages that is limited in current literature.

This part was indeed missing, thank you for pointing it out. Not many differences in terms of protein identification were found in the dataset but a few proteins had a great variation in their abundance from one assemblage to the other. Those proteins were put in a table (Table 2) and paragraphs were added in the results section and discussion sections.

Thirdly, while the authors do plan to deposit the data in the EuPath DBs, providing excel supplementary tables of protein IDs, peptide counts and IBAQ values for reproducibly identified

proteins is a very valuable resource for other researchers looking to compare identifications and expression values. Some of these people may not be familiar or aware of the EuPATH databases and their systems. I strongly advise providing these for future reviewers, as it will greatly affect the accessibility of your data.

We are happy to provide this and have added 4 supplementary tables: Table S5-S8 containing all the proteins identified peptide counts and iBAQ values for each technique and by sample< For example Table S5 represents the list of proteins identified in the pellet of GS strain trophozoites for both Q-Exactive and Orbitrap MS.

Minor Comments:

Methods:

1) In the non-supplemented DMEM, what was the oxygen levels? Were these capped, filled flasks?

Parasites were incubated for 45 min in 1 ml of non-supplemented DMEM under their standard in vitro growth conditions. Pellets were resuspended in DMEM and transferred in filtered glass tube and put at 37°C with 5% O_2. To help readers and reviewers understand the protocol, a supplemental figure was created and added with the whole protocol developed and used to cleanse and harvest pellets and supernatants: Figure S5. Reference to figure was added in the Method section under Proteomic analysis, Samples preparation. In addition, culture protocols are now provided.

2) What were the centrifugation speeds - higher speeds would have been likely to rupture cells, so it might be good to report this.

The centrifugation speed to separate pellet and supernatant was the speed used to usually pellet down Giardia "safely": 3,000 rpm. This speed is the standard speed used to pellet down the parasites before freezing cells down and is unlikely to rupture cells. However, as we incubated the, in DMEM before harvesting pellets and supernatants, we performed a flow cytometry viability assay on pellet samples after centrifugation. The parasites were still alive as shown by supplemental Figure S1. But to be sure readers have all the key elements, all the centrifugation speeds used were added onto Figure S5.

3)In the electrophysiology, what are the patient strains? Are these verified assemblage A or B? Have they been typed?

The patient strain used in the electrophysiology was not typed. It was a stabilate prepared from a clinical sample at the Norfolk and Norwich University Hospital (NNUH) and was used alongside the lab strains to control for atypical activity (e.g. attenuation) that might have occurred as an artefact of protracted culture of the laboratory strains. Since the results were similar to the laboratory strains it serves to provide support that the results are likely to be generalizable for most human giardia infections.

Results:

1) I think that adding some further comments about viability and harvesting contingencies to ensure cell integrity (i.e. cell not popping) would be valuable when reporting supernatant vs pellet IDs around pg 12, ln 42-49. While contamination of the supernatant with proteins is a very difficult thing to avoid in this type of experiment, hence your experimental design in averaging pellet to supernatant ratios, I think showcasing steps to mitigate contamination would add more credibility to the results.

To help the reader visualise what was done to obtain the proteomic samples, a supplementary figure: Fig S5 was created and added as well as its legend explaining every step of the protocol

2) Were the supernatants harvested with protease inhibitors at any stage, or how was protease activity controlled during harvesting? SDS-PAGE was mentioned as a quality control step (which can also show whether there is any evidence of protease activity), but pictures of these gels are not provided in the supplementary.

No protease was added in the final cleansing protocol. Several protein concentration protocols were tried before deciding to use the Vivaspin columns and we followed the protocol from the manufacturer which did not require the use of proteases. SDS-PAGE were run after the protein concentration to verify that proteins were not degraded, and BCA assay performed to obtain the protein concentration as well. A supplementary figure: Fig S6 is provided with a photo of a representative SDS-PAGE gel.
