# Supplementary material for: Giardia secretome highlights secreted tenascins as a key component of pathogenesis
Source: Gigascience. 2018 Jan 29;7(3):1–13. doi: 10.1093/gigascience/giy003 (PMC5887430; doi:10.1093/gigascience/giy003)

Figure S5:

A. Preparation of *Giardia* samples for Proteomic assay

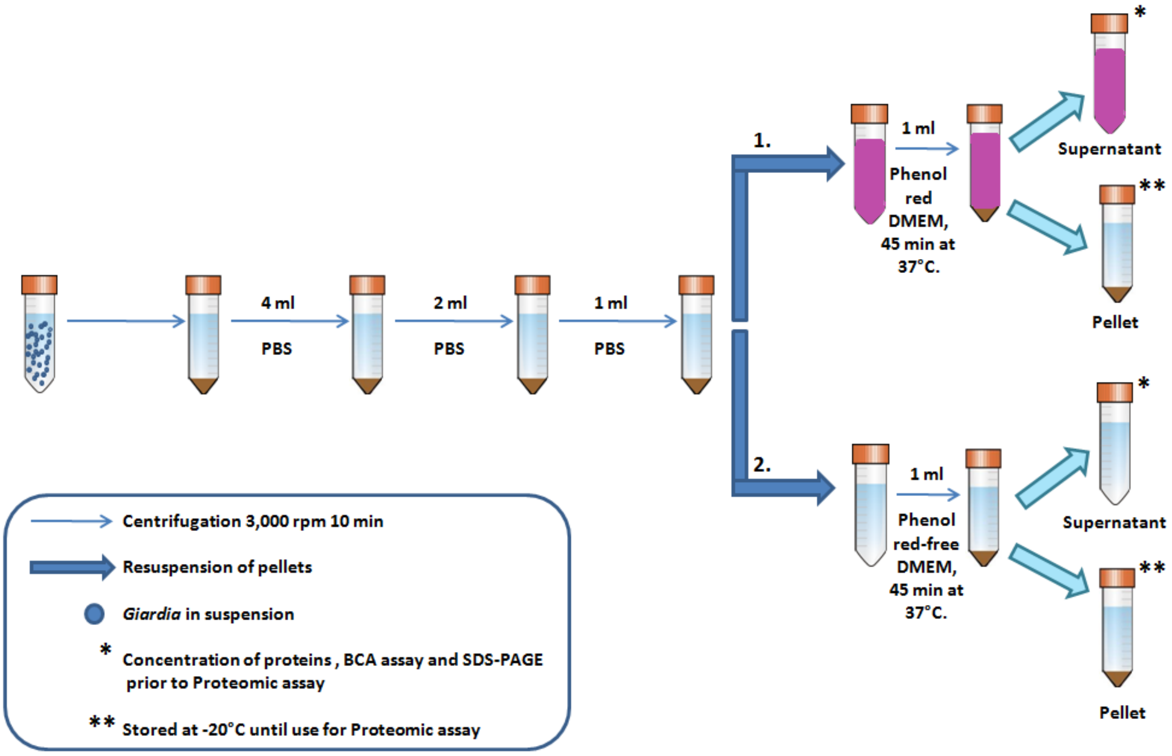

B. Protein concentration protocol for *Giardia* supernatant samples

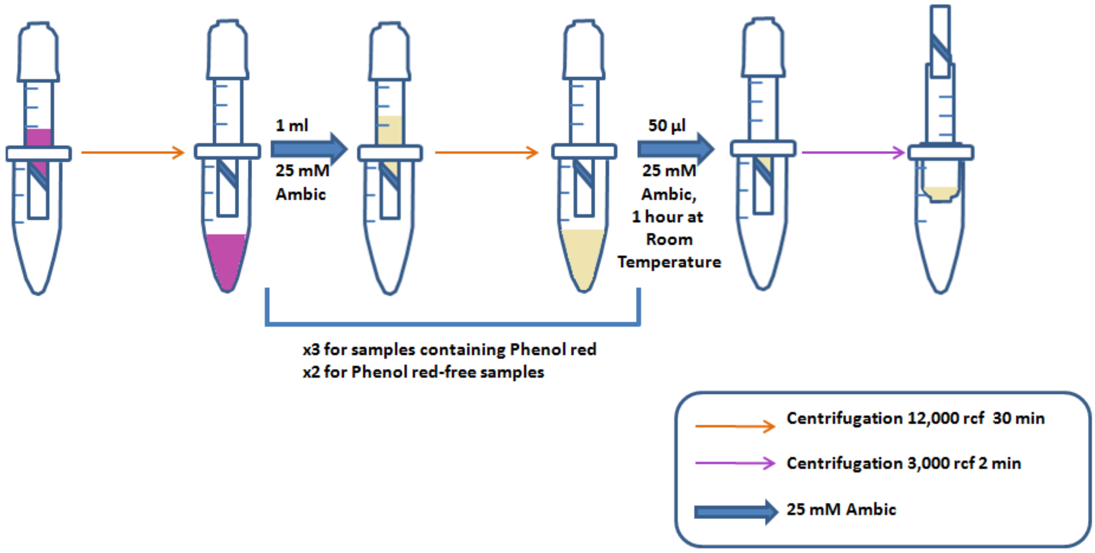

Supplement: Additional Files [file giy003_supp.zip › Additional file Figure S5.pdf]
