# Supplementary material for: Giardia secretome highlights secreted tenascins as a key component of pathogenesis
Source: Gigascience. 2018 Jan 29;7(3):1–13. doi: 10.1093/gigascience/giy003 (PMC5887430; doi:10.1093/gigascience/giy003)
Supplement: Additional Files [file giy003_supp.zip › Additional file Table S6.docx]

**
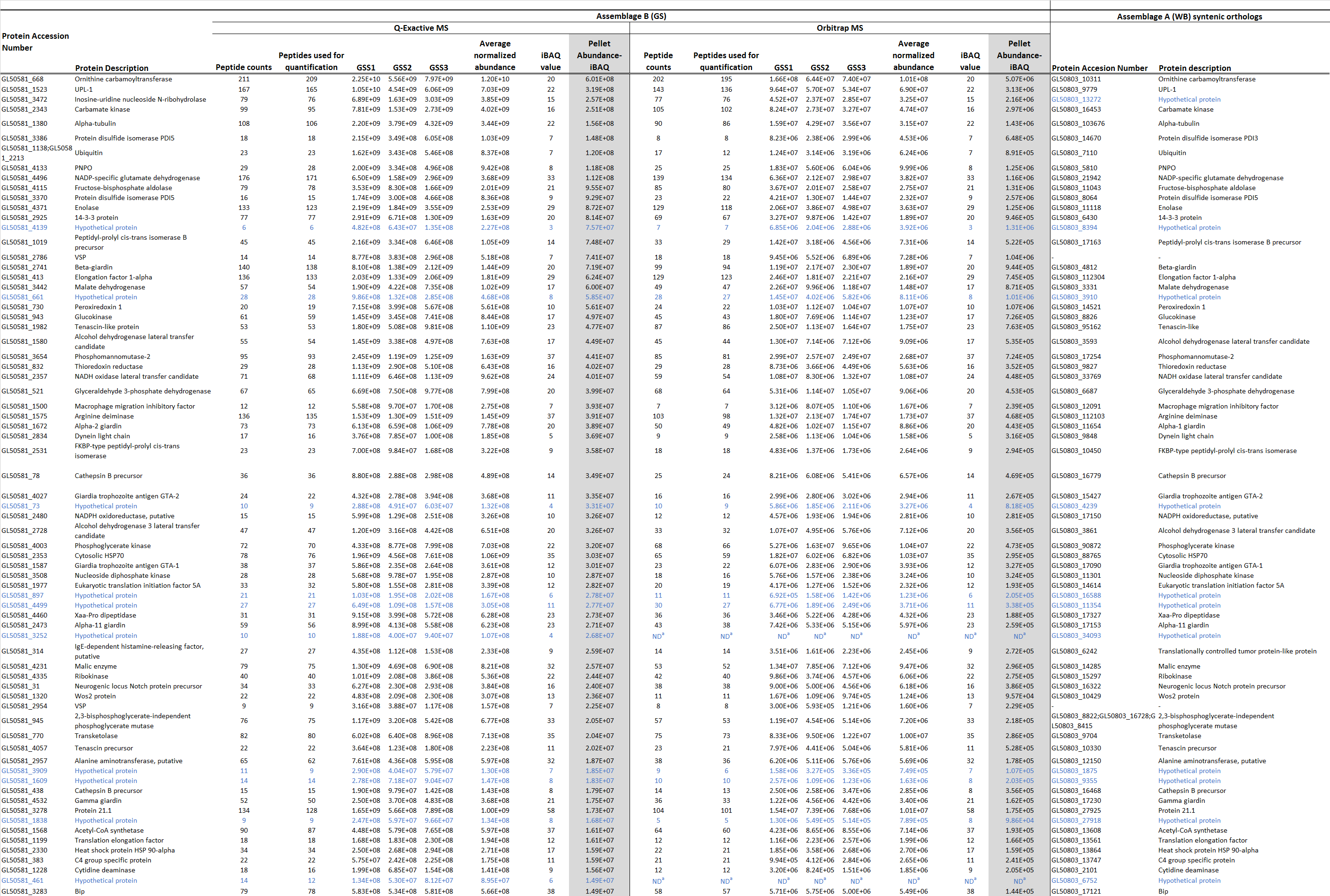
Table S6:**

**
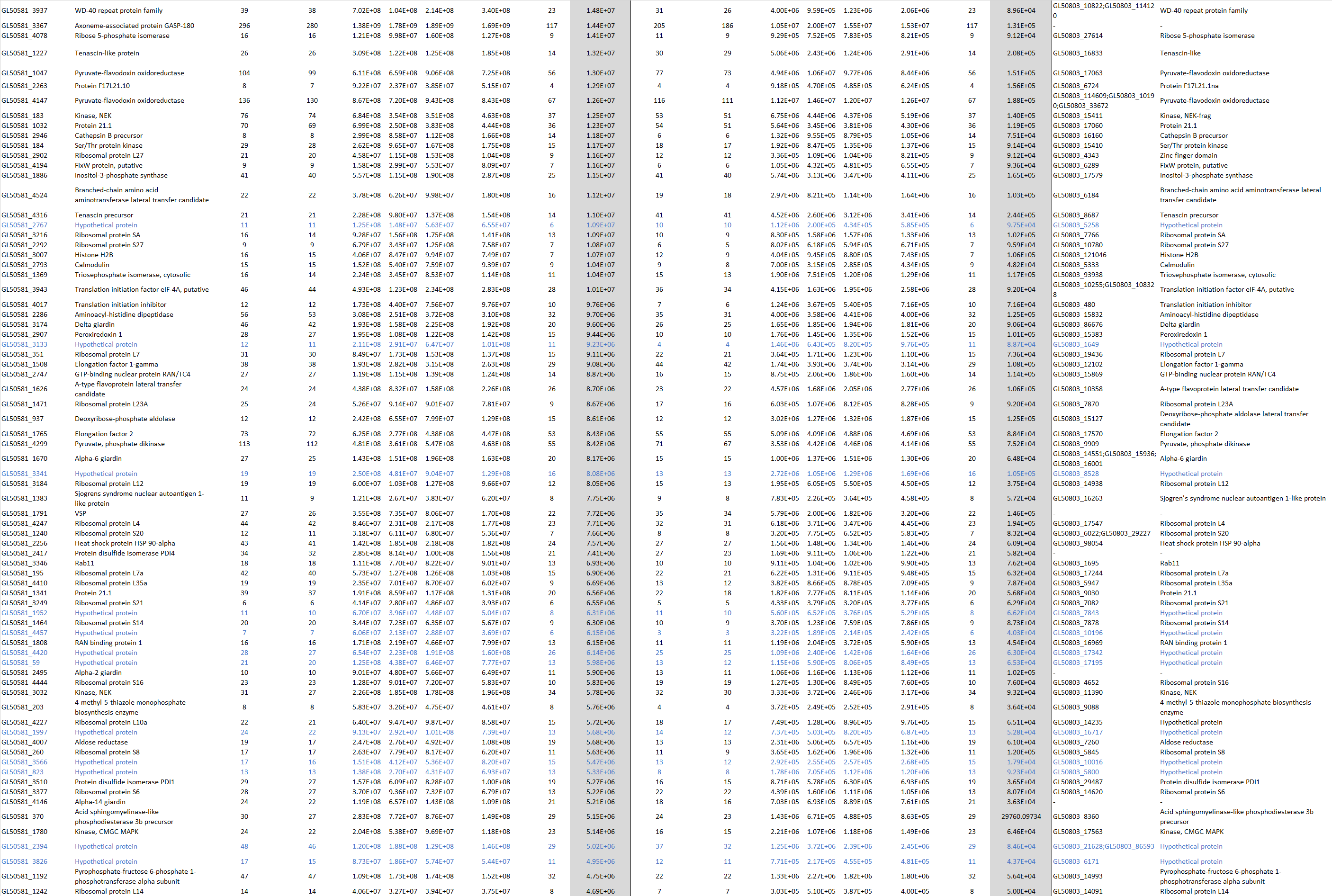
Table S6 (Cont.):**

**
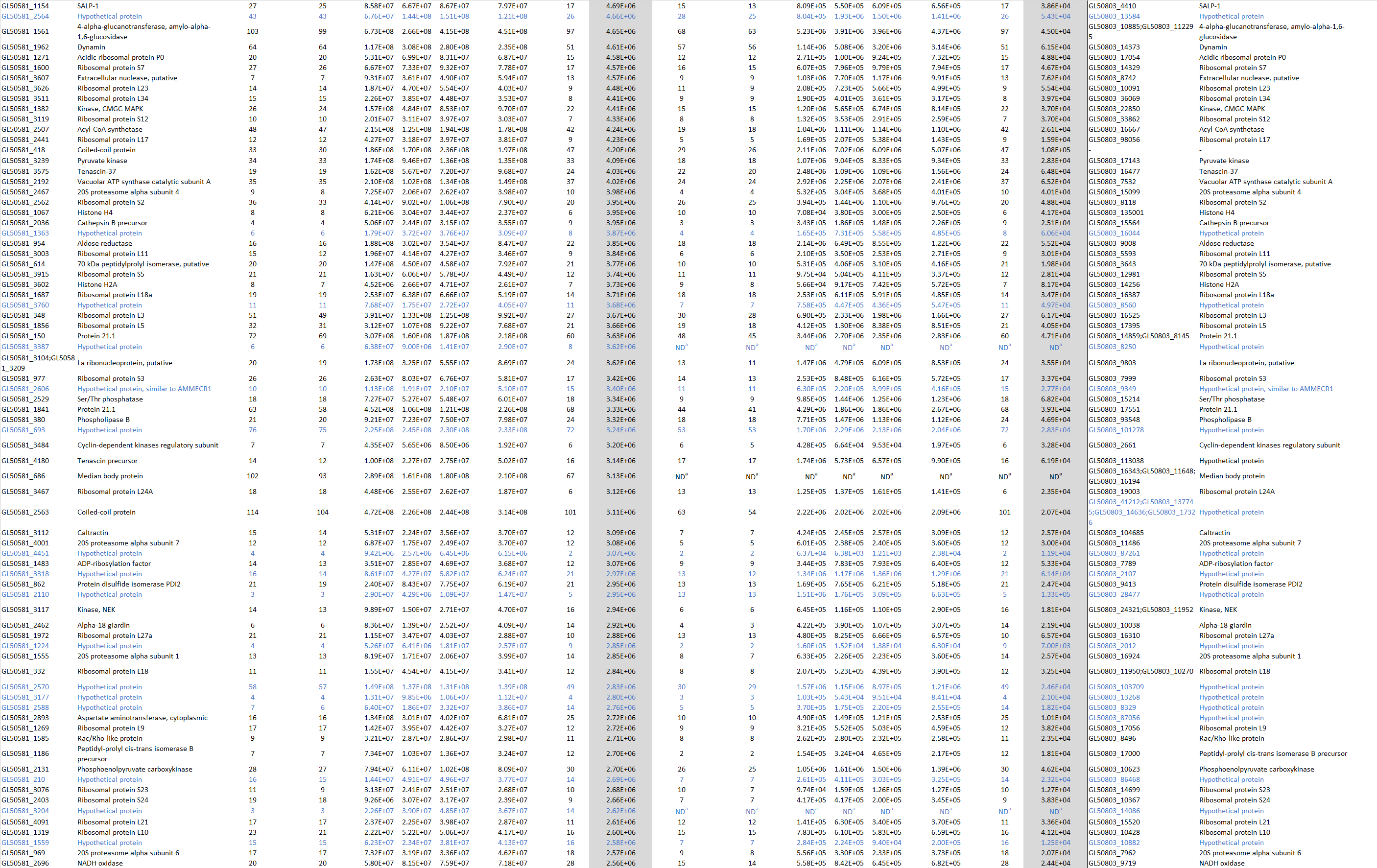
Table S6 (Cont.):**

**
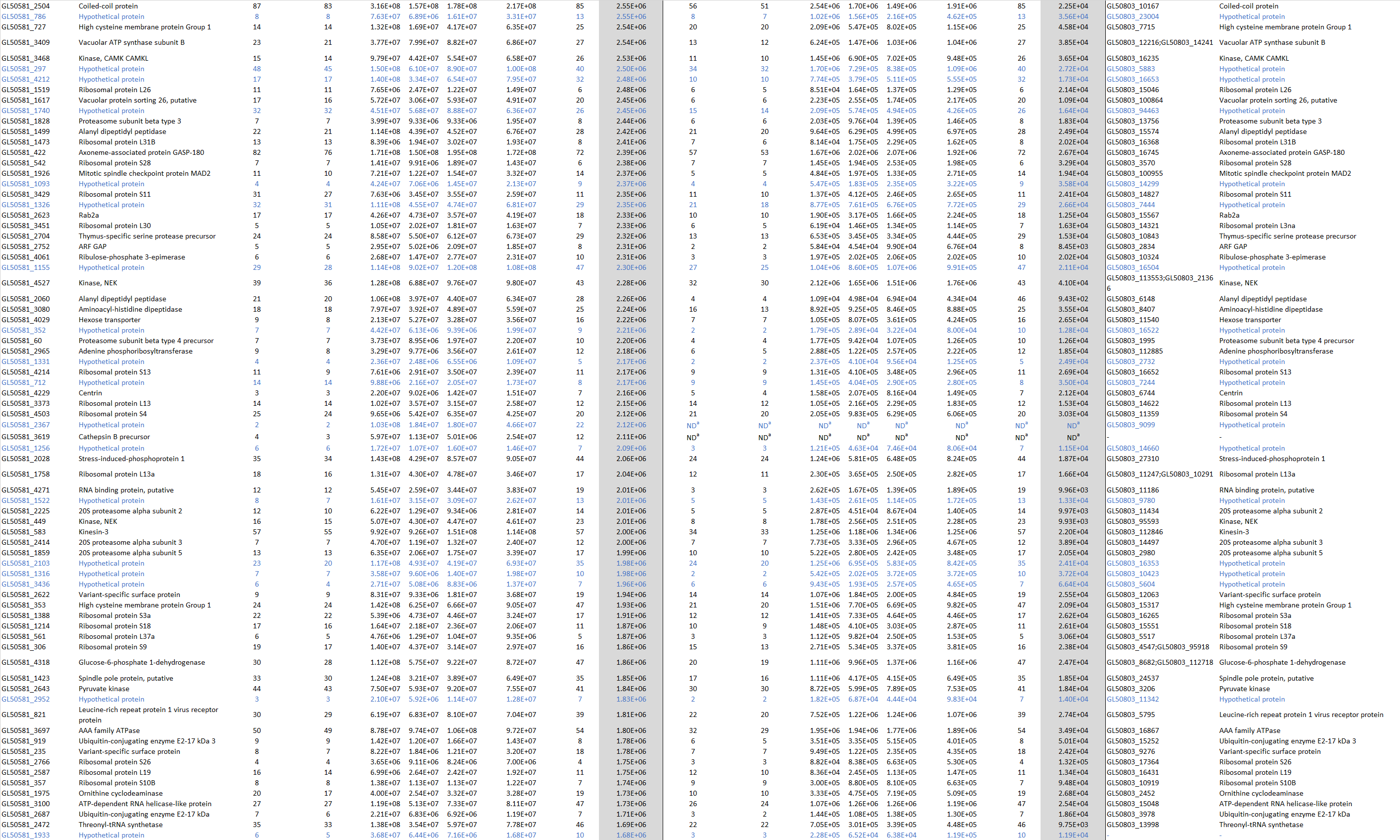
Table S6 (Cont.):**

**
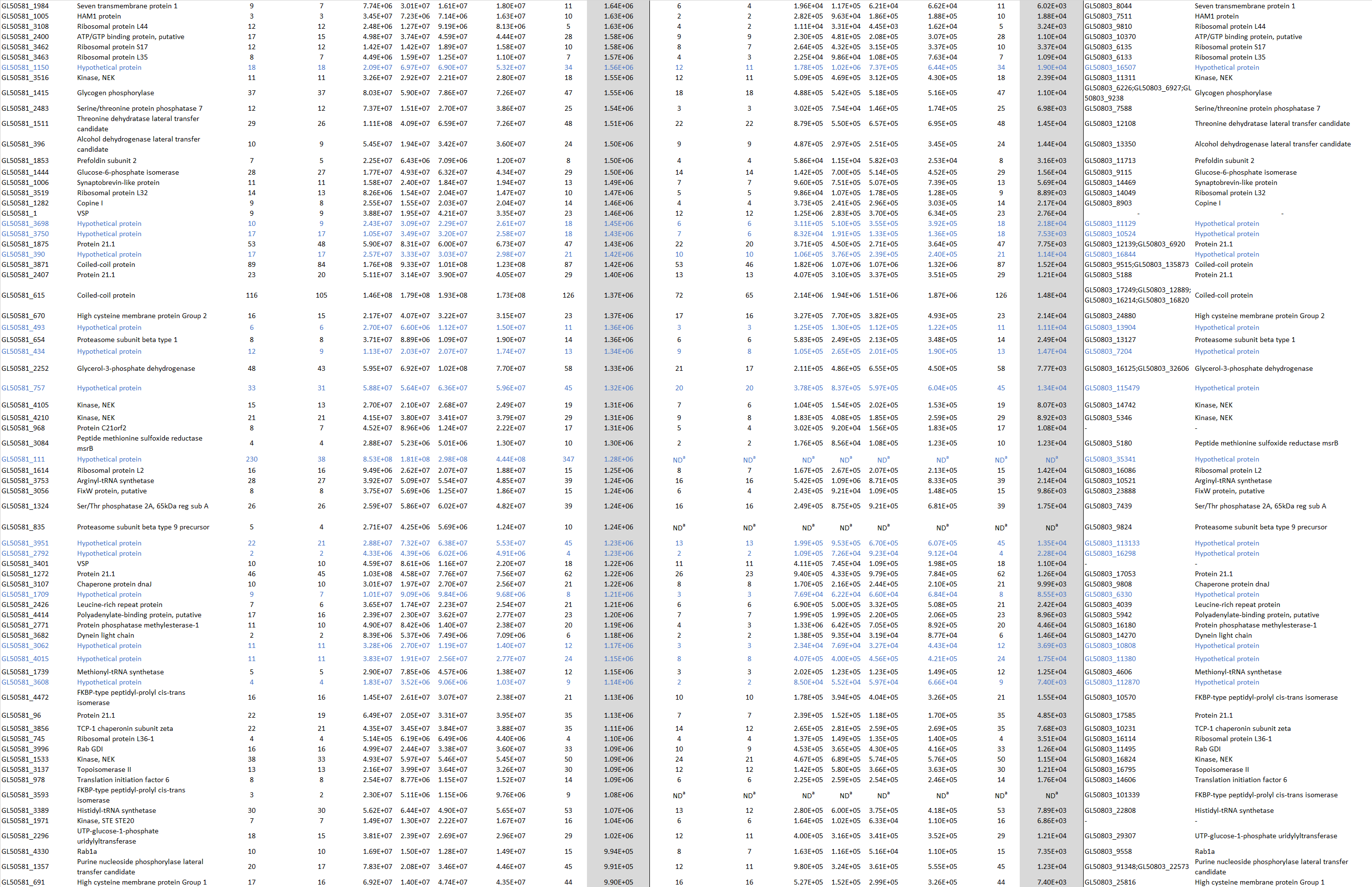
Table S6 (Cont.):**

**
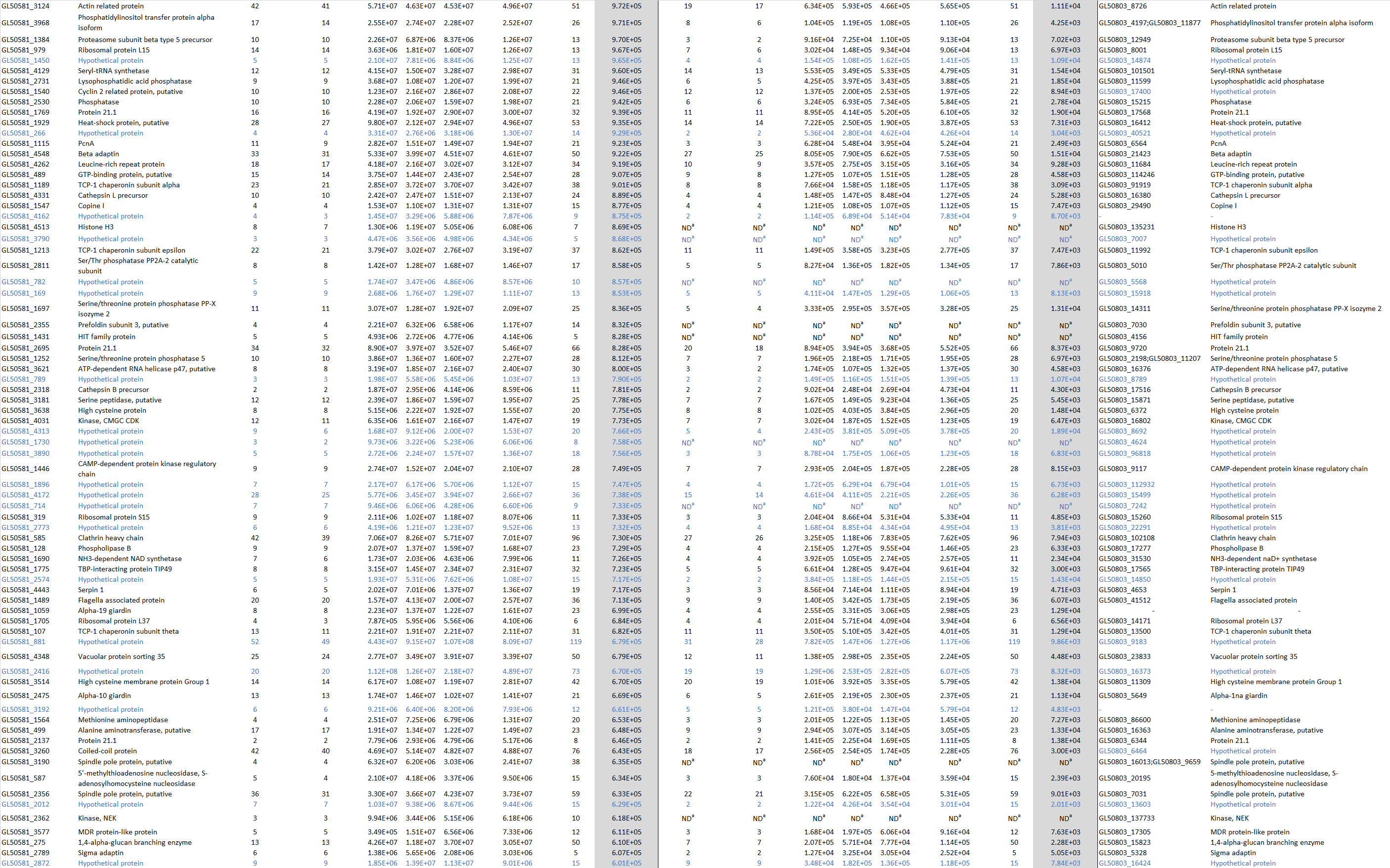
Table S6 (Cont.):**

**
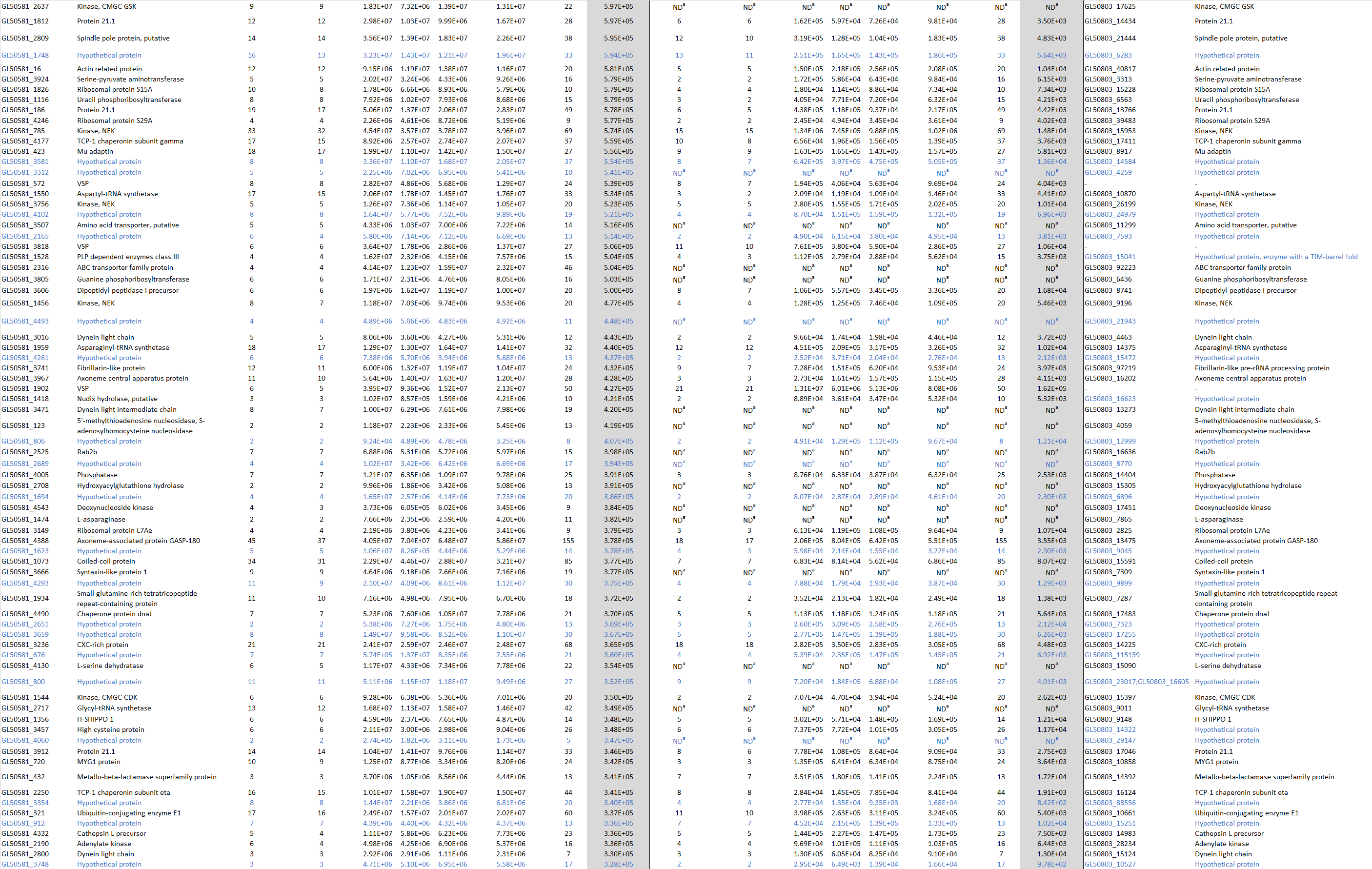
Table S6 (Cont.):**

**
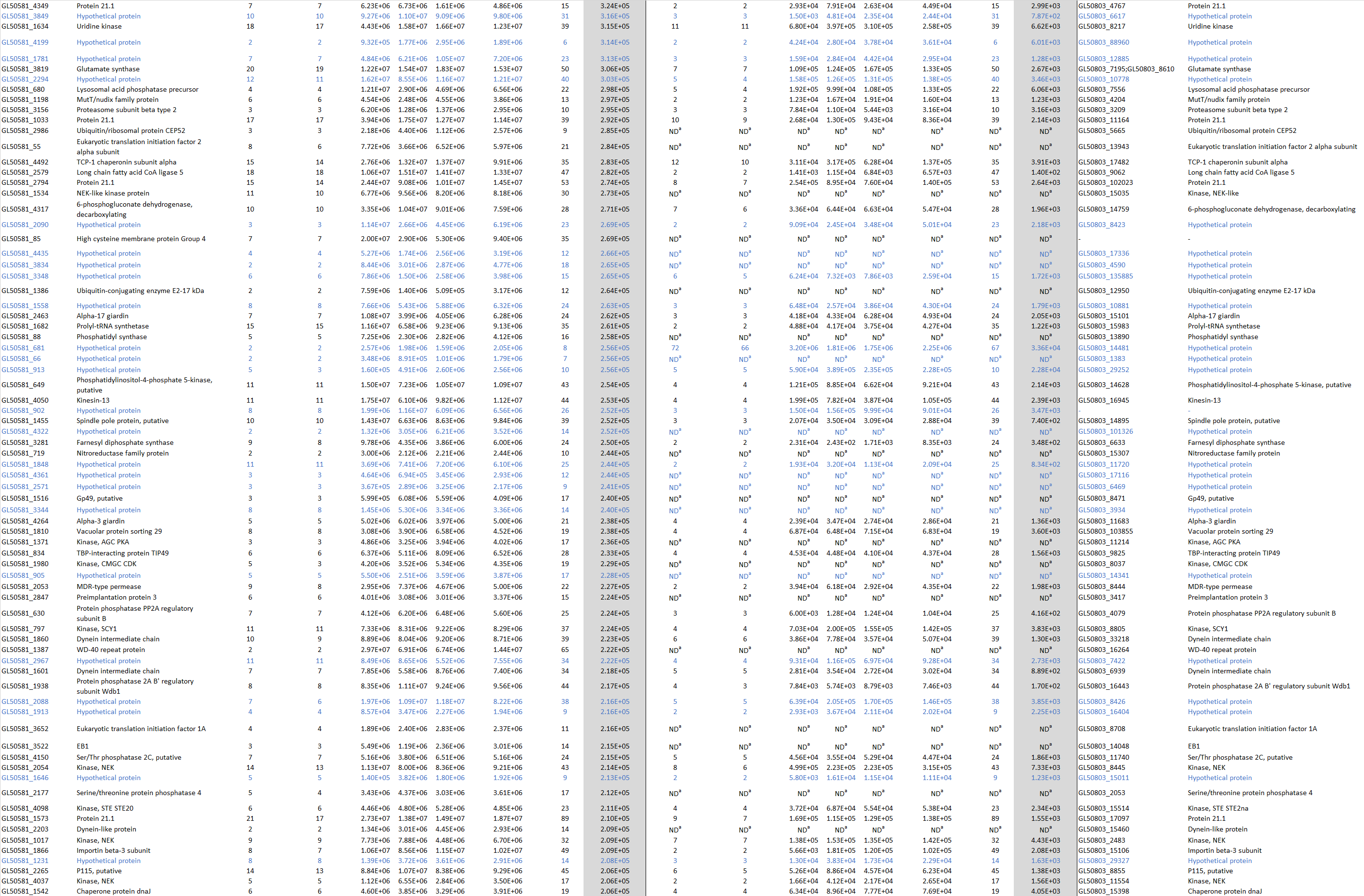
Table S6 (Cont.):**

**
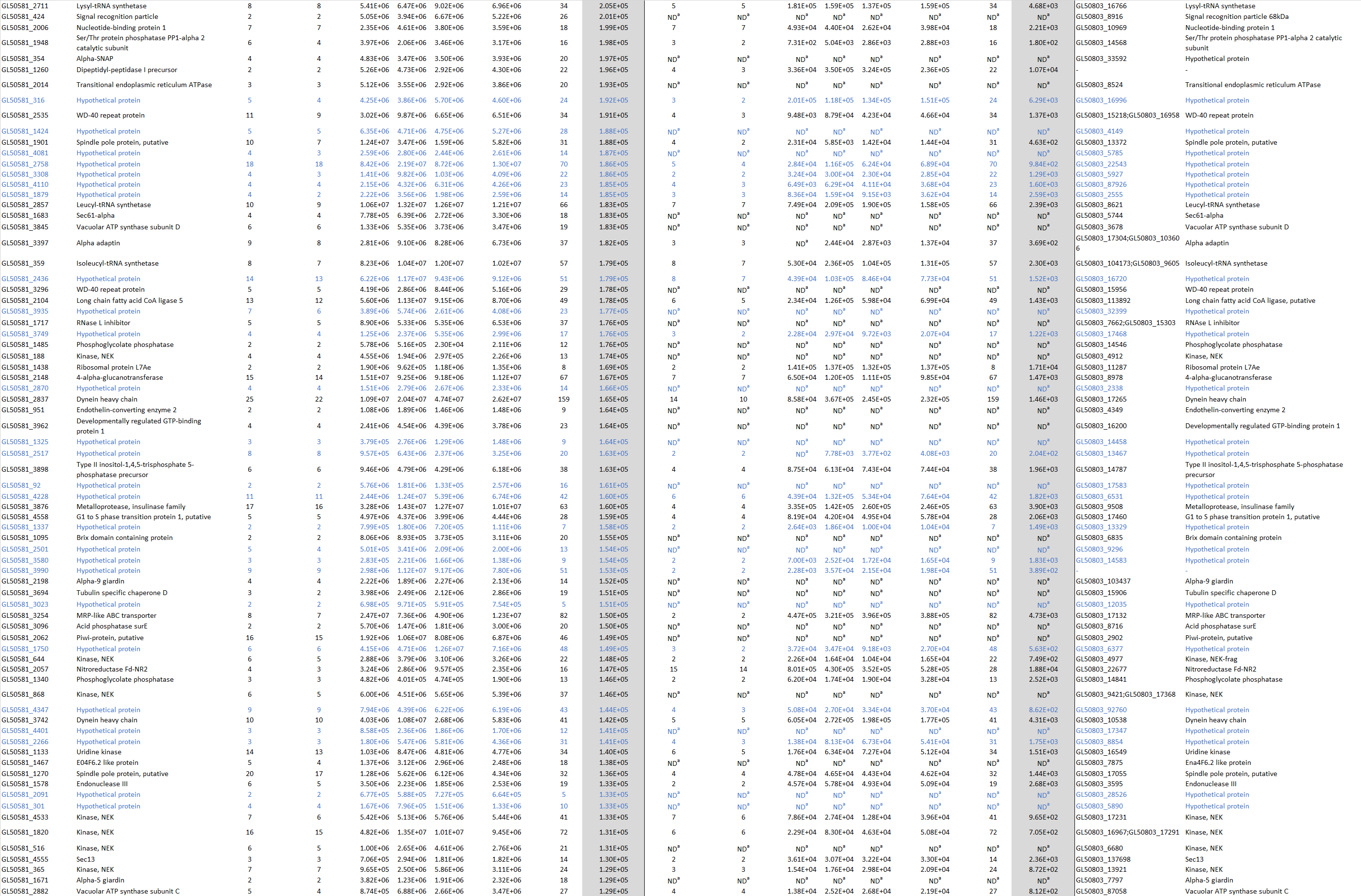
Table S6 (Cont.):**

**
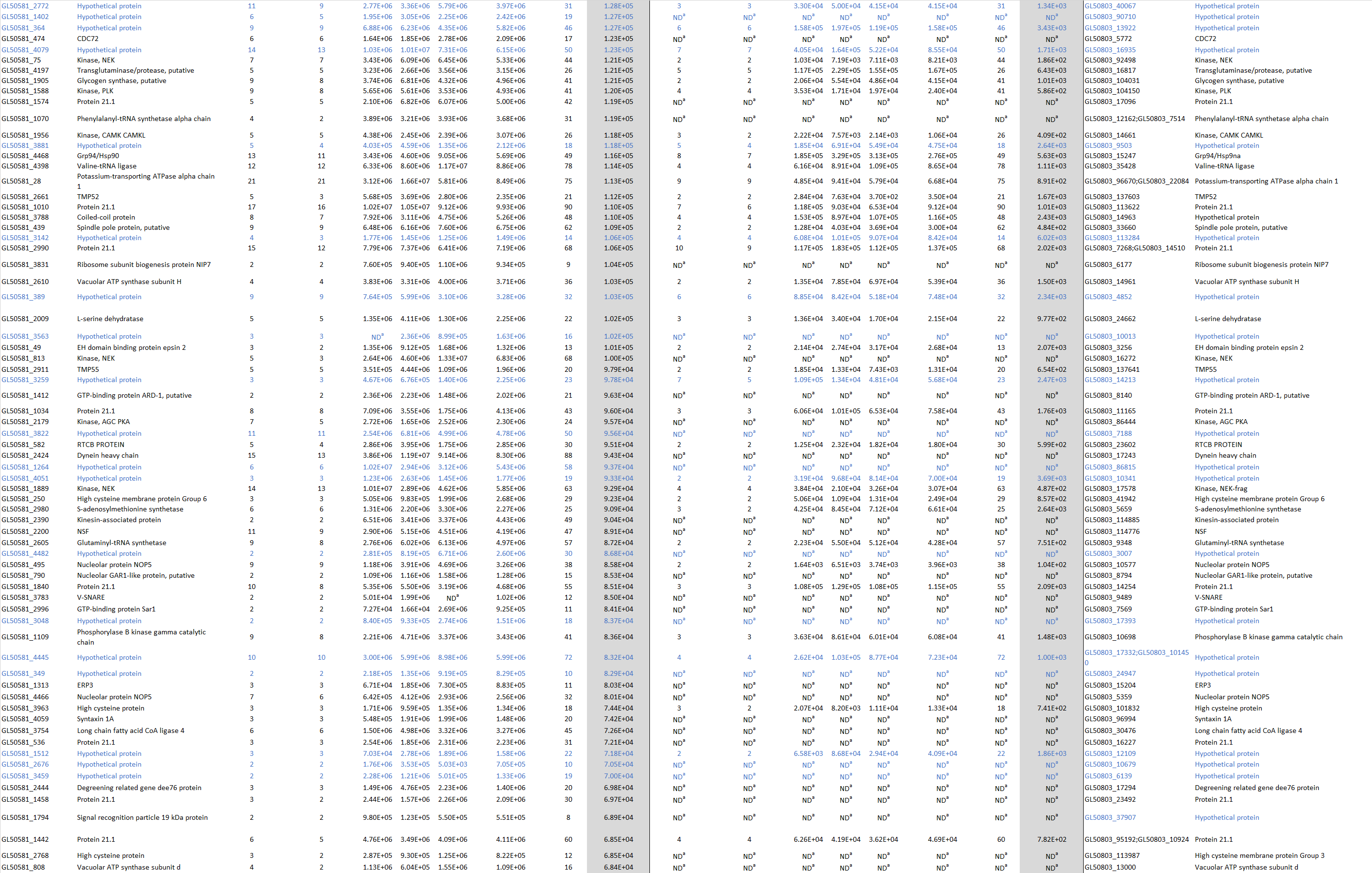
Table S6 (Cont.):**

**
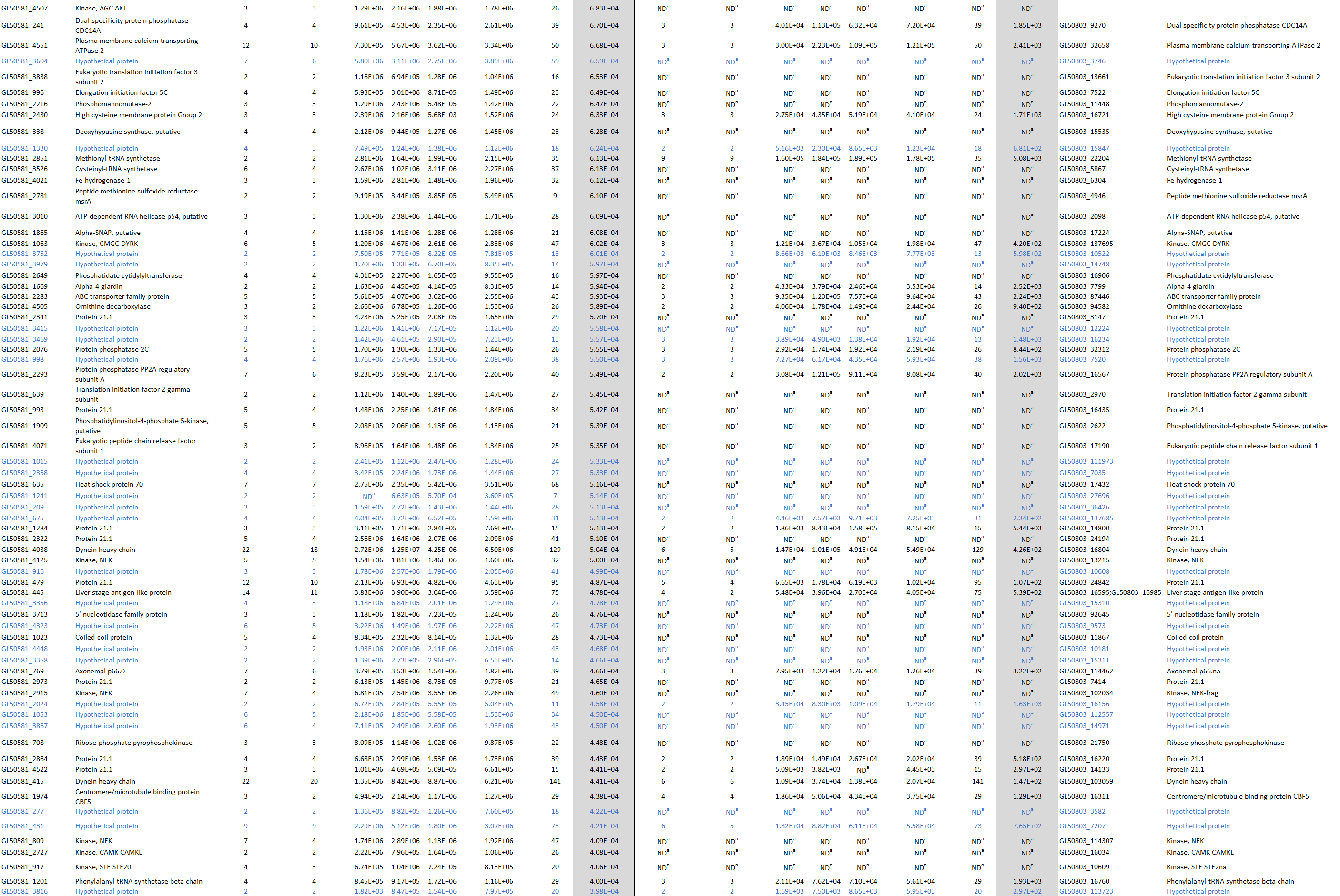
Table S6 (Cont.):**

**
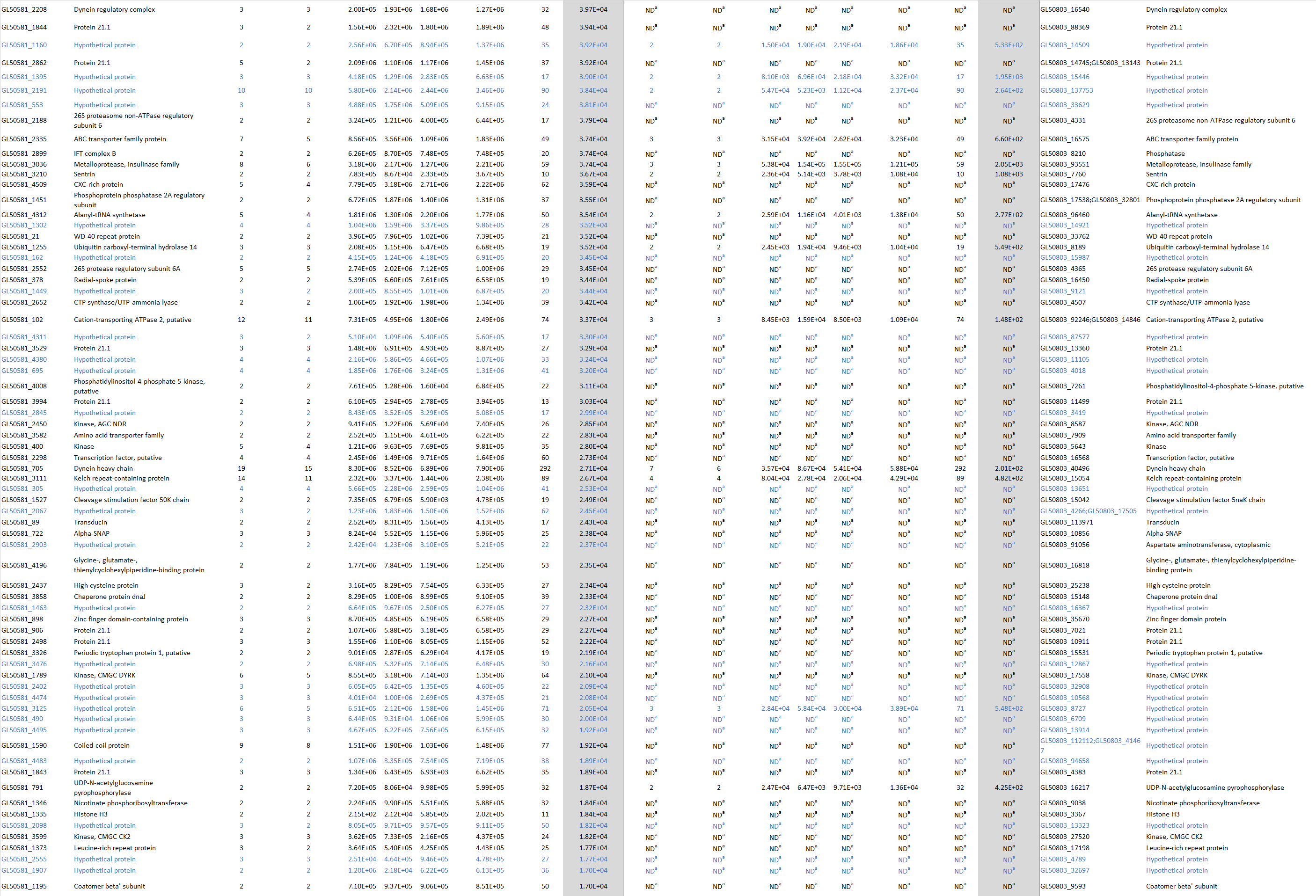
Table S6 (Cont.):**

**
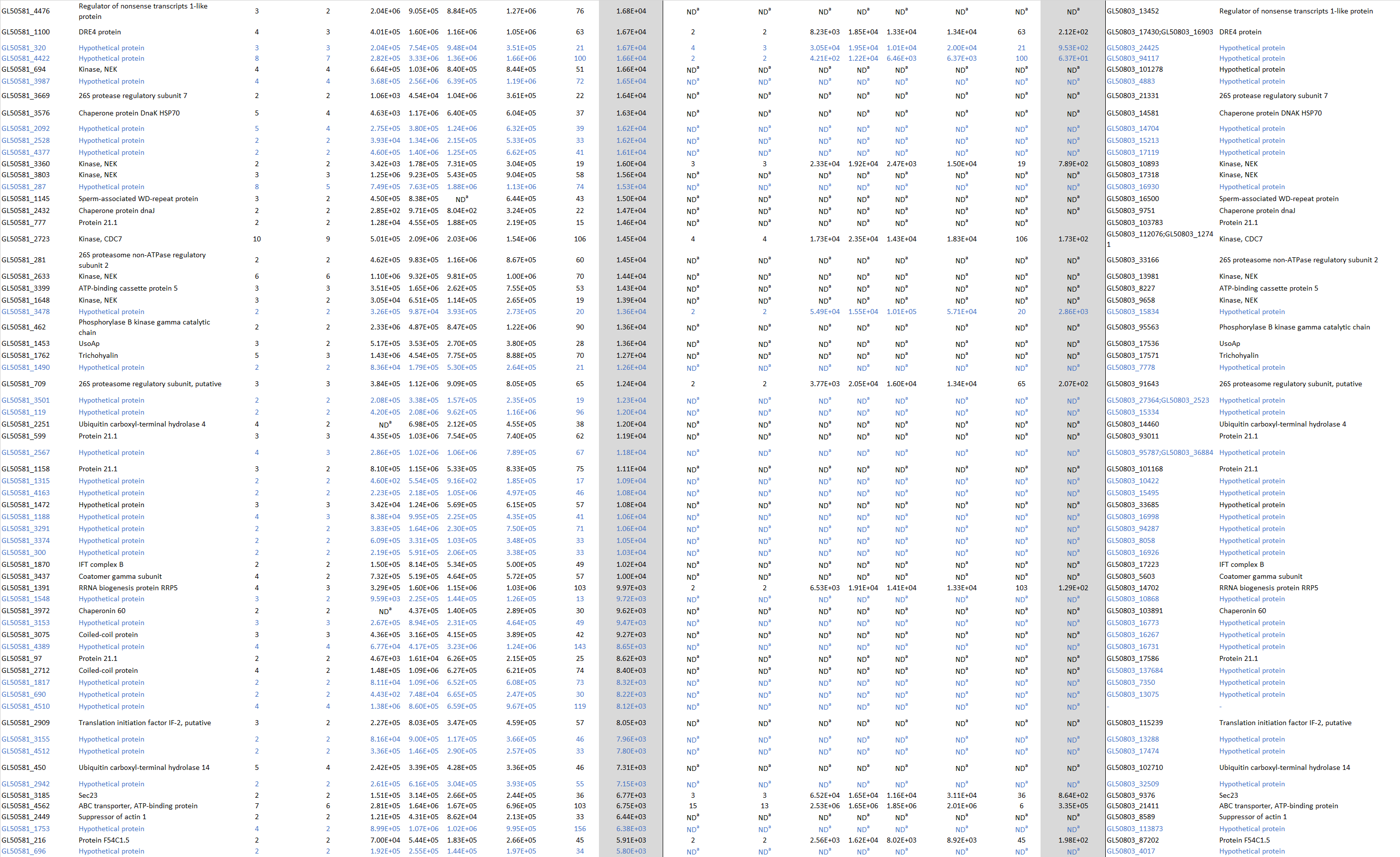
Table S6 (Cont.):**

**
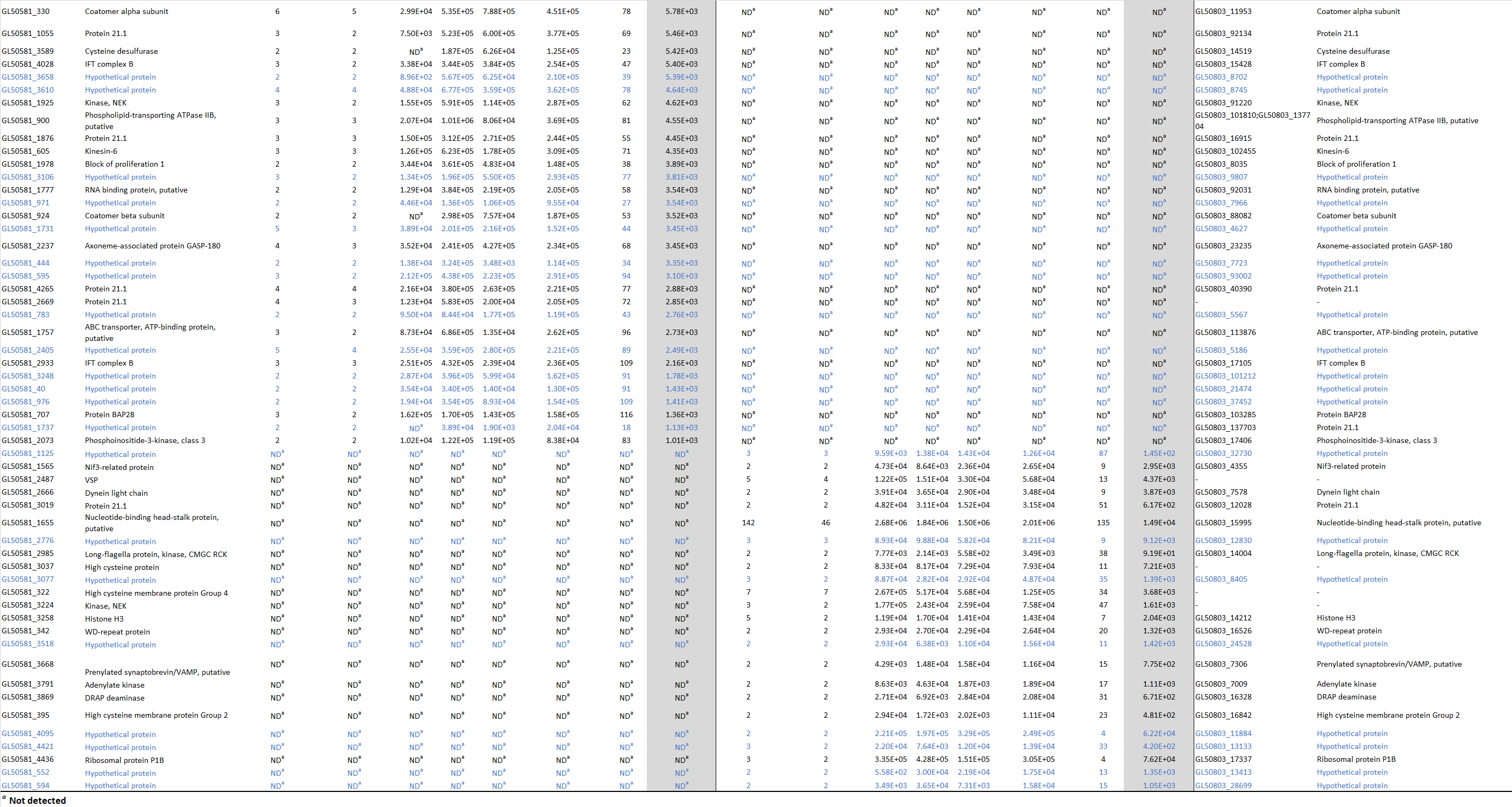
Table S6 (End):**
