# Supplementary figures and images for: Giardia secretome highlights secreted tenascins as a key component of pathogenesis
Source: Gigascience. 2018 Jan 29;7(3):1–13. doi: 10.1093/gigascience/giy003 (PMC5887430; doi:10.1093/gigascience/giy003)

### Figure S1

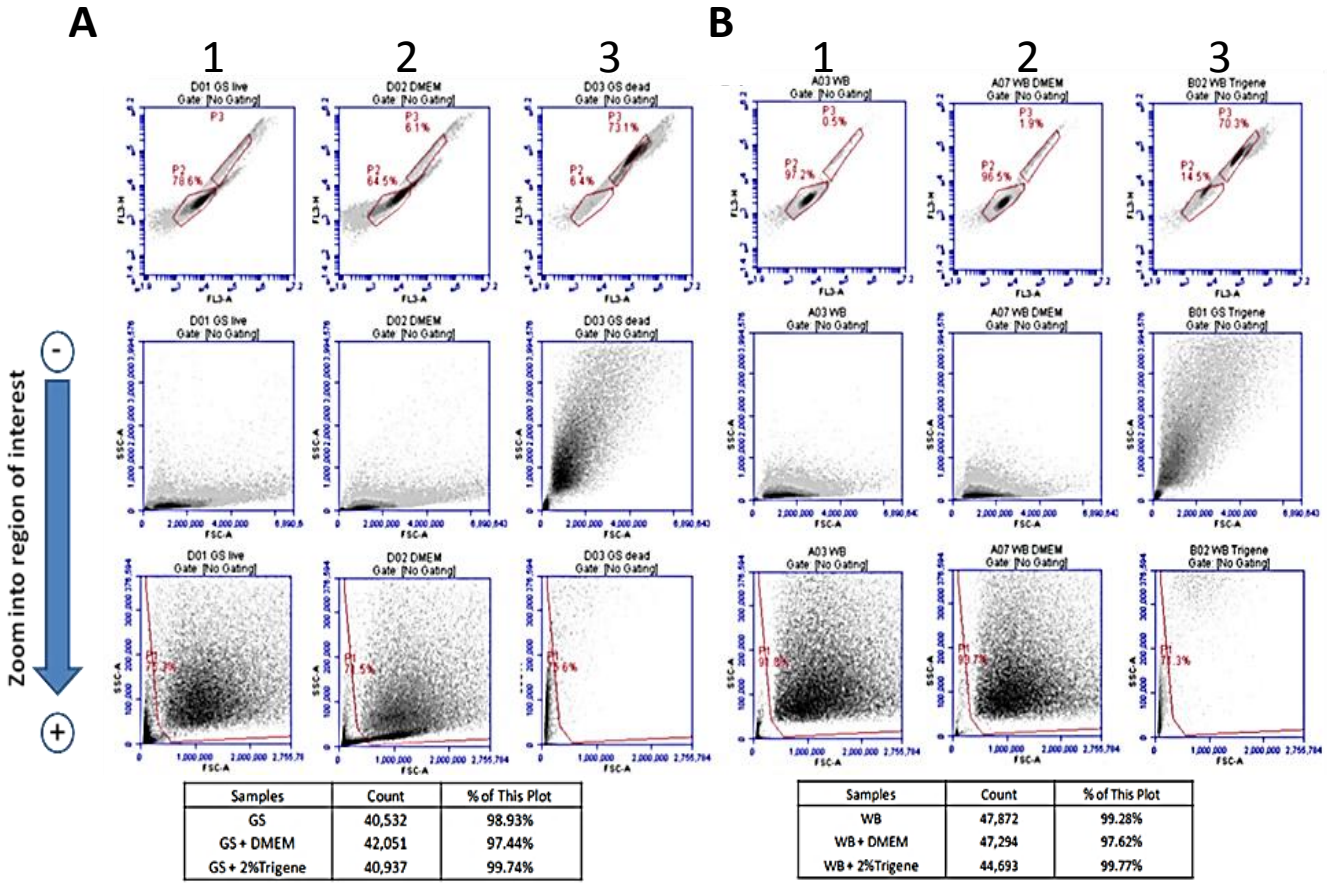

Supplement: Additional Files [file giy003_supp.zip › Additional Figure S1.pdf]

Figure S2

A. Assemblage B (GS)

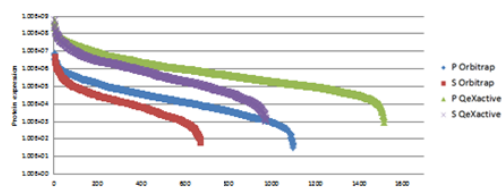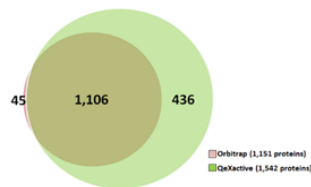

B. Assemblage A (WB)

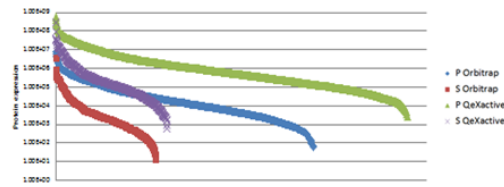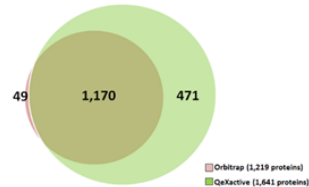

Supplement: Additional Files [file giy003_supp.zip › Additional File Figure S2.pdf]

Figure S3

A. Assemblage B (GS)

*Orbitrap MS*

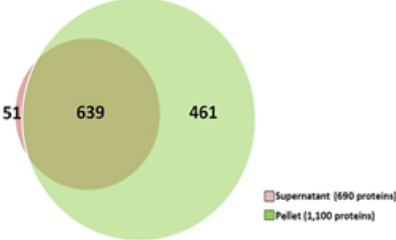

*Q Exactive MS*

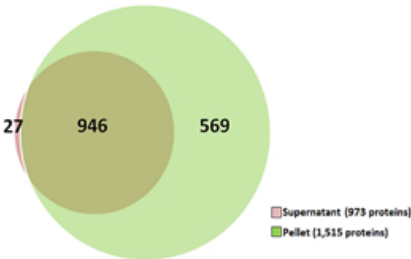

B. Assemblage A (WB)

*Orbitrap MS*

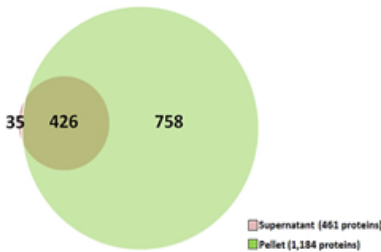

*Q Exactive MS*

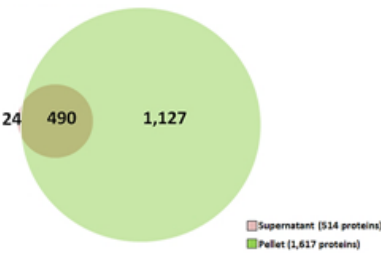

Supplement: Additional Files [file giy003_supp.zip › Additional File Figure S3.pdf]

Figure S4

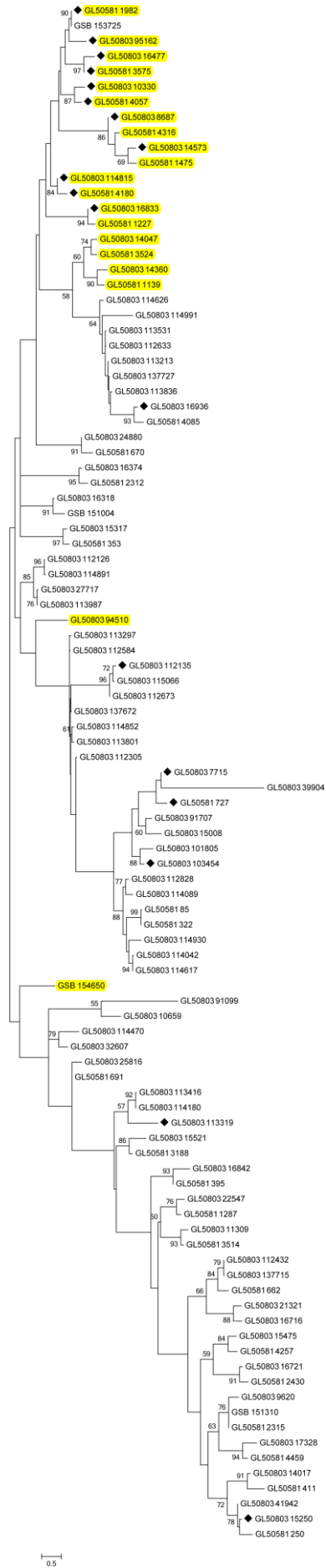

Supplement: Additional Files [file giy003_supp.zip › Additional File Figure S4.pdf]

**Figure S6:**


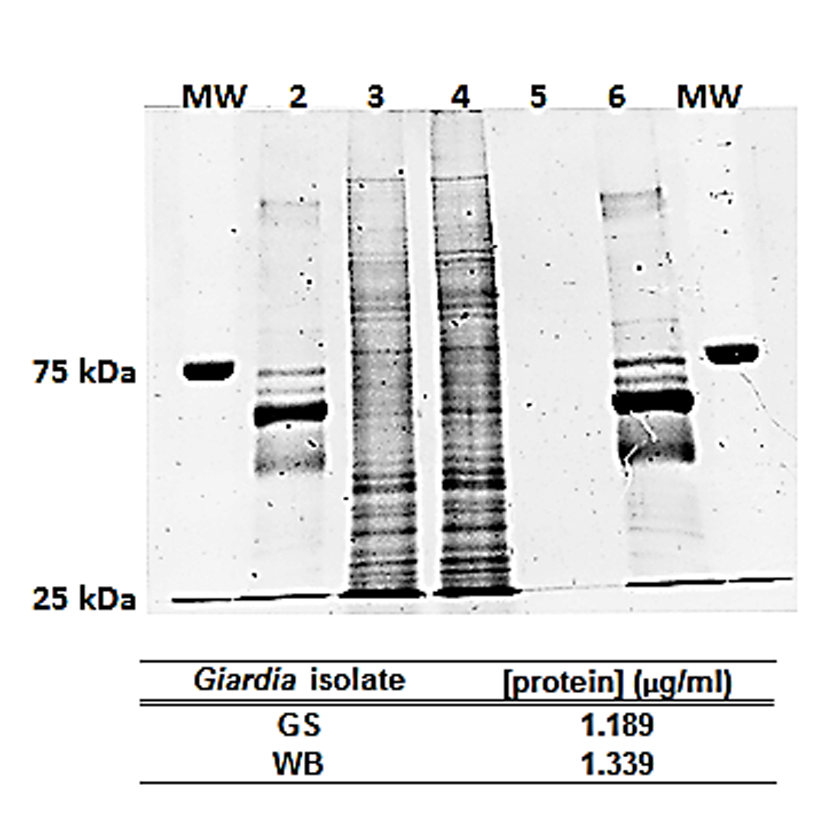

Supplement: Additional Files [file giy003_supp.zip › Additional file Figure S6.docx]

**Table S1**


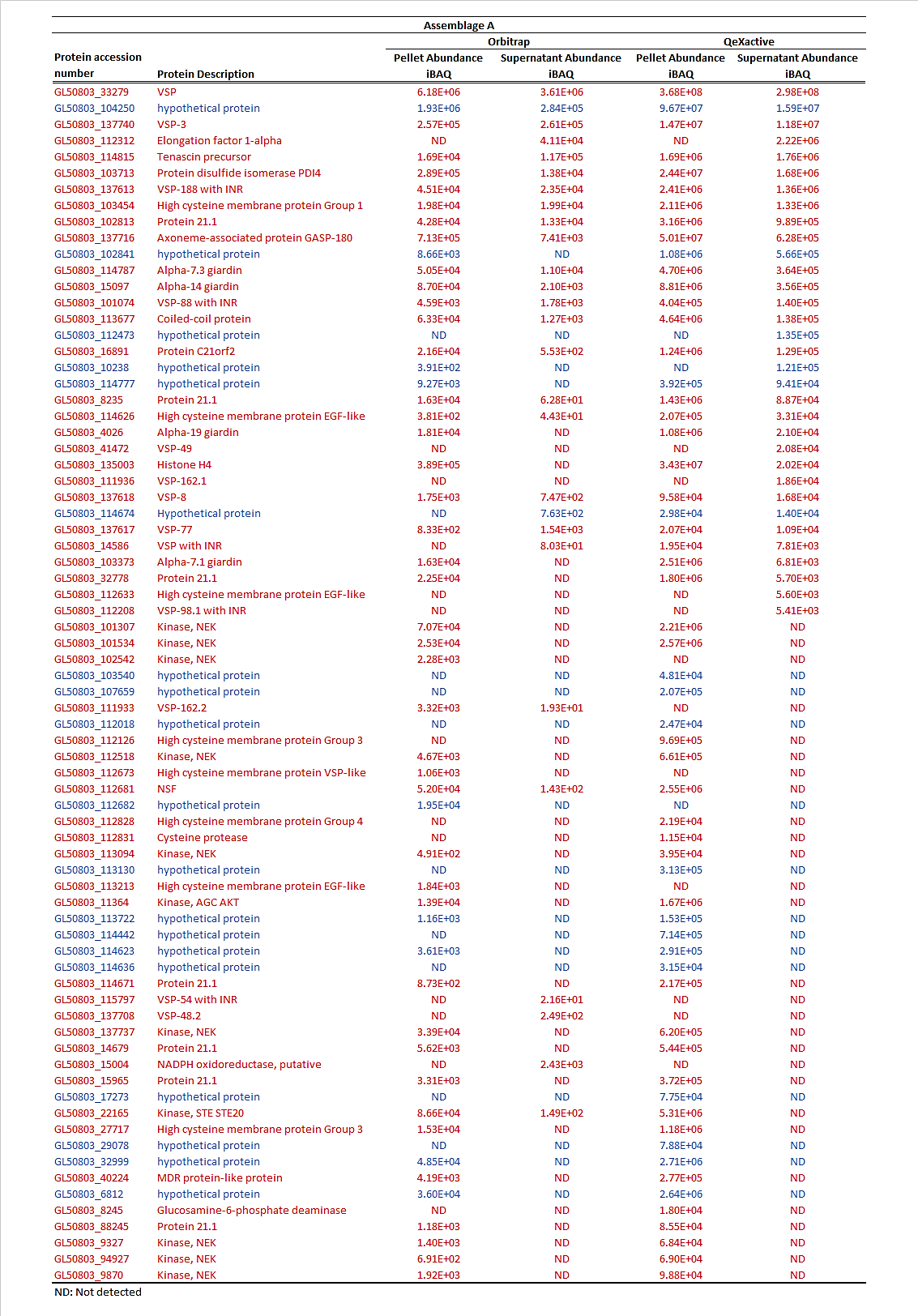

Supplement: Additional Files [file giy003_supp.zip › Additional file Table S1 Amended.docx]

**
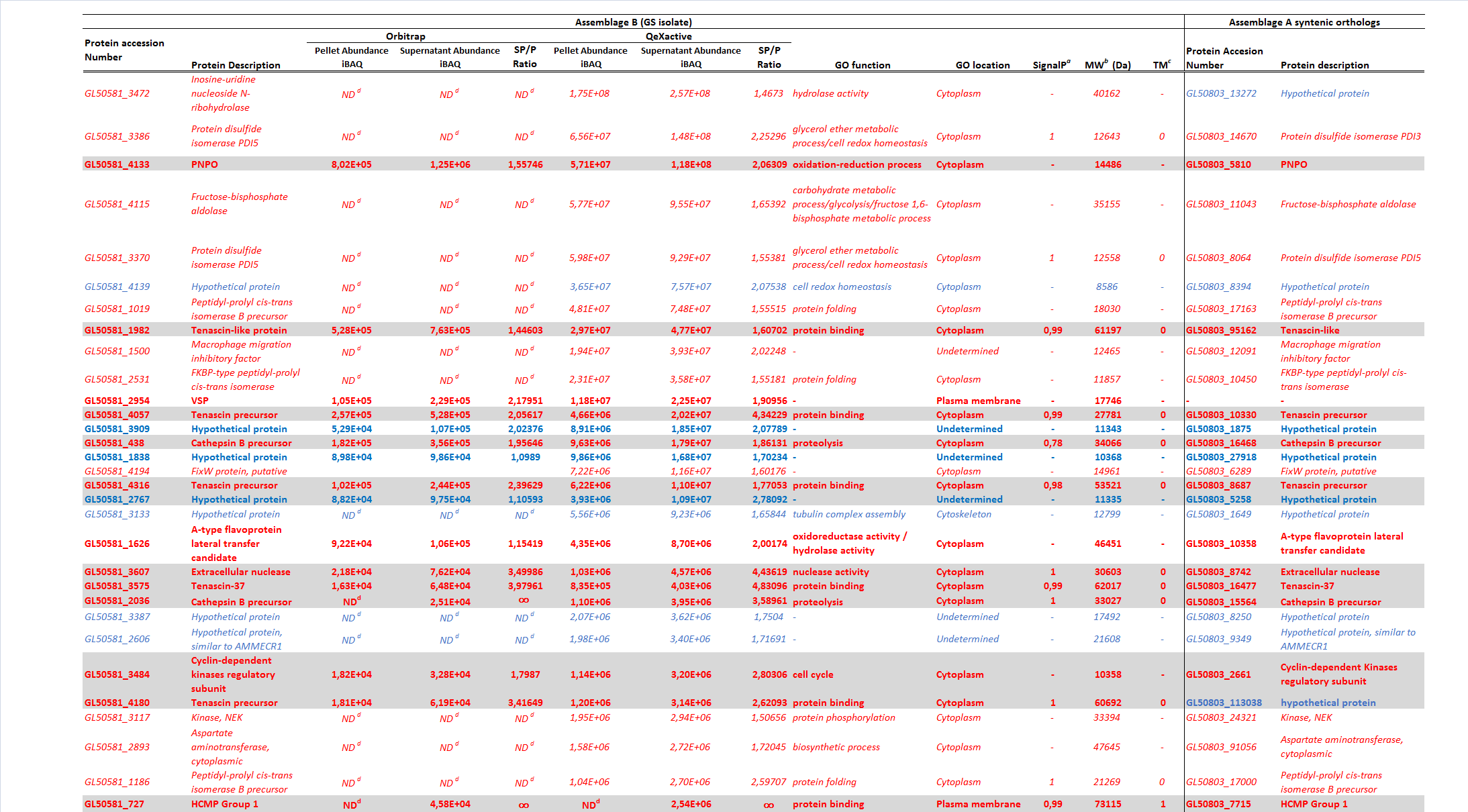
Table S3:**

**Table S3 (Cont.):**

**
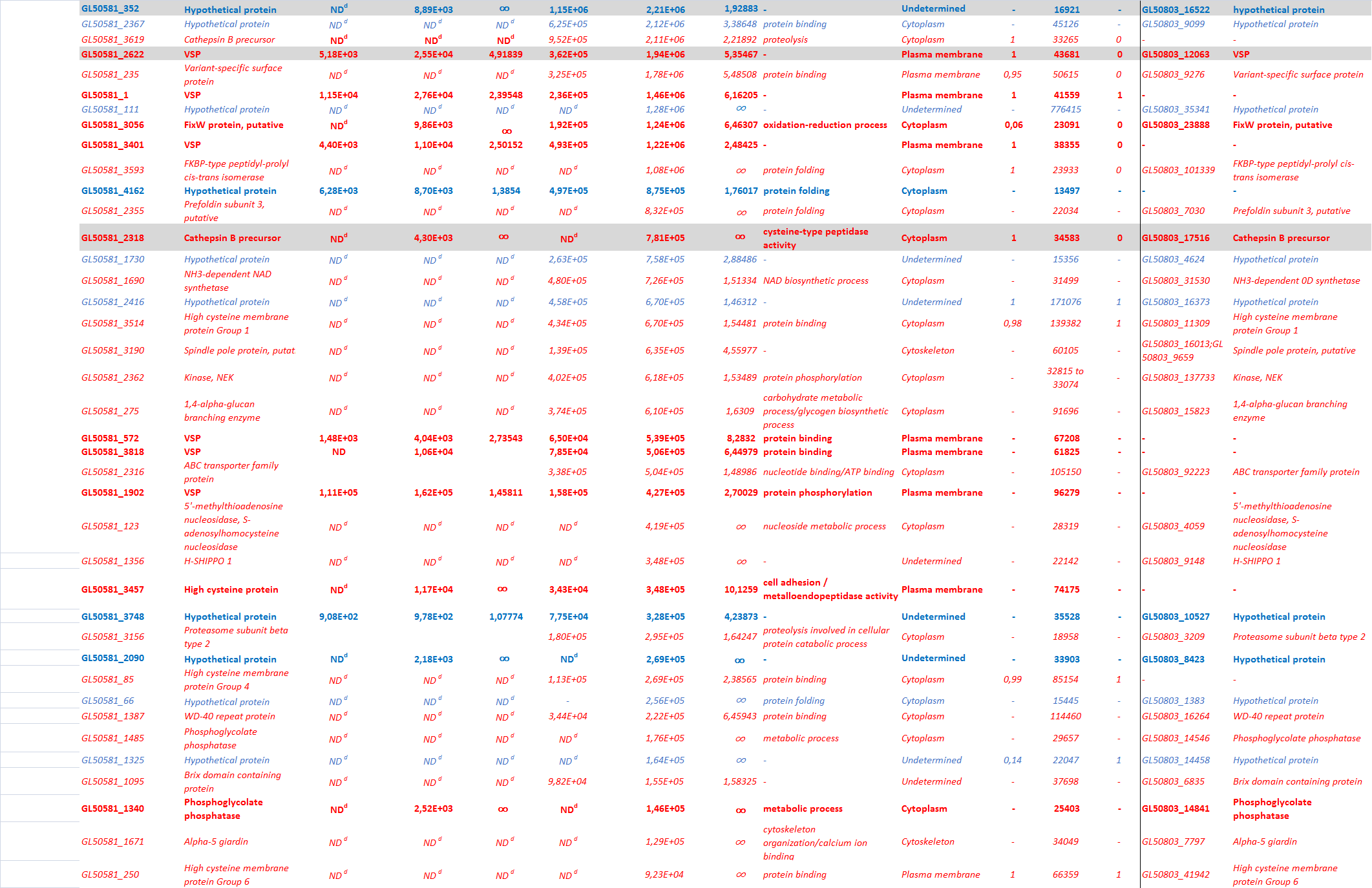
**

**Table S3 (Cont.):**

**
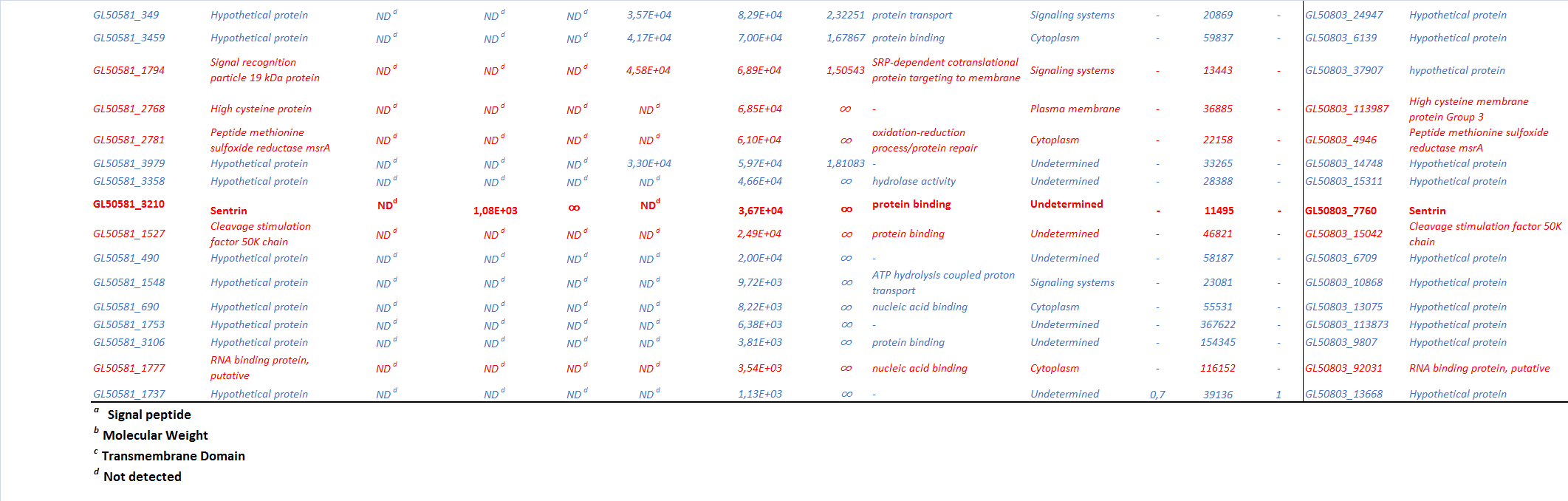
**

Supplement: Additional Files [file giy003_supp.zip › Additional file Table S3 amended.docx]

**Table S4:**

**
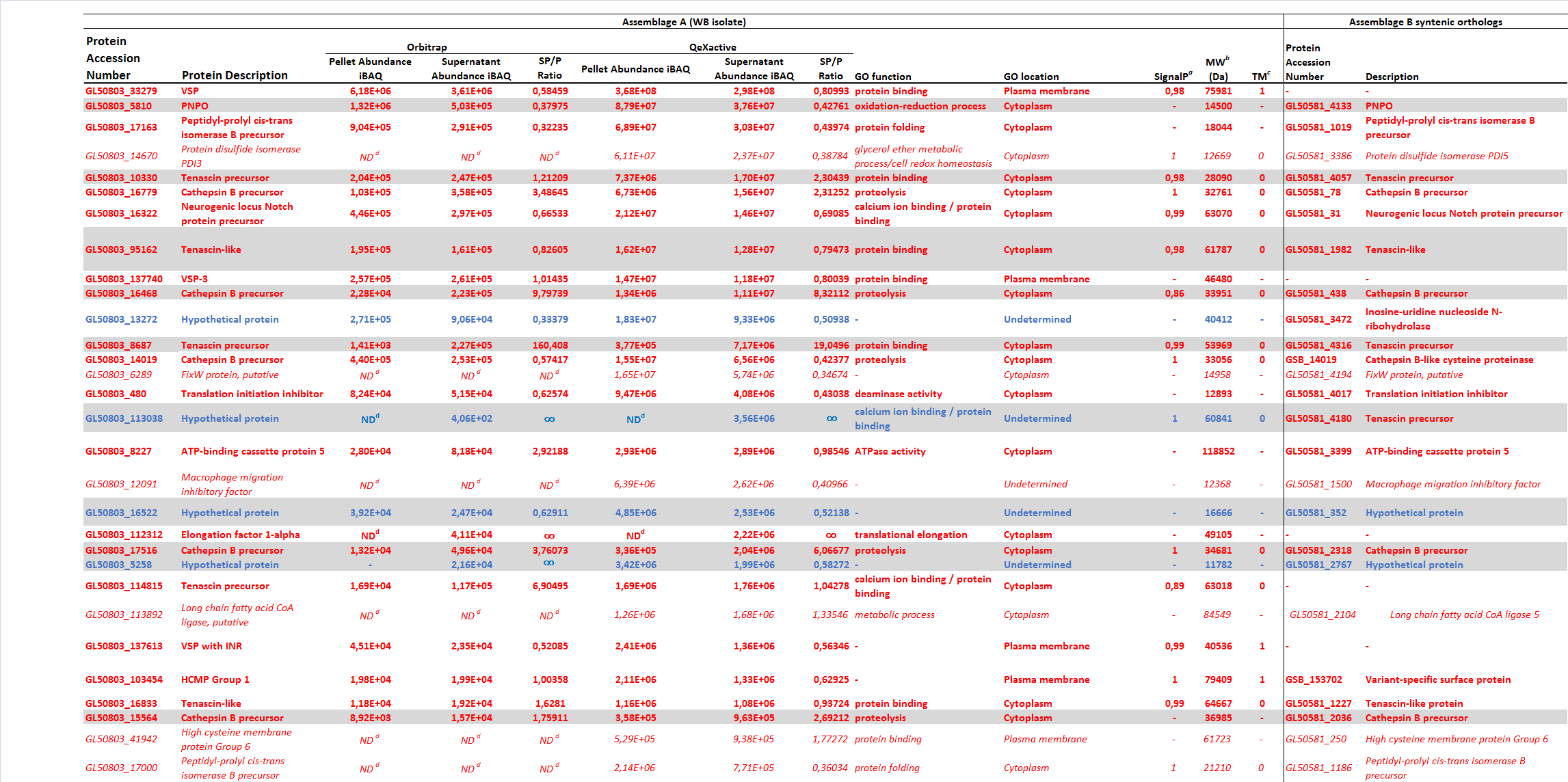
**

Table S4 (Cont.):


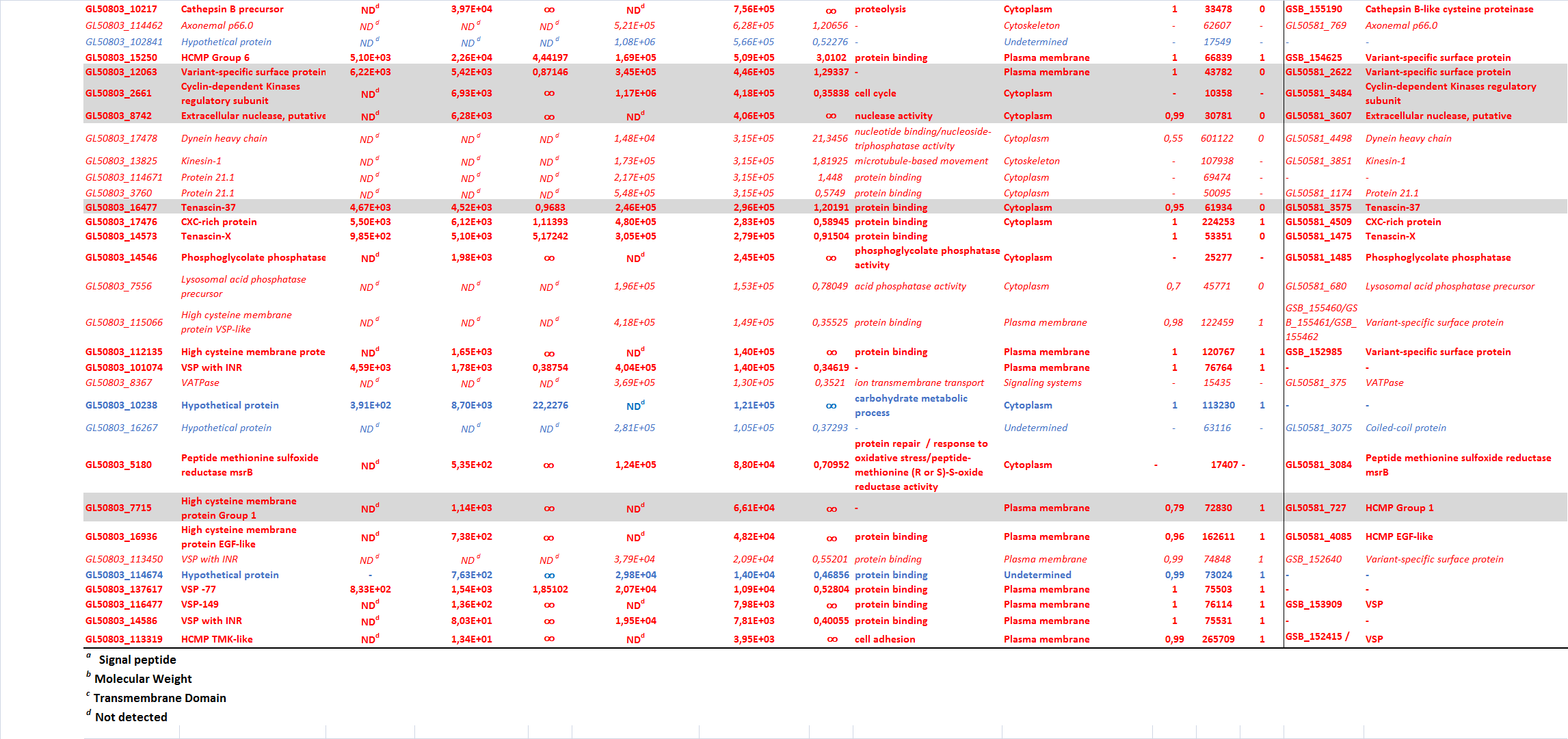

Supplement: Additional Files [file giy003_supp.zip › Additional file Table S4 amended.docx]

**
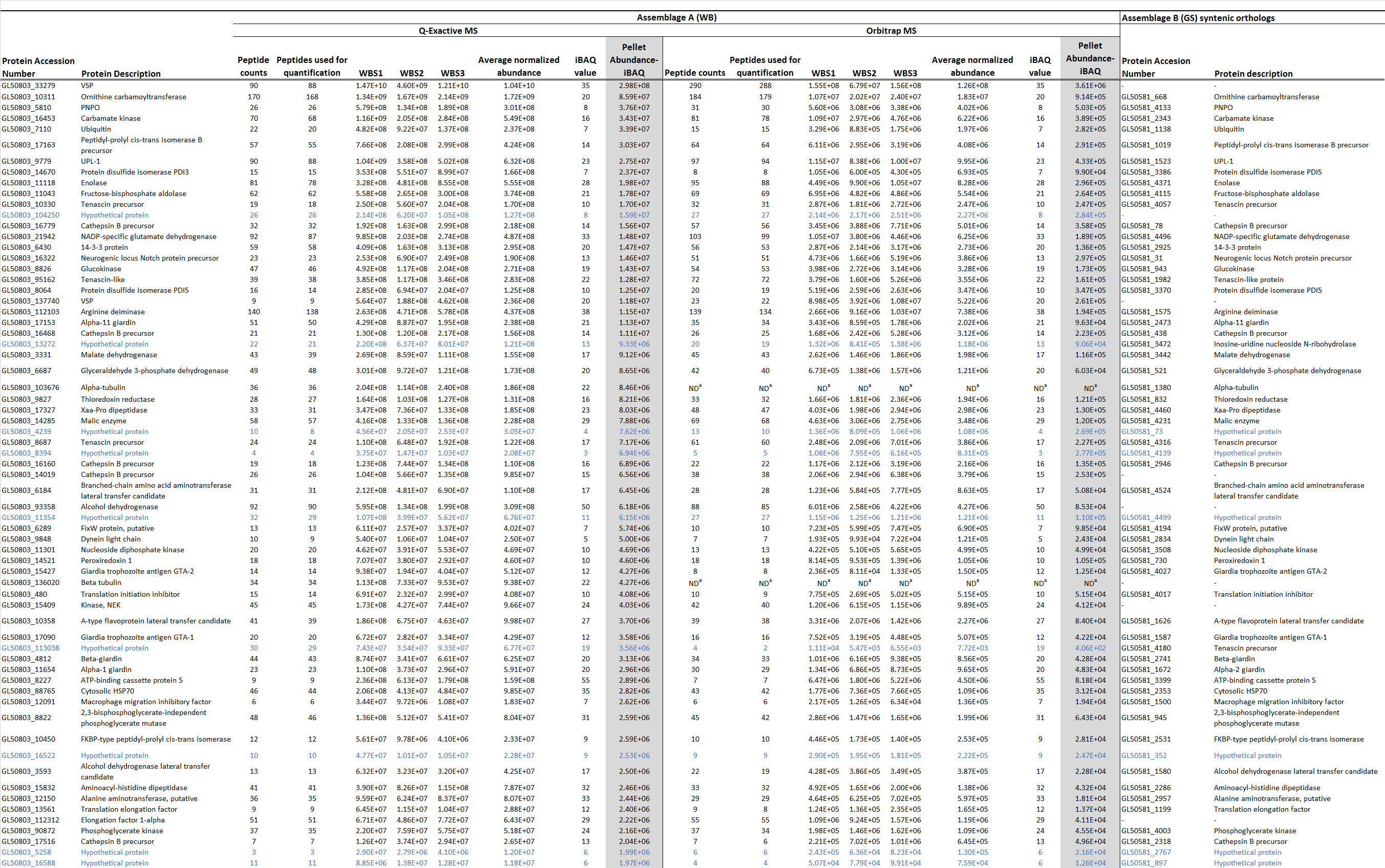
Table S8:**

**
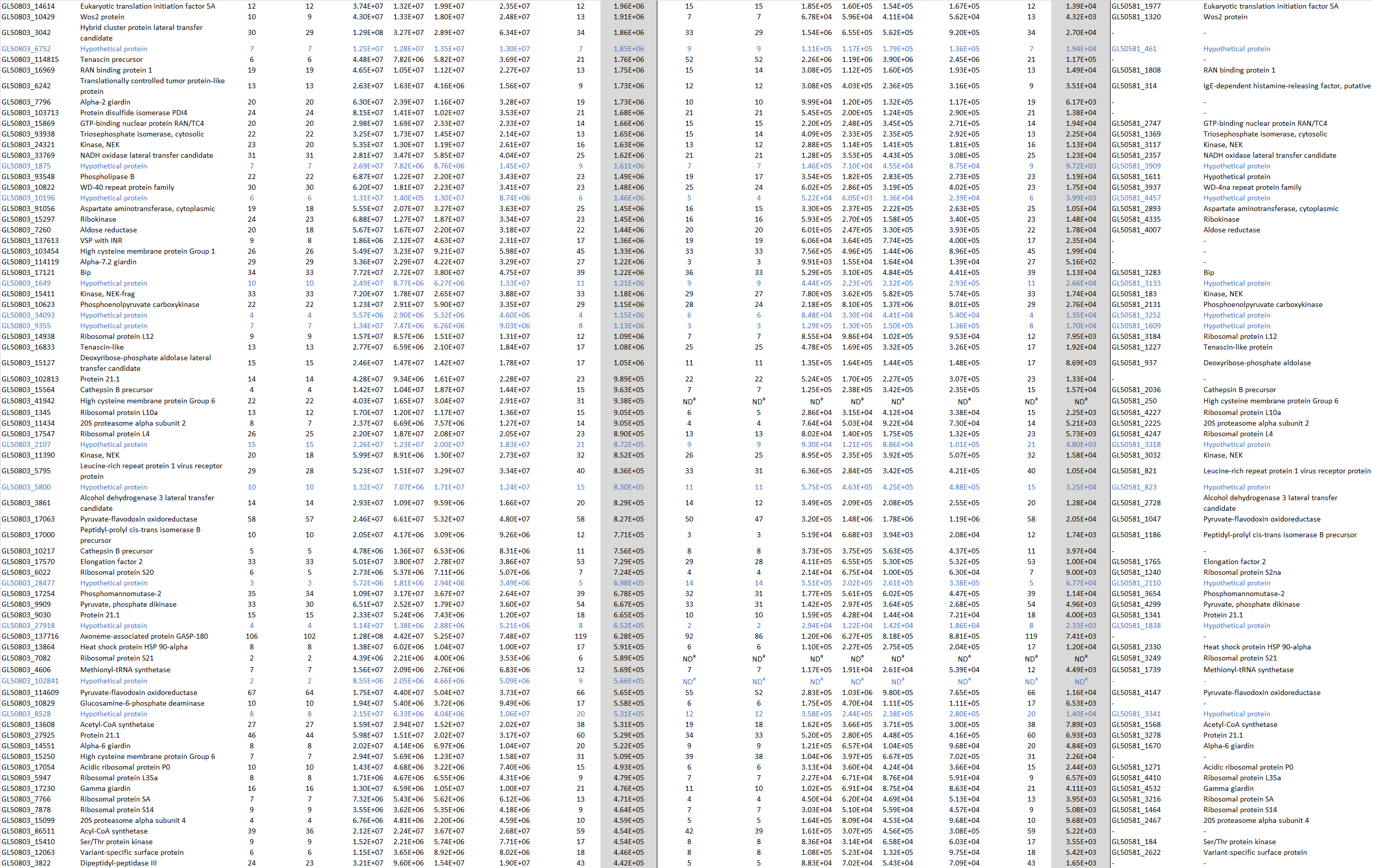
Table S8 (Cont.):**

**Table S8 (Cont.):**

**
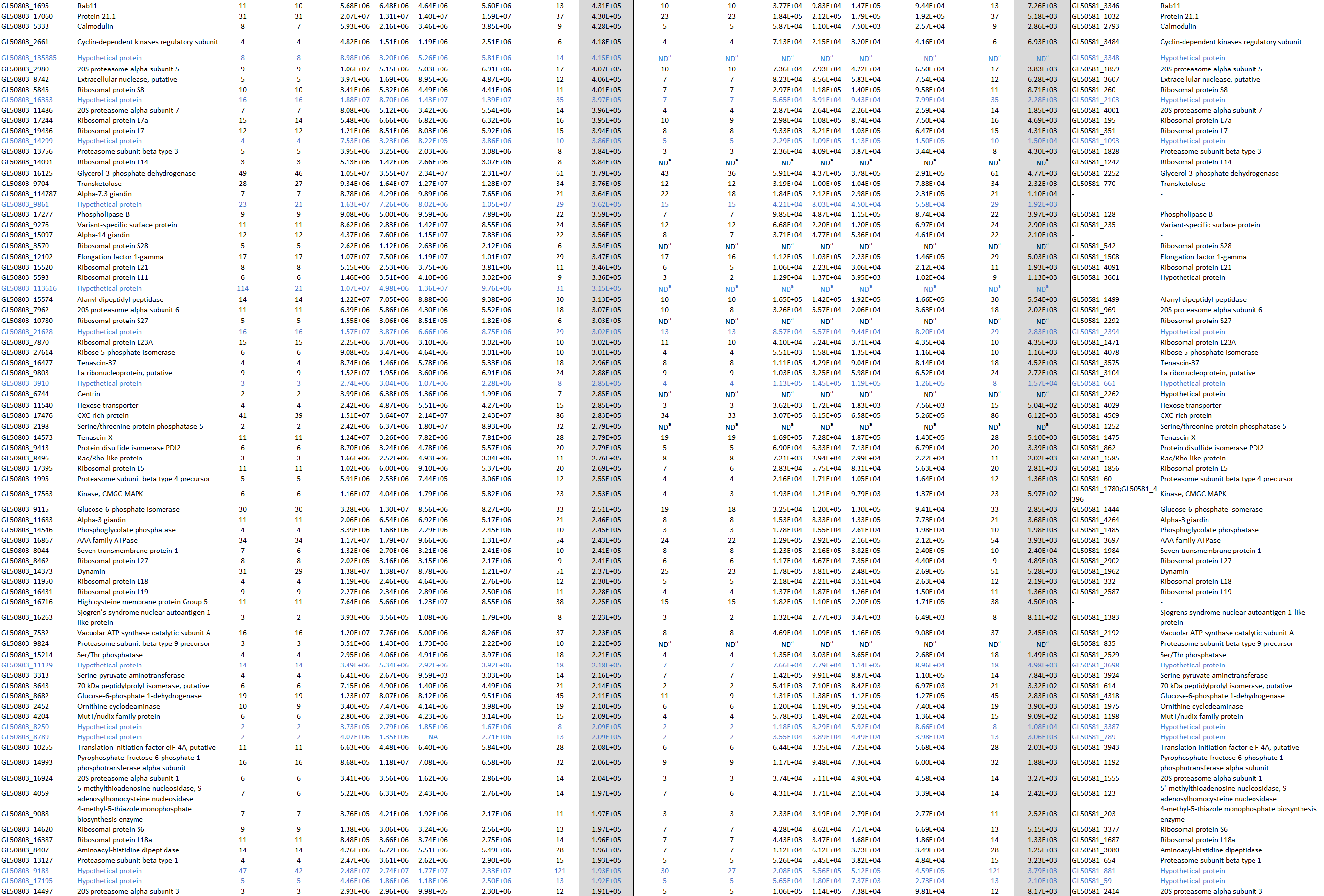
**

**
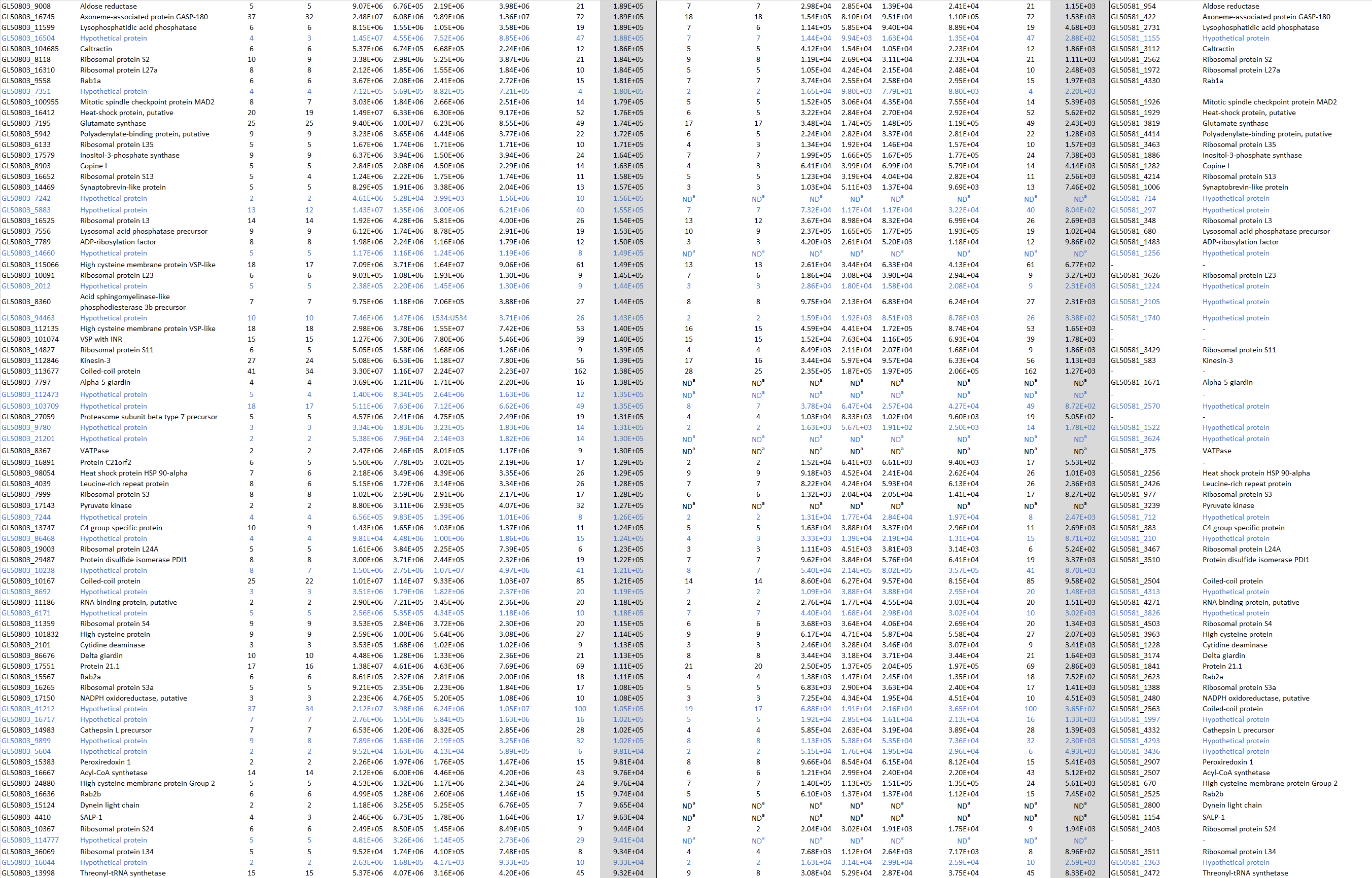
Table S8 (Cont.):**

**
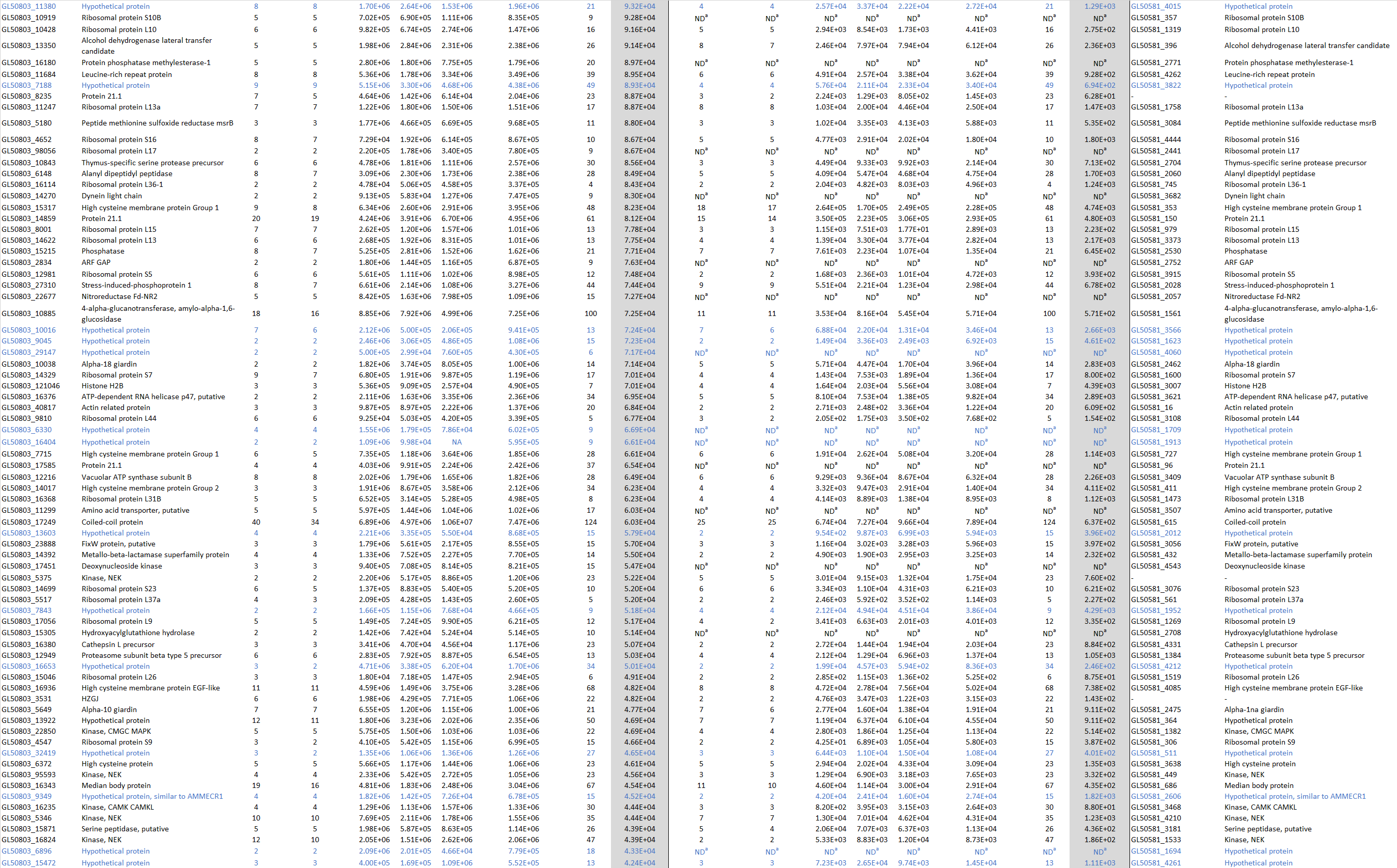
Table S8 (Cont.):**

**
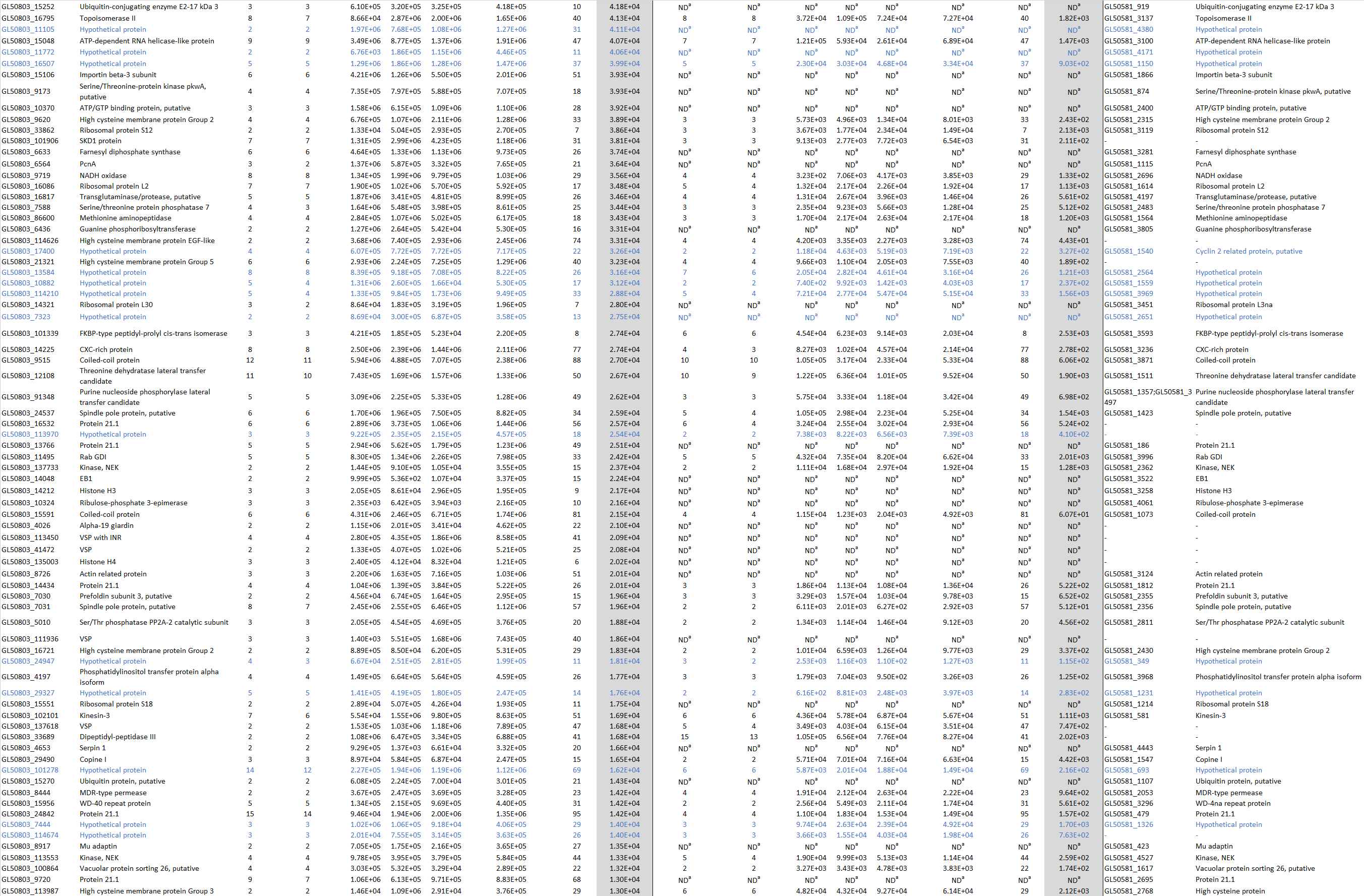
Table S8 (Cont.):**

**
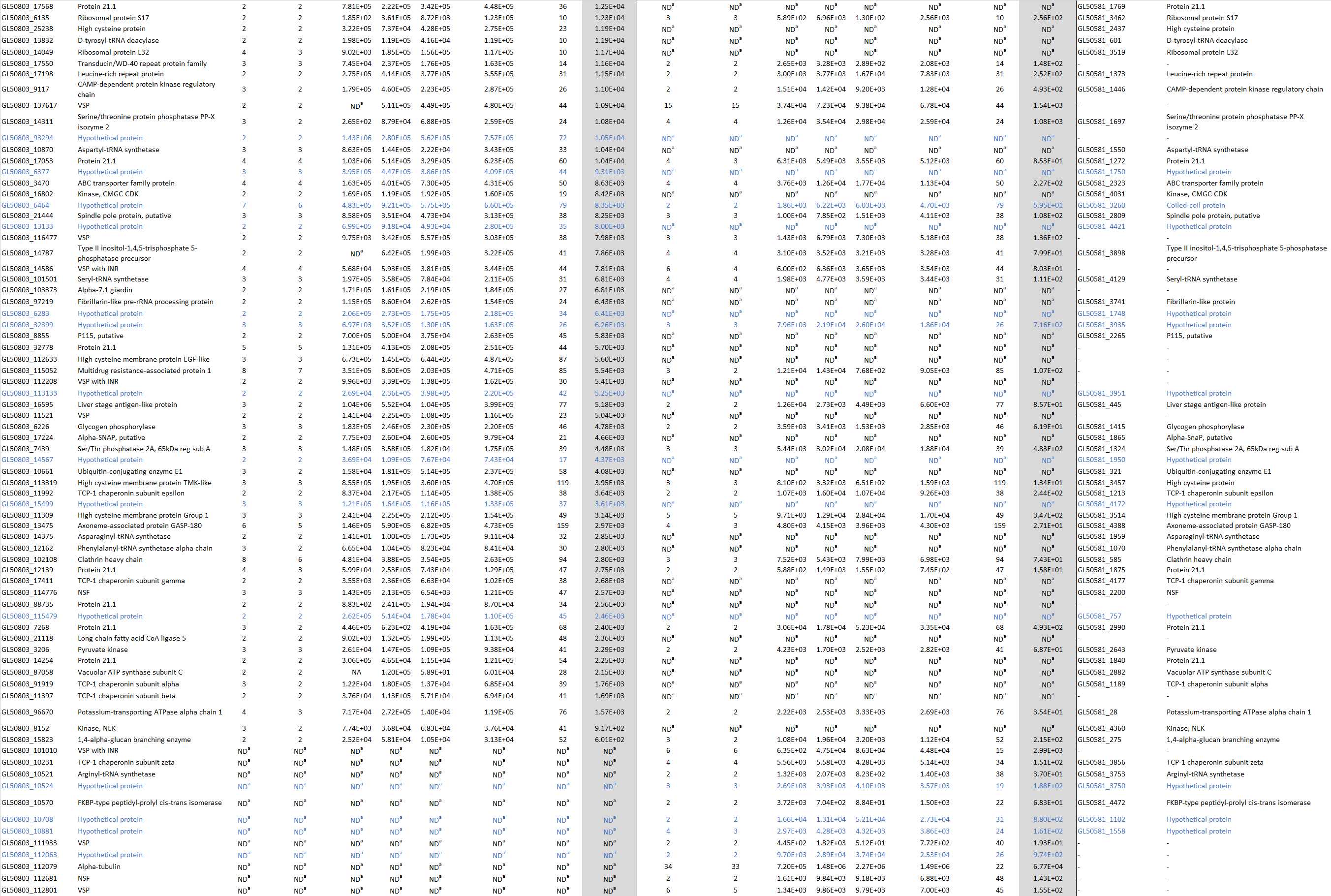
Table S8 (Cont.):**

**
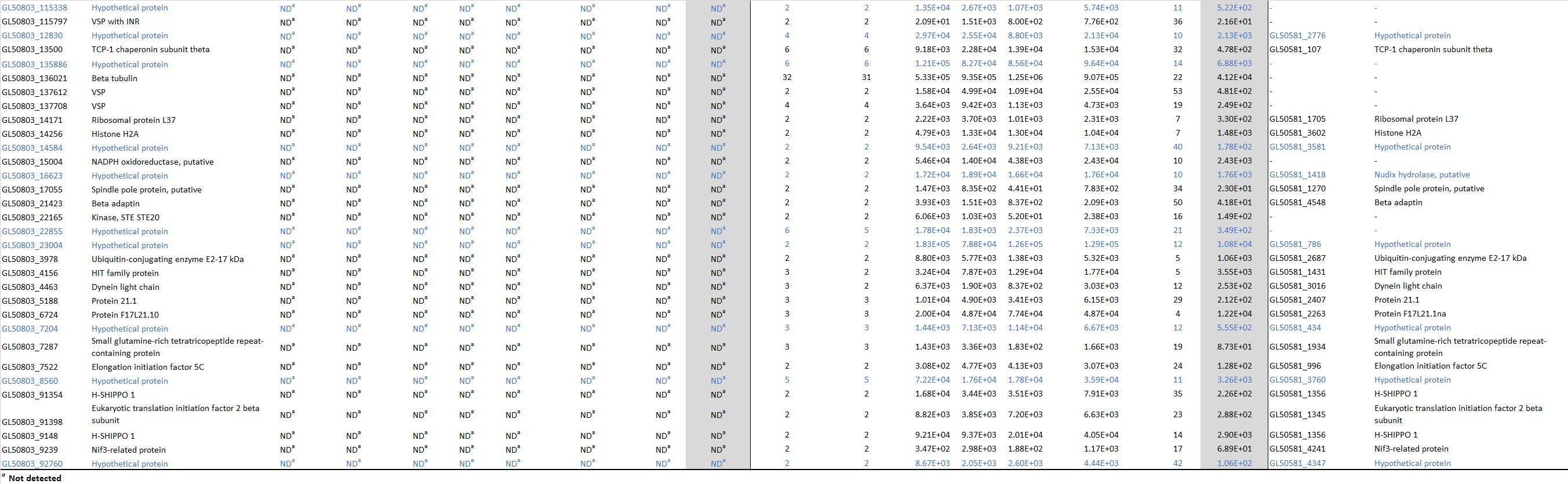
Table S8 (End):**

Supplement: Additional Files [file giy003_supp.zip › Additional file Table S8.docx]

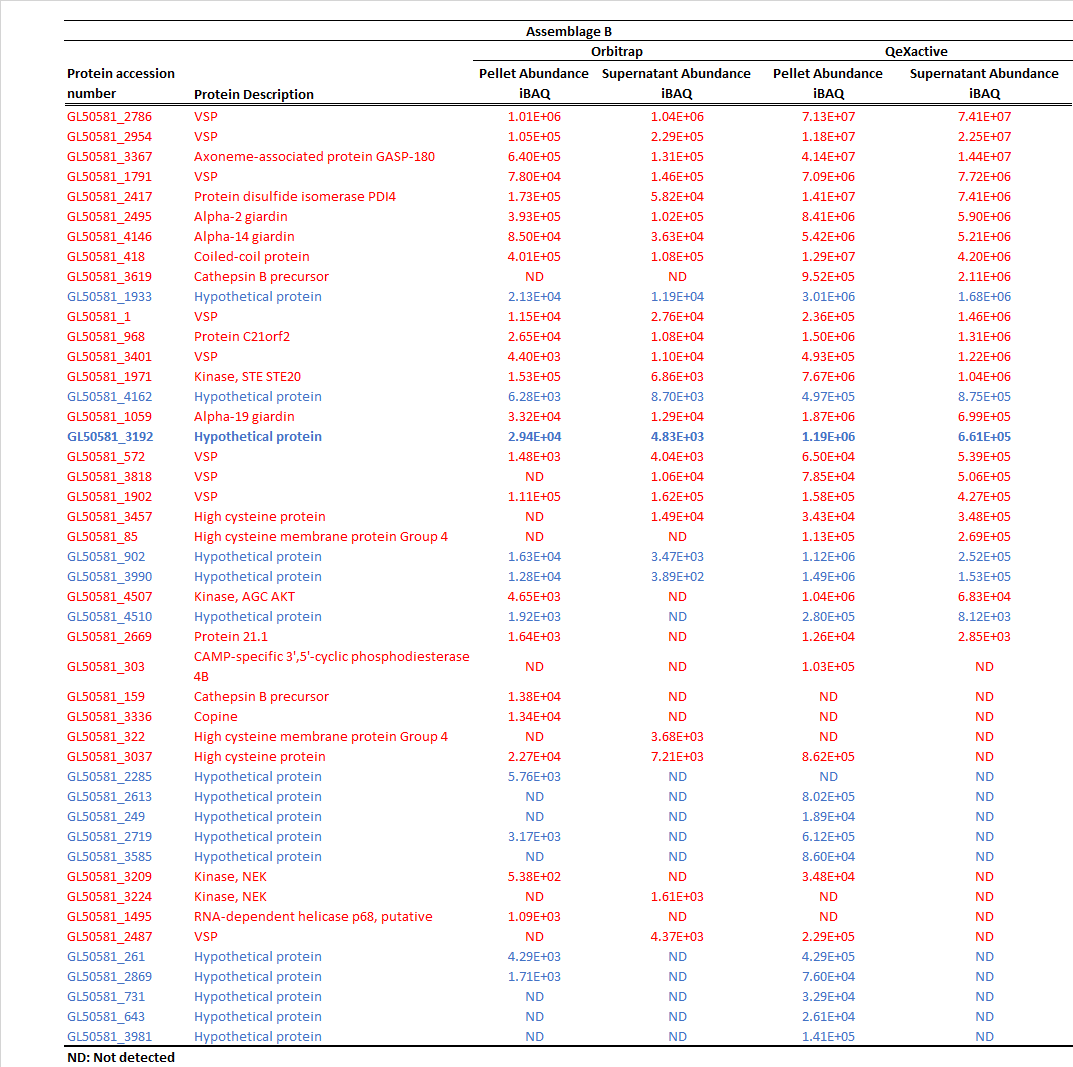
**Table S2:**

Supplement: Additional Files [file giy003_supp.zip › Additional Table S2 amended.docx]
