# Supplementary material for: Giardia secretome highlights secreted tenascins as a key component of pathogenesis
Source: Gigascience. 2018 Jan 29;7(3):1–13. doi: 10.1093/gigascience/giy003 (PMC5887430; doi:10.1093/gigascience/giy003)
Supplement: Response_to_Reviewer_Comments_Original_Submission.pdf [file giy003_response_to_reviewer_comments_original_submission.pdf]

Author's Response To Reviewer Comments

Responses to the Reviewers and Editor’s comments Regarding: “Giardia Secretome Highlights Secreted Tenascins as a Key Component of Pathogenesis”, GIGA-D-17-00132

Summary:
Thank you to you and your reviewers for a set of incisive comments and thorough suggestions on the manuscript. We have incorporated as far as it was possible for us to do so and as a result, we hope that you will find the paper much improved in general. The methods section in particular has been significantly improved and detailed. Fig 1 and 3 are now improved and of higher resolution, all the tables (supplemental tables included) were also updated to read protein abundance instead of protein expression and typos were corrected.

Response to Reviewer#1: (Text of Reviewer with authors’ responses in italic)
The authors have used proteomic technologies to fill in an important gap in the research regarding the secretome of Giardia. While this is a very important and very valuable venture, the manuscript fails to adequately report on the methods used to generate their data. The core of manuscript is a quantitative proteomic experiment, therefore it must describe the methods of acquiring proteomic data, how these data were searched and analysed quantitatively, and their consistency and reproducibility. Moreover, as this experiment involves transporting Giardia from normal optimised media to co-culture conditions and non-parasite media, more information is needed on these aspects in order to verify they were adequately controlled. I have provided some overarching comments that must be addressed before further evaluation, most of which are centralised on amending the methods so they adequately report the experiment. (Thank you for your suggestions, all those questions will be answered below in your comments)
a) Proteomics:
- You have stated you have used an LTQ-Velos Orbitrap and a Q-Exactive. While these are two mass spectrometers capable of generating the high-resolution data for quantitative analyses, they aren't necessarily complementary or orthogonal platforms. What is the rationale for doing technical injections on these machines?
The aim was not to assess the technical differences between the two techniques but to have a better coverage and to increase the robustness of the data as preliminary data showed a huge discrepancy in the quantification when both MS were used on different replicates. Only the proteins identified by both techniques were included in the quantitative analysis to increase the robustness of the data. This was added in the results section to explain the need to use both MS techniques here.
The methods for the mass spectrometry, including chromatography, fragmentation regime, MS and MS2 conditions, data acquisition are missing from the methods, so it isn't possible to tell if they were run in ways to acquire different types of data. Were there differences in the way the gradient was run, or ions were acquired to justify using both?
Both MS were similarly set technically to have conserved parameters and three independent replicates were run on both MS as we only wanted to increase coverage and not compare both MS techniques. All the conditions and protocol were added into the proteomics section of the Methods to make it clear for the reader.
Were the ratios generated by each method averaged for each identification? Were they in agreement?

Ratios were generated from the averaged iBAQ abundance for each identification and this for each method. Ratios were usually in agreement, when ranking and analysing the quantitative data, only the Q-Exactive value used to rank proteins as the technique has been shown to be more sensitive.
The authors need to justify why they have used both mass spectrometers, in what way they combined the data for quantitative analyses? This needs to be discussed in the manuscript. Given that the QExactive identified nearly all the proteins in the LTQ Orbitrap runs, was there a benefit for keeping both datasets and if so this needs to be discussed.
See above for explanations, it was added in the results section to explain the benefit for using both techniques
- As stated above, the mass spectrometry methods are absent in the methods. The injection conditions, chromatography gradients, fragmentation, MS and MS2 conditions, data acquisition must be included.
As stated above, all those conditions have been added to the Methods section.

- Sample preparation is not described. Methods say the trophs and s/n were separated and proteins harvested. How were the secreted proteins harvested/precipitated from the media? Was this done at Liverpool? No conditions for protein-peptide digestion are given. More information needed.
As stated above, all those conditions have been added to the Methods section. The protocol to prepare supernatant and pellet samples prior to MS was done on site before being sent to Liverpool for the MS analysis. Protein denaturation and peptide mixture preparation as well as the MS and peptides analysis and matching were done in Liverpool. Once all quantification and protein matching were done, the datasets were sent back to UEA for further analysis of Giardia secretome.
This has now been specified in the methods sections

- Please reference the genomes used for database searching, the release number from giardiadb.org and also what search software/algorithm was used for matching peptides. Please state the search conditions, such as variable and fixed modifications, tolerance, missed cleavages etc etc. This, like the mass spec, needs to be in the manuscript. I have noticed that some of these are given in the description for the PRIDE data deposit, but these also need to be in a supplementary methods or the methods themselves.
All genome references (ATCC), release number of giardiadb.org and software/algorithm used for matching peptides as well as search conditions were added in the methods section in the proteomics sub-section, under Data analysis.

- Judging from the tables the method of quantitation is iBAQ. There is no description of this in the methods or the results. The method for generating these abundance values must be in the methods and results. Some readers may not be familiar with iBAQ, so some explanation of it in general could be valuable to the readership.
Thank you for pointing that out, a brief explanation has been added in the results section and a more developed description in the methods section.

- As above, if you are choosing to keep both datasets from the mass spectrometers in the manuscript, then please start their consistency and agreement at the quantitative level of protein

abundance as the manuscript only states their agreement at the identification level.
Prior to perform any proteome or secretome analysis, all quantitative datasets were compared by running a Spearman correlation test. This showed that the datasets were exploitable for a quantitative analysis of the secretome and proteome. However, to avoid any bias in the analysis, datasets were first analysed separately. Only the quantitative data from the replicates run on one or the other MS were merged. Secretion profiles were obtained for both techniques and then compared to see any discrepancy in the protein ranking between the two techniques. Apart from a slight variation in values (which is expected as the techniques are different and also the Q-Ex is more sensitive than the Velos-Orb) the ranking were the same regardless of the MS technique. We decided to put the protein abundance for both technique on the supplemental tables and to simplify Table 1 with only the Q-Ex values as it is more sensitive.

- The top 50 proteins were considered to be the most likely candidates as secreted proteins. What was the ratio cutoff that this included (min/max)?
The top50 cut-off included proteins with a ratio and some proteins with only an abundance value in their supernatant dataset suggesting that the latter were most likely secreted as their abundance in the proteome was potentially too low to be picked on any of the MSs. For the proteins with a ratio, the range between the top50 proteins varies greatly from one assemblage to the other. E.g.: from 0.3 up to 21 for WB isolate and from 1.5 to 10. Therefore, to simplify this analysis we decided to just focus on the top50 for now.

What were the ratios of currently known secreted proteins in Giardia, and could these be used (or at least discussed) as internal standards considered as an alternative method to filter candidates?
Since at the time of the work there was no existing gold standard study for providing “known secreted proteins” of giardia trophozoites, because many of the studies which have looked at trophozoite secretion did so in the presence of intestinal epithelial cells not from steady state supernatants and because there did not exist a consensus for which proteins are secreted and which are not we undertook the analysis “de novo” without reference to “known positive controls” which we considered might be wrong and bias any result - except insofar as we did evaluate the number of proteins predicted to have a secretion signal. Our results do however strongly support and substantiate work published in scientific reports by this reviewer in the past few years and we have now expanded the discussion with regard to known secreted proteins in Giardia highlighting this point.

- You used three biological replicates, were these used in order to filter for only reproducibly identified proteins? All the supplementary files have data at the average level? How were replicates used in the identification and quantification of proteins? You say minimum 2 peptides were needed, but what if that was found in only one of the replicates?
As the reviewer suggests we used three independent biological replicates primarily to filter for reproducibly identified proteins but also robustness of the data. If a protein was identified and quantified in only one of the three replicates, it was excluded from our final analyses.

- You need to provide a supplementary table of your proteomic identifications and quantification for your datasets of the ~1600 proteins.
We can provide these in this way, but believe that the integration of our complete dataset with EuPathDB which will be available in the next release will provide this in a far more accessible

and elegant way and make any such spreadsheet appear cumbersome and redundant. The problem with accessing with the PRIDE files no double exacerbated the reviewer concern here which has now been fixed (below).

- The PRIDE deposit contains files, but there are no titles on these files? What are these? They have different numbers for accession but no titles? These are not the raw files? To what level have they been processed? Please clarify what these files are.
Thanks for looking into the PRIDE files, it appears that some of the raw files had been mixed up during the download onto the database. We contacted PRIDE IT team and got this sorted. The names of the raw files were also updated by adding the technique into the names. S stand for supernatant and P for pellet. The number 1, 2, and 3 represent the replicate number for each MS. The Progenesis (peaks) files with only the access number refer to the Velos data as we used it as the standard technique which created files without the technique into the title.

b) Culture conditions:
- Please state throughout if you used/harvested adhered or non-adhered or total trophozoites.
Thank for you spotting that missing bit, the total trophozoites were harvested to run the experiments. This was added in both the results and methods sections.

- You say the 'reference isolates', please cite the ATCC numbers.
ATTC numbers were added in the Data description and the Methods sections

- As you are moving isolates to separate media or co-culture conditions, please state whether the trophozoites were in aerobic or anaerobic conditions, including during co-culture.
For all experiments and the standard in vitro culture of the trophozoites, parasites were under aerobic conditions with 5% CO2. This was added into the methods section.

- There needs to be more information in the methods in regards to the experiment regarding Giardia/Giardia soluble products and the CaCO2 cells and the electrophysiology. In the Data description it says Giardia were added, in the results on page 6 is states cells were grown alone, with Giardia or with diluted supernatants. None of the information about these different conditions, their time frames or experiment design are given in the detailed methods regarding 'electrophysiology'. Not only does this need to be added, but the description of the experiment between Data description, results and methods needs to be made consistent throughout otherwise is it too confusing.

The full methodology for the culture of the Giardia parasites, the Caco-2 cells and the electrophysiological assays has now been given in the Methods section (p12-14) of the manuscript. We apologise for the confusion caused by our description of the experimental conditions. Clarification has now been given for the different experimental conditions used for the electrophysiological assays in the Methods section (p12-14), Figure 2 legend (p18) and Analyses section (p5-7).

c) Other
- Given that VSPs are known membrane proteins, and you are looking at media fractions, have you considered the possibility they may be cleaved protein products? What sequence coverage

are you getting for your VSPs, including the termini? Can you say they are indeed secreted, or could they be VSPs cleaved from the membrane during turnover?
We agree with the reviewer that this is likely, our data is consistent with at least some exterior (outward facing) plasma membrane proteins being clipped or shed from the surface rather than being actively secreted per se. We were unable to discriminate exact mechanism by which surface proteins were entering the supernatants during this study and have deliberately avoided describing them as secreted, but we are seeking funds to do exactly this using a more targeted approach and employing cell biological and transgenic as MS based methodologies.

Response to Reviewer#2: (Text of Reviewer with authors' responses in italic)
This manuscript revealed Giardia secretome under steady state of growth in vitro by proteomics methods and also confirmed that the secreted molecules adversely affect the homeostasis of enteric epithelia. The authors also concluded that Tenascins was a new class of virulence factors. This research provided a comprehensive proteomic data on Giardia secretome, introduced a novel pathogenicity mechanism, therefore is worthy of publication. However, there are still some problems needed to be revised before acceptance.
Thank you for your comments. We have modified the manuscript and hopefully improved it following your suggestions (see below)
1. More comparison between this research and previous Giardia proteomics research should be discussed, especially on the topic of whether the parasite-host cell interaction can induce the proteomic changes of the parasite.
This was indeed lacking in the discussion section, thank you for pointing it out.
Comparison between our findings and the known Giardia secreted proteins has been added in the discussion section. We have in particular discussed our findings on the metabolic proteins previously shown to be secreted upon interaction with host cells. Our data shows that these specific proteins are present in both secretome and proteome but at a very low abundance in secretome and they are therefore not in the top50 cut-off. This suggests an up-regulation of their secretion upon host-cell interaction as Svard and his team suggested. The other known to be secreted proteins were also discussed.
2. The major advantage of this research is that Tenascins was defined as a new class of virulence factor of Giardia. Considering that this is the major innovation and proposed novel mechanism of pathogenicity for Giardia, the authors should try to express them in 293T cells, insect cells or in vitro none-cell cell lysis system to analyze the pathological function of the recombinant proteins. This results will strongly support the Figure 3 novel mechanism proposal.
We agree entirely with the reviewer that just this type of in vitro experiment would indeed strongly support our findings about a novel pathogenesis mechanism. However, as much as we would love to research more and add more analysis to support our findings, we cannot do that at the moment due to lack of funding. We have included just such experiment in our current grant application along with the provision of transgenic mutants which will enable structure/function analysis.
3. Figure 1 is not necessary and can be transferred as a supplement figure. Other analysis, such as alignment of protein function domains, signalling peptide prediction also can be included in this figure. To support the proposed novel mechanism of pathogenicity, the authors should pay more attention on the protein functions, such as function domain, 2D and 3D structure, etc.
We thanks the reviewer for these ideas, we have of course begun to look closely at the tenascins which are an interesting new family of mimics as well as the cathepsins and in particular and

their recent evolutionary history. We think the key findings about the protein are adequately incorporated in the figure and will aid the understanding of the general reader. Simple structural analysis does not show yield much more than we have described unfortunately – the proteins homology to actual tenascins are limited to the EGF domains where are fully intact in most cases. The proteins do appear to have emerged under selective pressure from common ancestors with HCMPs and VSP proteins and they are polyphyletic. In the future we hope that by expressing recombinant versions of the proteins we can deduce and evidence more about their structure of these proteins but this is I think well beyond the scope of this paper.

Response to Reviewer#3: (Text of Reviewer with authors' responses in italic)
From the biological point of view, the results are sounding and interesting. The manuscript is well presented. The choices of methods and accuracy of the procedure are globally good; the comprehensive of description of methods must be detailed. The authors definitively identify many proteins using mass spectrometry, some of which may be important for the interaction of Giardia lamblia with host cell. The manuscript is lacking any confirmation of these secreted proteins by another biological technique; however, that may not be trivial given the potential lack of antibodies.
However, I have pointed out below several concerns I have regarding this publication. The scientific content of this manuscript is interesting. It is advised to correct all the typing errors (Some corrections that I found, I indicated) and to have a last editing of the manuscript. I suggest improving the quality of the figures (as 1, 3). Please do not write protein name with uppercase (for example: replace some Cathepsin by some cathepsin).
Considering all together I can state that several points need clarification or revision before final decision.
Thank you for your comments and suggestions. We have modified the manuscript and hopefully improved it following your suggestions (see below)

ABSTRACT
Please change "N' terminus signal peptides" by "N-terminal signal peptide" and in the main text as well.
This was modified throughout the text and in the abstract.
ANALYSES
5/3: The use of expression is not appropriate here, in the main text and in the suppl. table. It is not "expression" but "protein abundance". Moreover, the term "SP expression" does not sound good to explain the more abundant proteins identified from the supernatant.
This changed throughout the text as well as in all the tables (including supplemental. tables)
The authors should explain what they used to do the ratio SP/P to compare the "abundance" of proteins. What did the value considered? Did they use the PSMs? In fact, you used iBAQ algorithm but it is not mentioned in the main text.
The iBAQ algorithm and its meaning were indeed missing from the main text, thank you for letting us know. We have added an explanation for our abundance value and iBAQ in the results section as well as the Methods section. The formula for the ratio was modified to state Sp or P abundance-iBAQ as it is what we used for the analysis. We hypothesised that proteins with a ratio ≥ 1 would be more likely secreted as enriched in the supernatant and would therefore be included in the analysis if the proteins were part of our top50 cut-off.
DISCUSSION

The authors could discuss about the high number of proteins from the supernatants; why they identified some proteins only from the supernatants since proteins are synthetized in the cell; and why they used two different mass spectrometers and they could justify the choice of these 2 spectrometers in function of the different results.

Not many proteins were identified only in the supernatant, between 10 to 20 proteins which is fairly low compared to the 1,600 proteins identified in total. However, this could be surprising and yet expected if those proteins are mainly secreted, their abundance in the cytosol would be so low compared to other proteins that it would not be picked on by the MS with the default settings, this show the limitations of the MS techniques.

We used two different MS due to preliminary data showing major discrepancy between replicates, which is why we decided to use both MS to increase coverage and robustness of the analysis. This has now been explained in the results and discussion sections.

The authors discussed the function of the more abundant secreted proteins, but did they find other proteins known secreted that could be confirm these results?

This was missing in our analysis and our discussion. Thank you for suggesting it. We have now added comparison between our dataset and the known secreted proteins in Giardia.

METHODS

it should be indicated the origin of the strains, and if the parasites are subjected or not to regular passages in animal (for example mice).

This was added into the Methods section, parasites were not passed through any animals, only in vitro in glass tubes. This was specified in the methods and results.

The Proteomic Analysis must be detailed: Cell lysis; Protein trypsin digestion; Peptide separation by reverse-phase chromatography; Mass spectra acquisition for both mass spectrometers; data analysis (.raw and protein interpretation).

The proteomics protocol was expanded and greatly improved to include all the processes in the Methods section

Did the authors work with the same number of cells from each strain? Quantity of proteins and peptides? How did the authors quantify the proteins and peptides?

As we wanted to have a protein concentration (determined by BCA assay) as high as possible and the “cleansing” of the supernatants by incubation in serum-free DMEM before collecting Pellet and supernatant samples may have caused stress onto the trophozoites, we decided to not work with a conserved number of cells but with the total trophozoites in mid-log phase growth. However, after protein digestion, 1 μg of digest was injected in each MS and this for all replicates and each strain.

9/15: should change "SDS page electrophoresis and..." by "SDS PAGE and..."

This was changed

9/21: What the cut-off of the vivaspin filter? 5000 MWCO? change Ambic by ammonium bicarbonate

The cut-off of the columns was 3000 MWCO, this was added in the manuscript. Ambic was modified as well and then the abbreviation added

9/28 and other place: should change giardiaDB.org by giardiaDB.org

Not sure what needed to be changed here if it was GiardiaDB to giardiaDB or vice-versa.

9/47 and 10/10: should change "mls" by "ml".

This has been done

10/6: Consider the following sentence: "...GlyH-101 (50mM) and" what?

We have extended and clarified the sentence to read, “Stock solutions of Amiloride (10mM), GlyH-101 (50mM) were made by dissolving in DMSO.”
The sentence was corrected
10/27: should write Giardia in italic
This has been done
10/32: Abbreviations are incomplete, for example FDR
All abbreviations were added in the abbreviations section and also in the text when required
14/7: Delete S in the sentence: "…Q-Exactive S Supernatant (S) abundance, from most to least abundant…"
This has been done.
