# Supplementary material for: Giardia secretome highlights secreted tenascins as a key component of pathogenesis
Source: Gigascience. 2018 Jan 29;7(3):1–13. doi: 10.1093/gigascience/giy003 (PMC5887430; doi:10.1093/gigascience/giy003)
Supplement: Response_to_Reviewer_Comments_Revision_1.pdf [file giy003_response_to_reviewer_comments_revision_1.pdf]

Author's Response To Reviewer Comments

Responses to the Reviewers and Editor’s comments Regarding: “Giardia Secretome Highlights Secreted Tenascins as a Key Component of Pathogenesis”, GIGA-D-17-00132

Summary:
Thank you to you and your reviewers for these positive and constructive we have addressed them all as fully as we are able to do so.

Response to Reviewer#1: (Text of Reviewer with authors’ responses in italic)
I would like to congratulate the authors for their extensive revisions. I think this has greatly improved the manuscript and facilitated understanding of their methods. I have a few comments and revisions that I feel the authors should consider prior to final publication.
Firstly, I understand that the reviewers are using both MS platforms (Q-Exactive and LTQ Velos) to increase robustness of their data. I do not think they increase coverage, as they state they are highly correlated, and nearly all of the Velos IDs are also identified by the Q-exactive MS (which is more sensitive and makes sense). However, given there is little discrepancy between the data from both platforms, that the data is already filtered by reproducibility, and the Q-exactive is the more sensitive machine, might the authors think to keep the extra, reproducible IDs from the Q-exactive rather than discarding them totally because they are not seen on the LTQ Velos? As secreted proteins are going to be low abundance (given they are only being produced and secreted over an hour or so incubation in DMEM), the sensitivity of the Q-exactive may provide additional secreted proteins? Maybe these can at least be provided in an additional supplementary table?

Thank you for these very insightful statements and suggestions. Although we agree that the proteins identified only by Q-Exactive should not be completely excluded from the analysis in this work we would prefer to only include the proteins identified by both MS techniques to keep the robustness of our data. However, we do also agree that including the proteins identified as most likely secreted only by Q-Exactive MS may also bring additional information to the reader; therefore; those proteins were added in supplementary Table S3 and S4 in italic to differentiate them from the proteins included in the analysis.

Secondly, given the authors are comparing WB and GS, and that these most likely represent cryptic species with different clinical pathophysiology, maybe the discussion/results could be added to in order discuss differences between assemblages. The authors have already provided a robust set of proteins common between assemblages, and tables of proteins identified exclusively in WB or GS. However, significant differences between assemblages in the proteomics and/or electrophysiology could provide important insights into the differences between these two, human-infective assemblages that is limited in current literature.

This part was indeed missing, thank you for pointing it out. Not many differences in terms of protein identification were found in the dataset but a few proteins had a great variation in their abundance from one assemblage to the other. Those proteins were put in a table (Table 2) and paragraphs were added in the results section and discussion sections.

Thirdly, while the authors do plan to deposit the data in the EuPath DBs, providing excel supplementary tables of protein IDs, peptide counts and IBAQ values for reproducibly identified proteins is a very valuable resource for other researchers looking to compare identifications and expression values. Some of these people may not be familiar or aware of the EuPATH databases and their systems. I strongly advise providing these for future reviewers, as it will greatly affect the accessibility of your data.

We are happy to provide this and have added 4 supplementary tables: Table S5-S8 containing all the proteins identified peptide counts and iBAQ values for each technique and by sample< For example Table S5 represents the list of proteins identified in the pellet of GS strain trophozoites for both Q-Exactive and Orbitrap MS.

Minor Comments:
Methods:
1) In the non-supplemented DMEM, what was the oxygen levels? Were these capped, filled flasks?

Parasites were incubated for 45 min in 1 ml of non-supplemented DMEM under their standard in vitro growth conditions. Pellets were resuspended in DMEM and transferred in filtered glass tube and put at 37°C with 5% O2. To help readers and reviewers understand the protocol, a supplemental figure was created and added with the whole protocol developed and used to cleanse and harvest pellets and supernatants: Figure S5. Reference to figure was added in the Method section under Proteomic analysis, Samples preparation. In addition, culture protocols are now provided.

2) What were the centrifugation speeds - higher speeds would have been likely to rupture cells, so it might be good to report this.

The centrifugation speed to separate pellet and supernatant was the speed used to usually pellet down Giardia “safely”: 3,000 rpm. This speed is the standard speed used to pellet down the parasites before freezing cells down and is unlikely to rupture cells. However, as we incubated the, in DMEM before harvesting pellets and supernatants, we performed a flow cytometry viability assay on pellet samples after centrifugation. The parasites were still alive as shown by supplemental Figure S1. But to be sure readers have all the key elements, all the centrifugation speeds used were added onto Figure S5.

3)In the electrophysiology, what are the patient strains? Are these verified assemblage A or B? Have they been typed?

The patient strain used in the electrophysiology was not typed. It was a stabilate prepared from a clinical sample at the Norfolk and Norwich University Hospital (NNUH) and was used alongside the lab strains to control for atypical activity (e.g. attenuation) that might have occurred as an artefact of protracted culture of the laboratory strains. Since the results were similar to the laboratory strains it serves to provide support that the results are likely to be generalizable for most human giardia infections.

Results:
1) I think that adding some further comments about viability and harvesting contingencies to ensure cell integrity (i.e. cell not popping) would be valuable when reporting supernatant vs pellet IDs around pg 12, ln 42-49. While contamination of the supernatant with proteins is a very difficult thing to avoid in this type of experiment, hence your experimental design in averaging pellet to supernatant ratios, I think showcasing steps to mitigate contamination would add more credibility to the results.

To help the reader visualise what was done to obtain the proteomic samples, a supplementary figure: Fig S5 was created and added as well as its legend explaining every step of the protocol

2) Were the supernatants harvested with protease inhibitors at any stage, or how was protease activity controlled during harvesting? SDS-PAGE was mentioned as a quality control step (which can also show whether there is any evidence of protease activity), but pictures of these gels are not provided in the supplementary.

No protease was added in the final cleansing protocol. Several protein concentration protocols were tried before deciding to use the Vivaspin columns and we followed the protocol from the manufacturer which did not require the use of proteases. SDS-PAGE were run after the protein concentration to verify that proteins were not degraded, and BCA assay performed to obtain the protein concentration as well. A supplementary figure: Fig S6 is provided with a photo of a representative SDS-PAGE gel.
