# Supplementary material for: Giardia secretome highlights secreted tenascins as a key component of pathogenesis
Source: Gigascience. 2018 Jan 29;7(3):1–13. doi: 10.1093/gigascience/giy003 (PMC5887430; doi:10.1093/gigascience/giy003)
Supplement: Reviewer_1_Report_(Original_Submission) -- Samantha Emery — 09 Jul 2017 Reviewed [file giy003_reviewer_1_report_(original_submission).pdf]

Reviewer Report

Title: Giardia Secretome Highlights Secreted Tenascins as a Key Component of Pathogenesis

Version: Original Submission **Date:** 09 Jul 2017

Reviewer name: Samantha Emery

Reviewer Comments to Author:

The authors have used proteomic technologies to fill in an important gap in the research regarding the secretome of Giardia. While this is a very important and very valuable venture, the manuscript fails to adequately report on the methods used to generate their data. The core of manuscript is a quantitative proteomic experiment, therefore it must describe the methods of acquiring proteomic data, how these data were searched and analysed quantitatively, and their consistency and reproducibility. Moreover, as this experiment involves transporting Giardia from normal optimised media to co-culture conditions and non-parasite media, more information is needed on these aspects in order to verify they were adequately controlled. I have provided some overarching comments that must be addressed before further evaluation, most of which are centralised on amending the methods so they adequately report the experiment.

a) Proteomics:
- You have stated you have used an LTQ-Velos Orbitrap and a Q-Exactive. While these are two mass spectrometers capable of generating the high-resolution data for quantitative analyses, they aren't necessarily complementary or orthogonal platforms. What is the rationale for doing technical injections on these machines? The methods for the mass spectrometry, including chromatography, fragmentation regime, MS and MS2 conditions, data acquisition are missing from the methods, so it isn't possible to tell if they were run in ways to acquire different types of data. Were there differences in the way the gradient was run, or ions were acquired to justify using both? Were the ratios generated by each method averaged for each identification? Were they in agreement? The authors need to justify why they have used both mass spectrometers, in what way they combined the data for quantitative analyses? This needs to be discussed in the manuscript. Given that the QExactive identified nearly all the proteins in the LTQ Orbitrap runs, was there a benefit for keeping both datasets and if so this needs to be discussed.
- As stated above, the mass spectrometry methods are absent in the methods. The injection conditions, chromatography gradients, fragmentation, MS and MS2 conditions, data acquisition must be included.
- Sample preparation is not described. Methods say the trophs and s/n were separated and proteins harvested. How were the secreted proteins harvested/precipitated from the media? Was this done at Liverpool? No conditions for protein-peptide digestion are given. More information needed.
- Please reference the genomes used for database searching, the release number from giardiadb.org and also what search software/algorithm was used for matching peptides. Please state the search conditions, such as variable and fixed modifications, tolerance, missed cleavages etc etc. This, like the mass spec, needs to be in the manuscript. I have noticed that some of these are given in the description

for the PRIDE data deposit, but these also need to be in a supplementary methods or the methods themselves.
- Judging from the tables the method of quantitation is iBAQ. There is no description of this in the methods or the results. The method for generating these abundance values must be in the methods and results. Some readers may not be familiar with iBAQ, so some explanation of it in general could be valuable to the readership.
- As above, if you are choosing to keep both datasets from the mass spectrometers in the manuscript, then please start their consistency and agreement at the quantitative level of protein abundance as the manuscript only states their agreement at the identification level.
- The top 50 proteins were considered to be the most likely candidates as secreted proteins. What was the ratio cutoff that this included (min/max)? What were the ratios of currently known secreted proteins in Giardia, and could these be used (or at least discussed) as internal standards considered as an alternative method to filter candidates?
- You used three biological replicates, were these used in order to filter for only reproducibly identified proteins? All the supplementary files have data at the average level? How were replicates used in the identification and quantification of proteins? You say minimum 2 peptides were needed, but what if that was found in only one of the replicates?
- You need to provide a supplementary table of your proteomic identifications and quantification for your datasets of the ~1600 proteins.
- The PRIDE deposit contains files, but there are no titles on these files? What are these? They have different numbers for accession but no titles? These are not the raw files? To what level have they been processed? Please clarify what these files are.

b) Culture conditions:
- Please state throughout if you used/harvested adhered or non-adhered or total trophozoites.
- You say the 'reference isolates', please cite the ATCC numbers.
- As you are moving isolates to separate media or co-culture conditions, please state whether the trophozoites were in aerobic or anerobic conditions, including during co-culture.
- There needs to be more information in the methods in regards to the experiment regarding Giardia/Giardia soluble products and the CaCO2 cells and the electrophysiology. In the Data description it says Giardia were added, in the results on page 6 is states cells were grown alone, with Giardia or with diluted supernatants. None of the information about these different conditions, their time frames or experiment design are given in the detailed methods regarding 'electrophysiology'. Not only does this need to be added, but the description of the experiment between Data description, results and methods needs to be made consistent throughout otherwise is it too confusing.

c) Other
- Given that VSPs are known membrane proteins, and you are looking at media fractions, have you considered the possibility they may be cleaved protein products? What sequence coverage are you getting for your VSPs, including the termini? Can you say they are indeed secreted, or could they be VSPs cleaved from the membrane during turnover?

Methods

Are the methods appropriate to the aims of the study, are they well described, and are necessary controls included? No

Conclusions

Are the conclusions adequately supported by the data shown? Yes

Reporting Standards

Does the manuscript adhere to the journal's guidelines on minimum standards of reporting? No

Statistics

Are you able to assess all statistics in the manuscript, including the appropriateness of statistical tests used? Yes, and I have assessed the statistics in my report.

Quality of Written English

Please indicate the quality of language in the manuscript: Acceptable

Declaration of Competing Interests

Please complete a declaration of competing interests, considering the following questions:

- Have you in the past five years received reimbursements, fees, funding, or salary from an organisation that may in any way gain or lose financially from the publication of this manuscript, either now or in the future?
- Do you hold any stocks or shares in an organisation that may in any way gain or lose financially from the publication of this manuscript, either now or in the future?
- Do you hold or are you currently applying for any patents relating to the content of the manuscript?
- Have you received reimbursements, fees, funding, or salary from an organization that holds or has applied for patents relating to the content of the manuscript?
- Do you have any other financial competing interests?
- Do you have any non-financial competing interests in relation to this paper?

If you can answer no to all of the above, write 'I declare that I have no competing interests' below. If your reply is yes to any, please give details below.

I declare that I have no competing interests

I agree to the open peer review policy of the journal. I understand that my name will be included on my report to the authors and, if the manuscript is accepted for publication, my named report including any attachments I upload will be posted on the website along with the authors' responses. I agree for my report to be made available under an Open Access Creative Commons CC-BY license (http://creativecommons.org/licenses/by/4.0/). I understand that any comments which I do not wish to be included in my named report can be included as confidential comments to the editors, which will not be published.

I agree to the open peer review policy of the journal

To further support our reviewers, we have joined with Publons, where you can gain additional credit to further highlight your hard work (see: https://publons.com/journal/530/gigascience). On publication of this paper, your review will be automatically added to Publons, you can then choose whether or not to claim your Publons credit. I understand this statement.

Yes
