# Supplementary material for: Giardia secretome highlights secreted tenascins as a key component of pathogenesis
Source: Gigascience. 2018 Jan 29;7(3):1–13. doi: 10.1093/gigascience/giy003 (PMC5887430; doi:10.1093/gigascience/giy003)
Supplement: Reviewer_1_Report_(Revision_1) -- Samantha Emery — 05 Nov 2017 Reviewed [file giy003_reviewer_1_report_(revision_1).pdf]

Reviewer Report

Title: Giardia Secretome Highlights Secreted Tenascins as a Key Component of Pathogenesis

Version: Revision 1 **Date:** 05 Nov 2017

Reviewer name: Samantha Emery

Reviewer Comments to Author:

Response to Authors:

I would like to congratulate the authors for their extensive revisions. I think this has greatly improved the manuscript and facilitated understanding of their methods. I have a few comments and revisions that I feel the authors should consider prior to final publication.

Firstly, I understand that the reviewers are using both MS platforms (Q-Exactive and LTQ Velos) to increase robustness of their data. I do not think they increase coverage, as they state they are highly correlated, and nearly all of the Velos IDs are also identified by the Q-exactive MS (which is more sensitive and makes sense).
However, given there is little discrepancy between the data from both platforms, that the data is already filtered by reproducibility, and the Q-exactive is the more sensitive machine, might the authors think to keep the extra, reproducible IDs from the Q-exactive rather than discarding them totally because they are not seen on the LTQ Velos? As secreted proteins are going to be low abundance (given they are only being produced and secreted over an hour or so incubation in DMEM), the sensitivity of the Q-exactive may provide additional secreted proteins? Maybe these can at least be provided in an additional supplementary table?

Secondly, given the authors are comparing WB and GS, and that these most likely represent cryptic species with different clinical pathophysiology, maybe the discussion/results could be added to in order discuss differences between assemblages. The authors have already provided a robust set of proteins common between assemblages, and tables of proteins identified exclusively in WB or GS. However, significant differences between assemblages in the proteomics and/or electrophysiology could provide important insights into the differences between these two, human-infective assemblages that is limited in current literature.

Thirdly, while the authors do plan to deposit the data in the EuPath DBs, providing excel supplementary tables of protein IDs, peptide counts and IBAQ values for reproducibly identified proteins is a very valuable resource for other researchers looking to compare identifications and expression values. Some of these people may not be familiar or aware of the EuPATH databases and their systems. I strongly advise providing these for future reviewers, as it will greatly affect the accessibility of your data.

Minor Comments:
Methods:
1) In the non-supplemented DMEM, what was the oxygen levels? Were these capped, filled flasks?
2) What were the centrifugation speeds - higher speeds would have been likely to rupture cells, so it might be good to report this.
3)In the electrophysiology, what are the patient strains? Are these verified assemblage A or B? Have they been typed?

Results:
1) I think that adding some further comments about viability and harvesting contingencies to ensure cell integrity (i.e. cell not popping) would be valuable when reporting supernatant vs pellet IDs around pg 12, ln 42-49. While contamination of the supernatant with proteins is a very difficult thing to avoid in this type of experiment, hence your experimental design in averaging pellet to supernatant ratios, I think showcasing steps to mitigate contamination would add more credibility to the results.
2) Were the supernatants harvested with protease inhibitors at any stage, or how was protease activity controlled during harvesting? SDS-PAGE was mentioned as a quality control step (which can also show whether there is any evidence of protease activity), but pictures of these gels are not provided in the supplementary.

Methods

Are the methods appropriate to the aims of the study, are they well described, and are necessary controls included? Yes

Conclusions

Are the conclusions adequately supported by the data shown? Yes

Reporting Standards

Does the manuscript adhere to the journal's guidelines on minimum standards of reporting? Yes

Statistics

Are you able to assess all statistics in the manuscript, including the appropriateness of statistical tests used? Yes, and I have assessed the statistics in my report.

Quality of Written English

Please indicate the quality of language in the manuscript: Acceptable

Declaration of Competing Interests

Please complete a declaration of competing interests, considering the following questions:

- Have you in the past five years received reimbursements, fees, funding, or salary from an organisation that may in any way gain or lose financially from the publication of this manuscript, either now or in the future?
- Do you hold any stocks or shares in an organisation that may in any way gain or lose financially from the publication of this manuscript, either now or in the future?
- Do you hold or are you currently applying for any patents relating to the content of the manuscript?
- Have you received reimbursements, fees, funding, or salary from an organization that holds or has applied for patents relating to the content of the manuscript?
- Do you have any other financial competing interests?
- Do you have any non-financial competing interests in relation to this paper?

If you can answer no to all of the above, write 'I declare that I have no competing interests' below. If your reply is yes to any, please give details below.

I declare I have no competing interests

I agree to the open peer review policy of the journal. I understand that my name will be included on my report to the authors and, if the manuscript is accepted for publication, my named report including any attachments I upload will be posted on the website along with the authors' responses. I agree for my report to be made available under an Open Access Creative Commons CC-BY license (http://creativecommons.org/licenses/by/4.0/). I understand that any comments which I do not wish to be included in my named report can be included as confidential comments to the editors, which will not be published.

I agree to the open peer review policy of the journal

To further support our reviewers, we have joined with Publons, where you can gain additional credit to further highlight your hard work (see: https://publons.com/journal/530/gigascience). On publication of this paper, your review will be automatically added to Publons, you can then choose whether or not to claim your Publons credit. I understand this statement.

Yes
