# Supplementary material for: Giardia secretome highlights secreted tenascins as a key component of pathogenesis
Source: Gigascience. 2018 Jan 29;7(3):1–13. doi: 10.1093/gigascience/giy003 (PMC5887430; doi:10.1093/gigascience/giy003)
Supplement: Reviewer_2_Report_(Original_Submission) -- Feng Xue — 14 Jul 2017 Reviewed [file giy003_reviewer_2_report_(original_submission).pdf]

Reviewer Report

Title: Giardia Secretome Highlights Secreted Tenascins as a Key Component of Pathogenesis

Version: Original Submission **Date:** 14 Jul 2017

Reviewer name: Feng Xue

Reviewer Comments to Author:

This manuscript revealed Giardia secretome under steady state of growth in vitro by proteomics methods and also confirmed that the secreted molecules adversely affect the homeostasis of enteric epithelia. The authors also concluded that Tenascins was a new class of virulence factors. This research provided a comprehensive proteomic data on Giardia secretome, introduced a novel pathogenicity mechanism, therefore is worthy of publication. However, there are still some problems needed to be revised before acceptance.

1. More comparison between this research and previous Giardia proteomics research should be discussed , especially on the topic of whether the parasite-host cell interaction can induce the proteomic changes of the parasite.
2. The major advantage of this research is that Tenascins was defined as a new class of virulence factor of Giardia. Considering that this is the major innovation and proposed novel mechanism of pathogenicity for Giardiao, the authors should try to express them in 293T cells, insect cells or in vitro none-cell cell lysis system to analyze the pathological function of the recombinant proteins. This results will strongly support the Figure 3 novel mechanism proposal.
3. Figure 1 is not necessary and can be transfered as a supplement figure. Other analysis, such as alignment of protein function domains, signaling peptide prediction also can be included in this figure. To support the proposed novel mechanism of pathogenicity, the authors should pay more attention on the protein functions, such as function domain, 2D and 3D structure, etc.

Methods

Are the methods appropriate to the aims of the study, are they well described, and are necessary controls included? Yes

Conclusions

Are the conclusions adequately supported by the data shown? No

Reporting Standards

Does the manuscript adhere to the journal’s guidelines on minimum standards of reporting? Yes

Statistics

Are you able to assess all statistics in the manuscript, including the appropriateness of statistical tests used? Yes, and I have assessed the statistics in my report.

Quality of Written English

Please indicate the quality of language in the manuscript: Acceptable

Declaration of Competing Interests

Please complete a declaration of competing interests, considering the following questions:

- Have you in the past five years received reimbursements, fees, funding, or salary from an organisation that may in any way gain or lose financially from the publication of this manuscript, either now or in the future?
- Do you hold any stocks or shares in an organisation that may in any way gain or lose financially from the publication of this manuscript, either now or in the future?
- Do you hold or are you currently applying for any patents relating to the content of the manuscript?
- Have you received reimbursements, fees, funding, or salary from an organization that holds or has applied for patents relating to the content of the manuscript?
- Do you have any other financial competing interests?
- Do you have any non-financial competing interests in relation to this paper?

If you can answer no to all of the above, write 'I declare that I have no competing interests' below. If your reply is yes to any, please give details below.

I declare that I have no competing interests

I agree to the open peer review policy of the journal. I understand that my name will be included on my report to the authors and, if the manuscript is accepted for publication, my named report including any attachments I upload will be posted on the website along with the authors' responses. I agree for my report to be made available under an Open Access Creative Commons CC-BY license (http://creativecommons.org/licenses/by/4.0/). I understand that any comments which I do not wish to be included in my named report can be included as confidential comments to the editors, which will not be published.

I agree to the open peer review policy of the journal

To further support our reviewers, we have joined with Publons, where you can gain additional credit to further highlight your hard work (see: https://publons.com/journal/530/gigascience). On publication of

this paper, your review will be automatically added to Publons, you can then choose whether or not to claim your Publons credit. I understand this statement.

Yes
