# Supplementary material for: Giardia secretome highlights secreted tenascins as a key component of pathogenesis
Source: Gigascience. 2018 Jan 29;7(3):1–13. doi: 10.1093/gigascience/giy003 (PMC5887430; doi:10.1093/gigascience/giy003)
Supplement: Reviewer_3_Report_(Original_Submission) -- Sebastien Charneau — 21 Jul 2017 Reviewed [file giy003_reviewer_3_report_(original_submission).pdf]

Reviewer Report

Title: Giardia Secretome Highlights Secreted Tenascins as a Key Component of Pathogenesis

Version: Original Submission **Date:** 21 Jul 2017

Reviewer name: Sebastien Charneau

Reviewer Comments to Author:

From the biological point of view, the results are sounding and interesting. The manuscript is well presented. The choices of methods and accuracy of the procedure are globally good; the comprehensive of description of methods must be detailed. The authors definitively identify many proteins using mass spectrometry, some of which may be important for the interaction of Giardia lamblia with host cell. The manuscript is lacking any confirmation of these secreted proteins by another biological technique; however, that may not be trivial given the potential lack of antibodies.

However, I have pointed out below several concerns I have regarding this publication. The scientific content of this manuscript is interesting. It is advised to correct all the typing errors (Some corrections that I found, I indicated) and to have a last editing of the manuscript. I suggest improving the quality of the figures (as 1, 3). Please do not write protein name with uppercase (for example: replace some Cathepsin by some cathepsin).

Considering all together I can state that several points need clarification or revision before final decision.

ABSTRACT
Please change "N' terminus signal peptides" by "N-terminal signal peptide" and in the main text as well.

ANALYSES

5/3: The use of expression is not appropriate here, in the main text and in the suppl. table. It is not "expression" but "protein abundance". Moreover, the term "SP expression" does not sound good to explain the more abundant proteins identified from the supernatant.

The authors should explain what they used to do the ratio SP/P to compare the "abundance" of proteins. What did the value considered? Did they use the PSMs? In fact, you used iBAQ algorithm but it is not mentioned in the main text.

DISCUSSION
The authors could discussed about the high number of proteins from the supernatants; why they identified some proteins only from the supernatants since proteins are synthetized in the cell; and why they used two different mass spectrometers and they could justify the choice of these 2 spectrometers in function of the different results.

The authors discussed the function of the more abundant secreted proteins, but did they find other proteins known secreted that could be confirm these results?

METHODS

it should be indicated the origin of the strains, and if the parasites are subjected or not to regular passages in animal (for example mice).

The Proteomic Analysis must be detailed: Cell lysis; Protein trypsin digestion; Peptide separation by reverse-phase chromatography; Mass spectra acquisition for both mass spectrometers; data analysis (.raw and protein interpretation).

Did the authors work with the same number of cells from each strain? Quantity of proteins and peptides? How did the authors quantify the proteins and peptides?

9/15: should change "SDS page electrophoresis and..." by "SDS PAGE and..."
9/21: What the cut-off of the vivaspin filter? 5000 MWCO?
change Ambic by ammonium bicarbonate
9/28 and other place: should change giardiaDB.org by giardiaDB.org
9/47 and 10/10: should change "mls" by "ml".
10/6: Consider the following sentence: "...GlyH-101 (50mM) and" what?
10/27: should write Giardia in italic

10/32: Abbreviations are incomplete, for example FDR

14/7: Delete S in the sentence: "…Q-Exactive S Supernatant (S) abundance, from most to least abundant…"

Methods

Are the methods appropriate to the aims of the study, are they well described, and are necessary controls included? No

Conclusions

Are the conclusions adequately supported by the data shown? Yes

Reporting Standards

Does the manuscript adhere to the journal's guidelines on minimum standards of reporting? Yes

Statistics

Are you able to assess all statistics in the manuscript, including the appropriateness of statistical tests used? No, I do not feel adequately qualified to assess the statistics.

Quality of Written English

Please indicate the quality of language in the manuscript: Acceptable

Declaration of Competing Interests

Please complete a declaration of competing interests, considering the following questions:

- Have you in the past five years received reimbursements, fees, funding, or salary from an organisation that may in any way gain or lose financially from the publication of this manuscript, either now or in the future?
- Do you hold any stocks or shares in an organisation that may in any way gain or lose financially from the publication of this manuscript, either now or in the future?
- Do you hold or are you currently applying for any patents relating to the content of the manuscript?
- Have you received reimbursements, fees, funding, or salary from an organization that holds or has applied for patents relating to the content of the manuscript?
- Do you have any other financial competing interests?
- Do you have any non-financial competing interests in relation to this paper?

If you can answer no to all of the above, write 'I declare that I have no competing interests' below. If your reply is yes to any, please give details below.

I declare that I have no competing interests’

I agree to the open peer review policy of the journal. I understand that my name will be included on my report to the authors and, if the manuscript is accepted for publication, my named report including any attachments I upload will be posted on the website along with the authors' responses. I agree for my report to be made available under an Open Access Creative Commons CC-BY license (http://creativecommons.org/licenses/by/4.0/). I understand that any comments which I do not wish to be included in my named report can be included as confidential comments to the editors, which will not be published.

I agree to the open peer review policy of the journal

To further support our reviewers, we have joined with Publons, where you can gain additional credit to further highlight your hard work (see: https://publons.com/journal/530/gigascience). On publication of this paper, your review will be automatically added to Publons, you can then choose whether or not to claim your Publons credit. I understand this statement.

Yes
